# DIVERSITY in binding, regulation, and evolution revealed from high-throughput ChIP

**DOI:** 10.1371/journal.pcbi.1006090

**Published:** 2018-04-23

**Authors:** Sneha Mitra, Anushua Biswas, Leelavati Narlikar

**Affiliations:** Department of Chemical Engineering, CSIR-National Chemical Laboratory, Pune, India; bioinformatics, GERMANY

## Abstract

Genome-wide in vivo protein-DNA interactions are routinely mapped using high-throughput chromatin immunoprecipitation (ChIP). ChIP-reported regions are typically investigated for enriched sequence-motifs, which are likely to model the DNA-binding specificity of the profiled protein and/or of co-occurring proteins. However, simple enrichment analyses can miss insights into the binding-activity of the protein. Note that ChIP reports regions making direct contact with the protein as well as those binding through intermediaries. For example, consider a ChIP experiment targeting protein X, which binds DNA at its cognate sites, but simultaneously interacts with four other proteins. Each of these proteins also binds to its own specific cognate sites along distant parts of the genome, a scenario consistent with the current view of transcriptional hubs and chromatin loops. Since ChIP will pull down all X-associated regions, the final reported data will be a union of five distinct sets of regions, each containing binding sites of one of the five proteins, respectively. Characterizing all five different motifs *and* the corresponding sets is important to interpret the ChIP experiment and ultimately, the role of X in regulation. We present diversity which attempts exactly this: it partitions the data so that each partition can be characterized with its own de novo motif. Diversity uses a Bayesian approach to identify the optimal number of motifs and the associated partitions, which *together* explain the entire dataset. This is in contrast to standard motif finders, which report motifs *individually* enriched in the data, but do not necessarily explain all reported regions. We show that the different motifs and associated regions identified by diversity give insights into the various complexes that may be forming along the chromatin, something that has so far not been attempted from ChIP data. Webserver at http://diversity.ncl.res.in/; standalone (Mac OS X/Linux) from https://github.com/NarlikarLab/DIVERSITY/releases/tag/v1.0.0.

## Author summary

A high-throughput chromatin immunoprecipitation (ChIP) experiment identifies genomic regions bound by a protein in vivo. Current motif-discovery approaches seek an enriched motif signature in the reported regions, which they can attribute to the protein’s binding preferences. However, Diversity models the fact that since a ChIP experiment pulls down regions participating in all complexes involving the profiled protein, the reported regions are in all likelihood, a collection of different types of protein-DNA contacts. Diversity asks a different question: *what sequence component caused a specific region to be reported in a ChIP experiment?* The answer, in combination with additional data such as sequence conservation, SNPs, chromatin structure, downstream gene-expression, etc. can yield insights into the diverse regulatory mechanisms at play. The added benefits of a webserver and a standalone parallel version make diversity a practical tool for discovering new biology from ChIP experiments.

This is a *PLoS Computational Biology* Software paper.

## Introduction

Transcriptional regulation is a complex cellular process, governed in large part by interactions between chromatin remodeling complexes, transcription factors (TFs), and specific sequences on the DNA. The importance of these sequences, also known as regulatory regions, has been well-documented in various biological processes such as development, differentiation, maintenance, and apoptosis [1, 2]. Therefore, to better understand the role of these regions, millions of dollars have been spent by the ENCyclopedia Of DNA Elements (ENCODE) consortia and other laboratories to measure a wide range of regulation-related biochemical activities, genome-wide [3].

**Fig 1:**
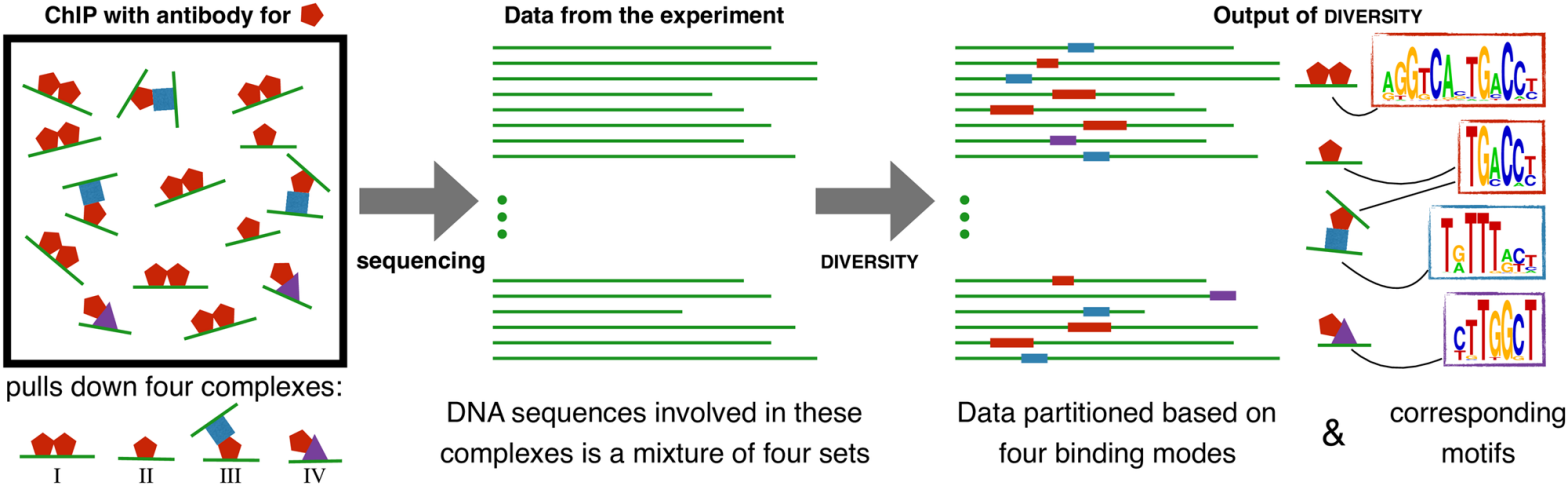
Overview of DIVERSITY. A ChIP experiment pulls down all complexes of which the profiled protein is a component. Sequencing therefore reports all DNA regions participating in these complexes. Diversity splits the regions into different sets based on motifs common to each set, while simultaneously learning the motifs, de novo. In this toy example, the red protein binds to DNA as (I) homodimer, (II) monomer, (III) indirectly to another DNA region via the blue protein, or (IV) indirectly to yet another DNA region via the purple protein, making no direct DNA contact this time. The expected output of diversity in this case are four causes represented as motifs: (a) the palindromic site corresponding to I, (b) half-site corresponding to II and the direct site of III, (c) site of the blue protein in III, and (d) site of the purple protein in IV.

However, in spite of these efforts, we still do not know how regulatory information is encoded in the four-letter “alphabet” of our genome [4]. We attribute this to the manner in which data from high-throughput experiments are currently interpreted and modelled. Although evidence points towards multiple distinct regulatory mechanisms being at play at any given point in time [5, 6], a common characteristic is nevertheless sought from the data. Motif finding is one such glaring example: a common sequence signature, typically a position weight matrix (PWM) [7], is learned from protein-DNA binding data or promoters of coregulated genes, under the assumption that the solution must be “overrepresented” in the full set. However, a TF can exert its influence on the DNA in more than one way, by changing co-factors, or through intermediaries, and at times, never making direct DNA contact [8]. In other words, it can adopt different configurations at different DNA locations causing the dataset to be highly diverse (Fig 1). Deciphering these configurations is key towards understanding the role of the protein in chromatin organization and gene regulation [8, 9].

We present diversity, a method that appreciates the fact that since a ChIP experiment pulls down regions participating in all complexes involving the profiled protein (Fig 1), it may report sequences that are a collection of different types of protein-DNA contacts. Diversity assumes the protein can make *m* types of contacts, each of which is modeled with its own PWM. Formulating the problem as a mixture model, it aims to split the complete dataset into *m* disjoint subsets, corresponding to the *m* contact types or modes of binding, with one PWM enriched in each subset. Neither the split nor the PWMs are known apriori. Both are inferred using a sampling-based approach. Several models with different values of *m* are learned and the best *m* is identified using Bayesian model selection.

## Design and implementation

### Overview

diversity builds upon our earlier work where we showed that ChIP-data contains multiple modes of TF-DNA binding [10]. There too each model was a mixture of modes as described above, but the structure of the model varied with the width of each motif. As a result, the number of distinct models to be learned grew exponentially with the number of widths under consideration. Therefore it was not feasible to run it for more than a few (typically six) modes or large datasets. These limitations are overcome in diversity through three algorithmic advances. First, diversity learns the width of each contact type, or PWM, during the sampling process, instead of relying on a set of distinct widths decided a priori. Second, it uses an improved procedure for identifying convergence, which is usually a confounding factor in sampling-based optimization methods. Both of these advances are described in greater detail below. Finally, it has a parallel implementation, making use of multiple cores, which is now standard in all computers. Models are learned in parallel using methods written in C. A Python wrapper is used to control multiprocessing.

### Model description

The input to diversity are the *n* DNA sequences ***X***_1_ …***X***_*n*_, reported by ChIP. *X*_*i*, *j*_ ∈ {A, C, G, T, N}: 1 ≤ *j* ≤ *L*_*i*_, where *L*_*i*_ is length of *X*_*i*_. When searching for *m* modes, we learn a model ***M***_*m*_ with parameters ***θ***_*m*_. ***θ***_*m*_ = {***Z***, ***I***, ***w***, ***ϕ***, ***γ***}, where:

- *Z*_*i*_: the position of the motif in ***X***_*i*_
- *I*_*i*_: the binding mode in ***X***_*i*_; 1 ≤ *I*_*i*_ ≤ *m*
- *w*_*k*_: the width of PWM of mode *k*; 1 ≤ *k* ≤ *m*
- ***ϕ***^*k*^: PWM parameters of mode *k*;
  $\phi_{a,b}^{k}$ is the probability of finding base *b* at position *a* of PWM *k*
- ***ϕ***^0^: parameters of the background probability distribution
  (2nd order Markov model learned from ***X***)
- ***γ***: categorical distribution over the *m* modes;
  *γ*_*k*_ is the probability of a sequence containing mode *k*

For any ***θ***_*m*_ the likelihood for a sequence *X*_*i*_ can be computed as:

$$\begin{array}{c} P\left(X_{i}|\theta_{m},M_{m}\right)=P\left(X_{i,1},\ldots ,X_{i,Z_{i}-1}|\phi^{0}\right)\times \prod_{a=1}^{w_{I_{i}}}\phi_{a,X_{i,Z_{i}+a-1}}^{I_{i}}\times P\left(X_{i,Z_{i}+w_{I_{i}}},\ldots ,X_{i,L_{i}}|\phi^{0}\right) \end{array}$$ (1)

and the full likelihood and the posterior distributions are, respectively:

$$\begin{array}{c} P\left(X|\theta_{m},M_{m}\right)=\prod_{i=1}^{n}P\left(X_{i}|\theta_{m},M_{m}\right) \end{array}$$ (2)

$$\begin{array}{c} P\left(\theta_{m}|X,M_{m}\right)\propto P\left(X|\theta_{m},M_{m}\right)\times P\left(\theta_{m}|M_{m}\right) \end{array}$$ (3)

All components of ***θ***_*m*_ except the background parameters ***ϕ***^0^ are learned with the aim of maximising (3) using collapsed Gibbs sampling [11]: each of *Z*_*i*_ and *I*_*i*_ are sampled iteratively based on their conditional distributions by integrating out ***ϕ***^*k*^ and ***γ*** as before [10]. Each sampling run is executed from a default of five random initial positions, although this value can be changed by the user.

Since we do not expect the value of *m* to be known apriori, we learn models with different values of *m*. Theoretically a model with more modes will never do worse than one with fewer if the posterior distribution value from (3) is used to compare them. But this can lead to overfitting, which we avoid by using Bayesian model selection and maximising:

$$\begin{array}{ccc} \underset{M_{m}}{\text{arg}\;\text{max}}\;P\left(M_{m}|X\right) & = & \underset{M_{m}}{\text{arg}\;\text{max}}\;P\left(M_{m}\right)\cdot P\left(X|M_{m}\right)\\ & = & \underset{M_{m}}{\text{arg}\;\text{max}}\;P\left(M_{m}\right)\cdot \\ & & \int_{\theta_{m}}P\left(X|\theta_{m},M_{m}\right)P\left(\theta_{m}|M_{m}\right)d\theta_{m}^{w} \end{array}$$ (4)

The prior on the model is exponential in the number of free parameters within the model. It therefore penalizes models with more parameters as before [10]:

$$\begin{array}{c} P\left(M_{m}\right)\propto \exp(-\lambda |M_{m}\left|\right) \end{array}$$ (5)

We use a λ of 5, although a Biologist might find it worthwhile to view the different models, which are anyway reported by diversity. The integral in (4) is approximated by the maximum a posteriori probability (MAP) estimate of ***θ***_*m*_ (from (3)).

#### Width sampling

In our original method, a model was defined with *m* as well as the vector of values for the widths *w*_1_, *w*_2_, …, *w*_*m*_. As a result, exponentially many more models had to be learned by iterating over all “reasonable” values of ***w***. This not only limited the method to smaller datasets, but could never identify motifs with arbitrary lengths. Diversity models the widths as parameters of the model instead of as the structure of the model. The widths are sampled along with the other parameters. For each mode *k*, *w*_*k*_ is sampled from a pool of a few values: it is allowed to not change, or increase/decrease by one position on the left and right of the motif.

#### Convergence of the sampler

Detecting convergence of a sampler is non trivial, especially when the target distribution is irregular. But we do not really need samples from the posterior, we only want the MAP estimate. We fit a linear curve through the last *n* iterations and stop sampling if the slope is close to zero. We also have a check in place for the number of *Z*_*i*_ & *I*_*i*_ sampling iterations exceeding 2*n*^2^, to ensure the program does report a model in reasonable time in case of a particularly unlucky initialisation. But the user has an option to increase (or decrease) this maximum iterations limit, if time is to be traded for more accurate results (or vice versa). At the end, we use a hill climbing approach starting from the sample with highest posterior probability.

### Input and output

Diversity takes as input a fasta file corresponding to ChIP-bound regions. The webserver also allows the input to be a bed file, in which case the reference genome has to be selected from a drop-down menu. Diversity can be run with several additional options such as changing the range of the motif width, number of modes, and many more. See Documentation (S1 File) for more details.

Diversity returns details of all models learnt in an html file which, for each model, links to: a table containing the identity of the mode in each sequence (*I*_*i*_) and position of the site (*Z*_*i*_), as well as a text file with all the mode parameters (***ϕ***) and corresponding sequence logos. It also calculates and reports the best model amongst all.

**Fig 2:**
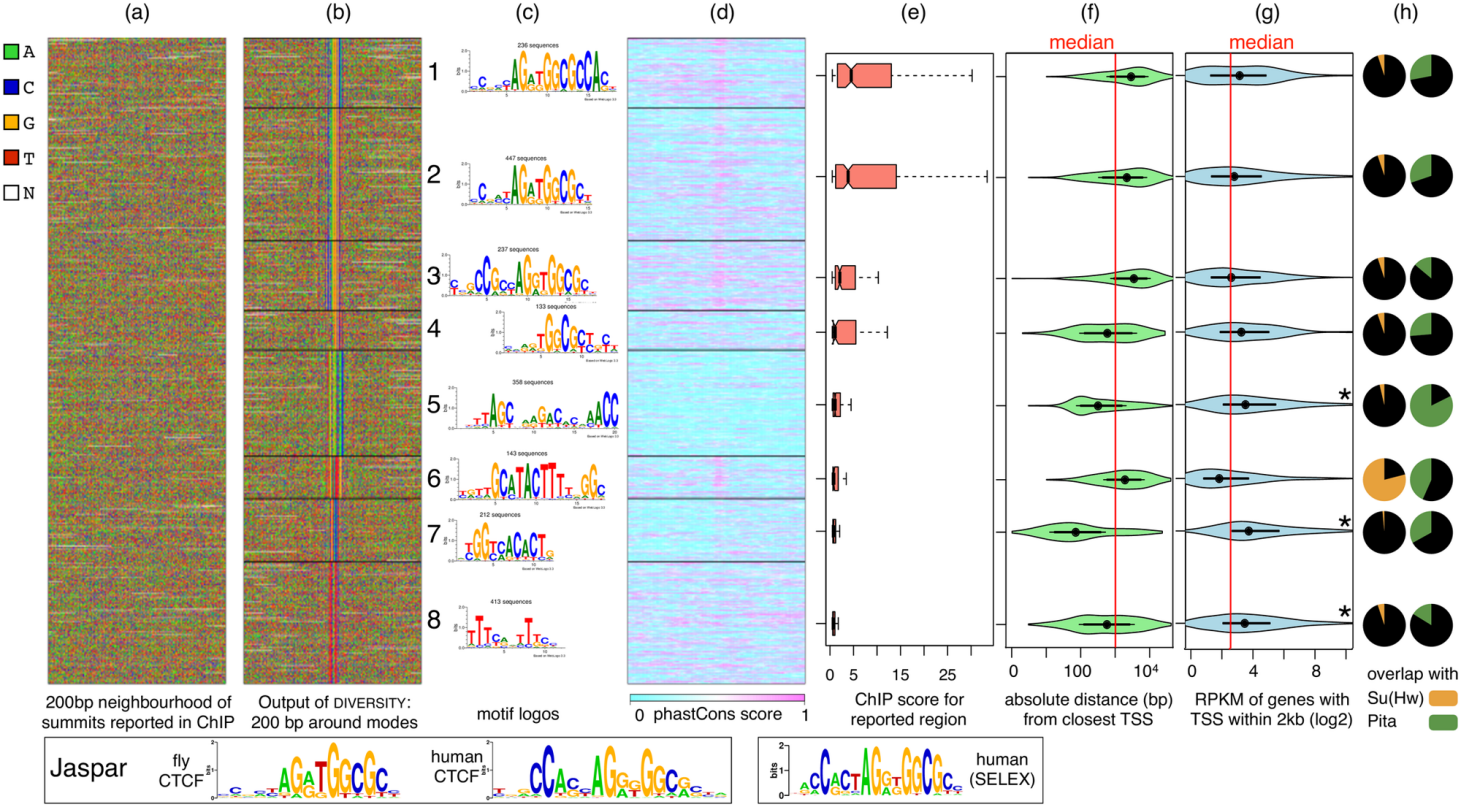
DIVERSITY finds multiple modes in fly CTCF ChIP data. (a) 200bp regions centered around the summit of ChIP peaks, input to diversity. (b) Diversity reorders and realigns the data, revealing eight modes. (c) Motifs corresponding to modes. CTCF motifs from JASPAR and from high throughput SELEX [25] are shown below. (d) Sequence conservation profile from phastCons, corresponding to nucleotides in b (e) The eight modes are displayed in decreasing ChIP score. (f) Violin plot of distance of each sequence from the closest transcription start site. (g) Violin plot of expression values of genes (log2(1+RPKM)) with TSS within 2000bp of the ChIP region. Red line shows the median value across all measured genes. (h) Overlaps with Su(Hw) and Pita ChIP experiments, respectively.

In the webserver, if the input is in the form of a bed file, then along with the above output, following additional images are created: aligned motifs based on their midpoint per sequence, phastCons scores at the sequences, and boxplots of distances between the modes and the closest transcription start site (TSS), similar to Fig 2.

### Datasets

All data are publicly available, accession numbers from GEO are mentioned in parentheses. Fly CTCF ChIP and RNA-seq data (GSE24449) is from Negre et al. [12]. Su(Hw) data (GSE23537) is from the same white pre-pupa stage [13]. Pita data (GSE76997) is from 0–12h embryos [14]. Human REST ChIP data (GSE32465,GSE49570) and neuronal RNA-seq data (GSE46562) is as processed and compiled by Rockowitz et al. [15]. All other human TF data are narrowpeak files from ENCODE in K562 (ENCFF144DMD, ENCFF264QLP, ENCFF440KMN, ENCFF443TUR, ENCFF503LMD, ENCFF529CTW, ENCFF484BSF, ENCFF433PKW, ENCFF602YIK, ENCFF886EVL). In all cases, a 200bp neighborhood around the summit (or center where summit was not reported) of reported ChIP regions is used as input. Regions where more than 150bp were repetitive nucleotides were ignored, based on repeatMasker as per UCSC genome browser [16]. Diversity was run with default parameters except for an increased number of sampling start points (10 instead of the default five) and models with modes in the range of 1 to 20. PhastCons scores were used to assess sequence conservation and refGene.txt for gene analysis from UCSC genome browser. The ChIP signal for each region as reported by the respective studies was considered the ChIP score for that sequence when constructing plots. JASPAR [17] motifs are shown for comparison where available. TOMTOM [18] was used to identify potential TFs binding the motifs. These motifs are from well-established databases and are at times constructed from ChIP data, but never from the sets used here. Nucleosome occupancy for GM12878 is from ENCODE [3]. Weblogo [19] has been used extensively throughout this paper and in diversity to create logos of the PWMs. All *p*-value based comparisons across sets are done using the Wilcoxon test. Violin plots were constructed using the vioplot package of R.

### Other programs

MEME [20] was run with the following optional parameters: -nmotifs 20 -minw 6 -revcomp -p 16. (S1 and S6 Figs)

DREME [21] was run with the following optional parameters: -maxk 20 -png. The maximum motif width was set to 20 to give it a fair chance of finding the full RE1 site. (S7 Fig)

InMoDe [22] was run in the flexible mode by setting the width to 20, motif orders to 0 (no dependencies), and number of modes as determined by diversity to be optimal when it too was run with a fixed width of 20. Diversity was therefore run twice, once where the motif-width is allowed to vary (standard) and once when it was fixed to 20, only to compare with InMoDe. (S8 Fig)

## Results

### CTCF in the fly makes diverse contacts

The CCCTC-binding factor, CTCF, is a highly conserved DNA-binding protein proven to have diverse roles in transcriptional regulation [23]. Previous work has indicated several dependencies within its 20bp binding site [10, 24] in mammals. To explore whether similar dependencies exist in invertebrates, we looked at data from the fruit fly. Ni et al. [12] have profiled CTCF in the white pre-pupa developmental stage across four related *Drosophila* species. They find a 9 bp core motif AGSKGGCGC to be enriched based on MEME [20] in each species, implying that the binding specificity of CTCF has not evolved across those flies. This motif is found when MEME is supplied a motif width of 9 as a parameter and it explains approximately half of the dataset. Diversity was run on all four CTCF sets. Fig 2 shows the *D. melanogaster* input to Diversity and its output. It finds eight different modes, displayed based on the median ChIP binding score (Fig 2b, 2c and 2e). Modes 1 and 2 have a similar ChIP score, although only mode 2 has been reported as the fly CTCF consensus. Mode 1 has an additional CAC at the 3’ end. Interestingly, mode 3 resembles the human CTCF motif with the CC at the 5’ end, but has a significantly lower ChIP score compared to modes 1 and 2. Unsurprisingly, modes 1–4 explain about half of the sequences.

Modes 5, 6, 7, and 8 have a lower ChIP score than the first four modes and have no resemblance to any CTCF literature motif, suggesting these may not be direct binding sites. Instead, mode 5 matches the motif of a newly discovered insulator protein Pita [26], mode 6 matches suppressor of hairy wing—Su(Hw)—a transcriptional repressor, and mode 7 matches a known fly promoter element [27]. Mode 8 does not appear in any of the standard TF databases. Surprisingly, the eight modes have different evolutionary profiles as evident from phastCons scores (Fig 2d). Modes 5 and 6 have opposite profiles, not only in terms of sequence conservation, but also in terms of functional conservation: mode 5 does not show up in *D. pseudoobscura*, which is farthest from *D. melanogaster* in the evolutionary tree, but is found in *D. simulans* and *D. yakuba*. In contrast mode 6 appears in each of the four flies (S3 Fig). This suggests that the partnership of CTCF with Pita is specific to the *melanogaster* subgroup, while that with Su(Hw) is not.

The first three CTCF modes are far more variable in terms of where they bind along the genome with respect to gene (Fig 2f), which is typical of proteins exhibiting barrier or enhancer-binding function. Mode 4, on the other hand, is more proximal to promoters and is also less conserved across the four species, suggesting this may be a non-functional artifact of more open regions being bound by the profiled protein and captured by ChIP. But additional evidence would be needed to be certain.

Mode 7 is a well-established core-promoter motif, with almost half of the instances occurring within 100bp of a TSS in the reported relative orientation [28]. Furthermore, the downstream genes are significantly more expressed (Fig 2g; *p*-value< 10^−15^), suggesting that CTCF possibly activates transcription of these genes, by indirectly binding to the transcription initiation machinery assembling at these promoters. This could admittedly be a case of highly expressed promoters getting reported in the ChIP experiment, which are not specific to the profiled TF (Discussion) [29]. But even if that were true, it is still interesting that the CTCF ChIP-seq reports only those promoters that contain this particular element out all the several different well-established fly promoter architectures [28].

### Variations in Su(Hw) binding specificities

We next explored ChIP datasets of the co-factors of CTCF identified from Fig 2c, based on motif matches with the JASPAR database. Su(Hw), a zinc finger protein instrumental in chromatin organization [30], has been profiled as part of modENCODE [13] in the same developmental stage. Only sequences of CTCF mode 6 have a significant (≈ 80%) overlap with this experiment (Fig 2h). Further, the CTCF motif is not one of the 10 modes identified by diversity on the Su(Hw) set (S3 Fig). This suggests that the Su(Hw)-CTCF contact might be like complex IV in Fig 1: where the hexagon is CTCF and the triangle is Su(Hw). Alternatively, CTCF may be bridging multiple Su(HW)-DNA binding events, but not making direct DNA contact in the process. In any of these situations, all regions in the complex will be pulled down in both CTCF-ChIP as well as Su(Hw)-ChIP, but will only contain contacts of Su(Hw), not CTCF. However, the CTCF-ChIP will additionally report regions where CTCF does bind DNA directly, since the ChIP is against CTCF.

**Fig 3:**
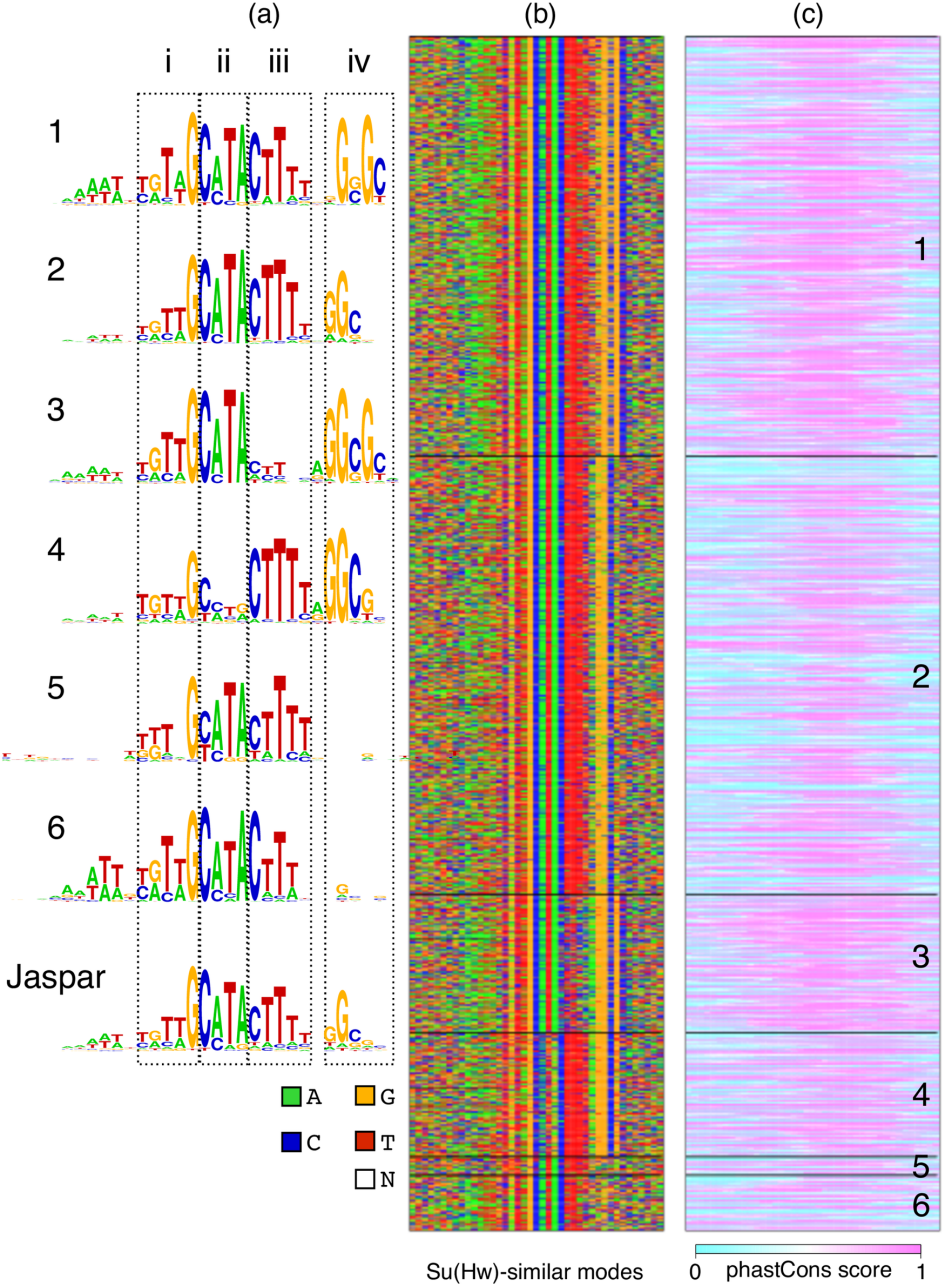
DIVERSITY finds six variants of Su(Hw) motifs, all highly conserved. (a) Logos, (b) sequences, and (c) phastCons scores corresponding to the Su(Hw)-like motifs. Modes 3 and 4 have strikingly complementary sequence information at pieces ii and iii, but are similarly conserved.

More surprisingly, diversity discovers six variants of the known Su(Hw) motif (Fig 3), of which mode 2 is most similar to the database motif. The ChIP scores are not significantly different across these modes (S3 Fig). We can split the motif into 4 pieces, based on the places where the variations occur. Piece i is invariant across the modes, while piece iv is the most variable. Indeed, binding sites of zinc finger proteins are known to have interdependent effects within positions [31]. Considering that individual zinc fingers interact with three or four consecutive nucleotides [32], variations in modes 3 and 4 are specially intriguing. We propose that the different zinc fingers of Su(Hw) bind two distant regions on the chromosome, one belonging to mode 3 and other to mode 4. Three pieces of evidence support this. First, the two modes are complementary in terms of information content at pieces ii and iii. Second, the number of sequences corresponding to the two modes is almost equal, suggesting that each region from mode 3 might have an interacting partner in mode 4. And finally and most importantly, if these were simply “weak” or non-consensus binding sites for Su(hw), the low information pieces would be under neutral selection. But that is not the case: the average conservation scores at the two pieces in both the modes is no different from the scores at the literature consensus, all under negative selection. This suggests the organism prefers non-consensus pieces in these modes, possibly ensuring that some zinc fingers are free to make contact with the corresponding “missing” piece at a different location.

### Pita interacts with CTCF and Su(Hw)

**Fig 4:**
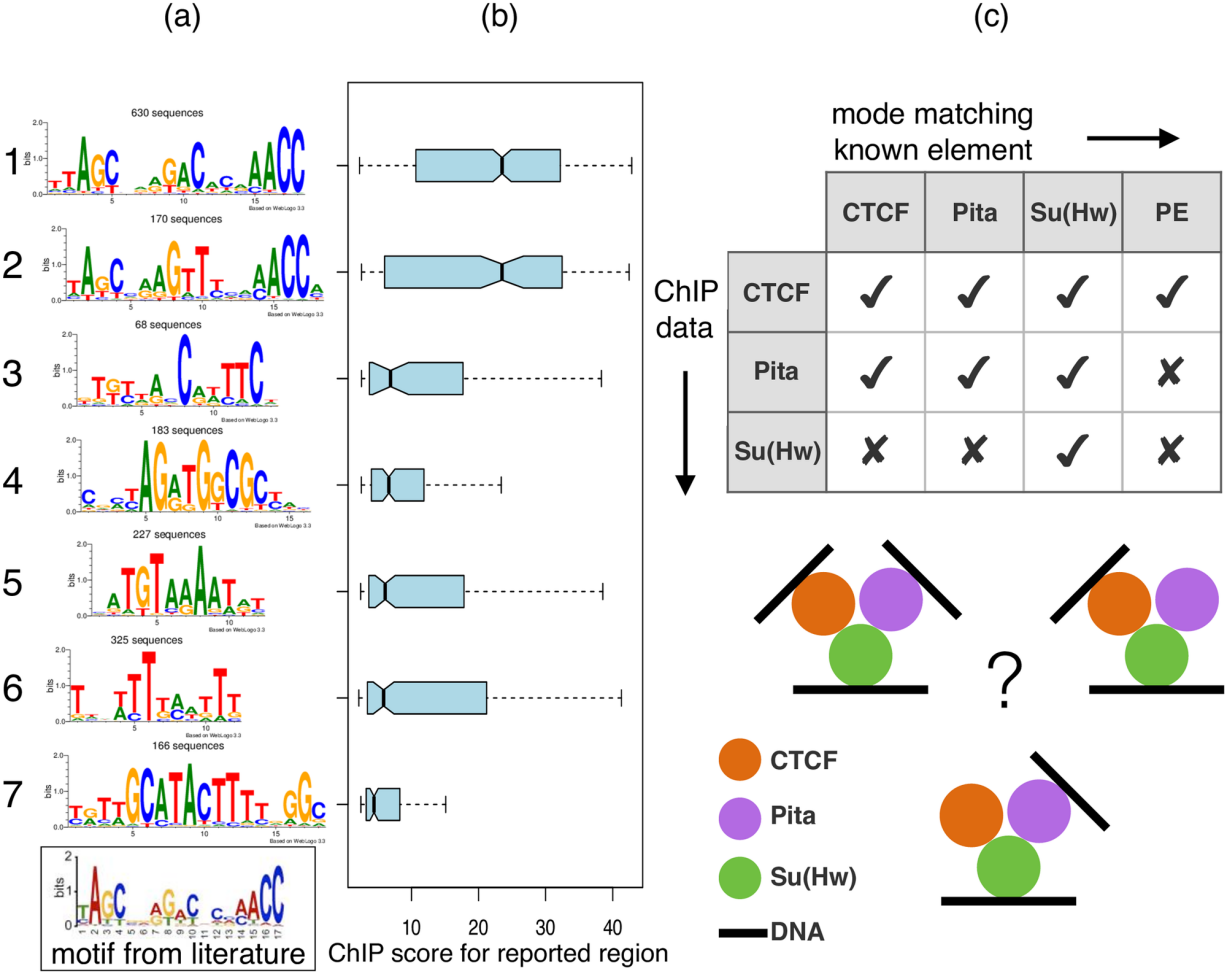
DIVERSITY finds cofactors of Pita and provides insights into chromatin complexes. (a) In addition to a novel Pita variant, diversity identifies the CTCF and Su(Hw) motifs. The literature motif is a result of conventional motif discovery on the same dataset [14]. (b) Both Pita motifs have a significantly high ChIP score. (c) Table describes the various known direct binding motifs identified in each of the three datasets.

We next explored the other potential CTCF co-factor, Pita, based on mode 5. A newly identified TF, also a C2H2-type zinc finger, it has not been profiled in the same very early stage of development, but in 0-12h embryos [14]. Diversity finds the literature Pita motif (mode 1), but also an additional variant, with the central piece differing in a fifth of sequences (Fig 4a). The protein may have a different structural conformation at those regions. The ChIP score, however, is not different across the modes (Fig 4b).

Given the overlaps of this Pita set with the CTCF modes (Fig 2h), it is not surprising that diversity finds CTCF and Su(Hw) motifs (modes 4 and 7, respectively). Taken together, this means, a CTCF ChIP pulls down direct sites of Pita and Su(Hw), a Pita ChIP pulls down direct sites of CTCF and Su(Hw), but Su(Hw) does not pull either sites of the other two (Fig 4a). While additional experiments are needed to ascertain this, one possible explanation could be that Su(Hw), interacts with CTCF and Pita as part of one or more complexes, but those complexes do not make direct DNA contacts at CTCF or Pita binding sites.

### REST has many co-factors in neuronal cells

**Fig 5:**
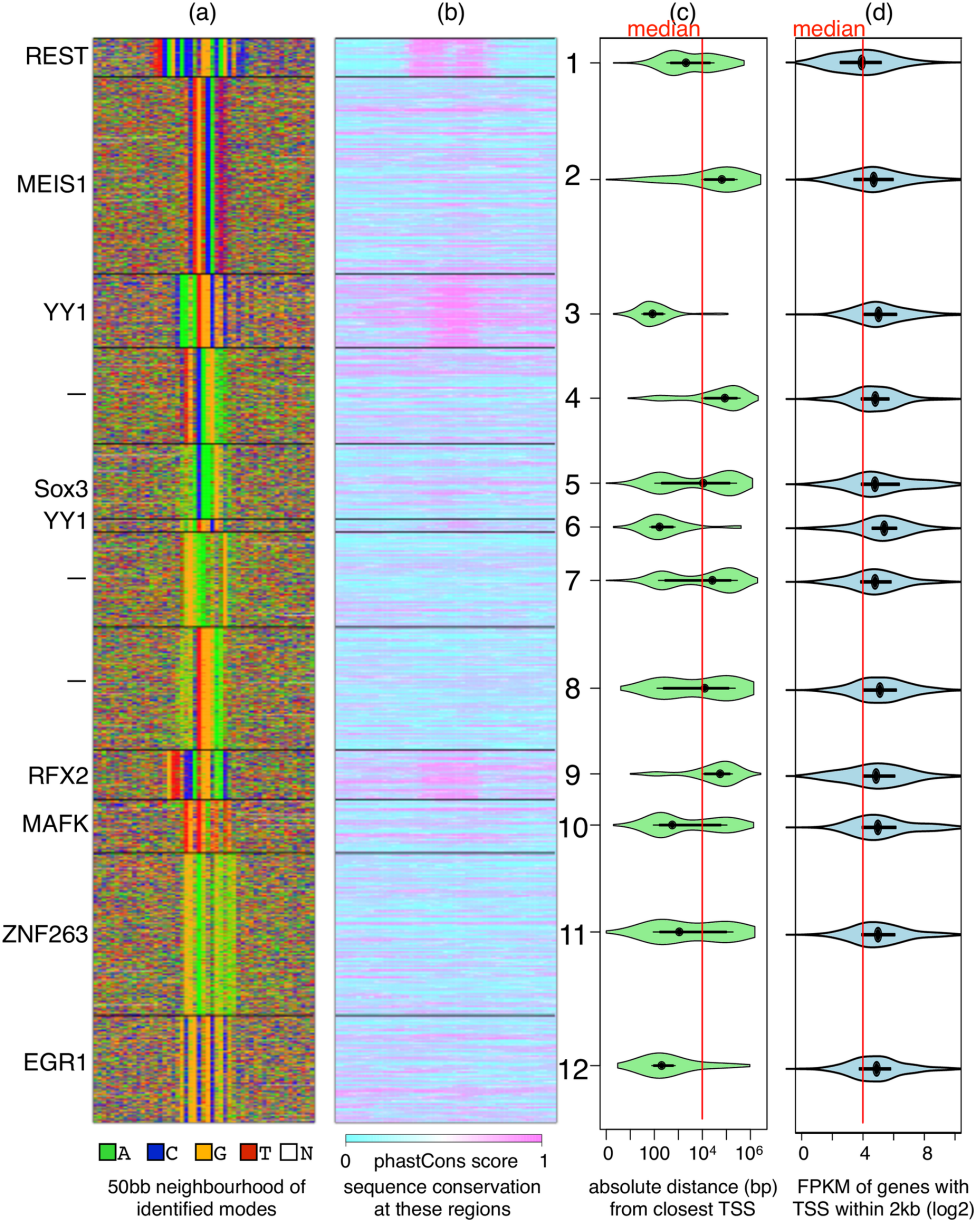
DIVERSITY finds 12 different modes for REST in neurons. (a) The 12 modes are sorted on the basis on average ChIP score. For simplicity, only the top TF predicted to bind each mode by TOMTOM (but with *p* < 10^−4^) is listed on the left. But note that in some cases a whole family of TFs have binding sites that match a mode, e.g., Sox2, Sox3, and Sox6 all have similar motifs—either one of them could be the factor in question. (b) Sequences corresponding to RE-1 (mode 1), YY1 (modes 3&6), and RFX2 (mode 9) are more conserved. (c) While many modes are close to transcription start sites, modes 2, 4, and 9, are more variable in terms of their relative position. (d) All genes except those close to the RE-1 mode are significantly more expressed (*p* < 10^−5^) than average.

**Fig 6:**
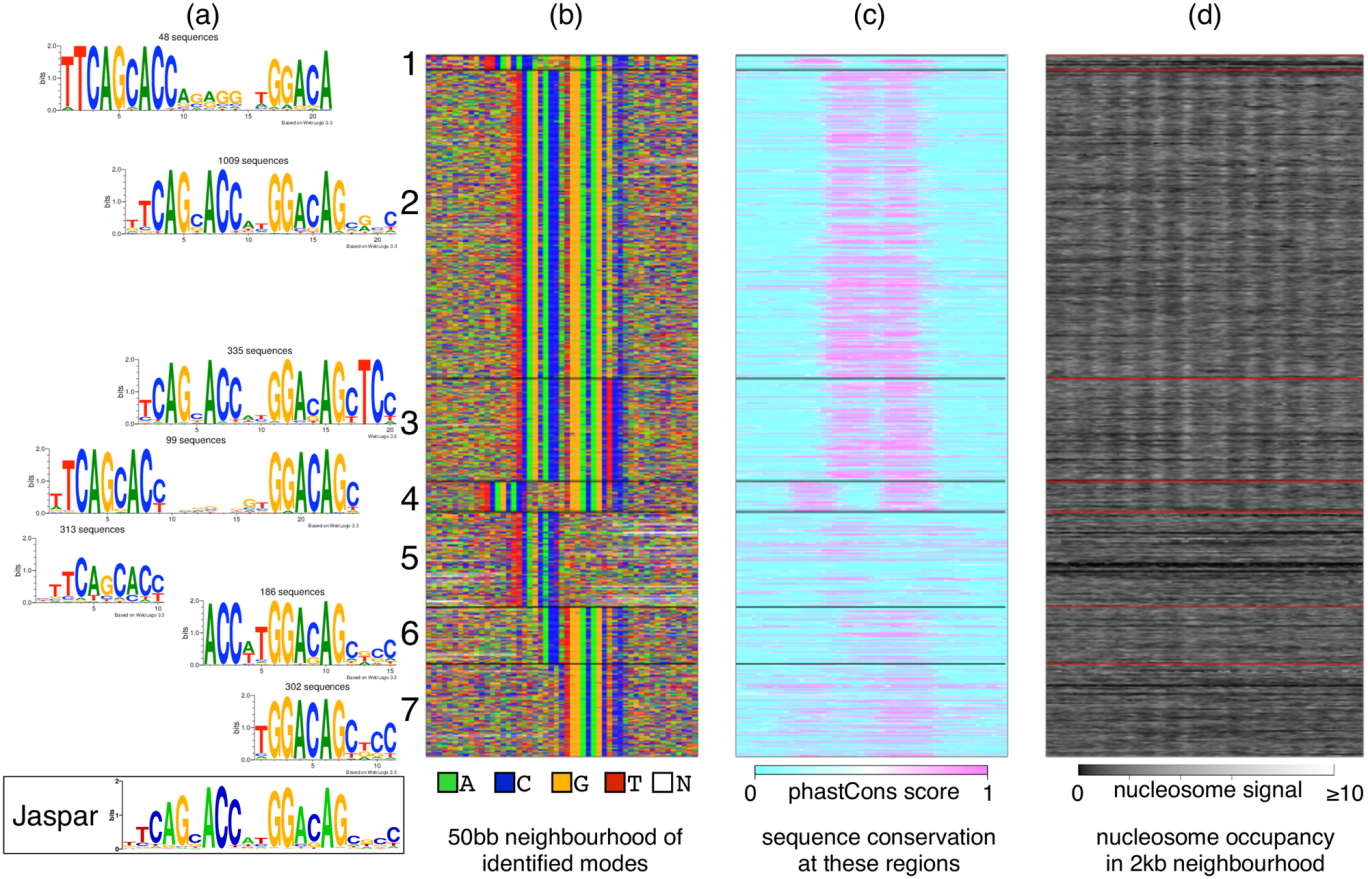
DIVERSITY finds full and half-sites of RE-1 in GM12878. (a,b) Diversity identifies seven modes, all variants of RE-1 (modes sorted based on average ChIP score). Database motif shown below. (c) The full sites are more conserved (d) The full sites have well-organized chromatin structure.

The RE-1 silencing transcription factor (REST) has been shown to repress neuronal genes in non-neuronal cell-types and play regulatory roles in differentiation and development of neuronal cells [33]. It binds directly to DNA, but it also interacts with a diverse set of co-factors and the recruitment of specific complexes is believed to result in distinct transcription outcomes [34]. We therefore consider it a fitting TF for testing diversity. Rockowitz et al. [15] have compiled and analysed REST binding in 15 non-neuronal human cell-types and differentiated human neurons. In the interest of space, here we discuss detailed results only on the neurons (Fig 5) and one non-neuronal cell-type: the lymphoblastoid cell line GM12878 (Fig 6; results on the other datasets are in S4 Fig). GM12878 was chosen due to availability of nucleosome occupancy data in this cell-type.

Rockowitz et al. applied the MAST tool of the MEME suite [35], which scans sequences reported in neuronal cells on the basis of a user-supplied PWM corresponding to the 21bp RE-1 motif (Fig 6 box). They showed there was only a marginal enrichment of RE-1, even in the top 600 sequences. Therefore, it is not surprising that diversity also finds only a small fraction of sequences (≈3.5%) contributing to a mode that looks like RE-1 (mode 1, Fig 5a).

In addition to the RE-1 motif, diversity finds 11 other modes. For all these modes, genes with transcription start sites within 2kb are significantly highly expressed (Fig 5d). This supports behavior of REST as an activator in neurons [36], but suggests this largely happens not by binding DNA directly.

The top TF match from established vertebrate databases is listed on the right: these are most likely co-factors of REST. Although the modes are ranked in the order of ChIP score, there is no significant difference in the scores across the first 10 modes (S4 Fig). This suggests that the binding of REST with DNA is as persistent as the binding of REST with the identified co-factors and of the co-factors with their own DNA recognition sites put together. This is of course, based on the fairly reasonable assumption that REST cannot directly bind non-RE-1 motifs.

We now look at the co-factors in detail. MEIS1 is a homeobox encoding TF, known to be crucial for neuronal differentiation [37]. REST has been shown to repress MEIS1 expression in non-neuronal cells via recruitment of Polycomb Repressor Complexes [38]. But how it interacts with MEIS1 in neuronal cells, where MEIS1 is expressed, is not yet known.

Diversity finds two variants of YY1 motif (modes 3 and 6). YY1 plays a key role in neuronal development and has been shown to positively regulate REST itself [39]. It is very likely that YY1 forms a complex with REST: both are zinc fingers of the same family and YY1 has been shown to bind with other zinc fingers [40]. Both modes are highly conserved and are significantly closer (*p* < 10^−5^) to the TSS.

Mode 9, which matches the winged helix RFX family, is the only other significantly conserved mode. All proteins in this family recognize a near-identical palindromic motif [17]. While the functions of RFX proteins are yet to be understood, at least one member RFX1, has been shown to be critical for the development of the central nervous system in mouse [41]. Diversity results suggest it also interacts with REST.

Mode 5 is a near perfect match to the SoxB1 proteins: Sox2 and Sox3, which have been shown be critical for neuronal differentiation [42]. Mode 10 matches MAFK, a basic leucine zipper TF, involved in several functions including HDAC recruitment [43], something REST is believed to do as well [15].

Modes differ in terms of distance from the closest genes and expression of the downstream gene. MEIS1, RFX2, and mode 4 are far more variable suggesting they might be distal regulatory regions such as enhancers or silencers. Without additional information such as ChIP of these proteins in the same set or chromatin structure information, we cannot say much more about what complexes must be forming along the genome. However these results, from a single ChIP experiment, do point towards a rather complex TF-TF interaction landscape for REST in neuronal cells.

### REST largely binds to RE-1 and variants in non-neuronal cells

In non-neuronal cell-types the picture is dramatically different from neuronal cells. In GM12878 cell line (Fig 6), diversity finds only variants of the canonical 21bp RE-1 motif, which contains two informative pieces separated by a small gap. These asymmetric half-sites of RE-1 have been shown to occur individually in REST-bound regions of non-neuronal cells [44]: these are detected in modes 5 and 7. We find an additional mode 6 that contains the right half and a piece of the left half. All these modes: 5,6,7 are consistently found in the other 14 non-neuronal cell-types as well (S4 Fig), but have a significantly lower ChIP score than the full site (*p* < 10^−5^).

The full sites are more conserved than half-sites. Chromatin structure around full sites is strikingly different: nucleosome are strongly phased and evenly spaced around the full sites similar to what is known to happen with the insulator binding protein CTCF in mammals [45]. A weak nucleosome signal has been described in an earlier study, around computationally predicted RE-1 (not de novo) sites within REST binding peaks [46].

### Number of modes is a characteristic of TF

Here we apply diversity to a collection of diverse TFs in the K562 cell-line (Table 1; S5 Fig for detailed output) to assess the generality of the method. There is a clear variation in the number of modes detected across the TFs. As expected, the P300 protein, a general activator that does not bind DNA directly, has the most number of contact-types, which suports our current understanding about its function: it binds to several different DNA-binding TFs [50] and is a marker for enhancers [51]. Indeed, in this dataset, diversity detects motifs resembling RUNX1, GATA, AP-1, SP1, and CTCF which are all active in this cell-type. In contrast, in the ChIP-seq of cell-type specific TFs FOXA1, GATA1, GATA2, and USF1, the number of modes is only two, one of which resembles the literature consensus of the respective TF. In the case of FOXA1, the second mode is the GATAA motif, while for the other three TFs, the second mode is a C-rich motif. This motif also occurs in some of the other TF sets, but in all cases the ChIP score at the sequences contributing to it is low. This may be a case of non-specific binding (Discussion). RUNX1, also a cell-type specific protein, has a mode that resembles the RUNX1 motif, one that is a variant and a third that matches SP1, a known RUNX1 co-factor in leukemia [49]. In all these cases, the sequences contributing to the known cognate motif of the profiled TFs have a significantly higher ChIP score than the other modes (S5 Fig), suggesting direct binding at those places.

**Table 1:** Output of diversity on ChIP-seq data from 10 TFs in K562.

TF	Known activity of TF [47]	Number of contacts discovered
FOXA1	A forkhead protein, binds DNA and interacts with chromatin	**2 modes**: Diversity identifies the FOXA1 motif and the GATA motif. FOXA1 is believed to stabilize GATA complexes by changing the local chromatin landscape [48].
GATA1 GATA2	Members of the GATA family of zinc finger TFs	**2 modes in each set**: One resembles GATAA; other is a C-rich motif
USF1	A member of the basic helix-loop-helix leucine zipper family, recognizes the E-box motif	**2 modes**: The larger mode matches the E-box, while the other is a new motif.
RUNX1	A heterodimeric TF that binds to a core element of many enhancers and promoters.	**3 modes**: Two are variants of the RUNX1 motif, one resembles SP-1, which is a known co-factor of RUNX1 [49].
JUNB	Is part of the AP-1 complex	**4 modes**: Over half of the sequences are accounted by AP-1 resembling mode, others are novel.
FOSL1	Dimerizes with other leucine zipper proteins, is part of complex AP-1, activator	**6 modes**: In addition to variants of the TGAsTCA AP-1 motifs, diversity discovers GATAA motif and two unknown motifs
IRF2	An interferon regulatory factor, known to have both activating and repressing functions	**10 modes**: The multiple modes include promoter motifs, CTCF, and variants of IRF known motif [17]
THAP1	Contains a THAP domain, colocalizes with the apoptosis response protein PAWR/PAR-4 in leukemia.	**11 modes**: Modes include motifs resembling SP1, AP-1, YY1, E-box motif, THAP11, and many promoter elements.
P300	A histone acetyltransferase that regulates transcription via chromatin remodeling	**17 modes**: CTCF, RUNX1, GATA, AP-1, SP1 are among motifs that feature in these modes.

FOSL1 from the Fos family and JUNB of the Jun family are part of the AP-1 complex, which is involved in multiple cellular processes. AP-1 complexes are known to be instrumental in looping DNA and involved in enhancer-promoter interactions [52], which explains the multiple modes in these TFs. But interestingly, there is no unique AP-1 complex: it can contain diverse combinations of TFs from both the Fos and Jun families [53]. This explains why other than the characterized AP-1 motif of TGAsTCA, there are no common modes between the two TF datasets. In fact, in the case of FOSL1, the mode with the highest ChIP score is a strong motif but not recorded in the standard databases.

IRF2 is one of the interferon regulatory factors (IRFs), which bind to AANNGAAA. Variants of this motif are discovered as distinct modes by diversity. IRFs have different C-terminal regions which help facilitate specific protein-protein interactions [54]. This may explain the additional modes found in this set.

THAP1 is a zinc finger protein that is known to interact majorly with a general transcriptional regulator HCFC1. HCFC1 does not bind DNA directly but via interactions with other TFs such as YY1, E2F1, and THAP11 [55], all of which diversity detects as separate modes.

### Comparison with other motif discovery methods

The goal in traditional motif discovery is to find a statistically overrepresented motif, typically one that appears in a large fraction of the data [56]. To identify more than one motif, the same approach is applied iteratively: occurrences of motifs identified in the previous passes are masked before searching for the next overrepresented motif. This is conceptually different from diversity, whose goal is to identify a set of motifs, which together explain the entire dataset. Here we compare and contrast diversity’s output with that from two standard approaches: MEME, which is targeted for wide motifs corresponding to complexes, and DREME, which is targeted for finding shorter monomeric motifs likely to be cofactors [35]. We discuss results on the 16 REST sets, where the direct binding motif is well-characterized. MEME detects the full RE-1 motif in only 11 of the 16 sets (in spite of relaxing the definition of a “full RE-1 site” to at least 14bp containing cores of both half-sites). In others, MEME finds the two half-sites or variants as separate motifs (S6A Fig). This is because in these five sets MEME identifies the two half-sites first during its sequential motif discovery, and then masks them to find the next most enriched motifs, therefore missing the full site. In contrast, if the full site is *more* overrepresented than the individual variants, it gets detected first and the half-sites subsequently get detected if they are individually overrepresented in the full dataset. We see a similar picture with DREME: it never identifies the full site, possibly because of its bias to short motifs.

The number of motifs returned by each of the three programs is different, as well. In GM12878, for example, although neither MEME nor DREME finds the full RE-1 motif, DREME finds eight non-RE-1 motifs and MEME finds 17, some of which are supported by only four sites. Indeed, multiple user-defined/default parameter values such as the minimum number of sites for a motif, the E-value cut-off for enrichment, etc. decide whether a motif will be reported in these methods. Diversity, in its Bayesian formulation, uses one primary hyperparameter λ to determine how much to penalize models with more modes. Fig 6 suggests that variants of the RE-1 motif are probably enough to explain the REST bound regions in GM12878: they cover the entirety of the set. We of course cannot rule out the biological role, if any, of the motifs reported by MEME/DREME.

We are aware of one method—InMoDe—published recently [22], that considers the data to be a mixture of “motif-types”. Developed with the motivation of identifying dependencies within binding sites, InMoDe relaxes the inherent assumption of independence in PWMs by learning inhomogeneous parsimonious Markov models instead. But it needs both, the width of the motifs and the number of modes to be specified by the user. We therefore ran InMoDe on the REST datasets with the same number of modes that diversity finds as optimal. We set the width to 20 (see “Other programs” in Design and implementation), to ensure that the full RE-1 binding site has a chance of getting discovered (S8 Fig). In 15 of the 16 REST datasets the full motif is one of the detected modes, but only in five are both half-sites detected as separate modes, which are understood to be prevalent across cell-types [57] and are detected by diversity. Instead, InMoDe finds several modes with low information content, which may have biological significance, but at this point we cannot explain. We stress that this is not a fair comparison, since the motivation behind InMoDe and therefore its objective function is very different from diversity’s, implying that the number of optimal modes as determined by diversity may not be optimal for InMoDe. But there is no mechanism currently, in InMoDe, to identify the optimal width or number of modes. That said, InMoDe is significantly faster: it takes only 20 minutes on the neuronal REST dataset to find 12 modes on a single processor, compared with 85 minutes taken by diversity in parallel mode. InMoDe uses stochastic EM, a promising direction to explore for diversity.

## Discussion

A ChIP experiment is like a black-box: it reports all regions that are cross-linked and associated with the profiled protein, often constituting a highly diverse set of DNA sequences. Diversity identifies the different components of this mixture, leaving no data behind, and at the same time, using no prior motif/TF knowledge. The proportion of sequences in each component can be highly variable: an example is the discovery of the tiny set of sequences containing RE-1 in the neuronal REST set (Fig 5).

With the algorithmic advances presented here, diversity is now comparable in speed with standard motif discovery methods. The actual time for convergence depends on the structure of the search space: a dataset with a few clear modes will result in faster convergence. For neuronal REST, which is one of the most diverse of our sets, diversity takes an hour and 25 minutes to learn a model with 12 modes. In contrast, for the similarly sized K562 REST, diversity takes less than an hour. Since diversity has to learn all models with number of modes in the range given by the user (1 to 20 in this case), before it can report the optimal model, the total time taken for the neuronal set is about 27 hours. On the same machine, MEME, also running in parallel mode, takes over 40 hours to find 20 motifs (S1 Fig).

Our results support the fact that diversity in regulation is driven in large part by diversity in sequence: the chromatin structure correlates with the different modes and so does sequence conservation. Indeed, diversity opens up avenues for understanding the functional role of each reported ChIP region by examining the characteristics of the detected modes. Certain modes may play a role in chromatin organization, some in activation, some in repression, and so on. This can be learned by combining information from other sources such as histone/DNA modification, sequence conservation, gene ontology (GO) of downstream genes, etc. In particular, we showed that diversity can give new insights into protein-DNA interactions even in widely studied TFs like the fly CTCF: it appears to bind human-like CTCF sites with lower efficiency; it interacts with specific promoter architectures; and that CTCF-Pita is likely a *melanogaster* subgroup-specific interaction, at least in the embryonic stage. Furthermore, our results suggest that one Su(Hw) molecule may be interacting with two distant DNA regions through its various zinc fingers. This is not an outlandish claim: Gata3, which has two zinc fingers, has been shown to bind to two GAT half sites separated by a long linker region [58]. Admittedly, such experiments for Su(Hw) are necessary to confirm our hypothesis.

Diversity currently does not model the efficiency of the cross-linking or the immunoprecipitation step. Consider a situation where the profiled protein binds a DNA region through a chain of intermediaries. For the region to be reported, each interaction in the chain must get fixed during cross-linking and the antibody should be capable of recognizing the protein when it is part of this complex. Perhaps incorporating the accompanying ChIP binding score in the model will give further insights into the stability of the complexes.

We also note that a ChIP experiment has its own limitations. Phantom peaks biased towards highly expressed regions have been reported in ChIP-seq experiments [29, 59]. Indeed, several promoter elements are identified as separate modes in many of the datasets studied here. Therefore we cannot discard the possibility that other modes are picked up perhaps because the regions are open and the TF “happens” to co-localise there, without its own cognate motif or is simply a result of an artifact of the ChIP experiment. One needs to be cautious when calling an identified mode the motif of a “co-factor”. Since the only information diversity uses is the DNA sequence at the ChIP regions, it can make no claim of the function of the identified components; that needs to be validated by separate means, with additional experiments/data.

The framework of diversity is conceptually distinct from standard motif discovery tools, since it asks and answers a very different question. Therefore Diversity does not seek to replace these tools, but it can provide insights in cases where diverse configurations of the same TF are to be detected, specifically from ChIP data. We note that other high-throughput experiments that identify regulatory regions such as active enhancers [60], accessible chromatin [3], transcription initiation [61] will also benefit from such analysis, since there is even more likelihood for such data to be a mixture of multiple sequence components. For example, DNase I hypersensitive sites (DHSs) are accessible regions: they may be reported because they are active promoters, or enhancers, or insulators, or even matrix-attachment regions. There must be diverse sequence signatures that will explain the function of the DHSs. However, diversity limits each sequence to have only one binding site: in other words, one mode is defined by only one motif. For a ChIP-seq experiment that measures a specific TF-DNA interaction, this is a reasonable assumption, but to use diversity on the above mentioned high-throughput experiments the definition of a mode needs to be relaxed to a collection of motifs. We hope to incorporate this in the next version of diversity.

## Supporting information

S1 Fig Time taken for DIVERSITY and MEME on the REST datasets. (PDF)

S2 Fig Screen-shots of the webserver (input form and sample output). (PDF)

S3 Fig Output of DIVERSITY on fly datasets. (PDF)

S4 Fig Output of DIVERSITY on the 16 REST datasets. (PDF)

S5 Fig Output of DIVERSITY on 10 different TFs in K562. Logo from JASPAR is shown when available.
(PDF)

S6 Fig Output of MEME on (A) REST ChIP-seq, (B) Fly TFs, and (C) K562 TFs. (PDF)

S7 Fig Output of DREME on (A) REST ChIP-seq, (B) Fly TFs, and (C) K562 TFs. (PDF)

S8 Fig Side-by-side output of InMoDe and DIVERSITY on 16 REST ChIP-seq datasets, when both were given a fixed motif-width of 20 (see “Other programs”). (PDF)

S1 File Archive of the source code of DIVERSITY, documentation, all the datasets used in the study, and instructions for installation/running DIVERSITY. (GZ)
