# Supplementary material for: DIVERSITY in binding, regulation, and evolution revealed from high-throughput ChIP
Source: PLoS Comput Biol. 2018 Apr 23;14(4):e1006090. doi: 10.1371/journal.pcbi.1006090 (PMC5933800; doi:10.1371/journal.pcbi.1006090)
Supplement: S1 Fig — (PDF) [file pcbi.1006090.s001.pdf]

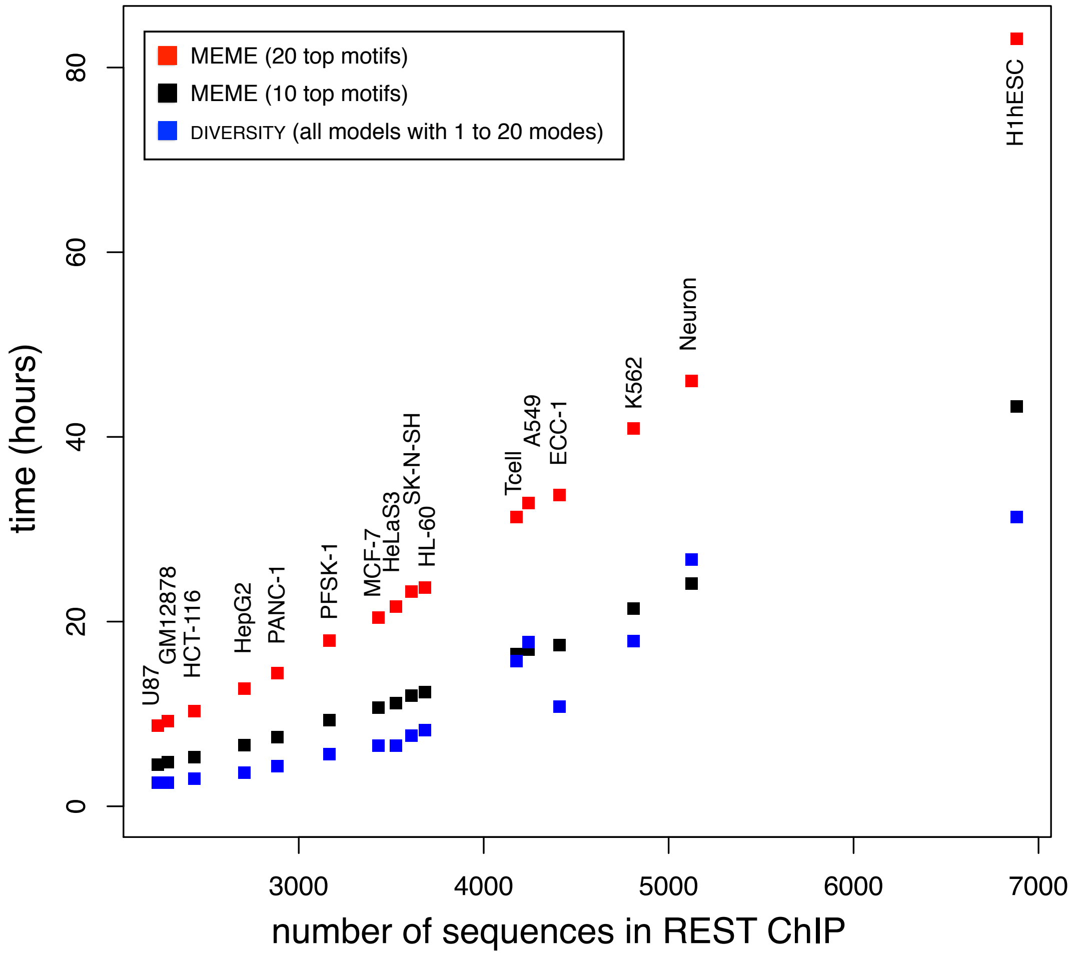

Supplementary figure S1: Run time of DIVERSITY is comparable to that of MEME DIVERSITY and MEME were run on all 16 REST datasets as compiled by Rockowitz et al. (2014). Both were run with the option of using 16 processors, on Intel(R) Xeon(R) CPU E5-2630 v3.
