# Supplementary material for: DIVERSITY in binding, regulation, and evolution revealed from high-throughput ChIP
Source: PLoS Comput Biol. 2018 Apr 23;14(4):e1006090. doi: 10.1371/journal.pcbi.1006090 (PMC5933800; doi:10.1371/journal.pcbi.1006090)
Supplement: S3 Fig — (PDF) [file pcbi.1006090.s003.pdf]

CTCF in *D. melanogaster*
$n = 2180$

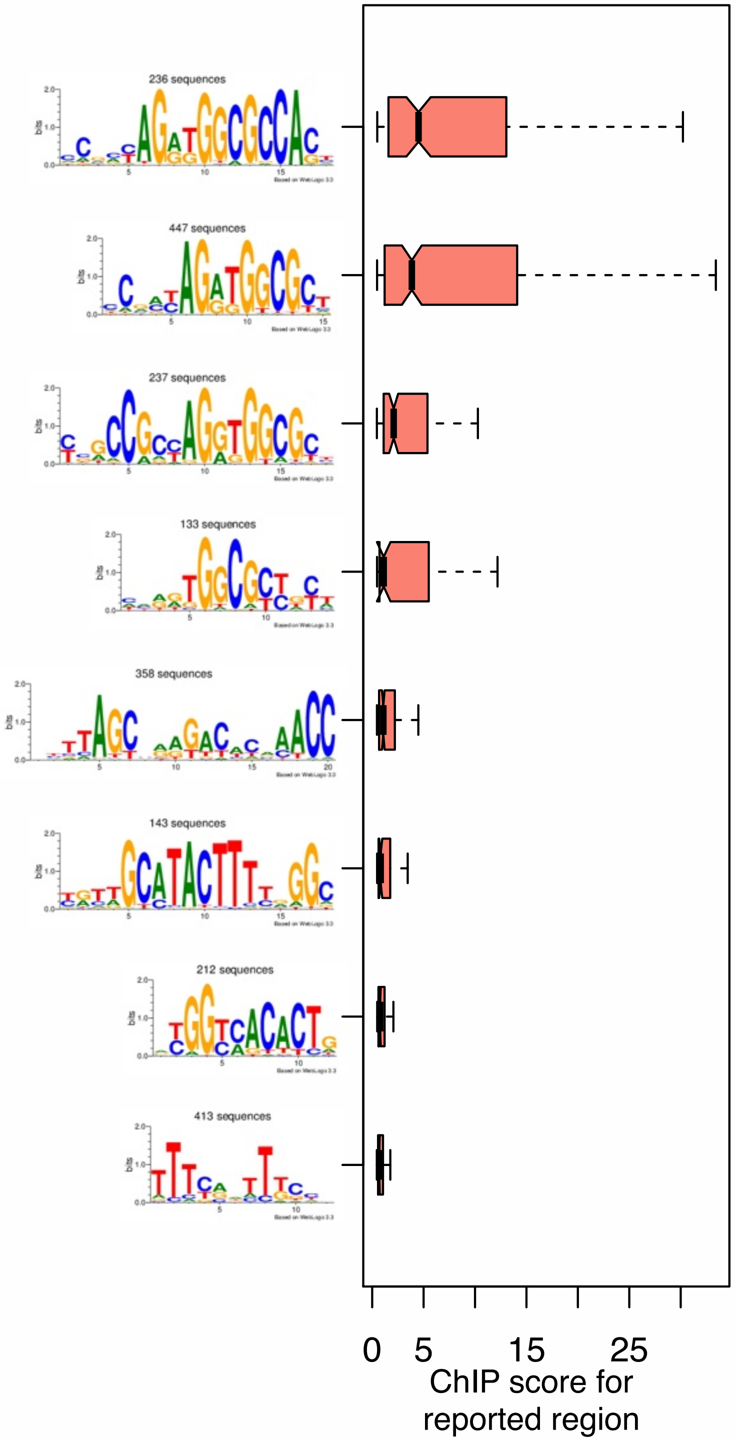

CTCF in *D. simulans*
$n = 2191$

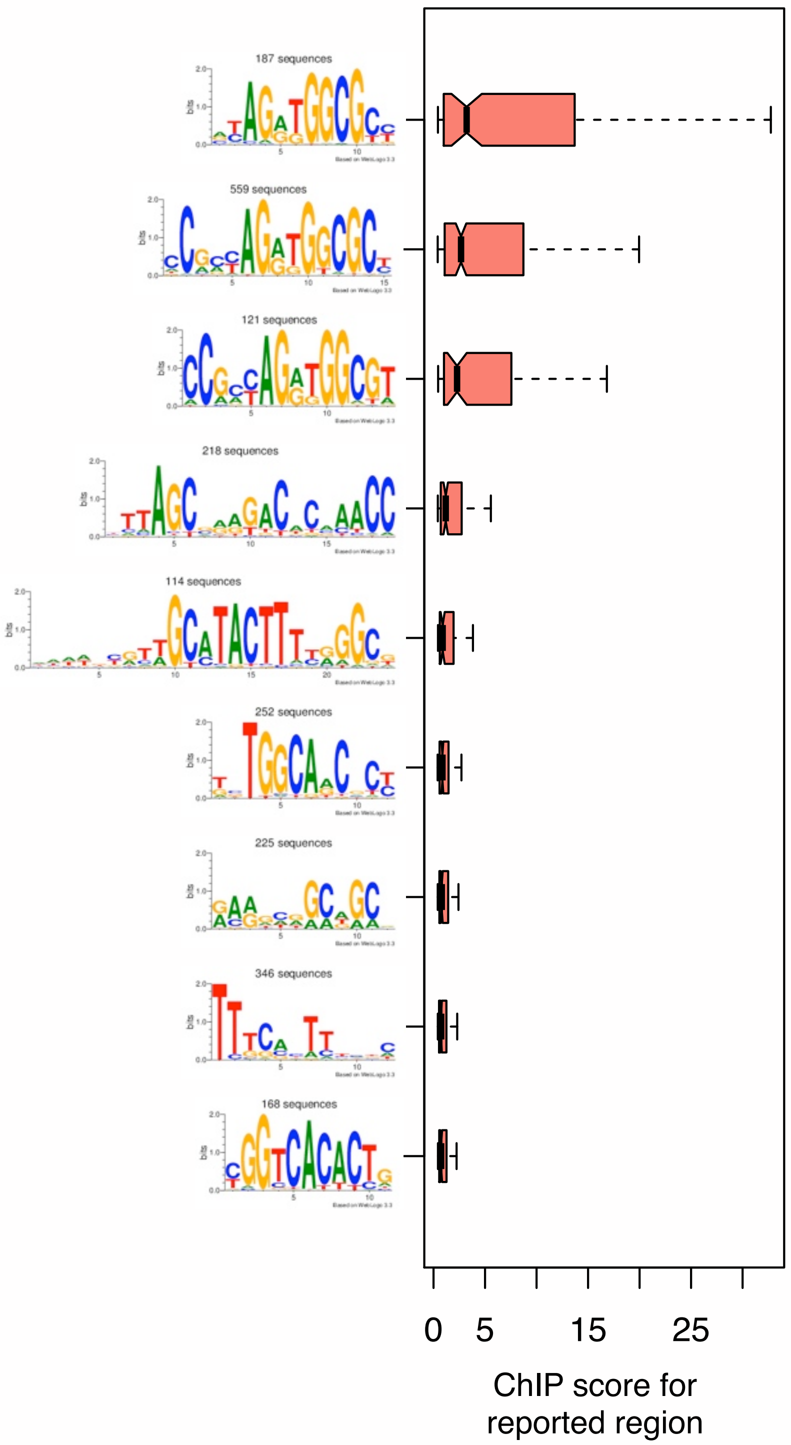

CTCF in *D. yakuba*
$n = 2984$

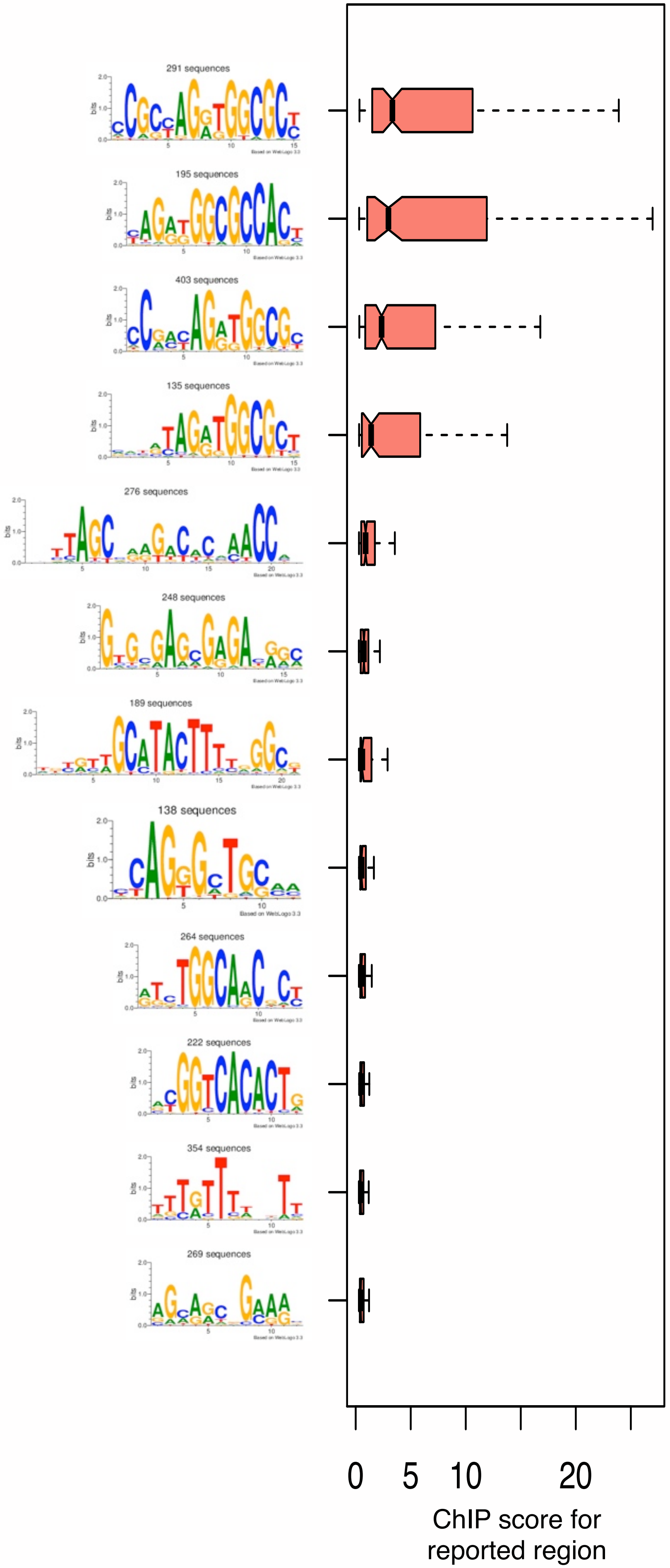

CTCF in *D. pseudoobscura*
n = 2381

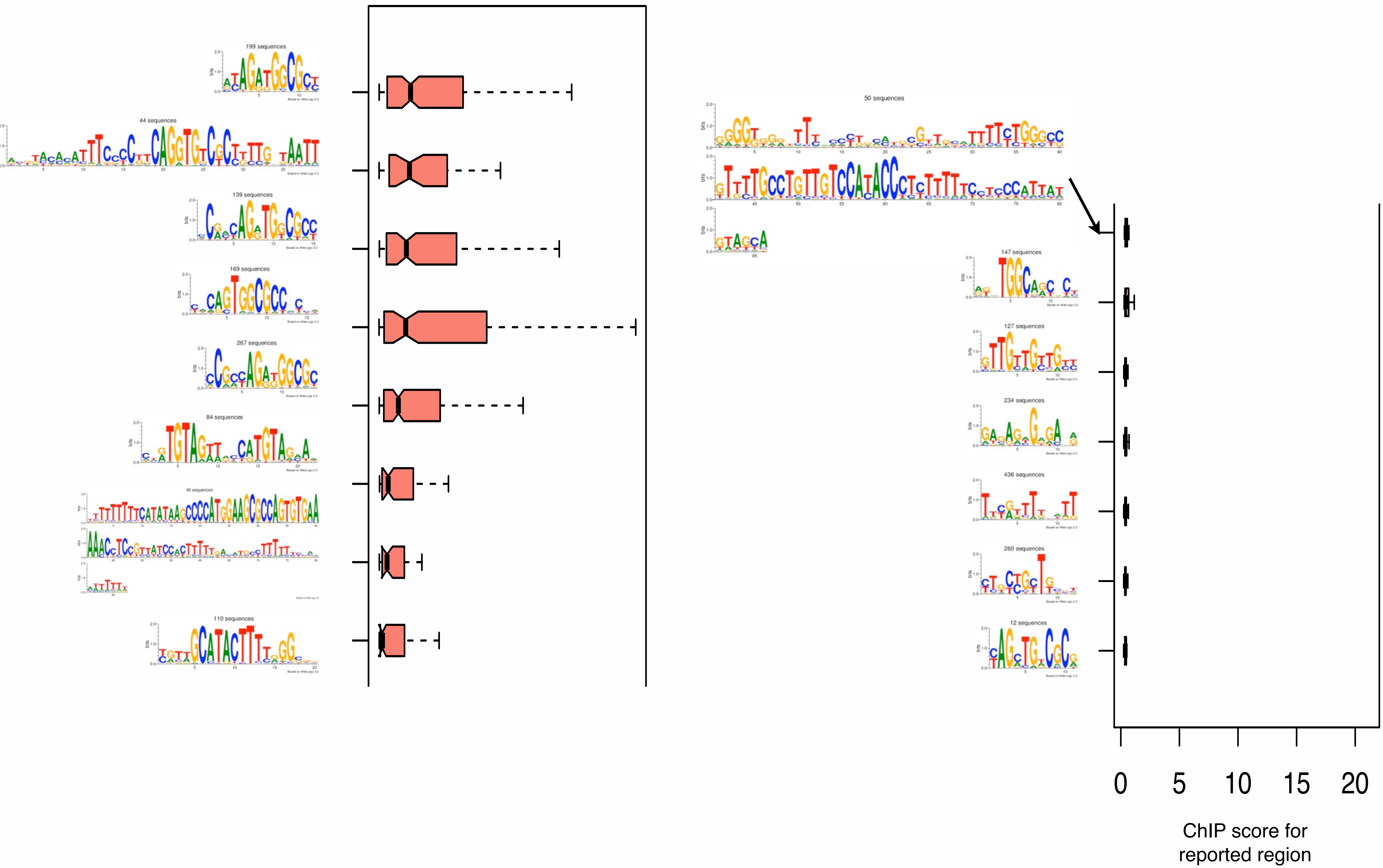

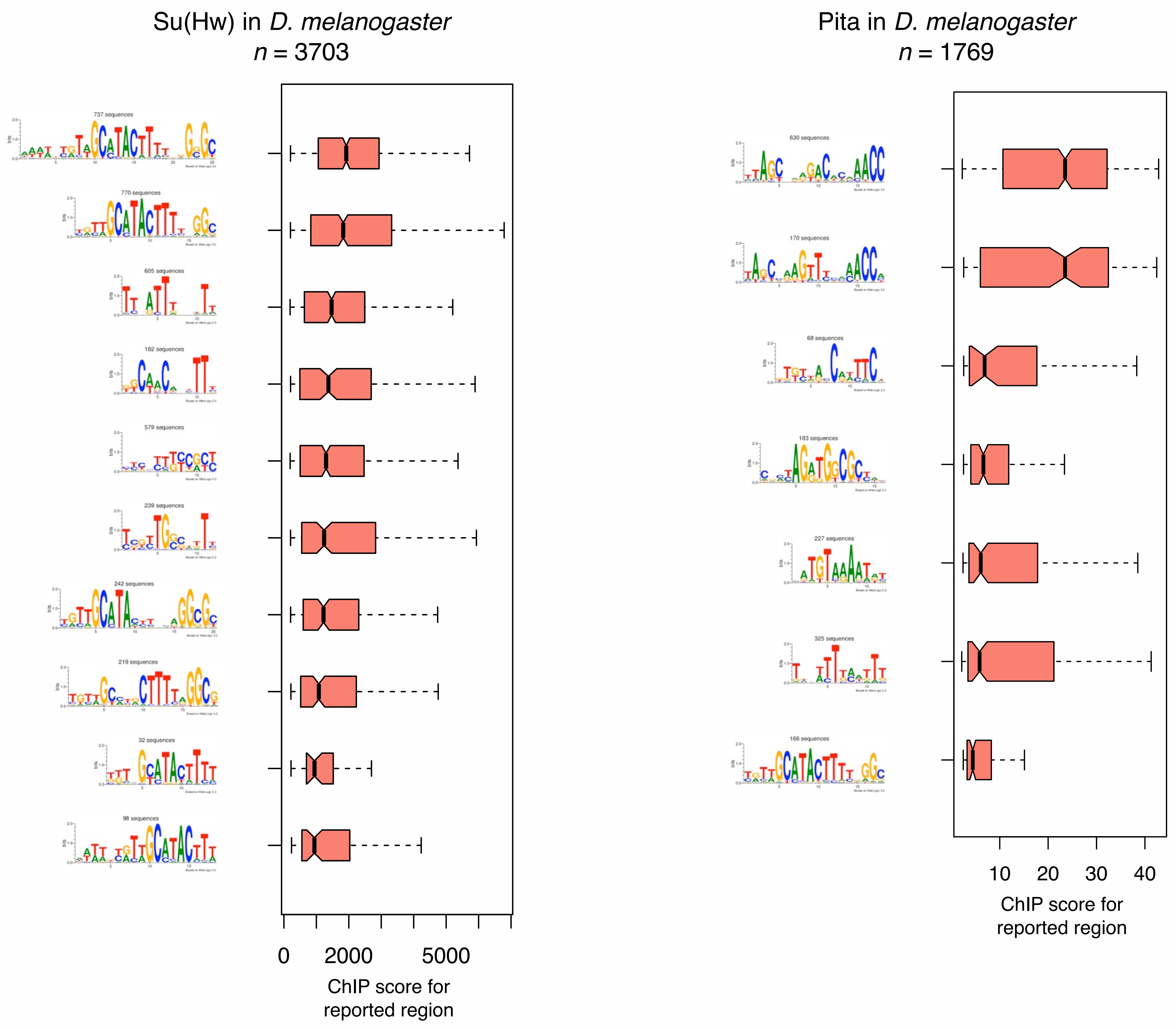
Su(Hw) in *D. melanogaster*
n = 3703
737 sequences
770 sequences
605 sequences
182 sequences
579 sequences
239 sequences
242 sequences
219 sequences
32 sequences
98 sequences
0
2000
5000
ChIP score for reported region
Pita in *D. melanogaster*
n = 1769
630 sequences
170 sequences
68 sequences
183 sequences
227 sequences
325 sequences
166 sequences
10
20
30
40
ChIP score for reported region
