# Supplementary material for: DIVERSITY in binding, regulation, and evolution revealed from high-throughput ChIP
Source: PLoS Comput Biol. 2018 Apr 23;14(4):e1006090. doi: 10.1371/journal.pcbi.1006090 (PMC5933800; doi:10.1371/journal.pcbi.1006090)

A549 (4243 sequences)

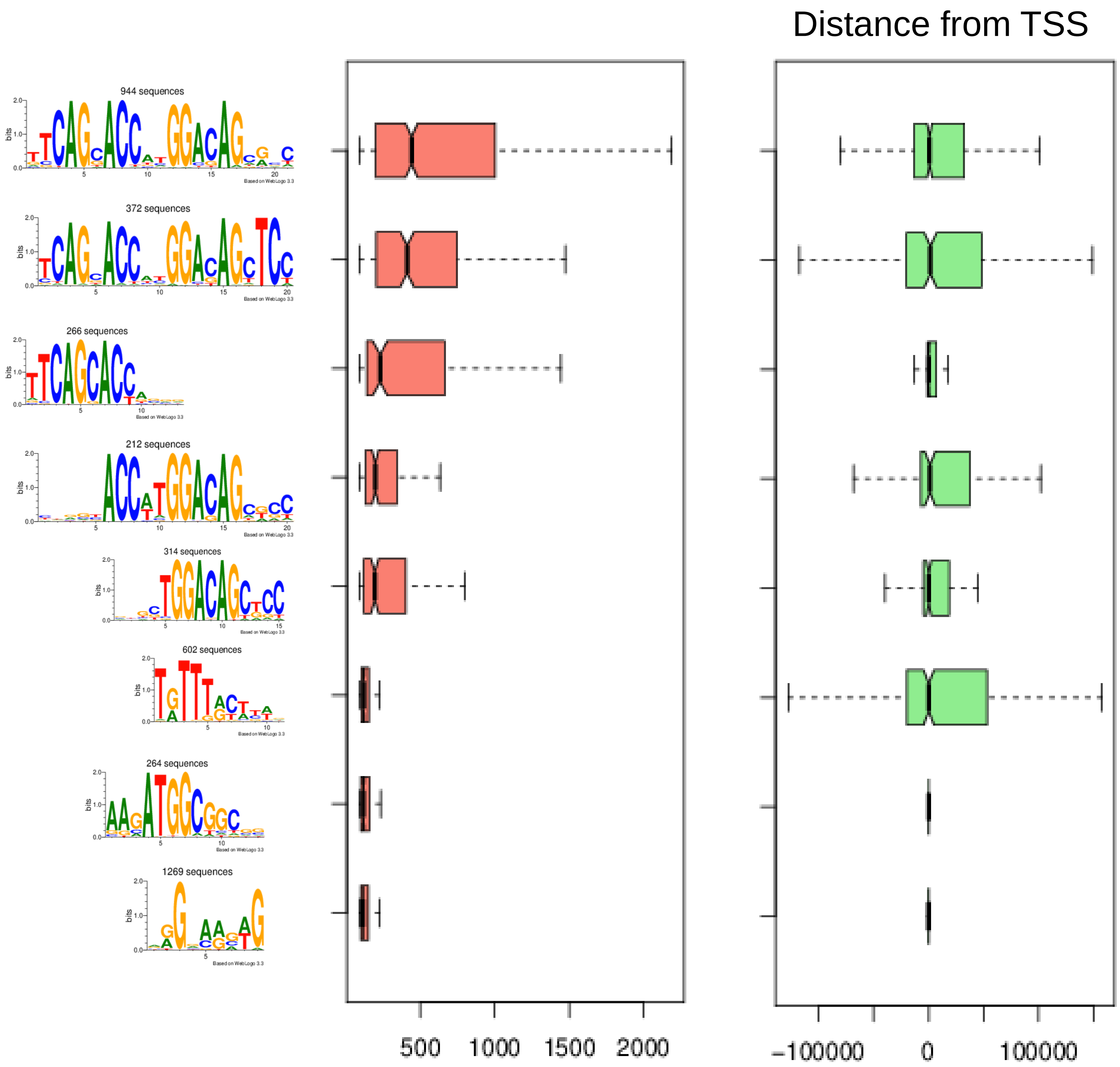

ECC-1 (4410 sequences)

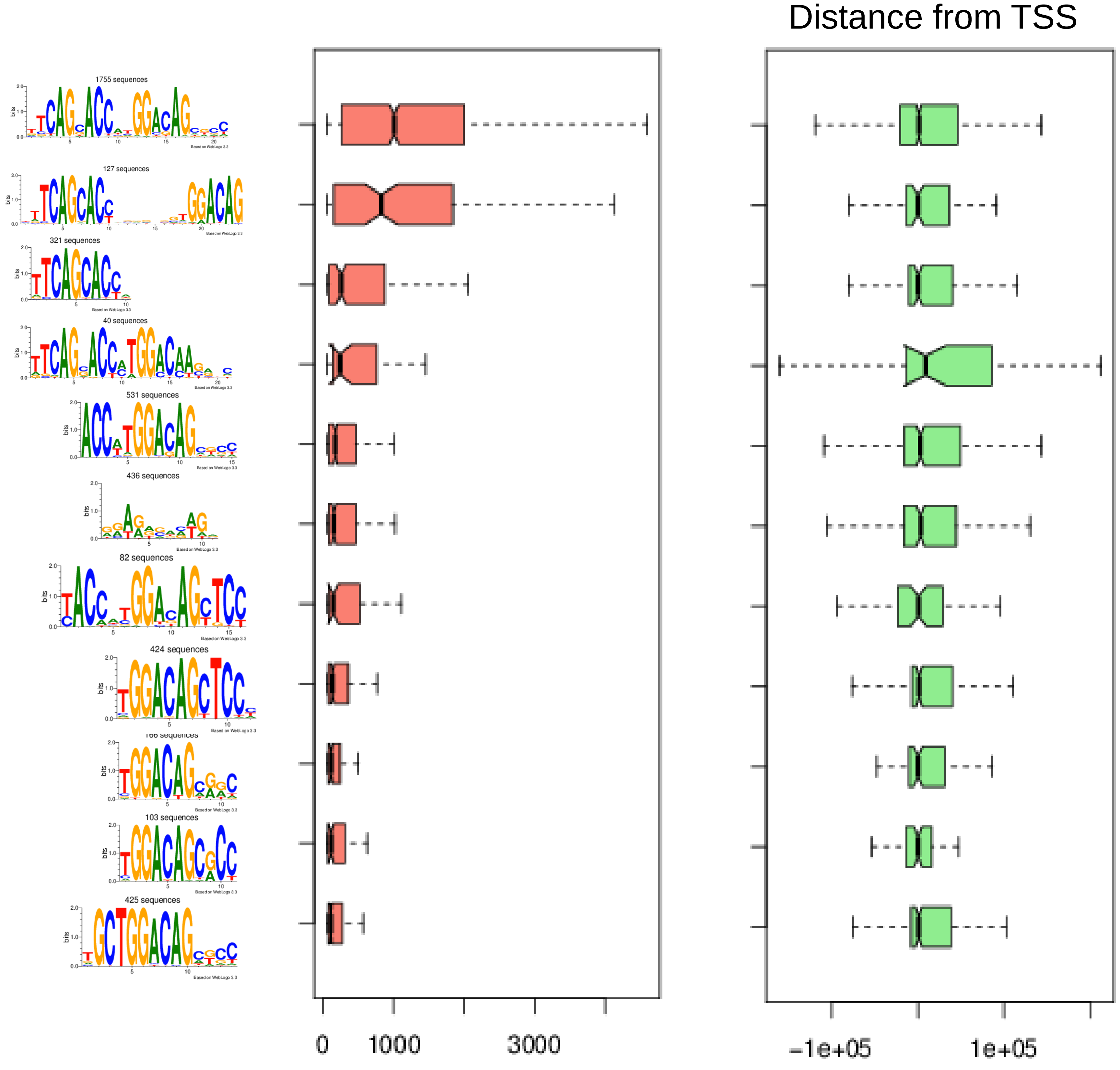

GM12878 (2292 sequences)

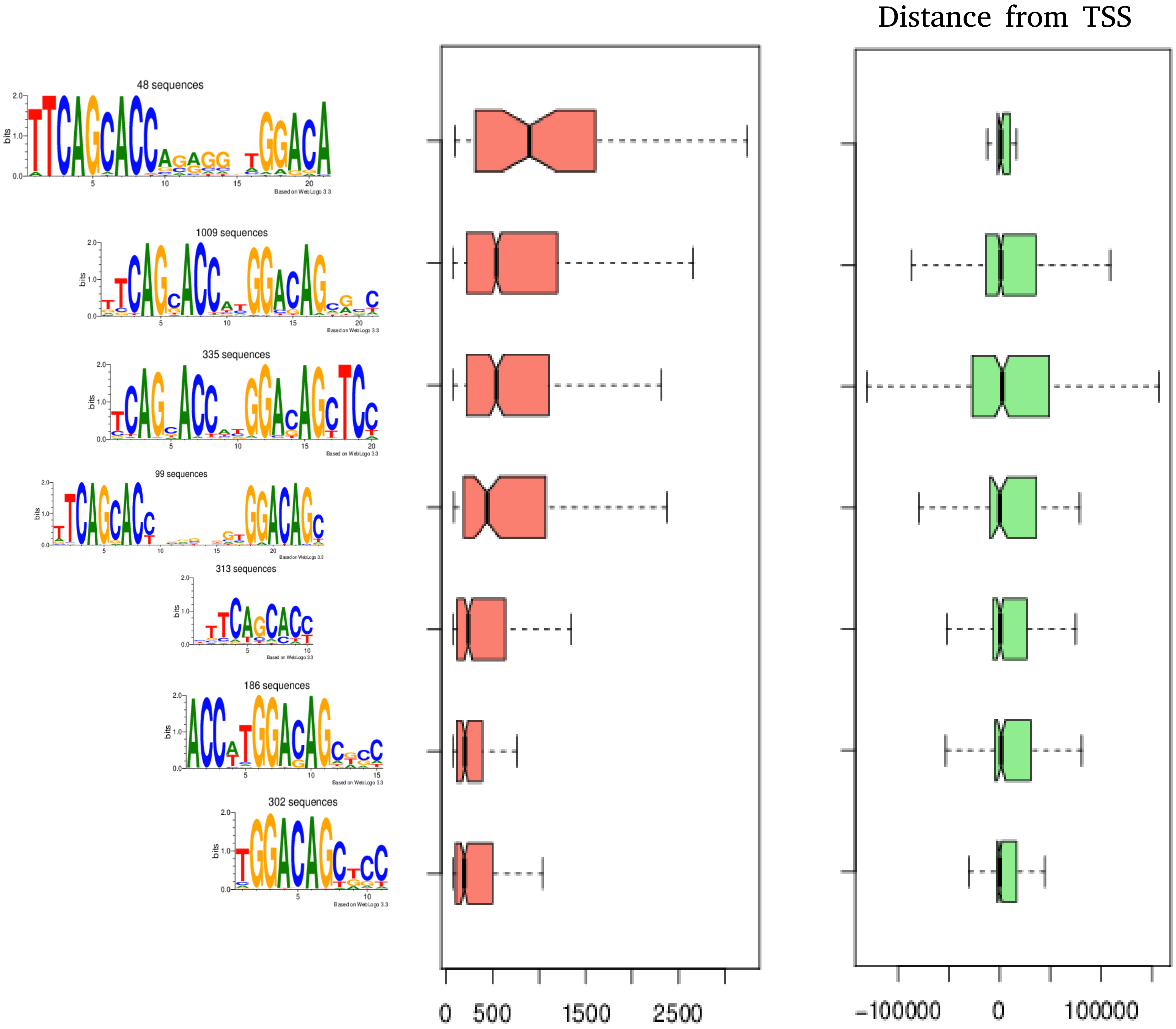

H1-ESC (6882 sequences)

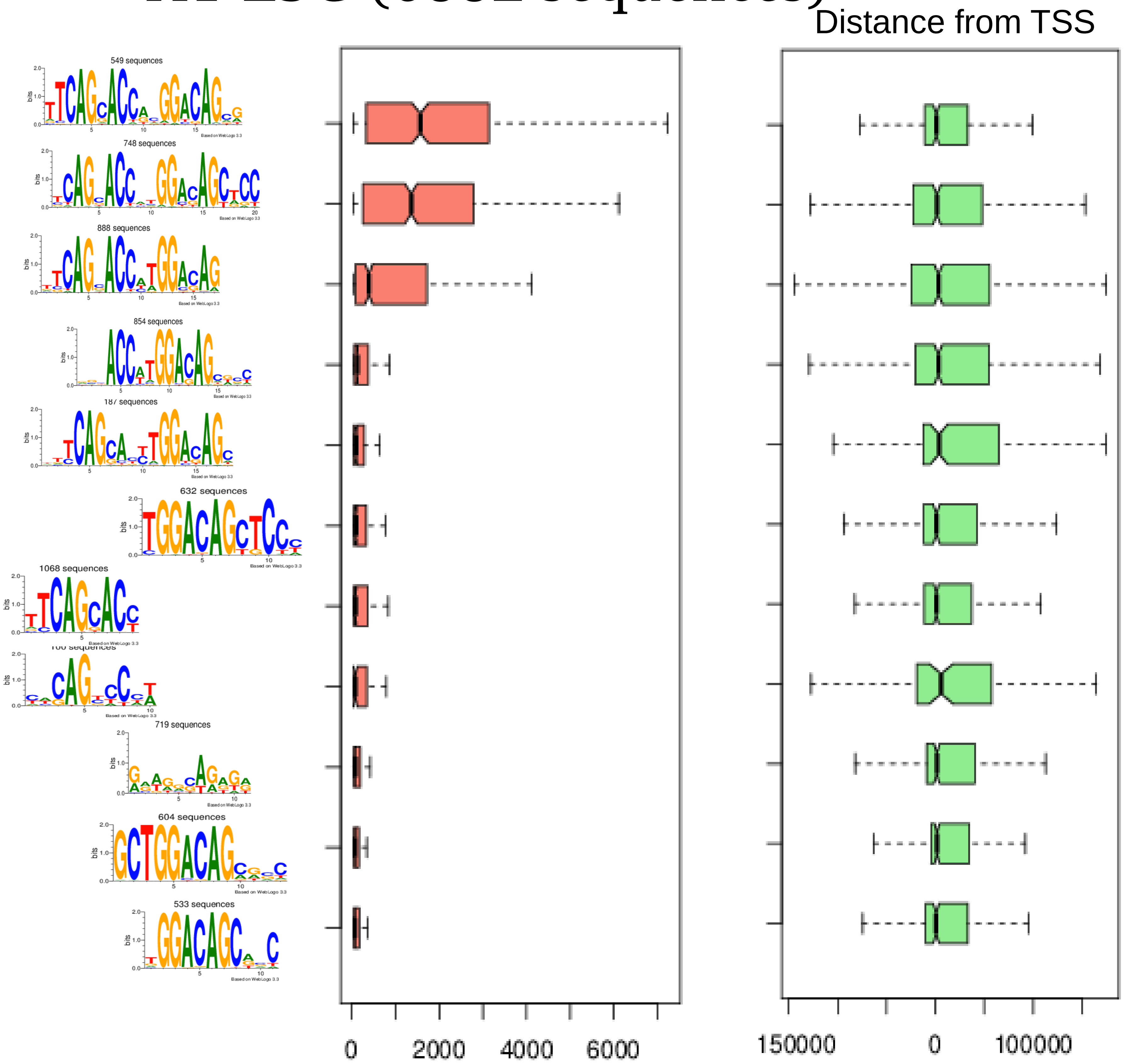

HCT-116 (2436 sequences)

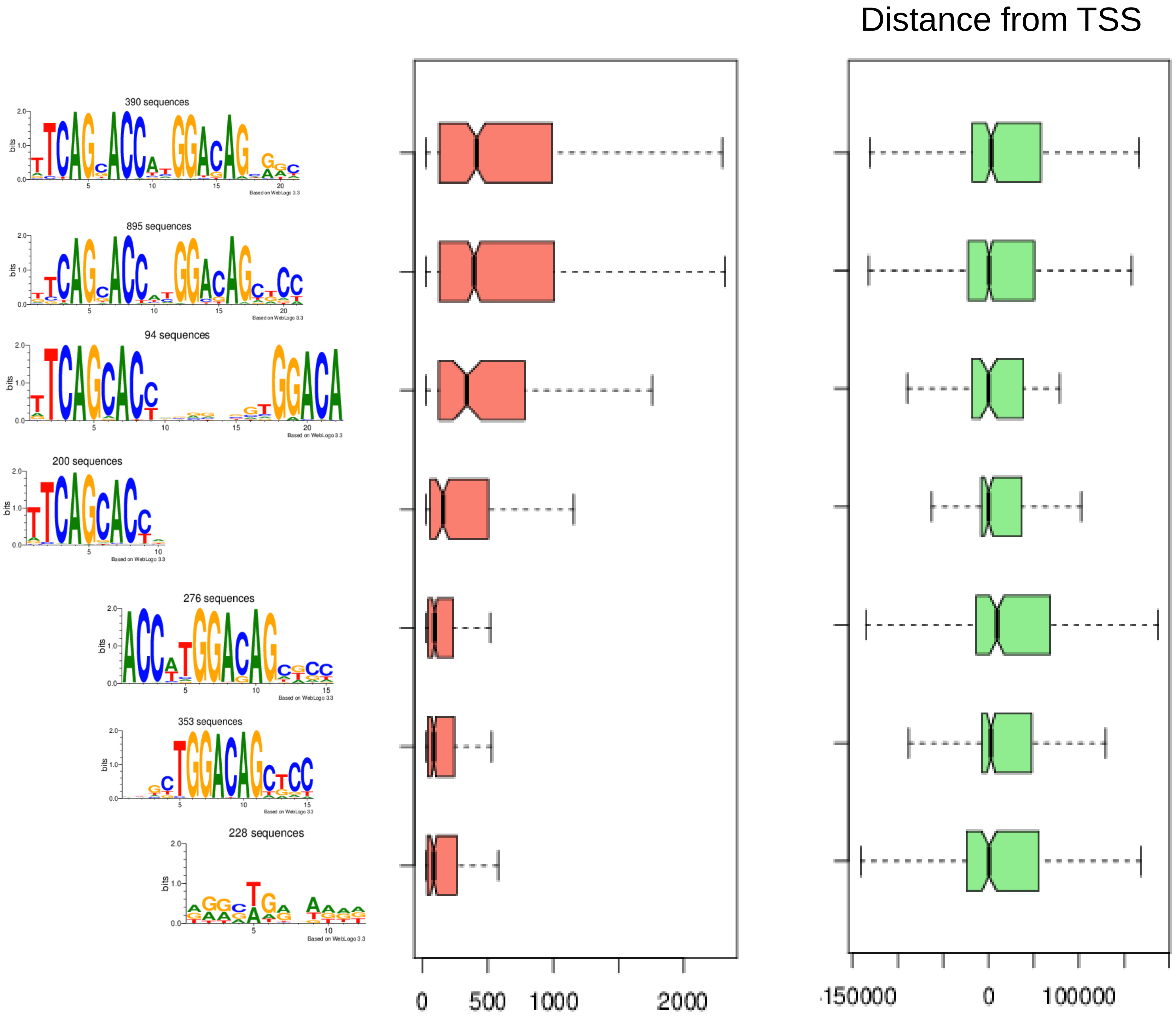

HelaS3 (3526 sequences)

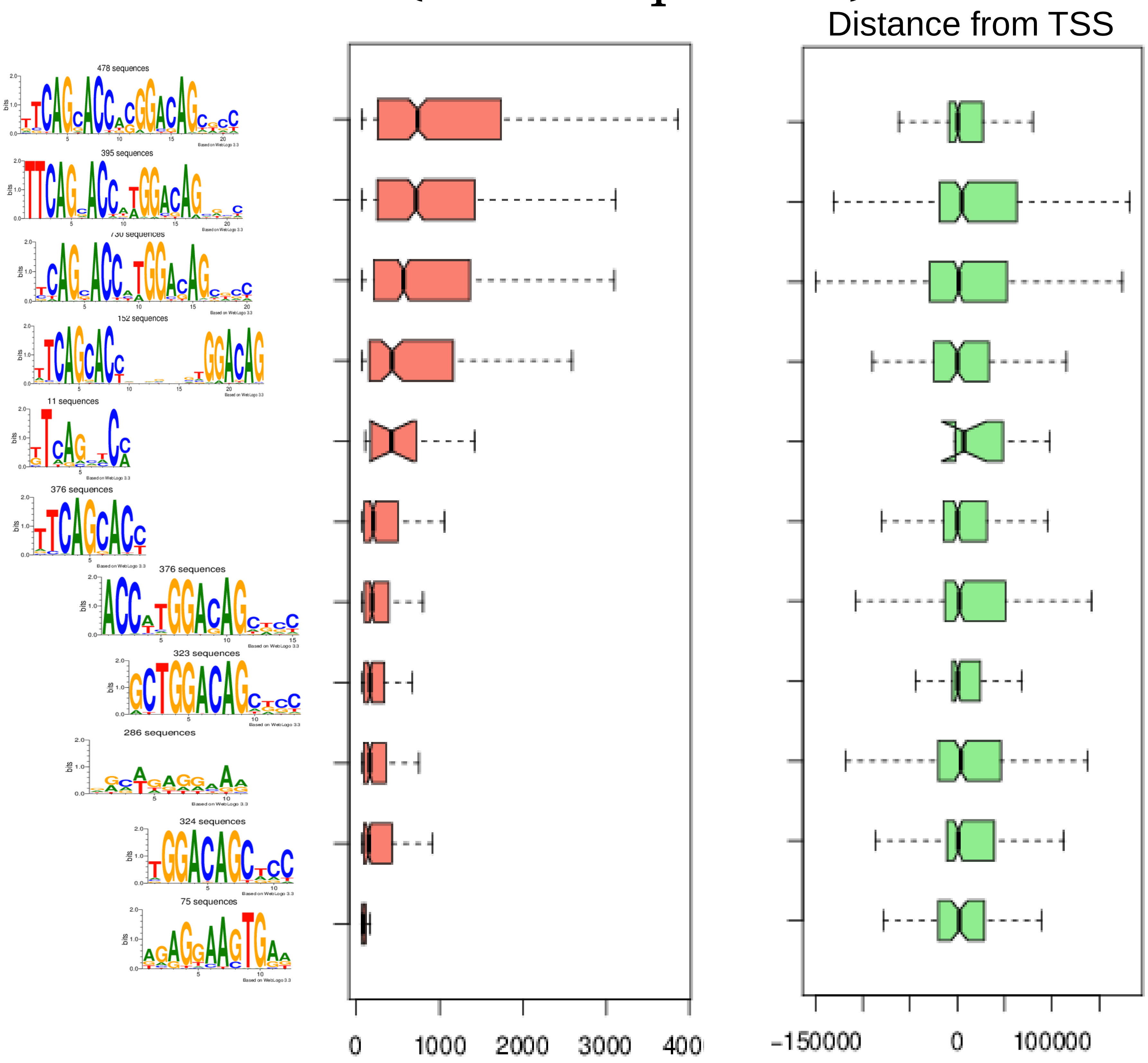

HepG2 (2706 sequences)

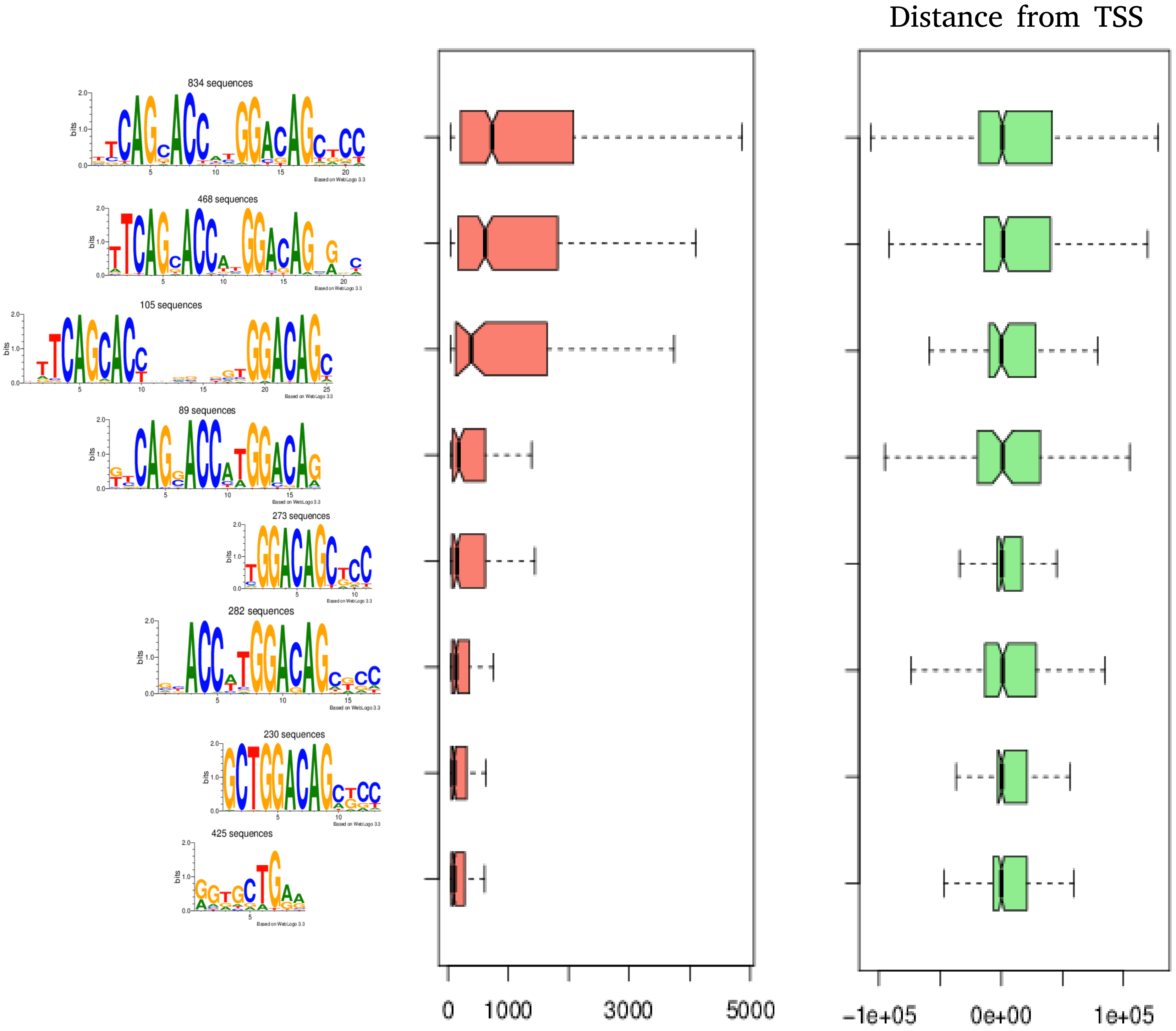

HL-60 (3683 sequences)

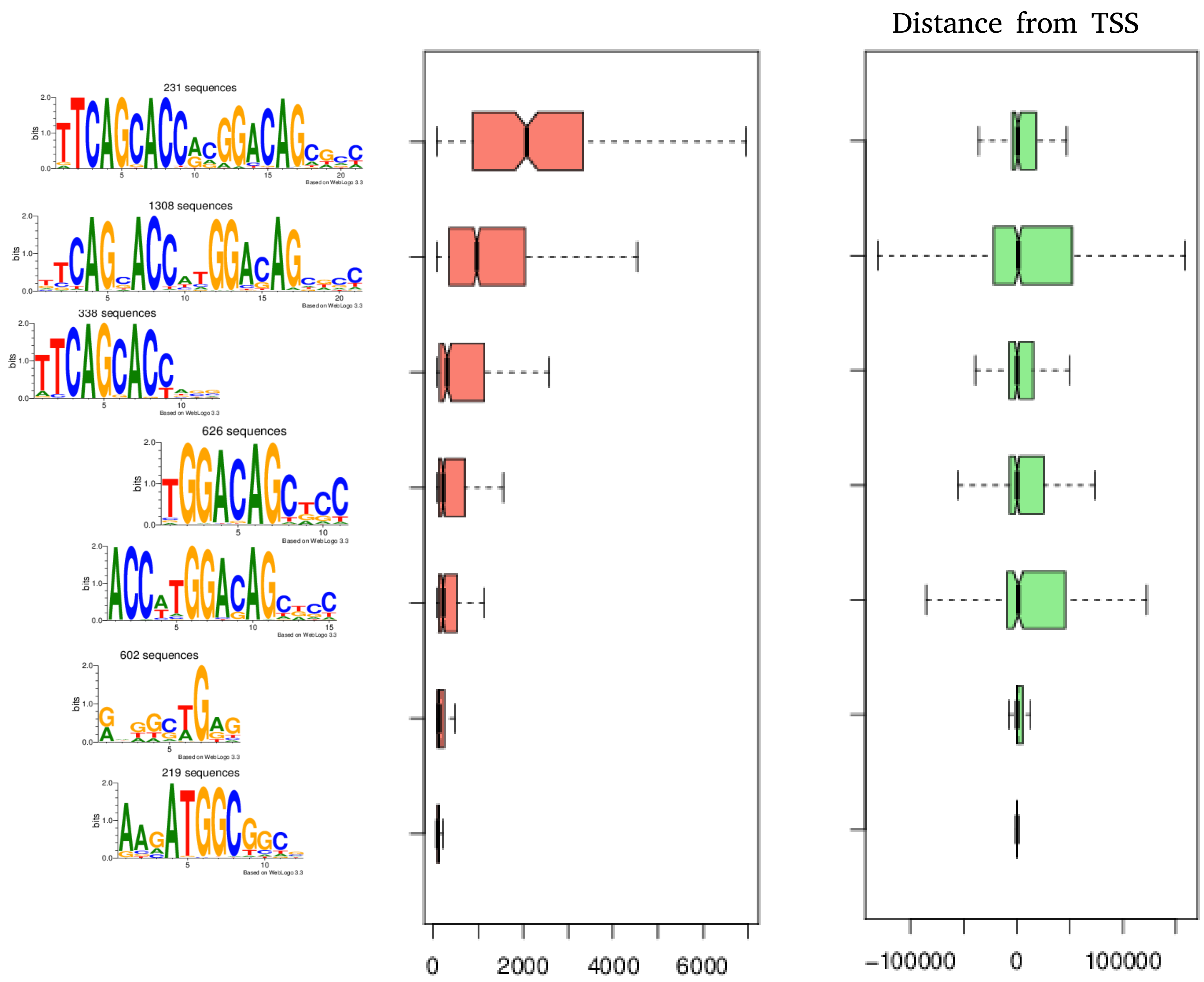

K562 (4810 sequences)

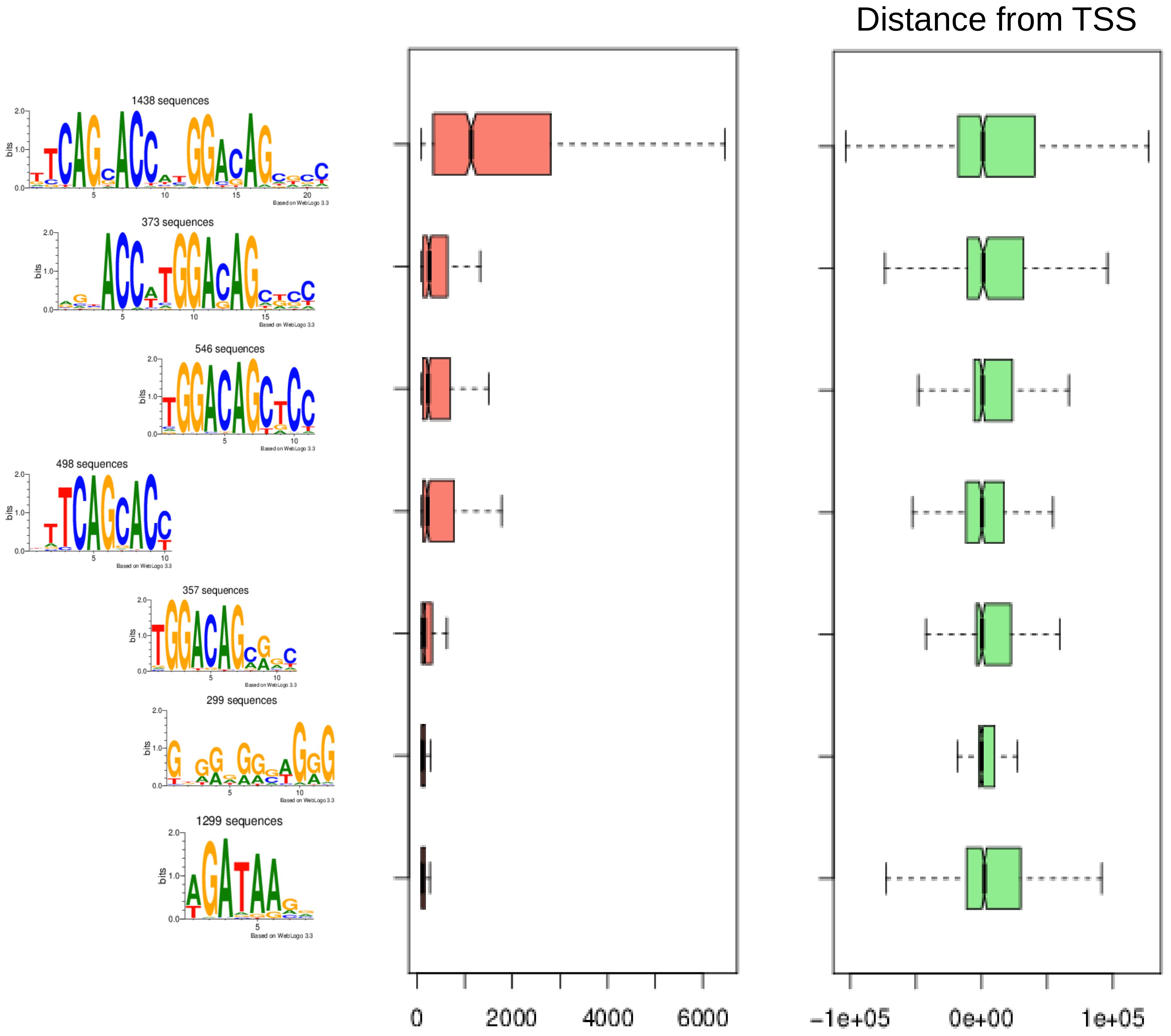

MCF-7 (3431 sequences)

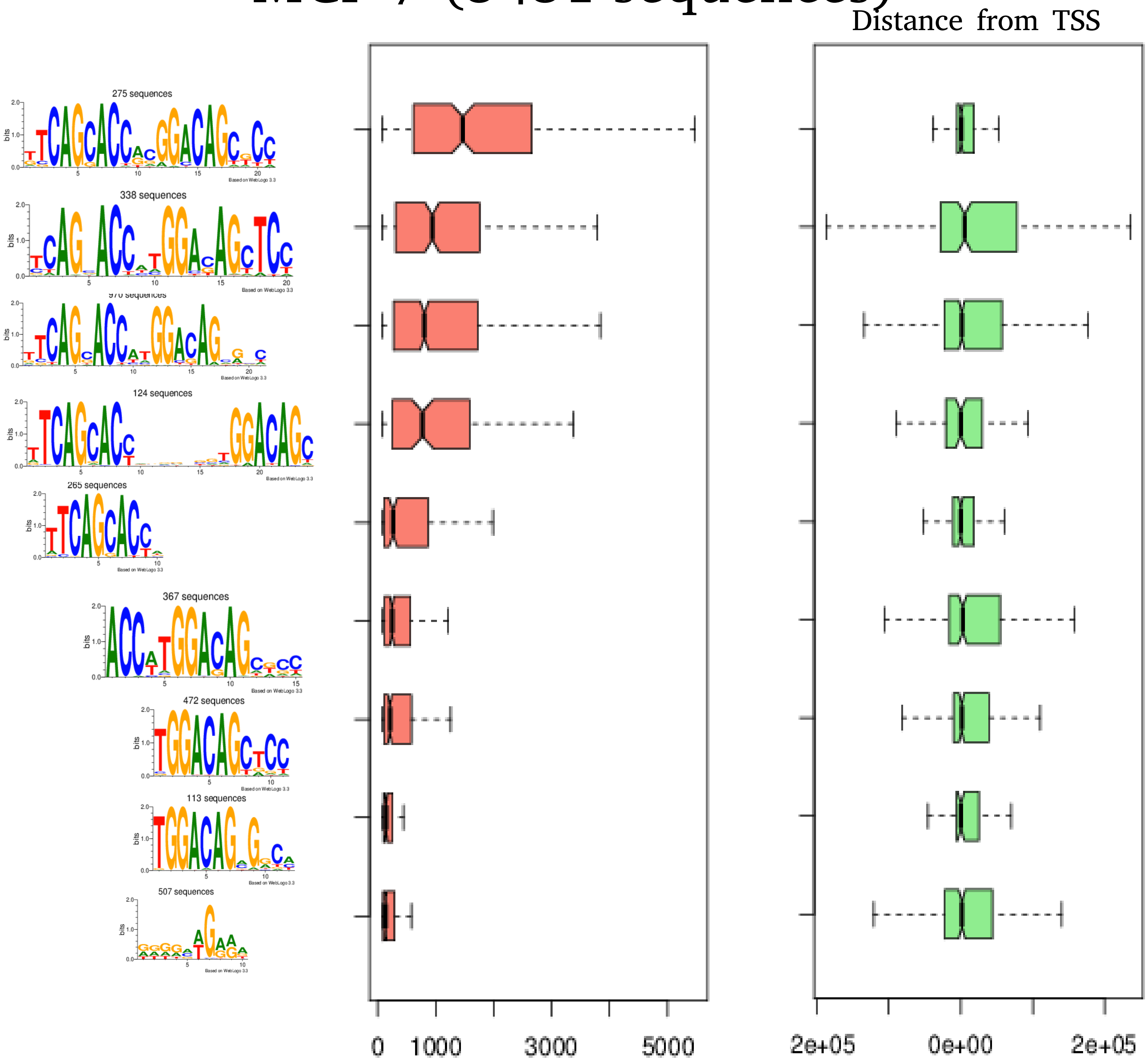

Neurons (5124 sequences)

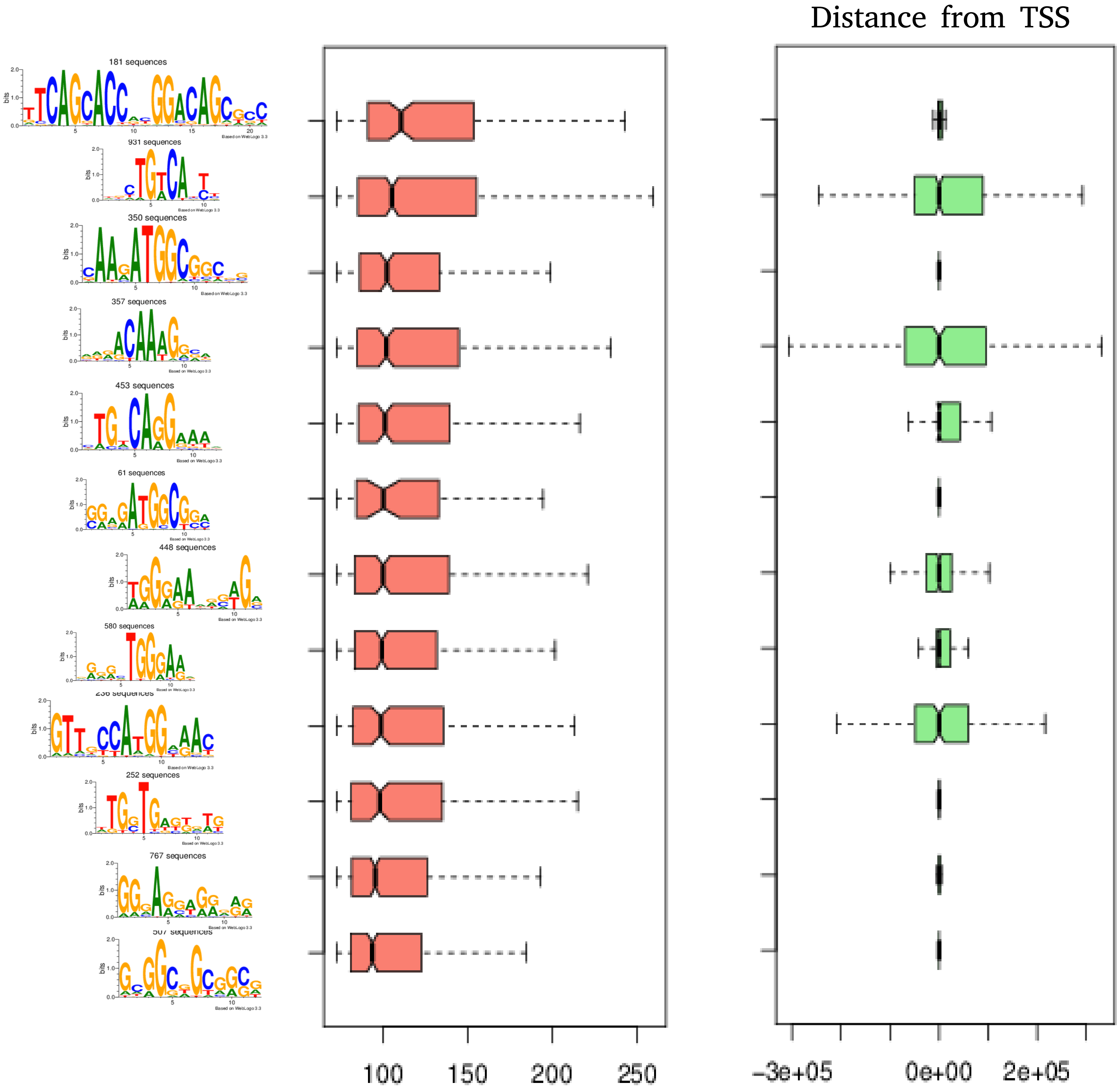

PANC-1 (2885 sequences)

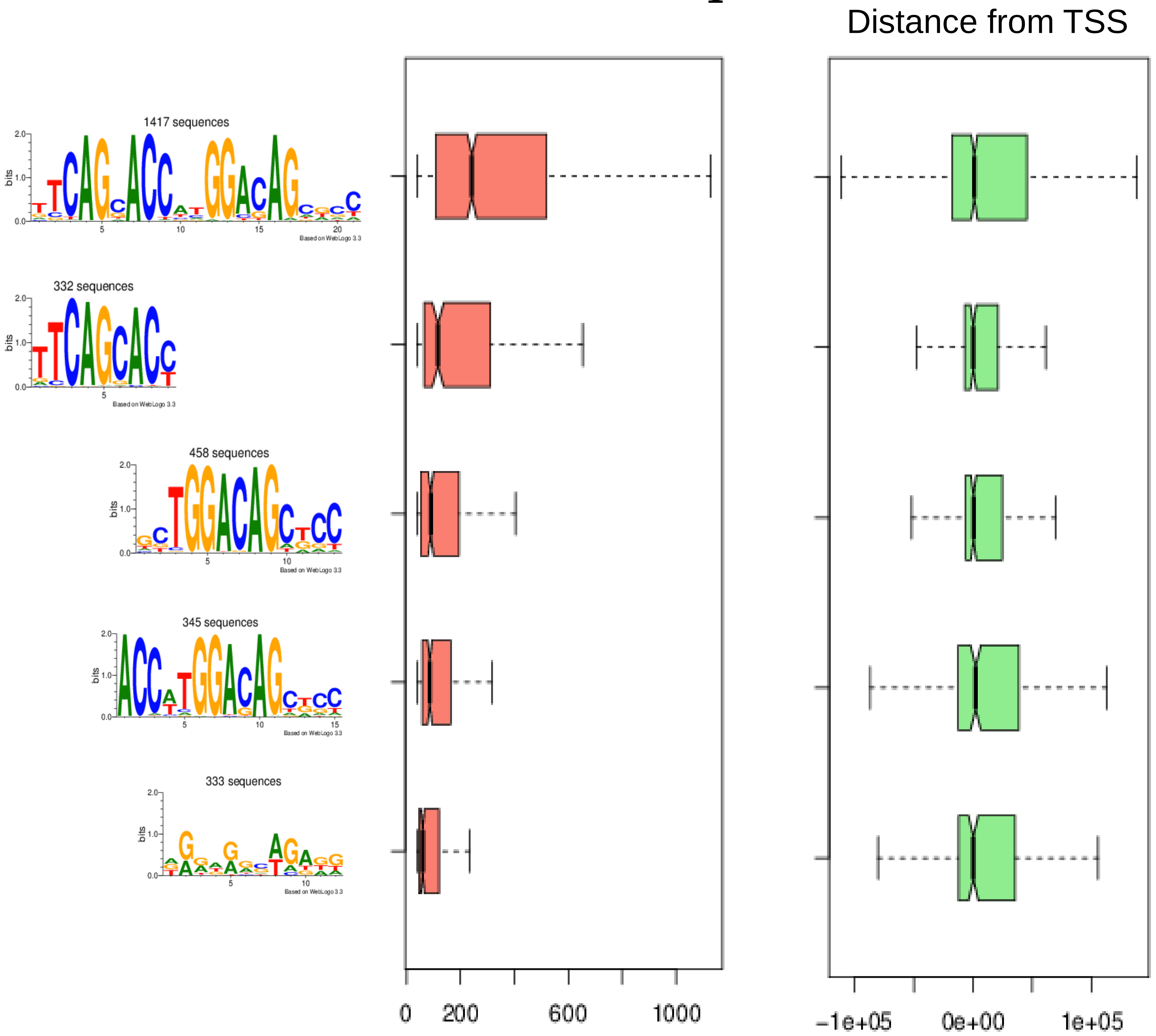

PFSK-1 (3167 sequences)

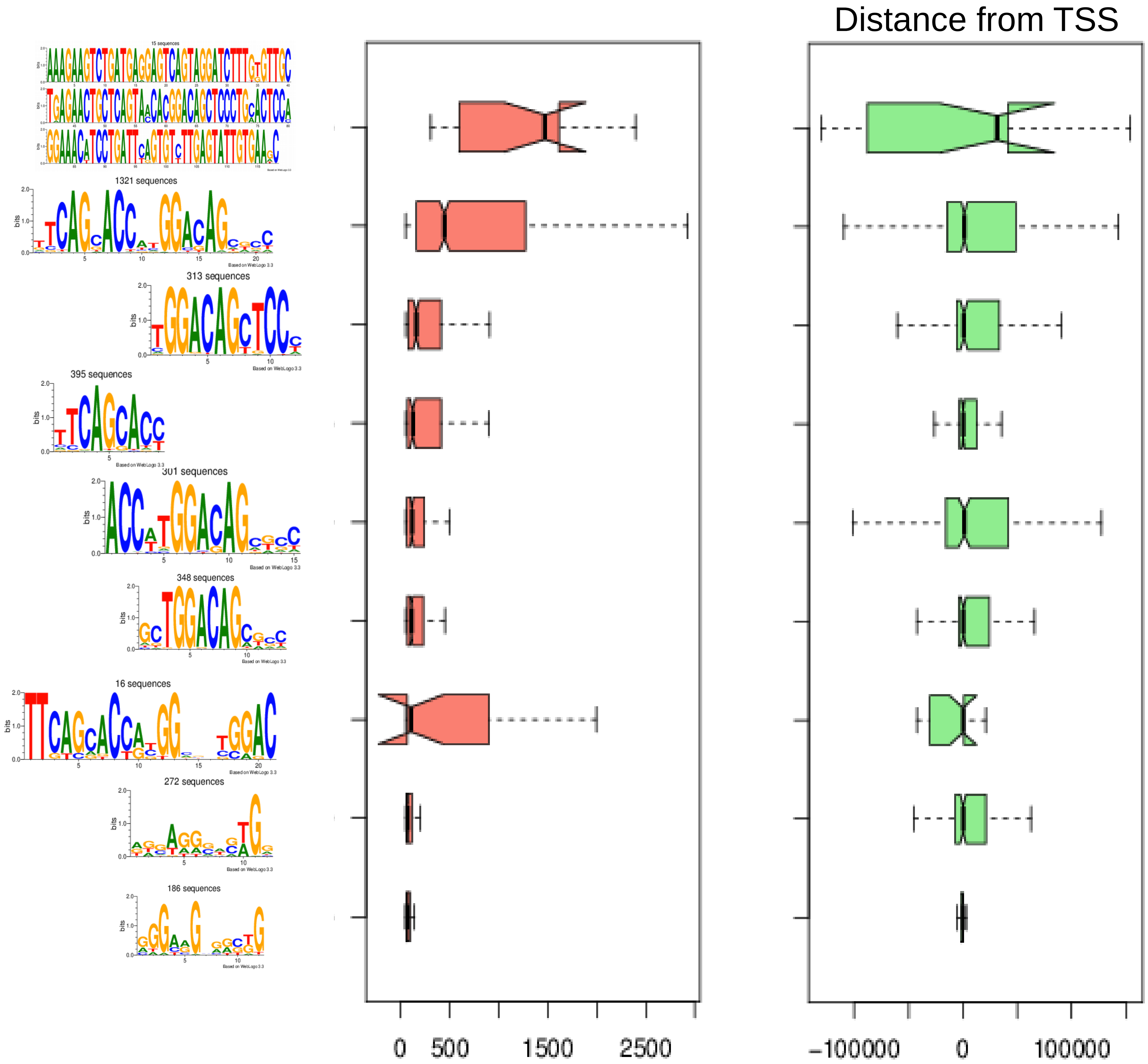

SK-N-SH (3610 sequences)

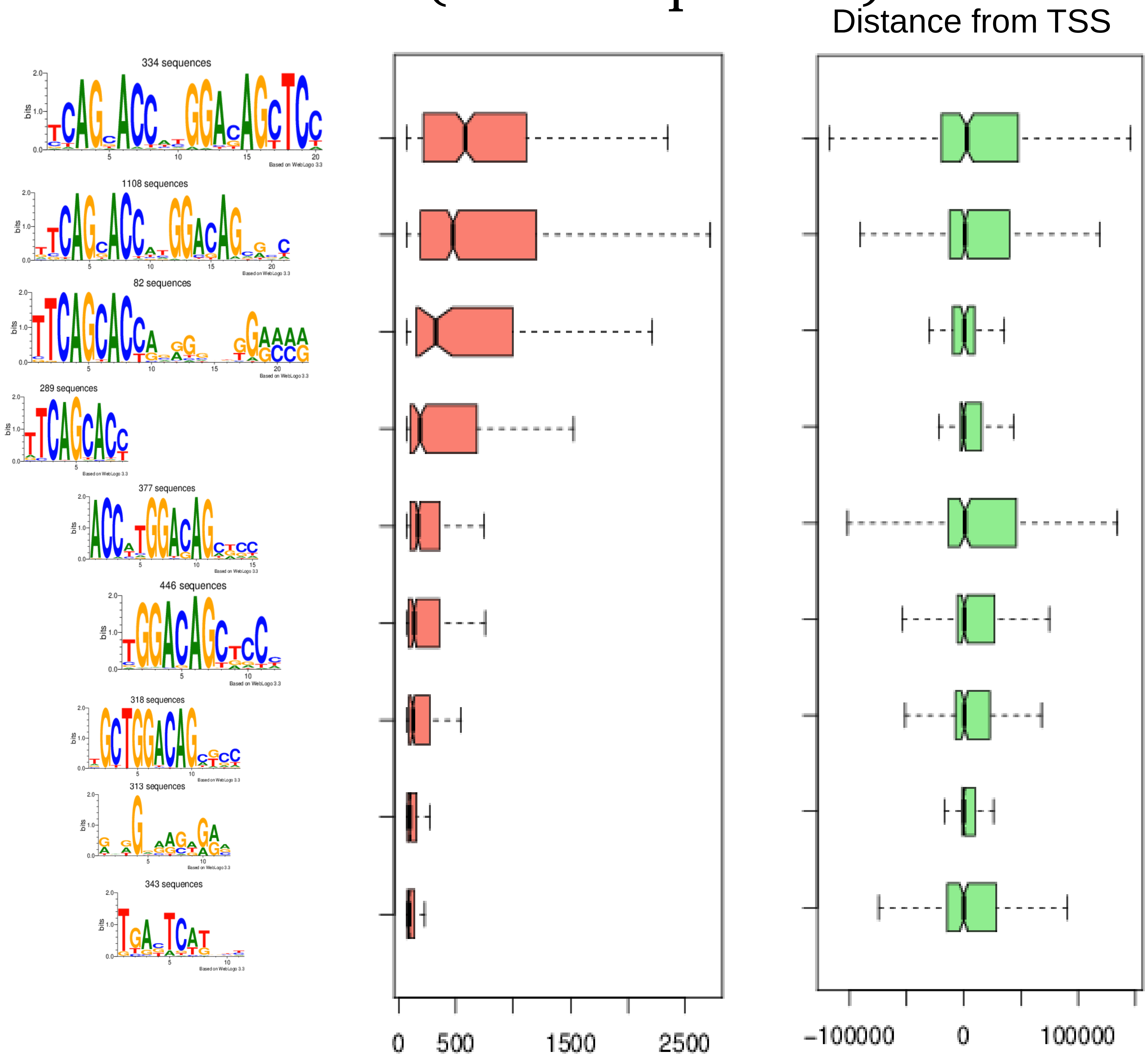

CD4+ Tcell (4177 sequences)

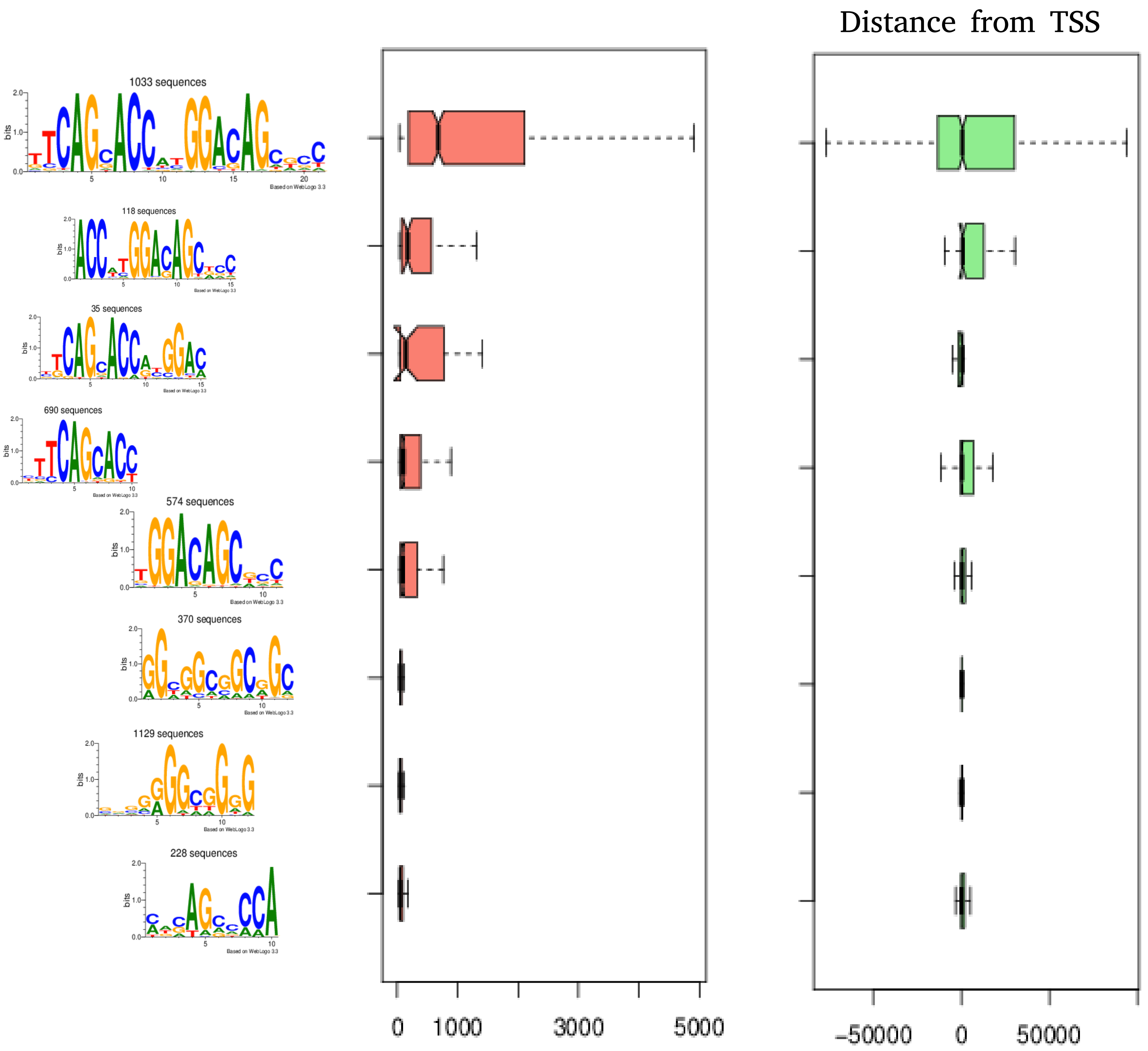

U87 (2238 sequences)

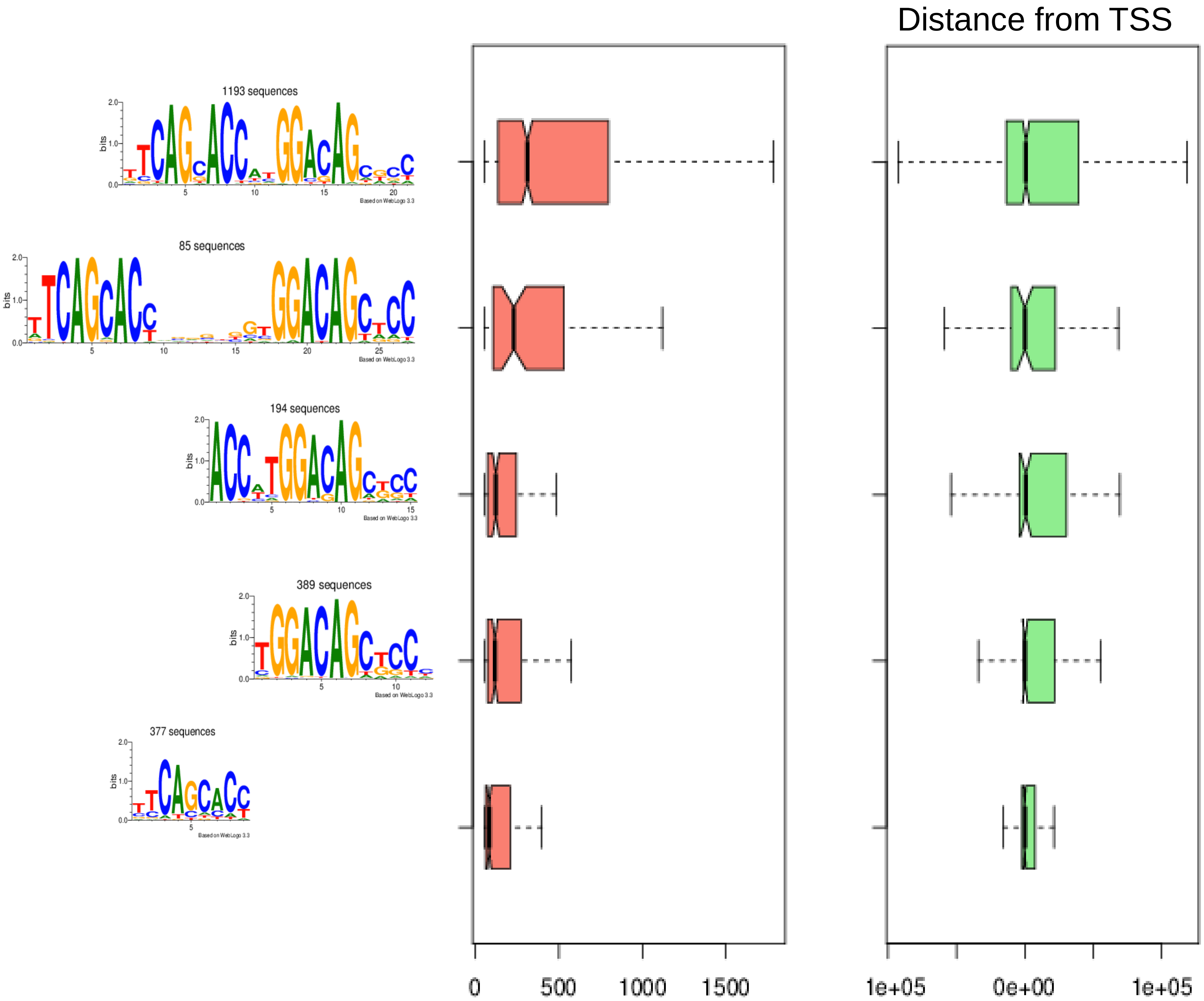

Supplement: S4 Fig — (PDF) [file pcbi.1006090.s004.pdf]
