# Supplementary material for: DIVERSITY in binding, regulation, and evolution revealed from high-throughput ChIP
Source: PLoS Comput Biol. 2018 Apr 23;14(4):e1006090. doi: 10.1371/journal.pcbi.1006090 (PMC5933800; doi:10.1371/journal.pcbi.1006090)

JASPAR
(Homo sapiens)

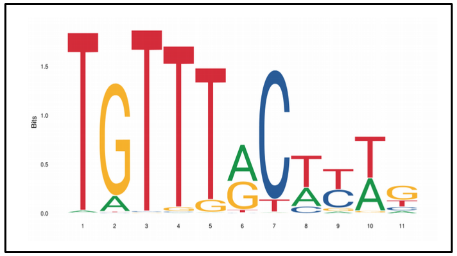

FOXA1 (529 sequences)

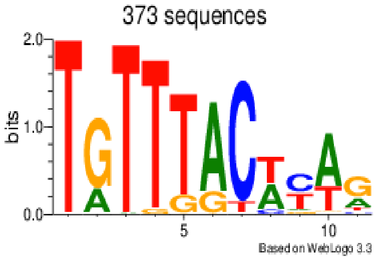

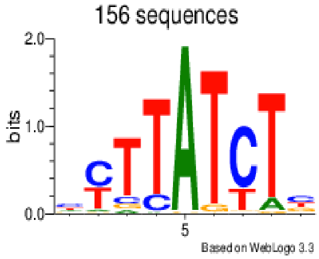

Enrichment

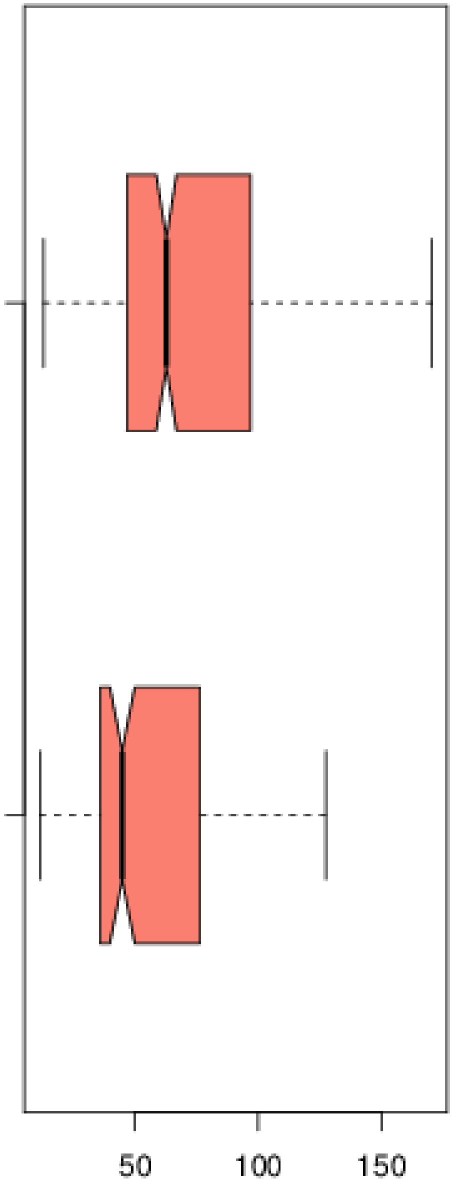

Distance from TSS

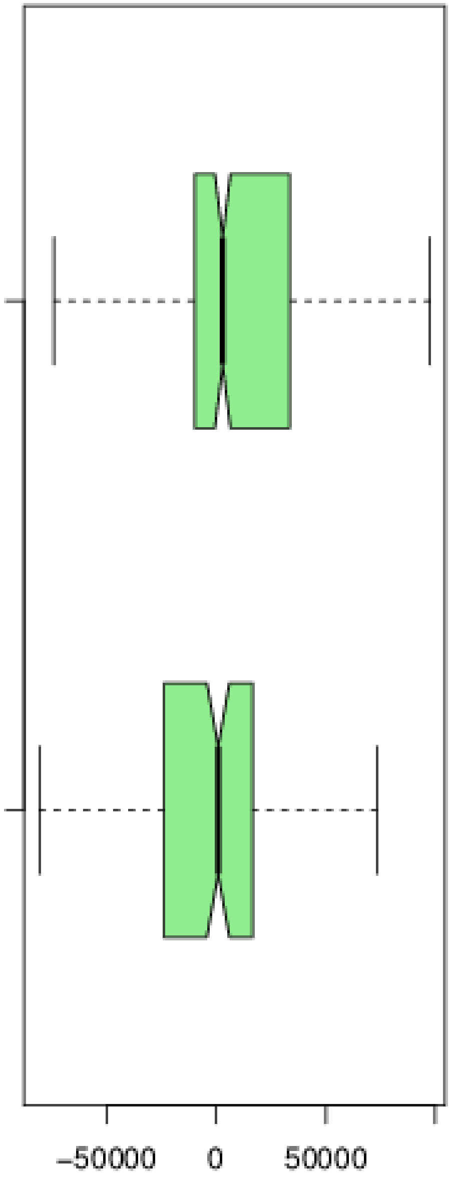

GATA1 (2306 sequences)

JASPAR
(Mus musculus)

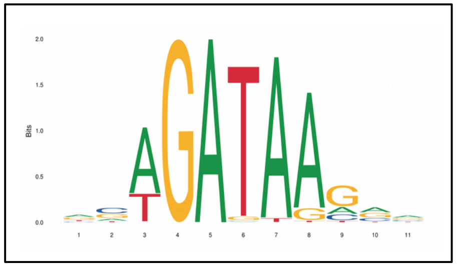

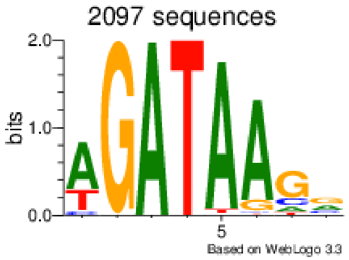

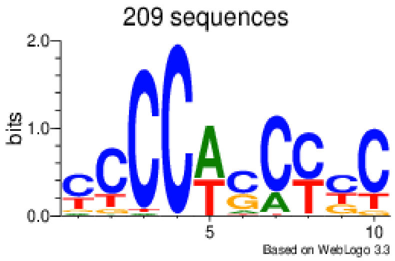

Enrichment

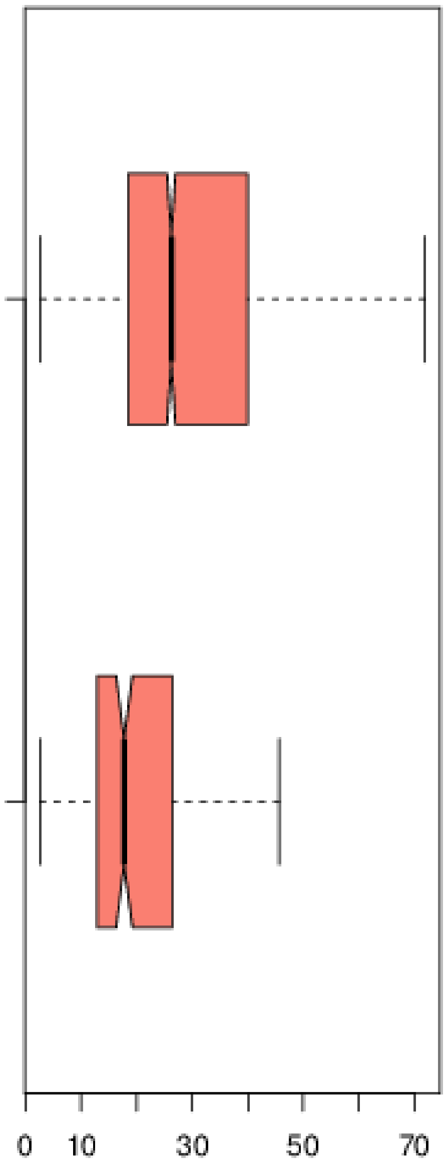

Distance from TSS

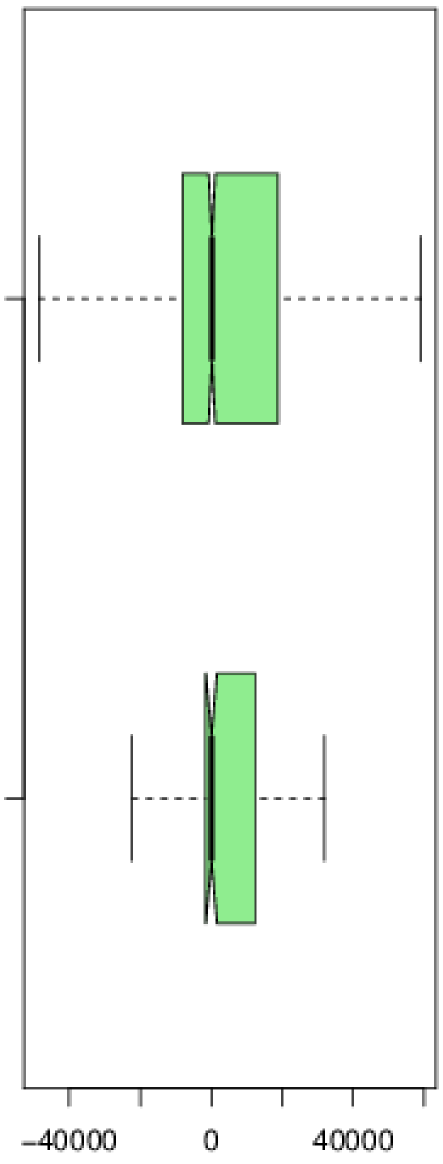

JASPAR
(Homo sapiens)

GATA2 (3329 sequences)

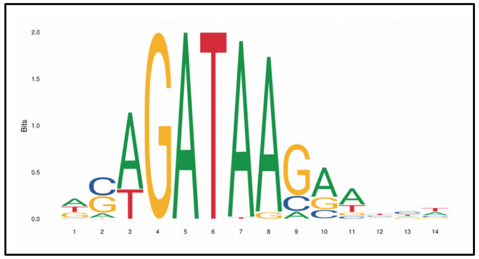

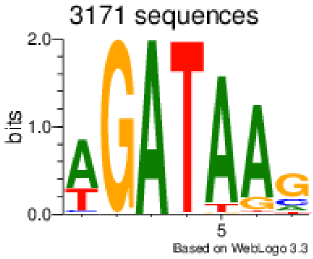

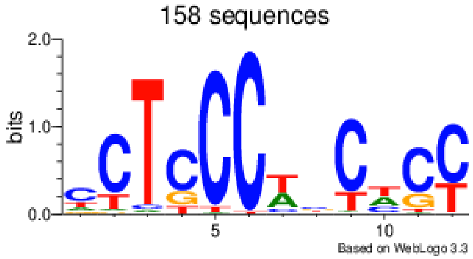

Enrichment

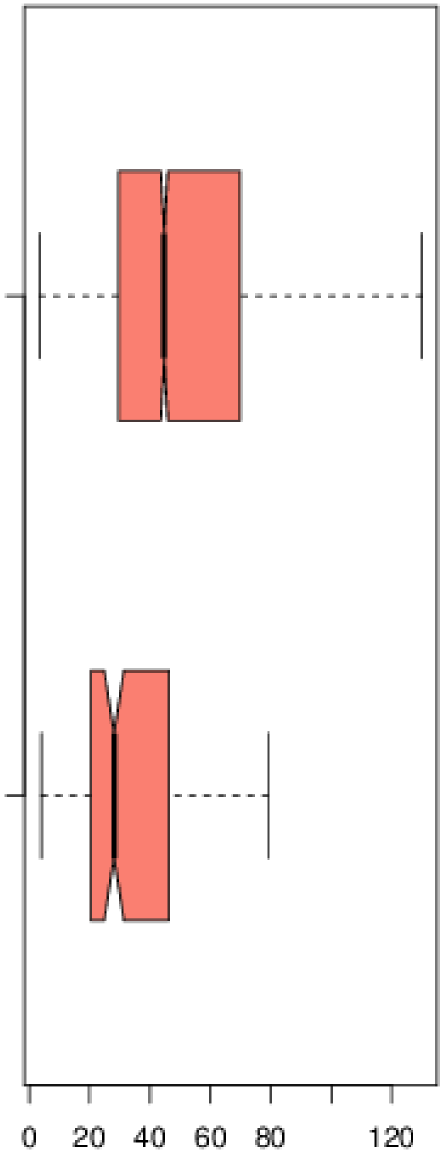

Distance from TSS

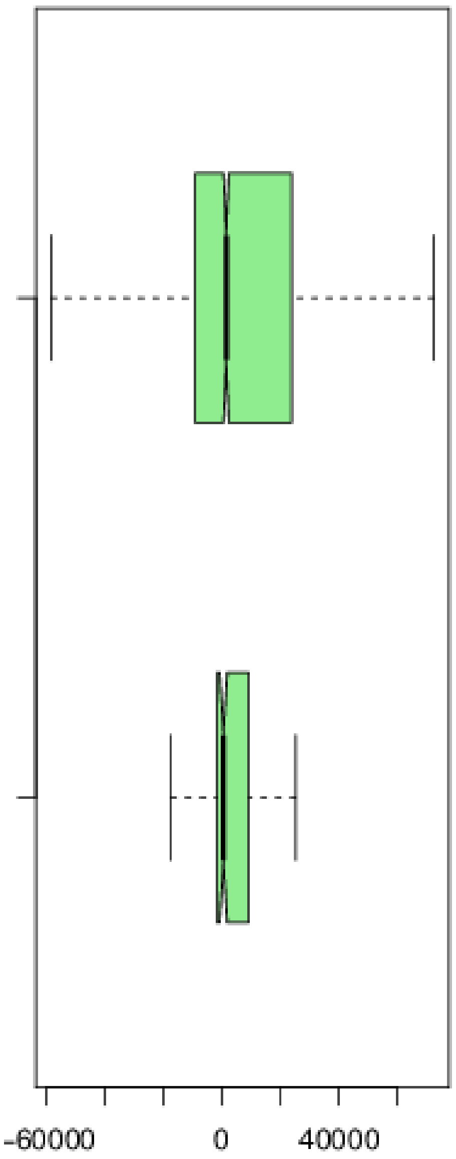

JASPAR
(Homo sapiens)

USF1 (2311 sequences)

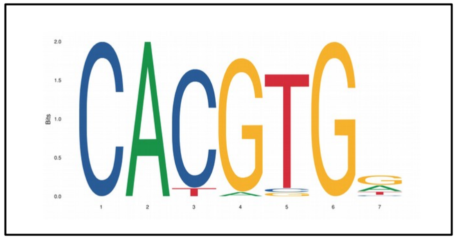

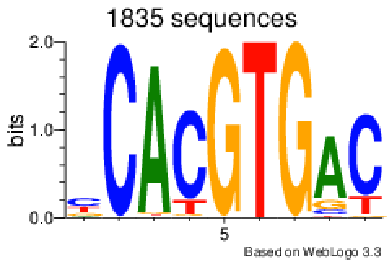

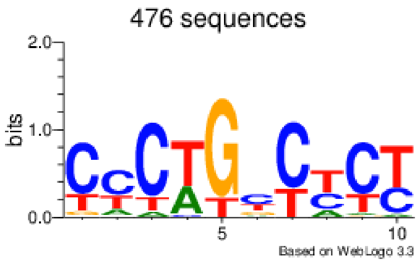

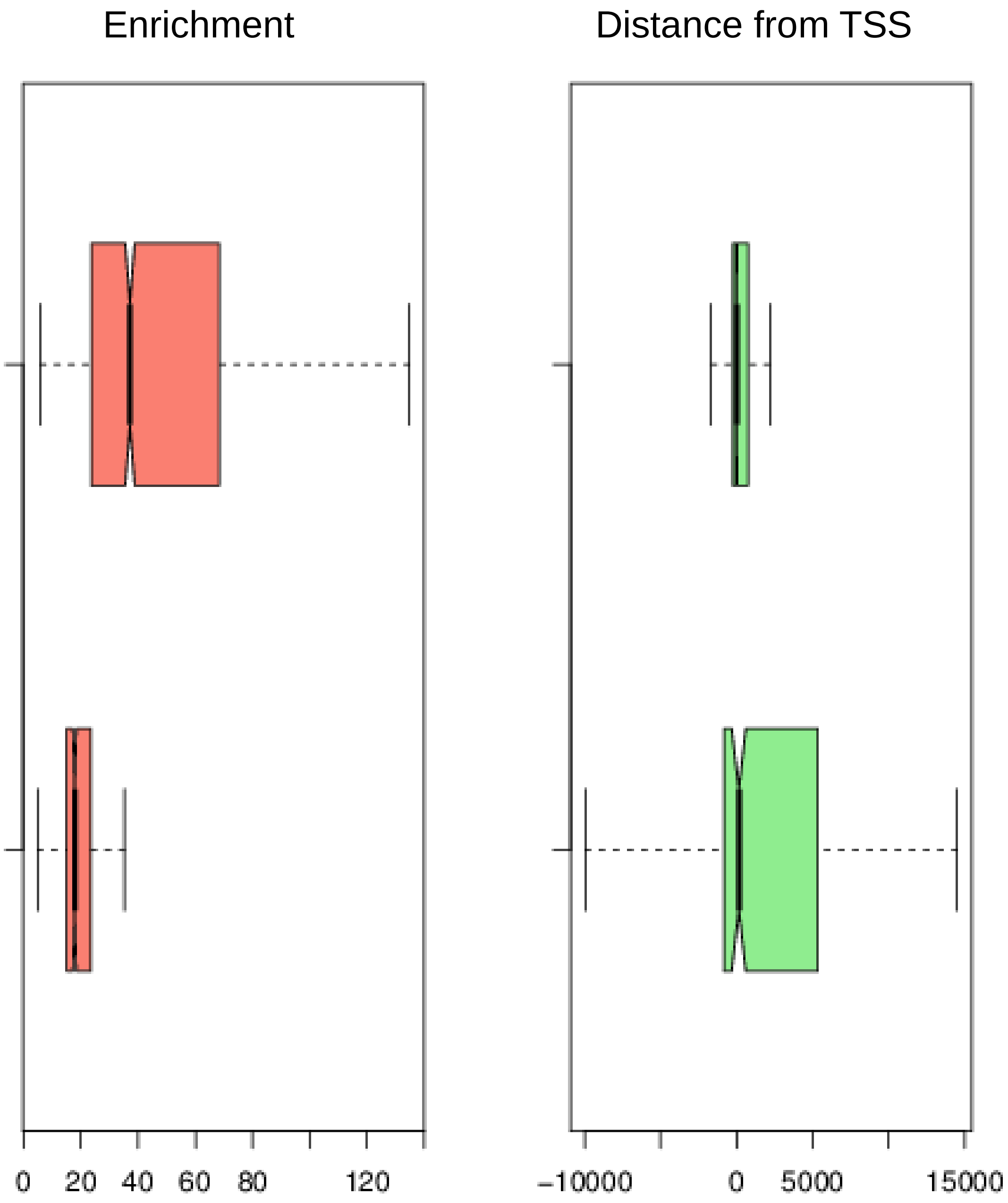

JASPAR
(Homo sapiens)

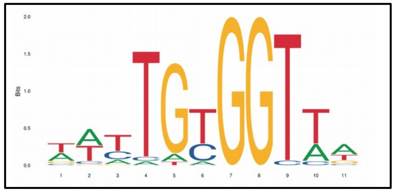

RUNX1 (2644 sequences)

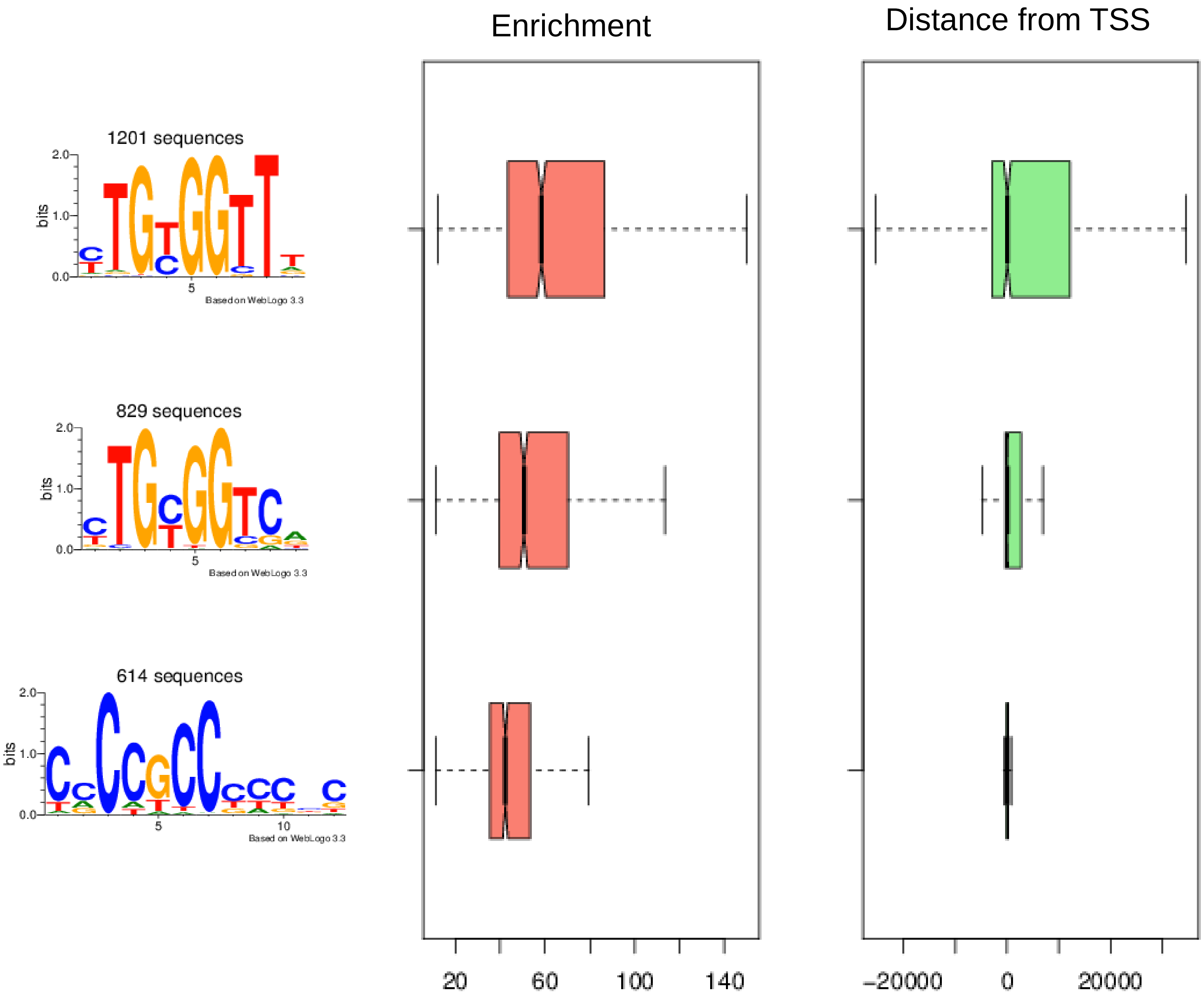

JASPAR
(Homo sapiens)

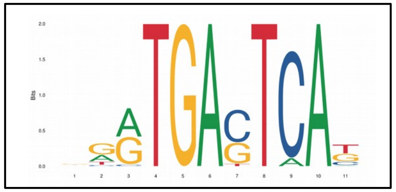

JUNB (2540 sequences)

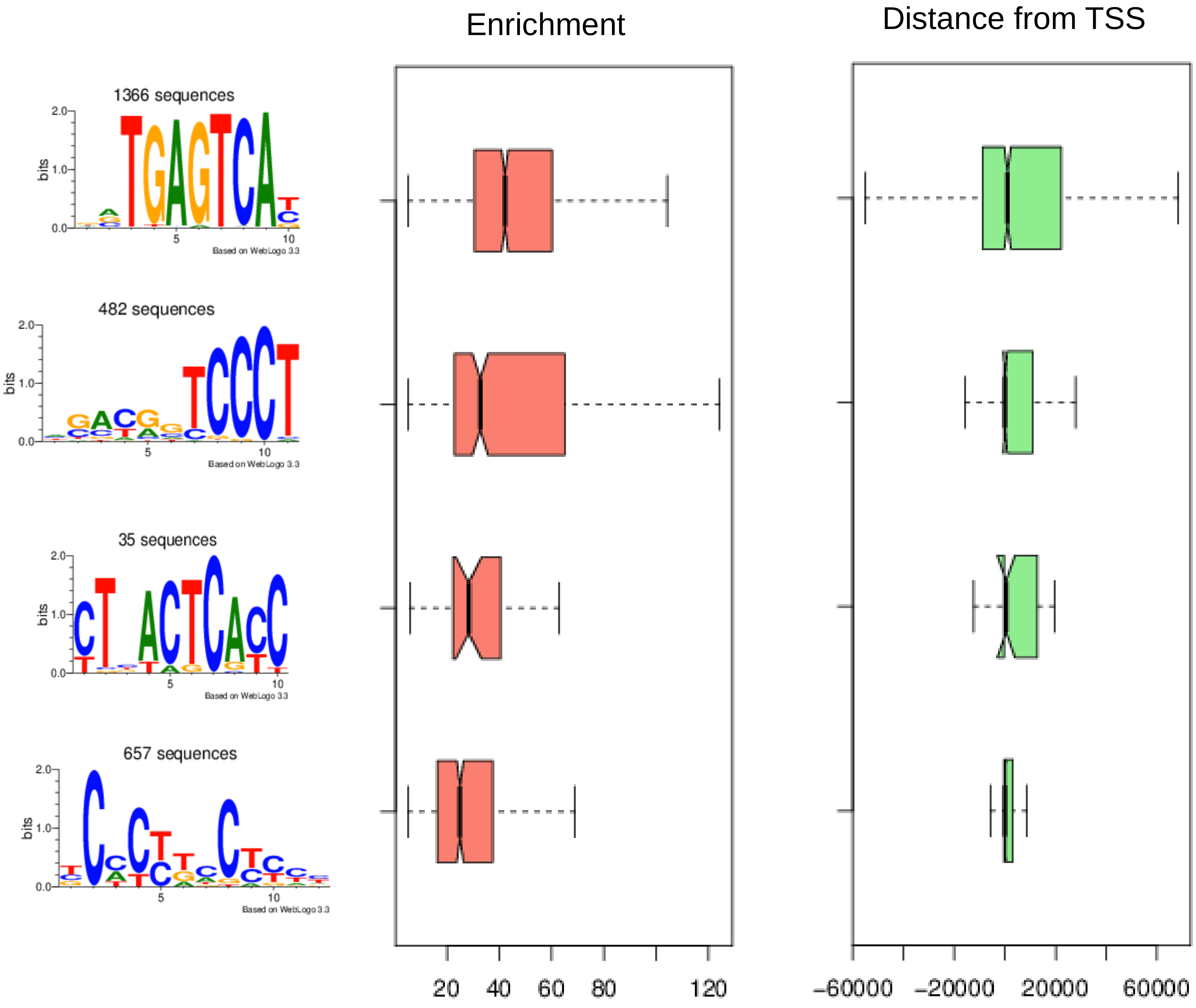

JASPAR
(Homo sapiens)

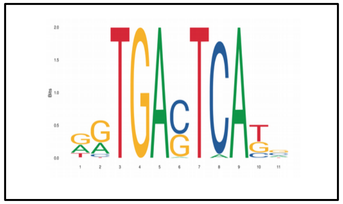

FOSL1 (4609 sequences)

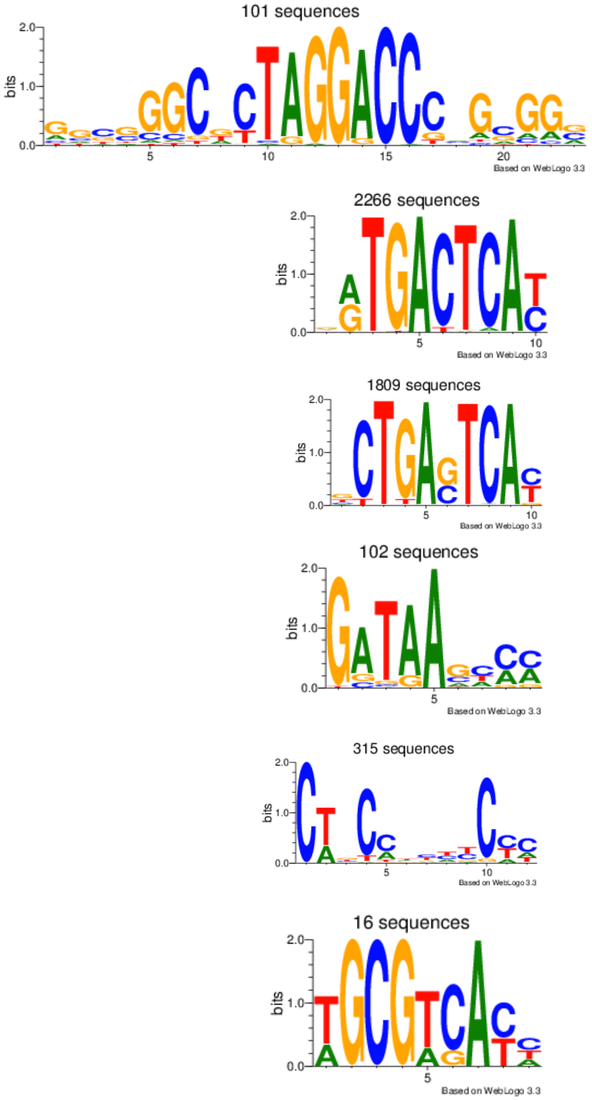

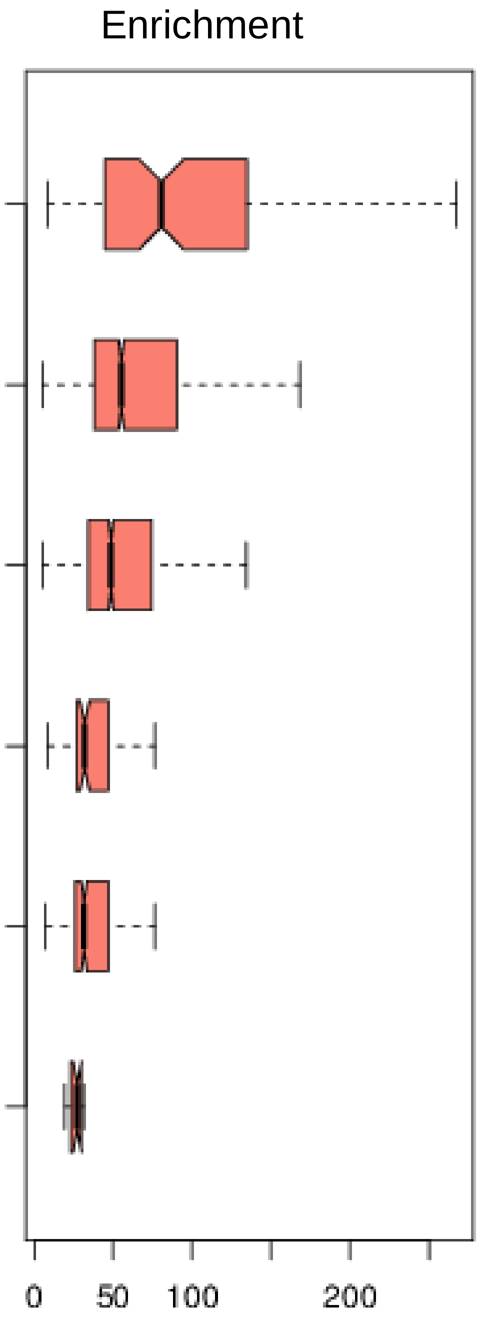

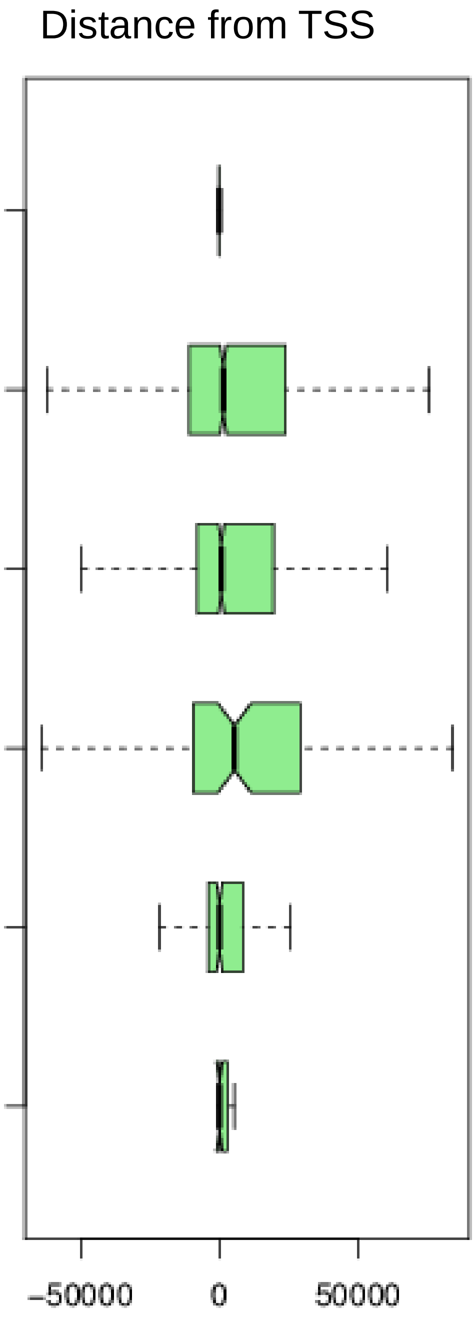

IRF2 (7158 sequences)

JASPAR
(Homo sapiens)

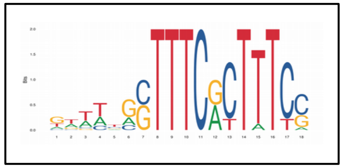

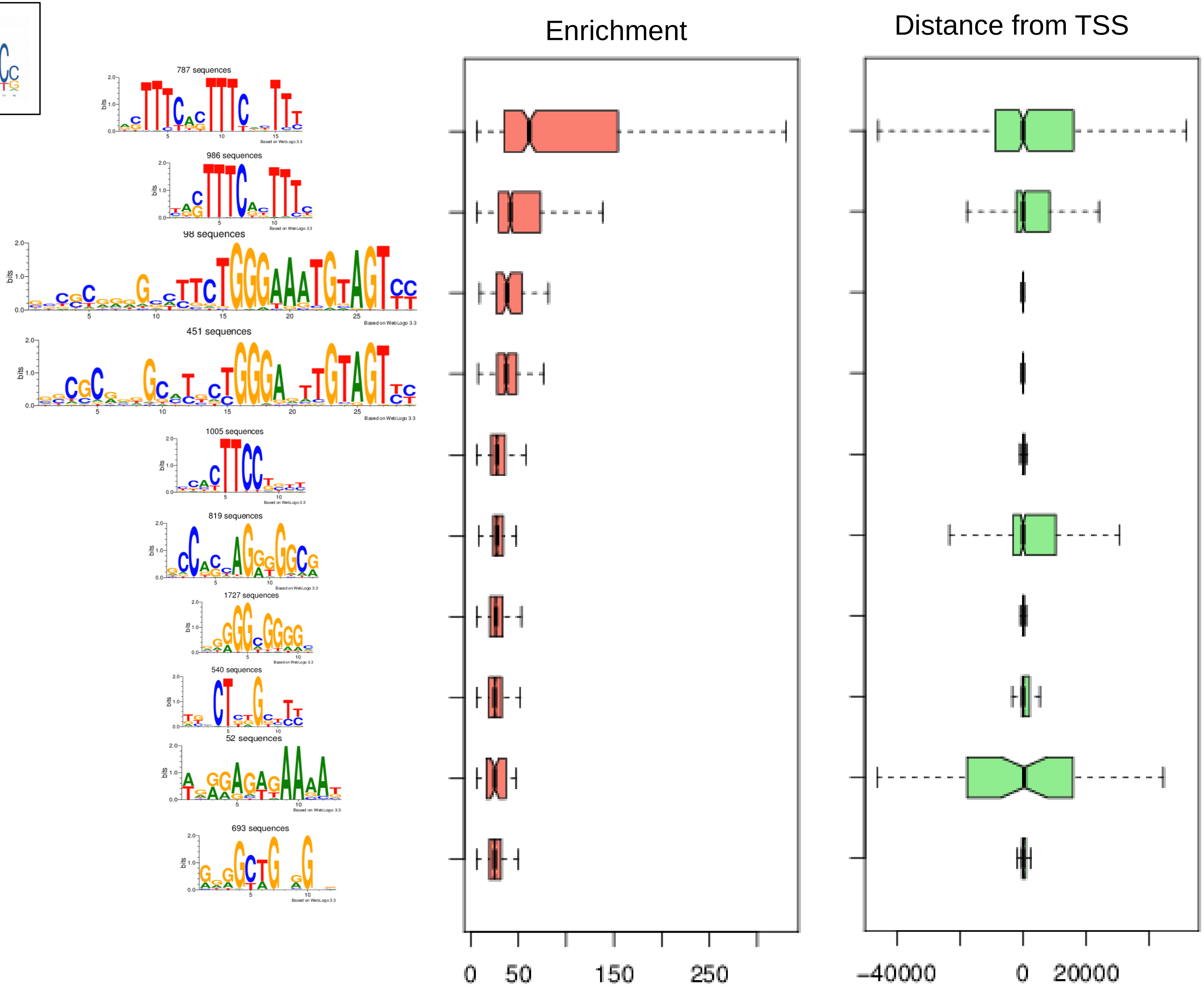

THAP1 (4363 sequences)

JASPAR
(Homo sapiens)

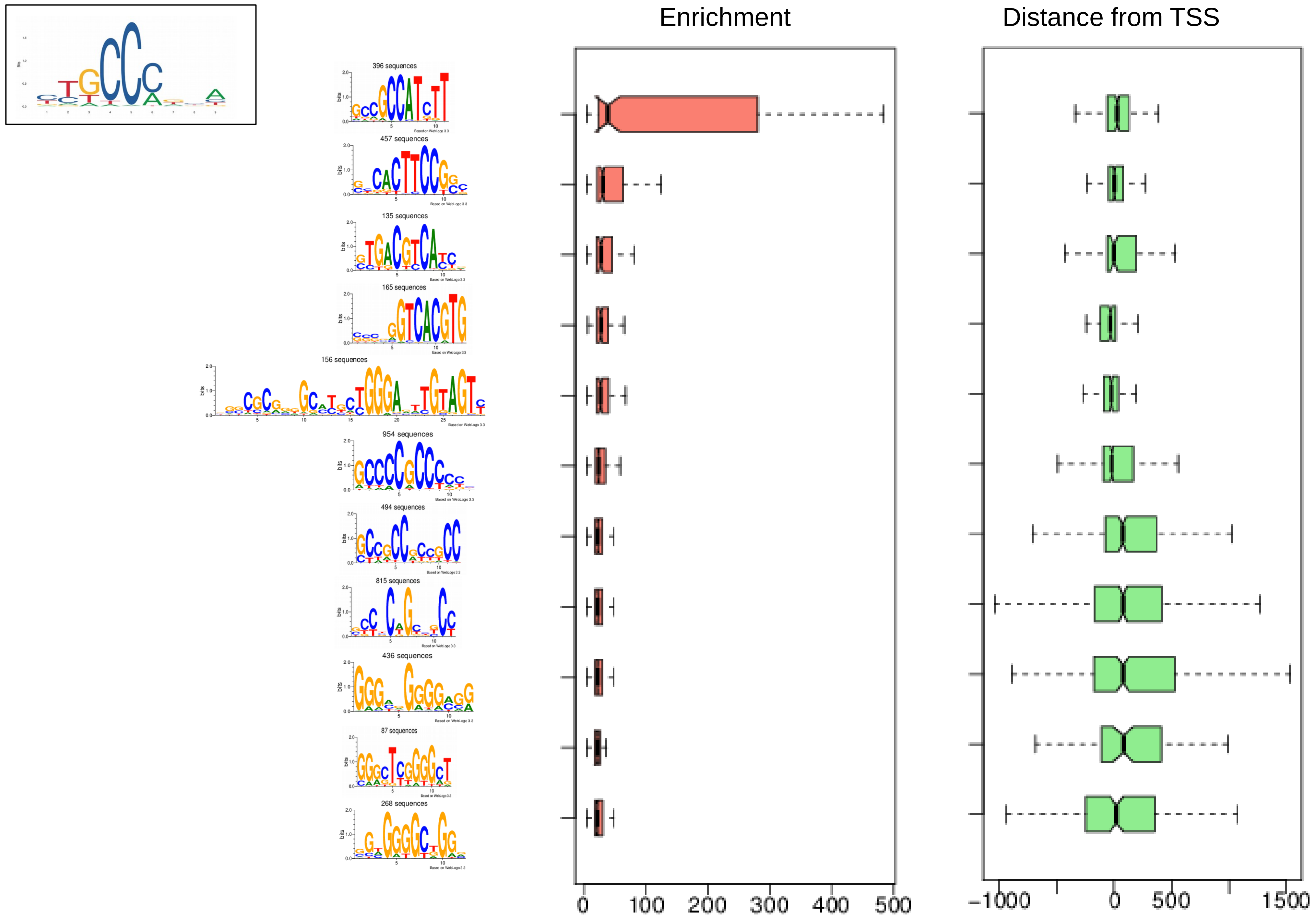

P300 (17,202 sequences)

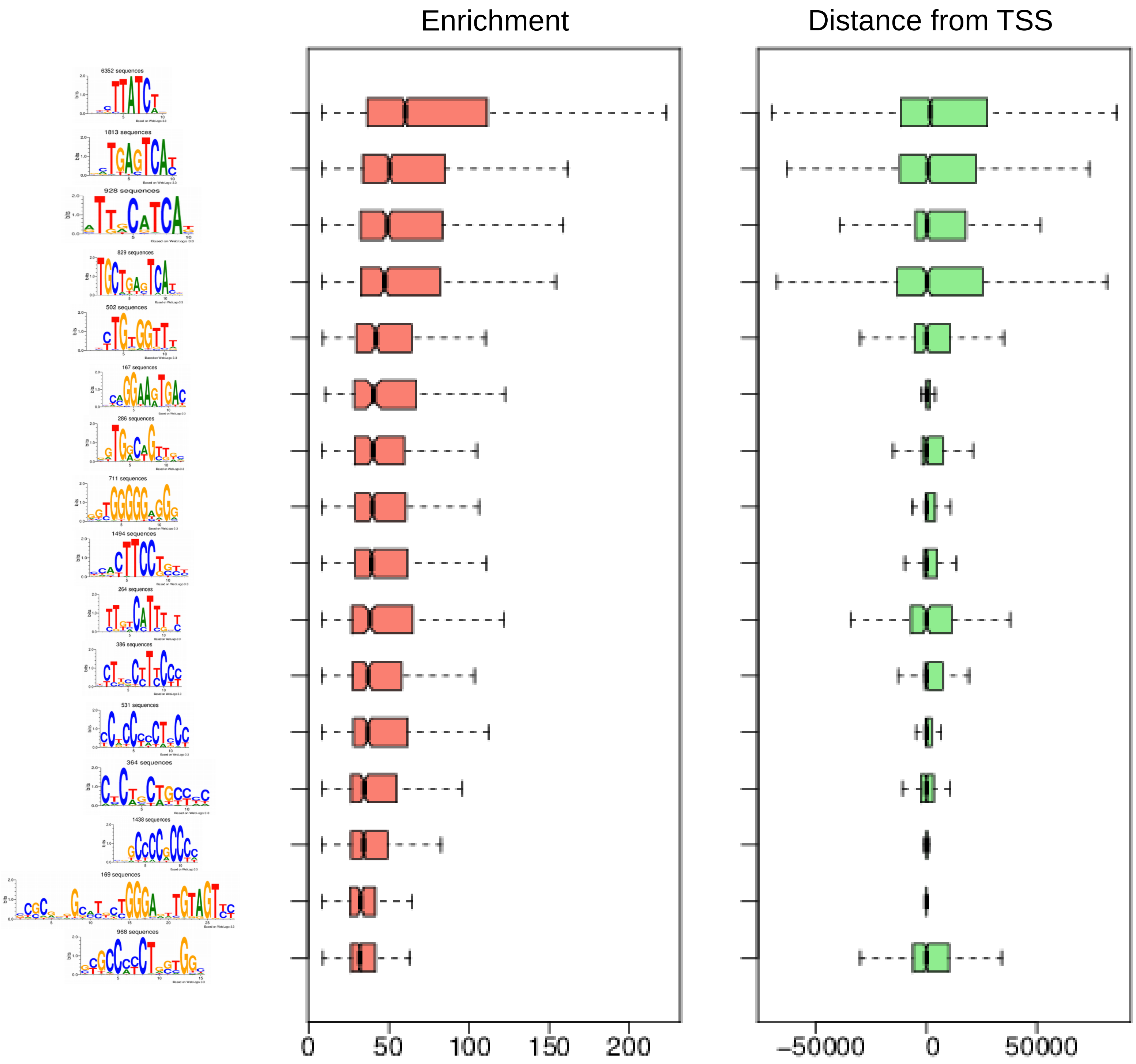

Supplement: S5 Fig — Logo from JASPAR is shown when available. (PDF) [file pcbi.1006090.s005.pdf]
