# Supplementary material for: DIVERSITY in binding, regulation, and evolution revealed from high-throughput ChIP
Source: PLoS Comput Biol. 2018 Apr 23;14(4):e1006090. doi: 10.1371/journal.pcbi.1006090 (PMC5933800; doi:10.1371/journal.pcbi.1006090)
Supplement: S6 Fig — (PDF) [file pcbi.1006090.s006.pdf]

(A) MEME output on REST

A549

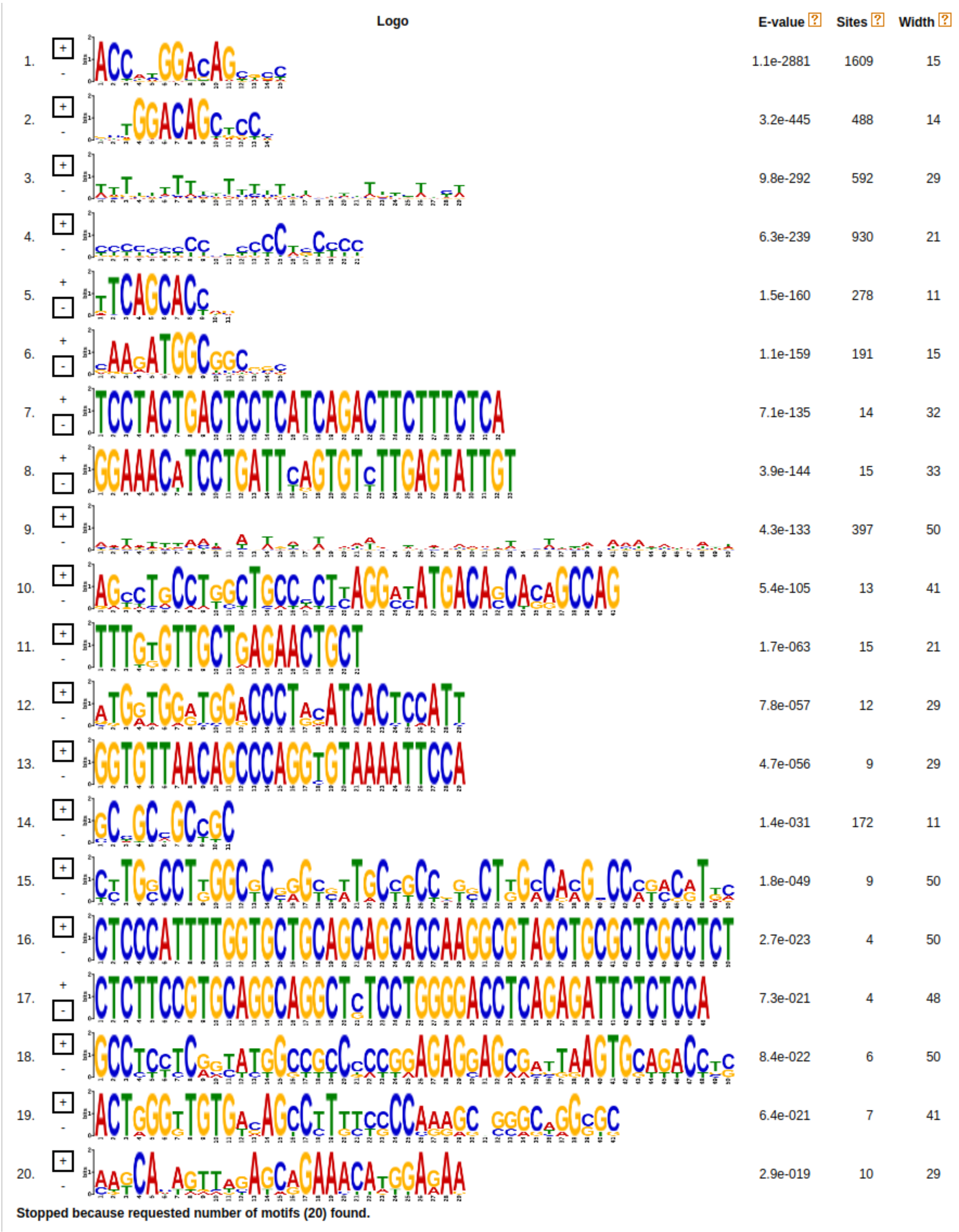

ECC-1

		Logo	E-value	Sites	Width
1.	+ -	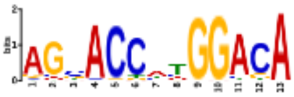	5.3e-3756	2451	13
2.	+ -	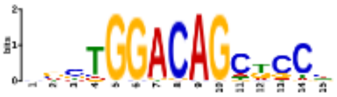	1.7e-1319	1202	15
3.	+ -	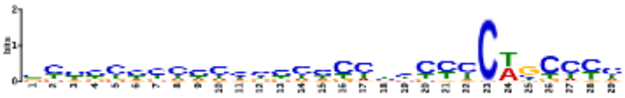	5.6e-385	1188	29
4.	+ -	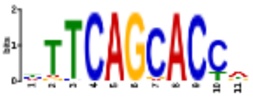	5.0e-284	400	11
5.	+ -	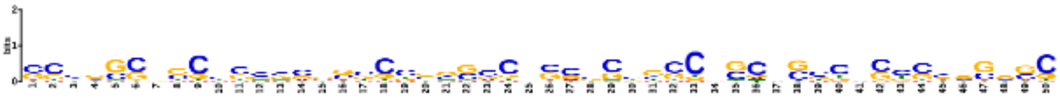	1.2e-250	395	50
6.	+ -	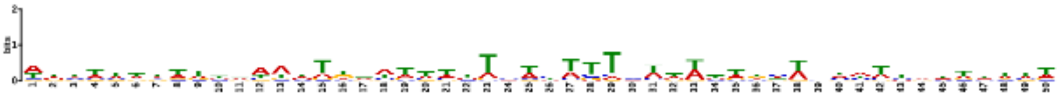	2.2e-193	374	50
7.	+ -	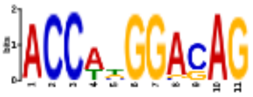	2.2e-046	134	11
8.	+ -	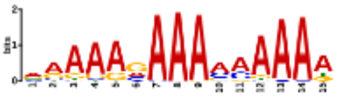	9.1e-038	101	15
9.	+ -	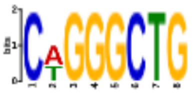	9.2e-032	263	8
10.	+ -	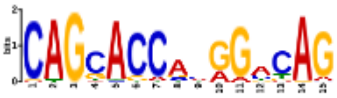	1.2e-013	60	15
11.	+ -	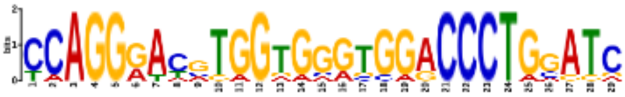	3.2e-012	9	29
12.	+ -	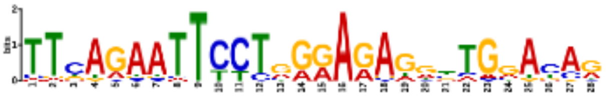	4.2e-014	19	28
13.	+ -	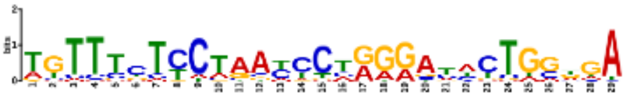	1.0e-008	33	29
14.	+ -	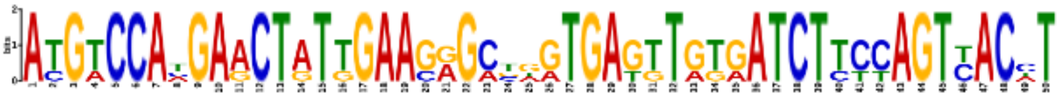	7.4e-005	4	50
15.	+ -	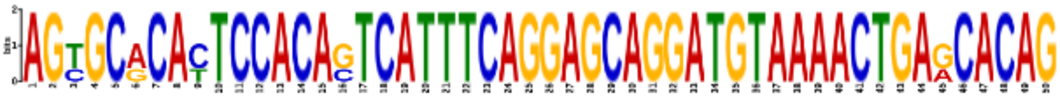	3.8e-004	3	50
16.	+ -	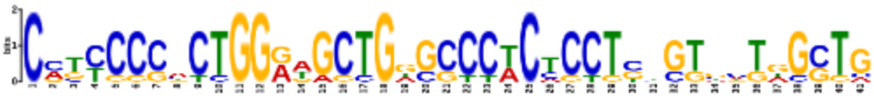	1.3e-002	8	41
17.	+ -	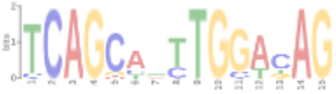	2.6e-001	22	15
18.	+ -	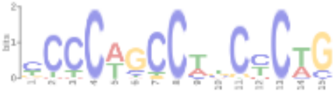	4.0e+001	90	15
19.	+ -	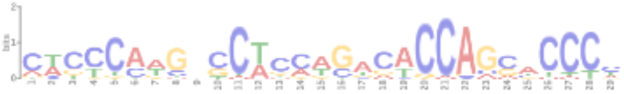	9.4e+001	24	29
20.	+ -	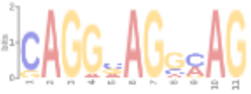	1.1e+002	91	11

GM12878

	Logo	E-value	Sites	Width
1.	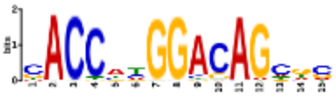	3.1e-3262	1600	15
2.	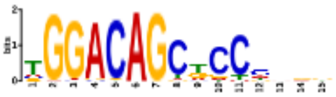	1.7e-490	494	15
3.	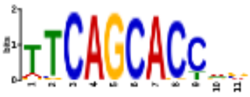	9.1e-266	355	11
4.	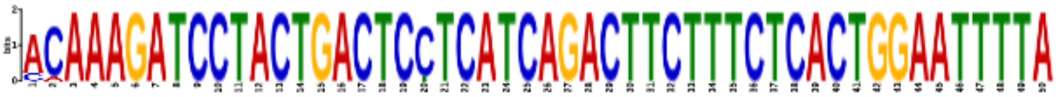	4.7e-206	12	50
5.	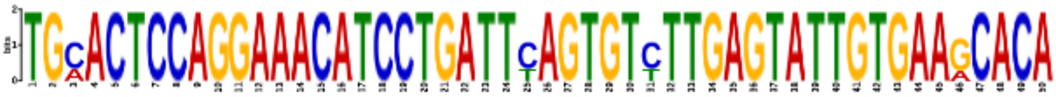	1.2e-219	13	50
6.	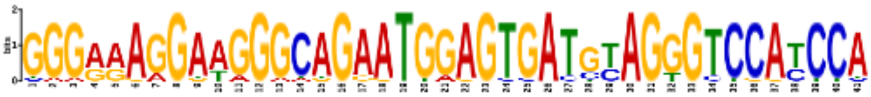	1.6e-194	19	41
7.	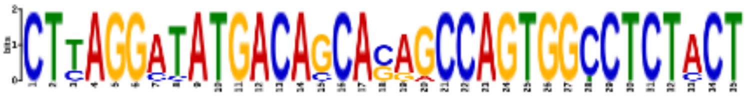	2.1e-104	12	35
8.	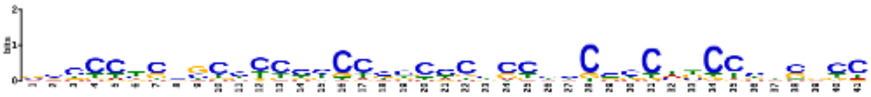	3.4e-205	343	41
9.	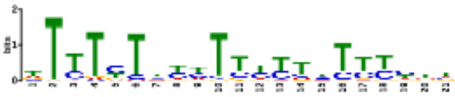	2.0e-111	257	21
10.	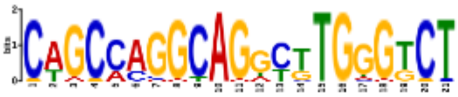	4.4e-085	29	21
11.	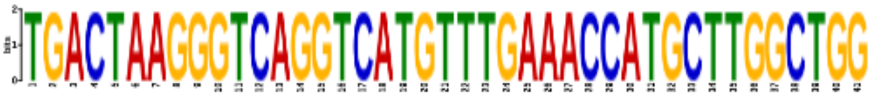	1.6e-067	7	41
12.	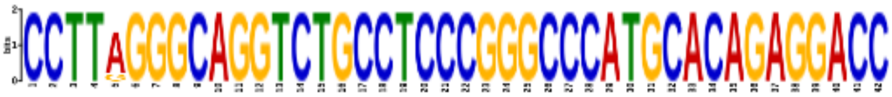	7.3e-064	7	42
13.	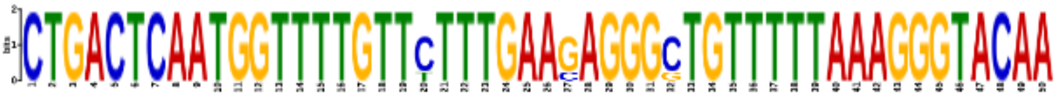	2.0e-046	5	50
14.	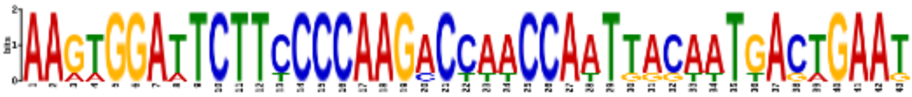	6.4e-039	6	43
15.	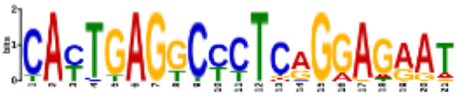	2.2e-037	19	21
16.	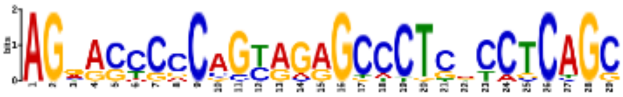	4.4e-037	16	29
17.	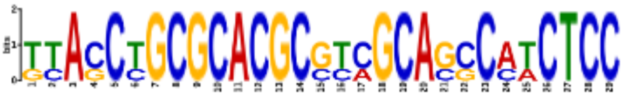	9.3e-037	10	29
18.	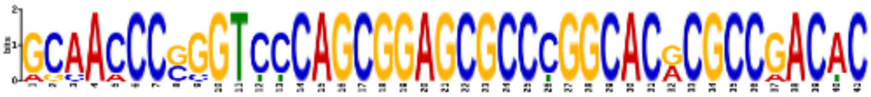	2.6e-045	7	41
19.	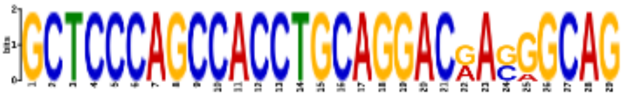	2.0e-028	7	29
20.	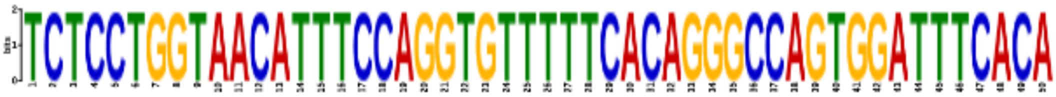	1.6e-027	4	50

H1ESC

	Logo	E-value	Sites	Width
1.	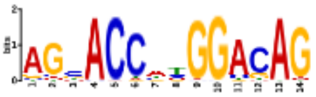	1.0e-6172	3310	14
2.	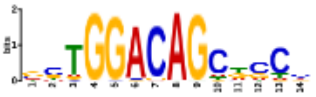	6.4e-2237	2112	14
3.	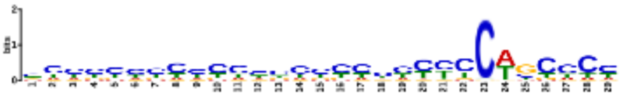	5.8e-682	1648	29
4.	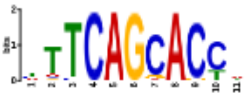	2.6e-506	852	11
5.	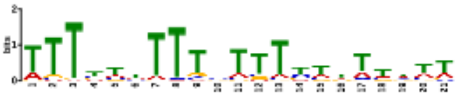	6.5e-173	431	21
6.	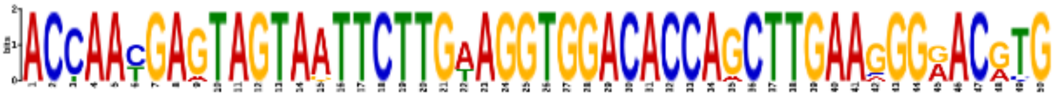	5.7e-132	10	50
7.	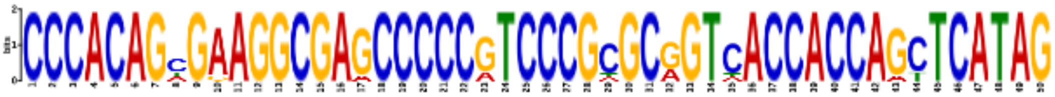	8.1e-127	10	50
8.	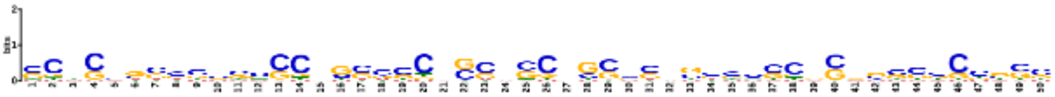	6.2e-257	544	50
9.	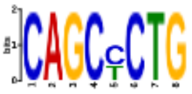	7.0e-056	396	8
10.	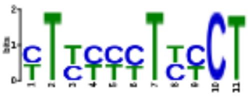	1.6e-062	372	11
11.	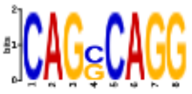	1.1e-052	408	8
12.	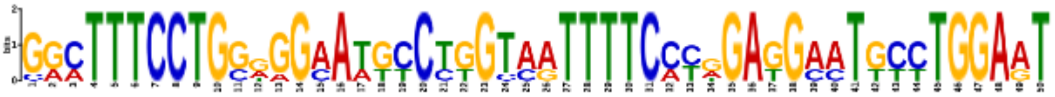	1.0e-040	7	50
13.	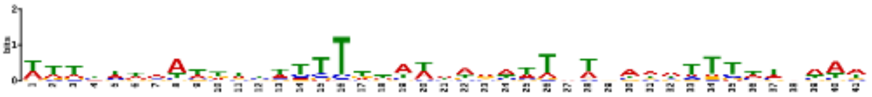	1.4e-039	254	41
14.	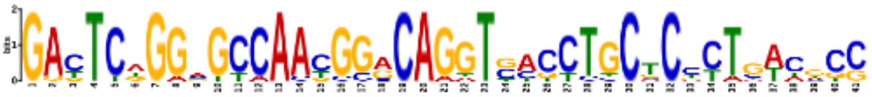	1.8e-034	11	41
15.	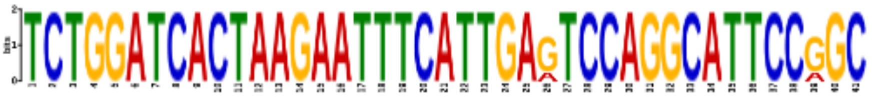	2.8e-029	5	41
16.	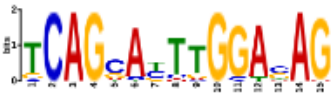	7.3e-017	40	15
17.	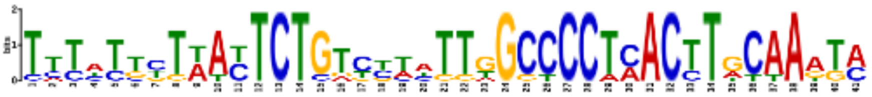	1.8e-016	8	41
18.	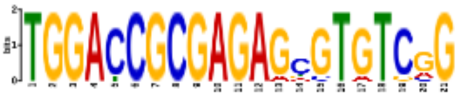	7.1e-016	10	21
19.	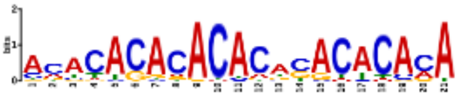	1.5e-016	39	21
20.	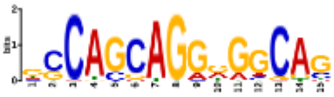	5.4e-016	112	15

HCT-116

		Logo	E-value	Sites	Width
1.	+ -	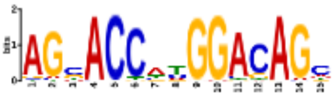	1.2e-4381	1613	15
2.	+ -	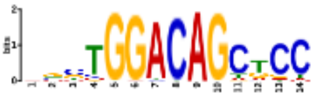	9.0e-688	529	14
3.	+ -	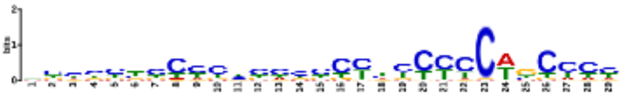	2.0e-200	598	29
4.	+ -	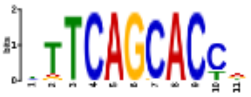	1.3e-169	235	11
5.	+ -	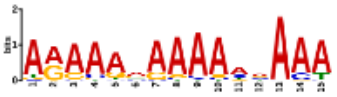	9.0e-058	168	15
6.	+ -	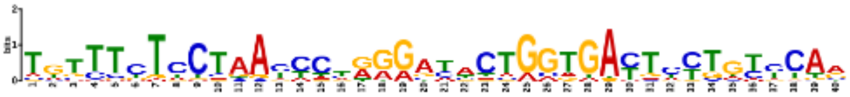	1.8e-028	24	40
7.	+ -	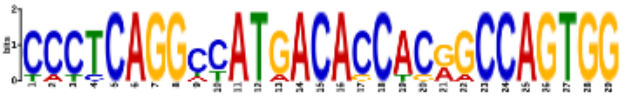	1.5e-027	8	29
8.	+ -	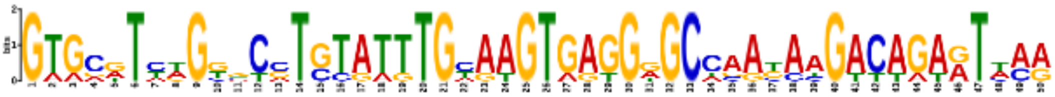	2.1e-010	6	50
9.	+ -	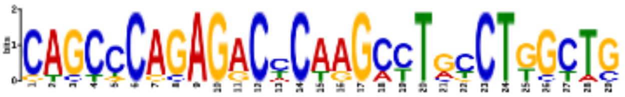	2.2e-017	9	29
10.	+ -	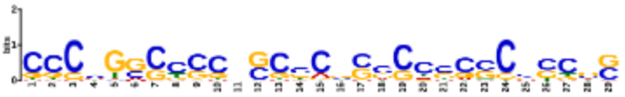	4.0e-055	139	29
11.	+ -	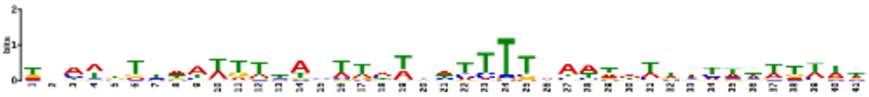	3.2e-014	122	41
12.	+ -	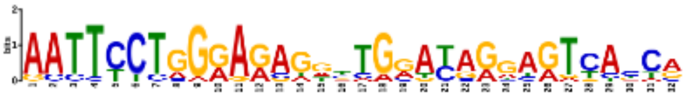	7.5e-011	14	32
13.	+ -	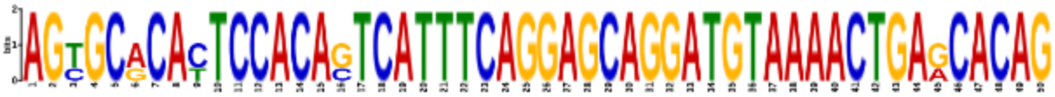	1.7e-004	3	50
14.	+ -	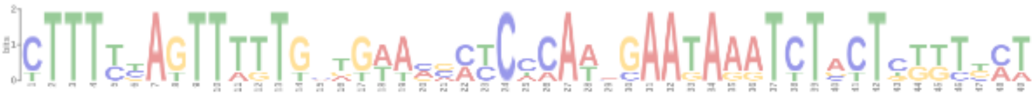	5.2e-002	5	49
15.	+ -	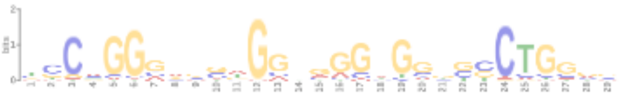	2.6e-001	73	29
16.	+ -	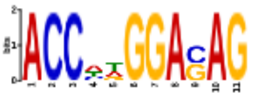	3.7e-006	47	11
17.	+ -	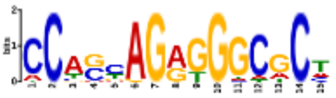	4.2e-007	43	15
18.	+ -	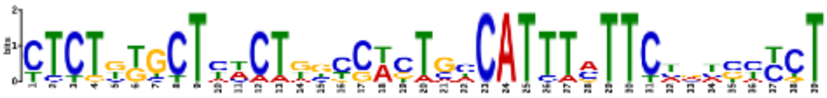	1.7e-003	8	39
19.	+ -	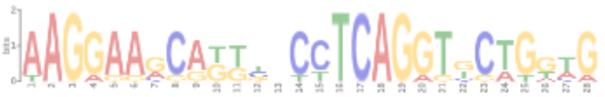	5.1e-001	8	28
20.	+ -	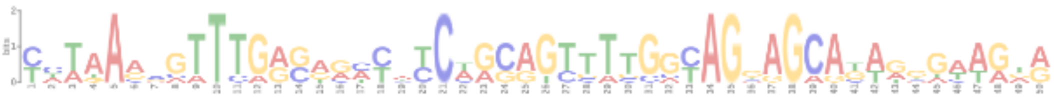	9.7e+000	7	50

HL-60

	Logo	E-value	Sites	Width
1.	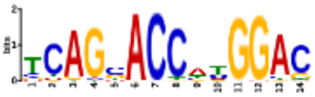	8.2e-3430	1814	14
2.	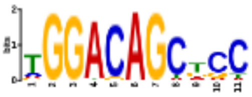	6.6e-841	862	11
3.	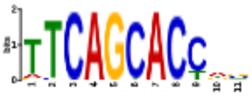	9.1e-323	397	11
4.	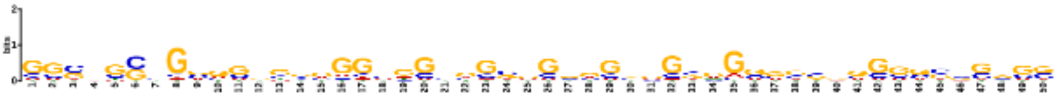	2.7e-455	711	50
5.	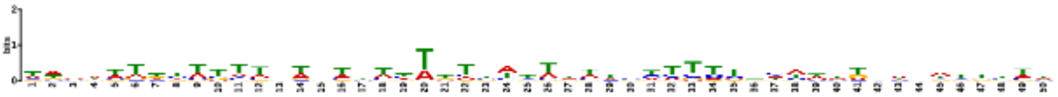	2.9e-307	514	50
6.	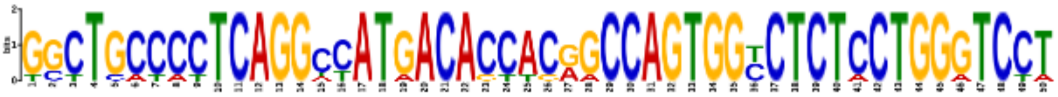	5.1e-075	8	50
7.	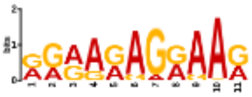	1.9e-068	506	11
8.	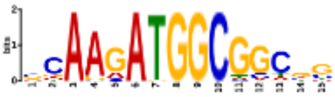	5.0e-062	99	15
9.	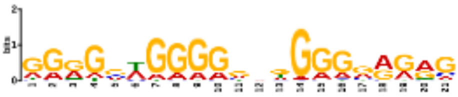	2.1e-029	267	21
10.	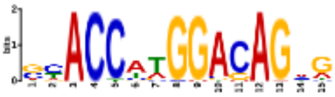	8.4e-021	65	15
11.	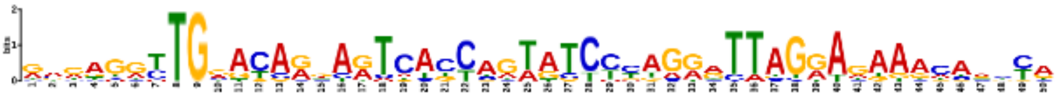	1.7e-018	15	50
12.	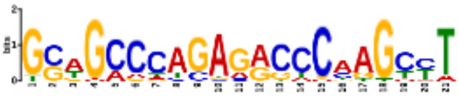	7.3e-009	29	21
13.	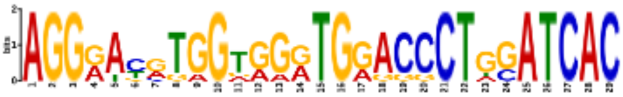	4.3e-007	7	29
14.	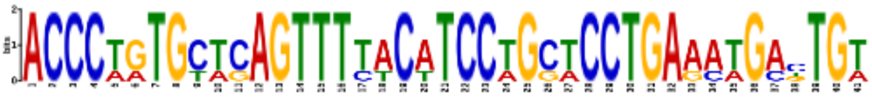	2.5e-004	4	41
15.	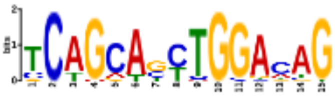	4.4e-004	36	15
16.	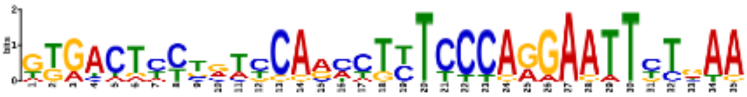	5.5e-004	9	35
17.	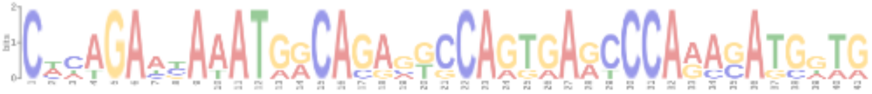	1.3e-001	5	41
18.	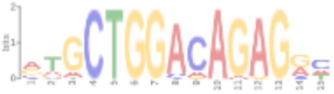	2.2e-001	24	15
19.	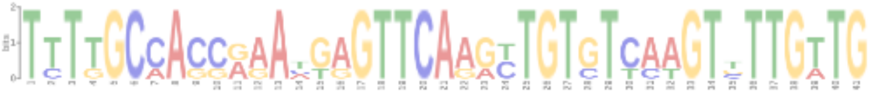	3.6e-001	4	41
20.	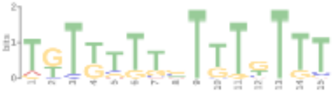	1.2e+000	55	15

HeLaS3

	Logo	E-value	Sites	Width
1.	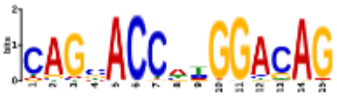	4.5e-5456	2105	15
2.	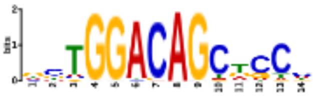	3.3e-1004	837	14
3.	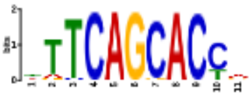	3.9e-312	393	11
4.	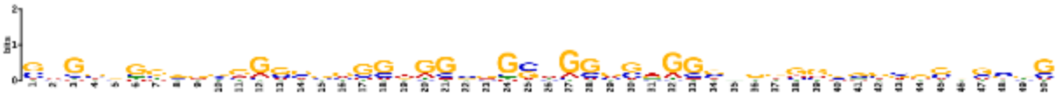	2.3e-299	492	50
5.	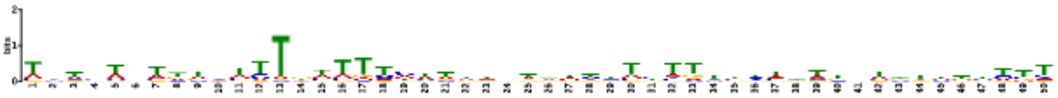	4.4e-134	432	50
6.	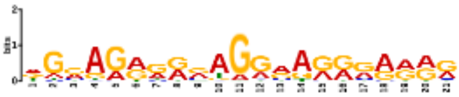	2.1e-093	384	21
7.	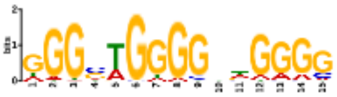	3.6e-022	141	15
8.	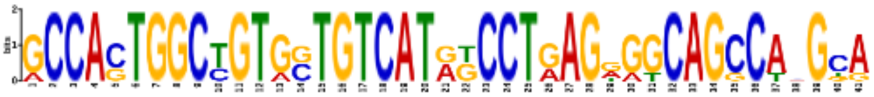	3.7e-021	6	41
9.	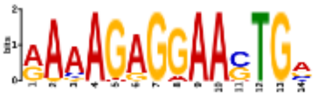	1.8e-016	30	14
10.	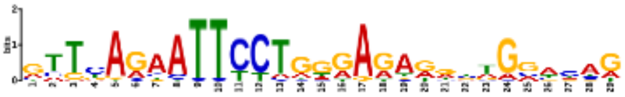	1.3e-012	30	29
11.	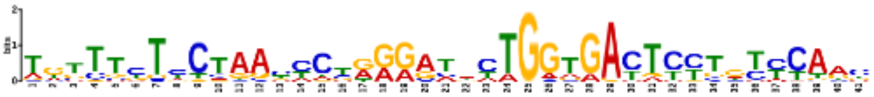	4.8e-016	20	41
12.	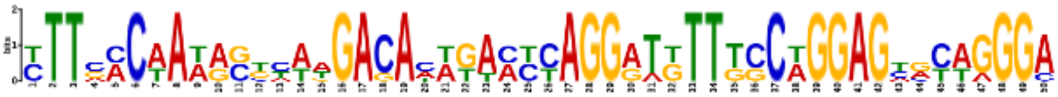	6.9e-013	6	50
13.	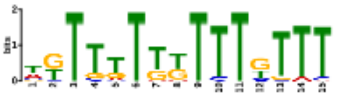	8.9e-009	39	15
14.	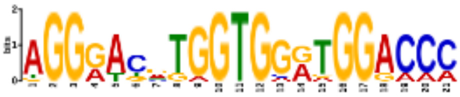	1.5e-005	12	21
15.	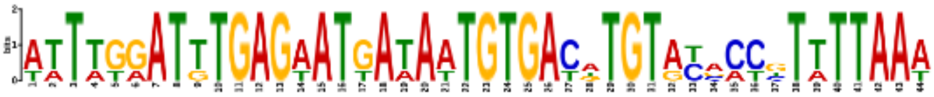	1.1e-004	4	44
16.	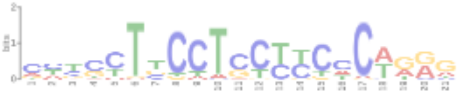	3.6e-001	70	21
17.	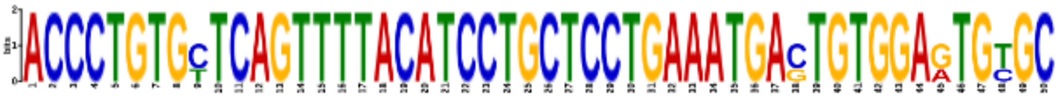	3.1e-004	3	50
18.	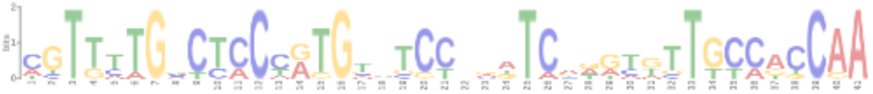	6.9e-001	8	41
19.	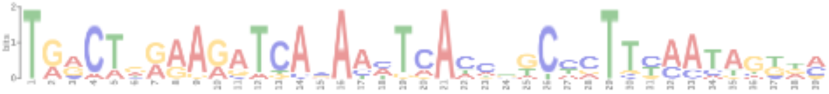	4.5e-001	10	39
20.	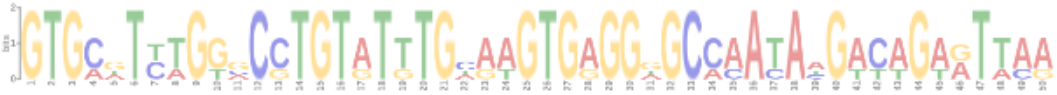	2.0e-001	4	50

HepG2

	Logo	E-value	Sites	Width
1.	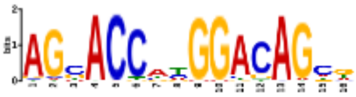	3.8e-4747	1718	16
2.	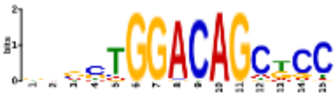	1.2e-771	653	15
3.	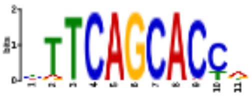	3.9e-185	250	11
4.	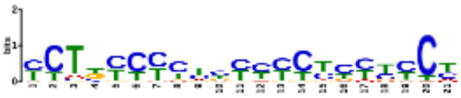	4.2e-177	510	21
5.	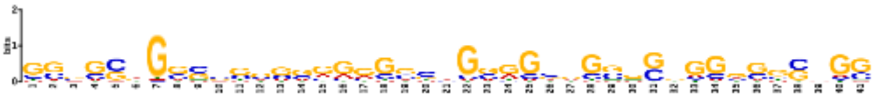	1.1e-178	345	41
6.	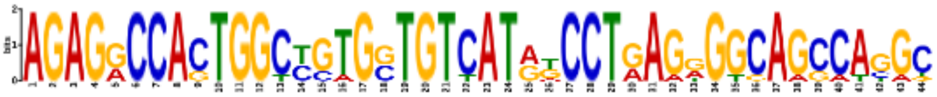	6.3e-065	9	44
7.	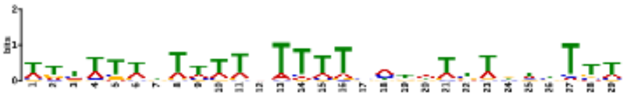	3.5e-049	154	29
8.	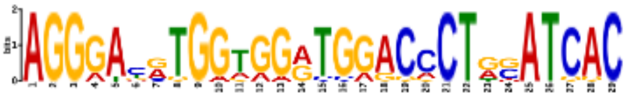	9.5e-029	11	29
9.	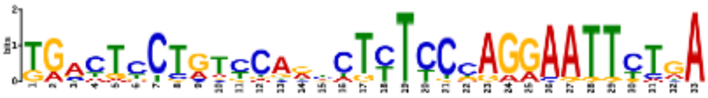	3.6e-017	14	33
10.	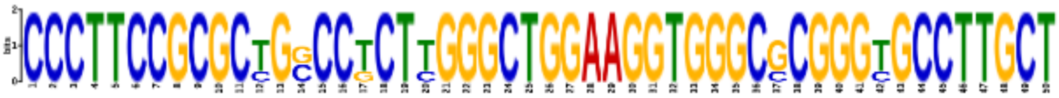	1.1e-016	4	50
11.	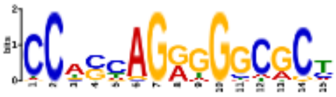	3.1e-011	83	15
12.	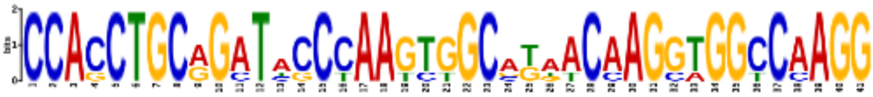	3.3e-011	5	41
13.	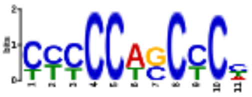	1.5e-010	252	11
14.	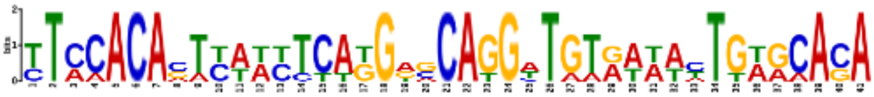	1.7e-007	6	41
15.	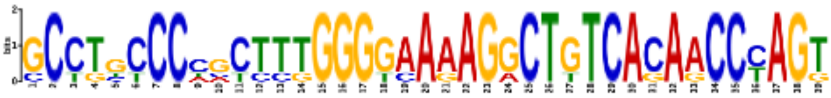	8.6e-007	5	39
16.	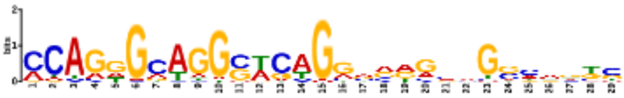	5.7e-006	56	29
17.	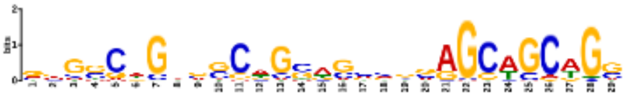	8.3e-006	86	29
18.	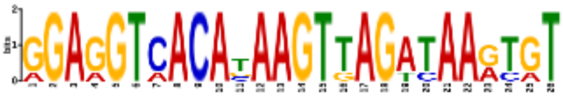	1.5e-002	5	26
19.	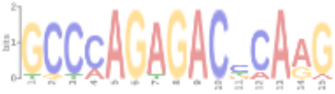	9.9e-001	14	15
20.	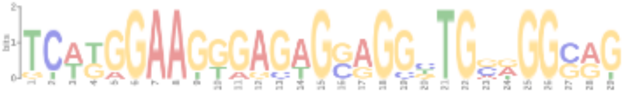	1.6e+000	7	29

K562

	Logo	E-value	Sites	Width
1.	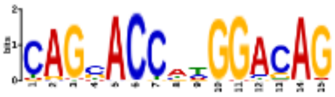	2.6e-4812	1868	15
2.	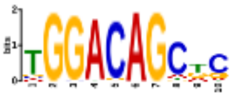	1.9e-708	1056	10
3.	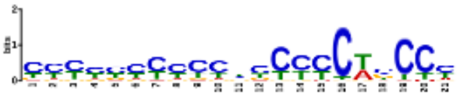	9.2e-606	1489	21
4.	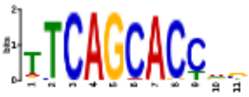	4.5e-345	541	11
5.	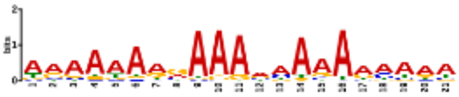	2.0e-112	248	21
6.	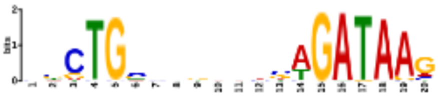	3.5e-310	420	20
7.	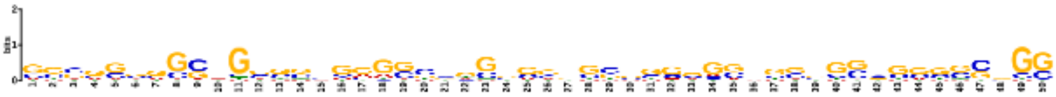	1.0e-203	398	50
8.	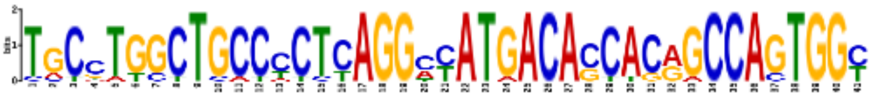	4.8e-088	12	41
9.	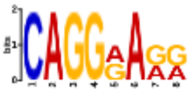	2.2e-040	754	8
10.	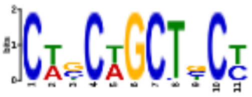	5.7e-033	219	11
11.	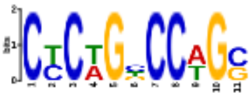	1.6e-033	179	11
12.	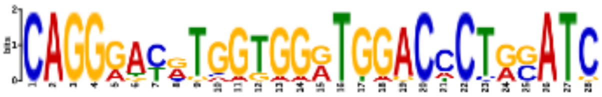	1.7e-027	11	28
13.	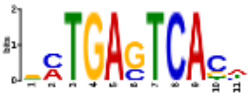	1.6e-021	235	11
14.	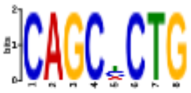	3.9e-036	376	8
15.	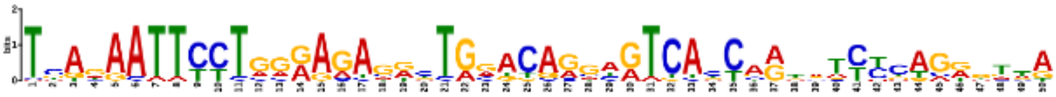	2.2e-015	13	50
16.	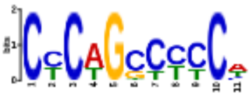	2.6e-013	357	11
17.	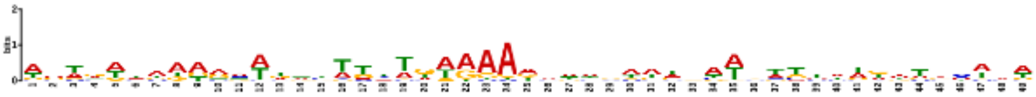	1.5e-023	122	49
18.	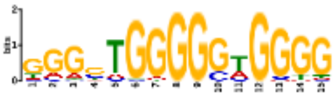	3.0e-011	65	15
19.	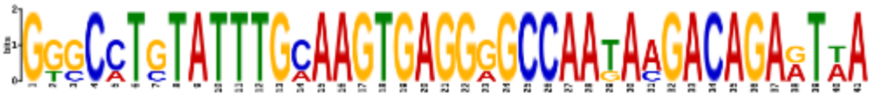	8.0e-008	4	41
20.	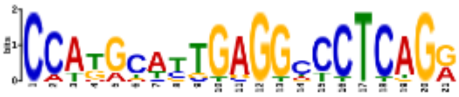	1.9e-005	15	21

MCF-7

		Logo	E-value	Sites	Width
1.	+ -	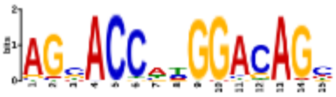	1.4e-5115	2045	15
2.	+ -	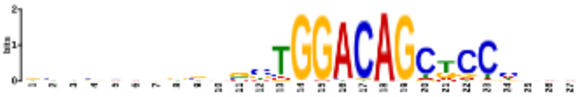	1.0e-962	738	27
3.	+ -	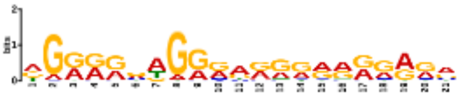	2.4e-218	641	21
4.	+ -	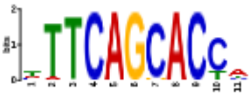	1.1e-070	138	11
5.	+ -	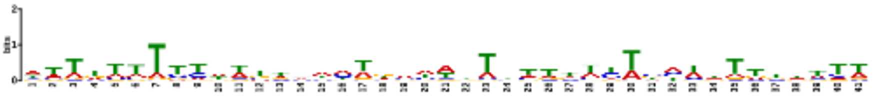	1.9e-081	258	41
6.	+ -	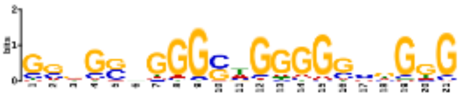	3.9e-081	271	21
7.	+ -	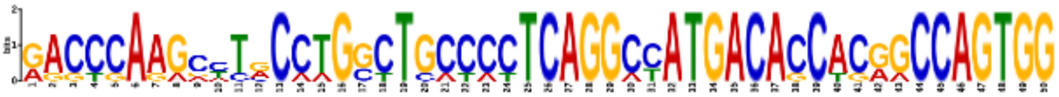	3.4e-029	6	50
8.	+ -	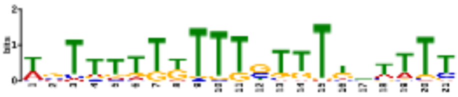	1.3e-022	79	21
9.	+ -	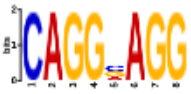	3.3e-020	268	8
10.	+ -	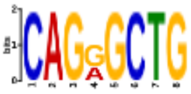	8.2e-014	186	8
11.	+ -	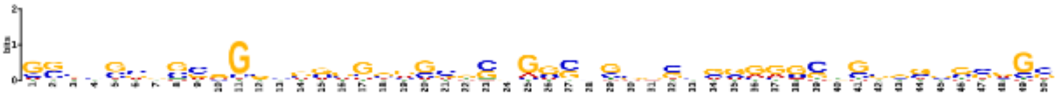	2.4e-078	353	50
12.	+ -	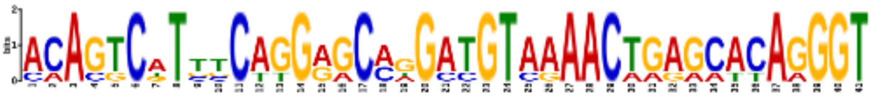	5.9e-005	5	41
13.	+ -	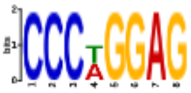	2.4e-004	179	8
14.	+ -	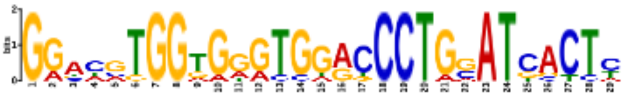	6.9e-005	9	29
15.	+ -	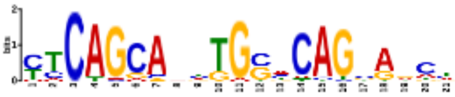	4.4e-002	45	21
16.	+ -	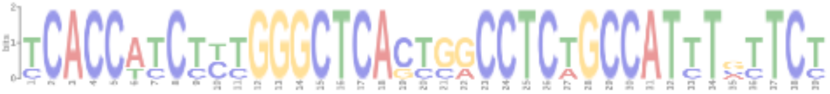	2.5e-001	4	39
17.	+ -	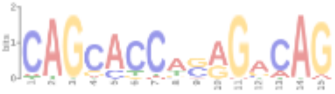	2.8e+000	46	15
18.	+ -	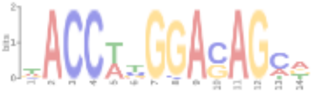	5.3e-002	36	14
19.	+ -	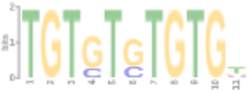	4.1e+002	34	11
20.	+ -	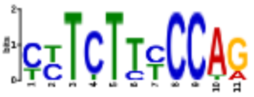	4.0e-002	84	11

Neuron

		Logo	E-value	Sites	Width
1.	+ -	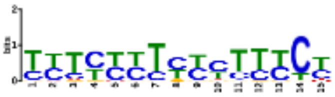	3.7e-624	1649	15
2.	+ -	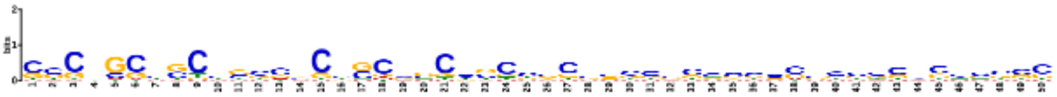	3.4e-1193	1502	50
3.	+ -	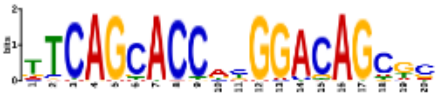	5.4e-397	140	20
4.	+ -	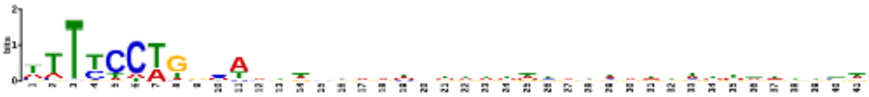	7.5e-312	1184	41
5.	+ -	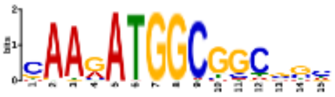	9.1e-219	265	15
6.	+ -	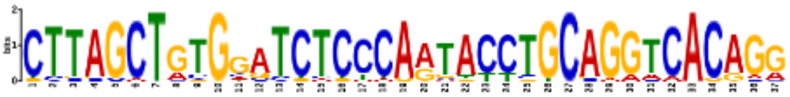	3.7e-187	24	37
7.	+ -	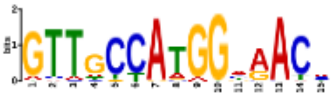	6.0e-151	167	15
8.	+ -	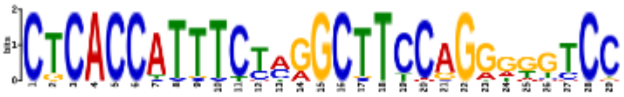	4.3e-149	27	29
9.	+ -	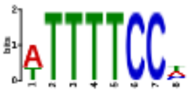	4.1e-126	636	8
10.	+ -	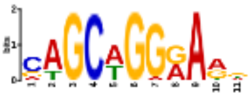	2.0e-039	420	11
11.	+ -	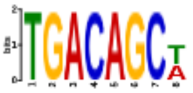	5.8e-043	273	8
12.	+ -	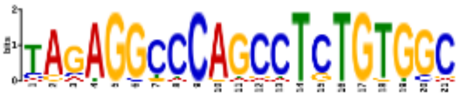	3.7e-038	18	21
13.	+ -	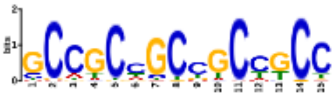	1.0e-031	179	15
14.	+ -	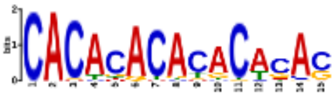	2.1e-024	61	15
15.	+ -	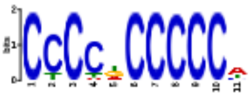	5.2e-041	151	11
16.	+ -	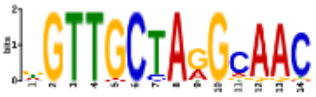	3.1e-017	32	14
17.	+ -	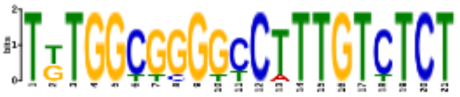	4.4e-014	9	21
18.	+ -	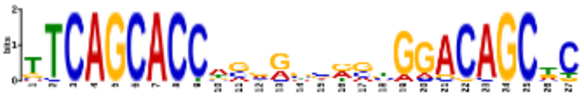	5.9e-017	16	27
19.	+ -	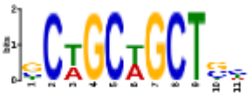	4.9e-021	252	11
20.	+ -	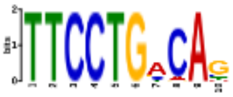	4.2e-014	113	10

PANC-1

	Logo	E-value	Sites	Width
1.	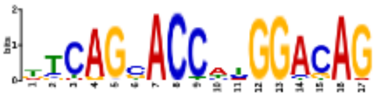	7.4e-5599	1719	17
2.	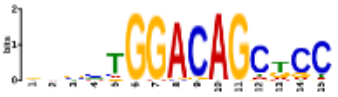	1.7e-861	783	15
3.	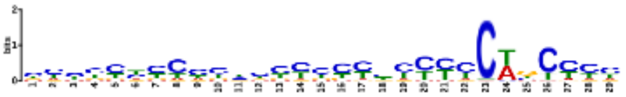	1.1e-255	631	29
4.	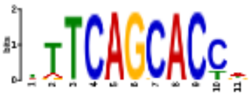	1.9e-195	268	11
5.	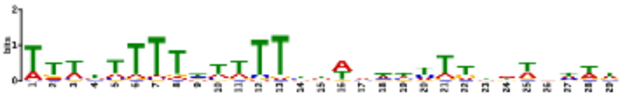	4.4e-097	264	29
6.	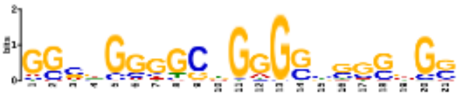	1.6e-037	210	21
7.	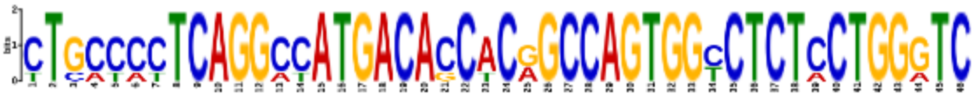	6.9e-023	5	46
8.	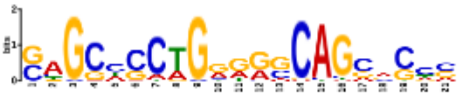	7.9e-009	54	21
9.	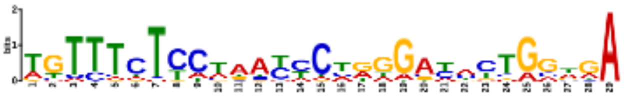	2.0e-008	30	29
10.	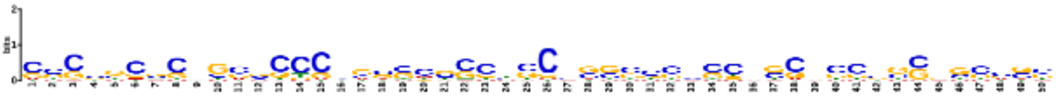	2.7e-086	210	50
11.	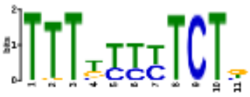	9.6e-005	98	11
12.	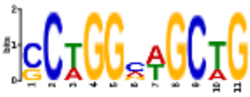	1.7e-005	84	11
13.	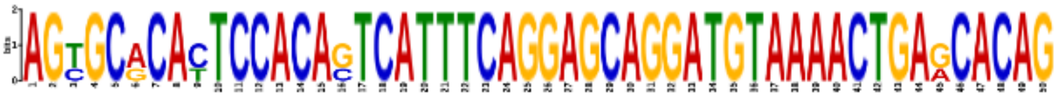	1.4e-004	3	50
14.	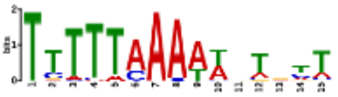	3.2e-003	60	15
15.	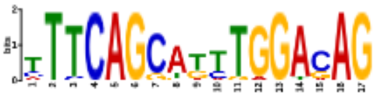	4.4e-003	13	17
16.	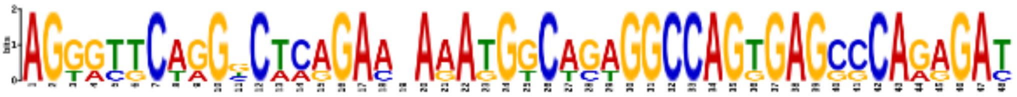	1.1e-002	4	48
17.	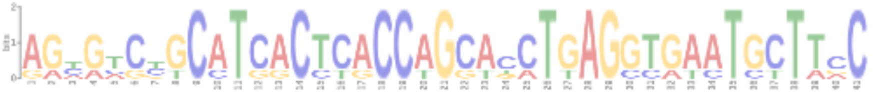	3.9e-001	5	41
18.	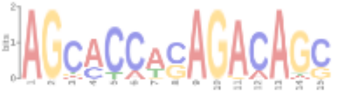	3.0e+001	18	15
19.	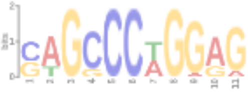	1.7e+002	74	11
20.	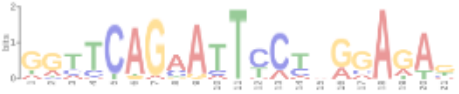	1.7e+003	22	21

PFSK-1

		Logo	E-value	Sites	Width
1.	+ -	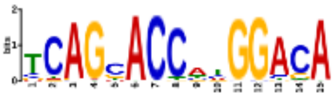	1.6e-4265	1607	15
2.	+ -	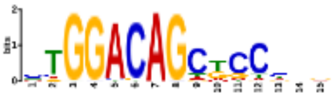	7.1e-1109	977	15
3.	+ -	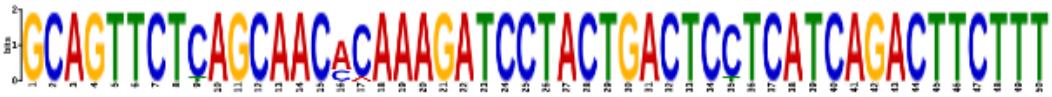	7.4e-271	15	50
4.	+ -	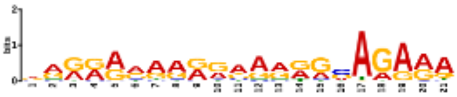	4.7e-232	579	21
5.	+ -	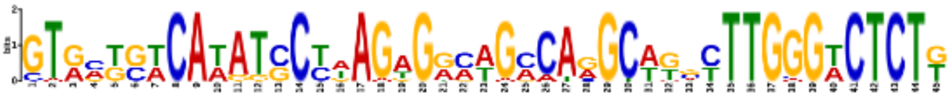	2.9e-217	23	45
6.	+ -	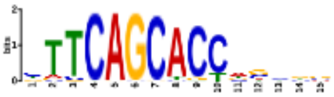	2.4e-198	317	15
7.	+ -	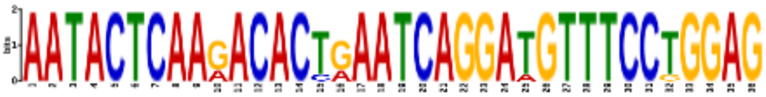	2.0e-166	15	36
8.	+ -	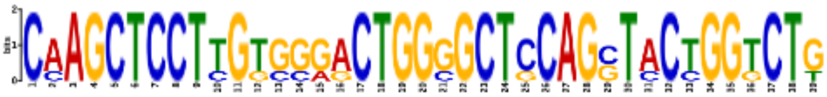	3.6e-113	13	39
9.	+ -	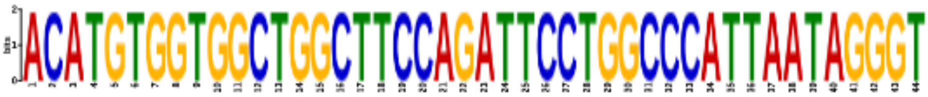	7.2e-132	10	44
10.	+ -	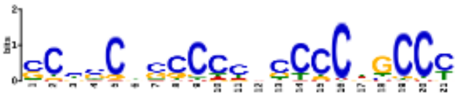	1.5e-088	391	21
11.	+ -	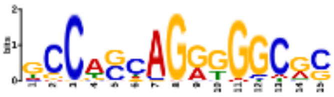	9.5e-075	205	15
12.	+ -	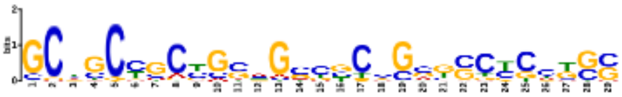	2.4e-081	138	29
13.	+ -	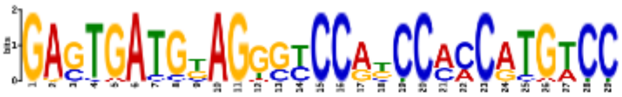	4.8e-071	17	29
14.	+ -	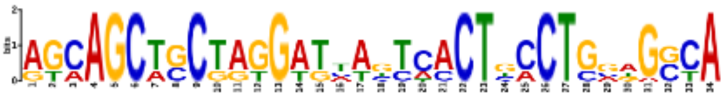	4.8e-065	16	34
15.	+ -	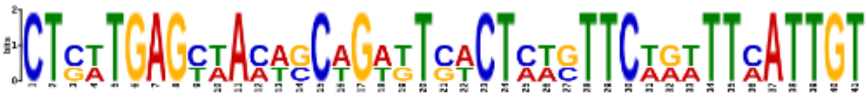	2.0e-109	13	41
16.	+ -	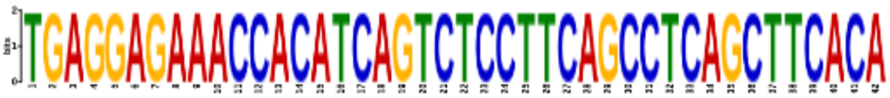	2.2e-087	8	42
17.	+ -	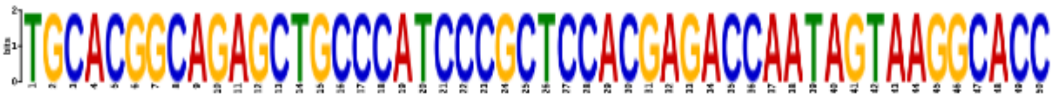	1.3e-085	7	50
18.	+ -	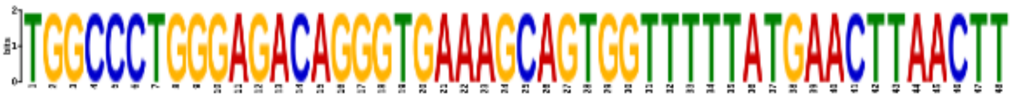	1.1e-044	5	48
19.	+ -	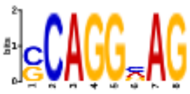	4.0e-043	520	8
20.	+ -	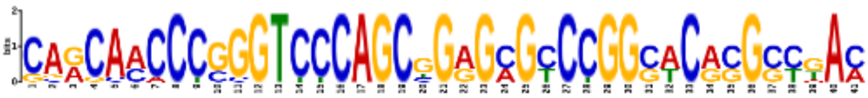	7.7e-063	11	41

SK-N-SH

	Logo	E-value	Sites	Width
1.	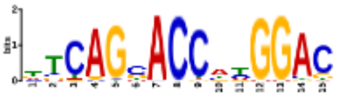	1.0e-3098	1584	15
2.	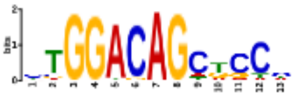	1.7e-1217	1138	13
3.	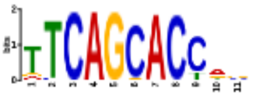	1.6e-298	409	11
4.	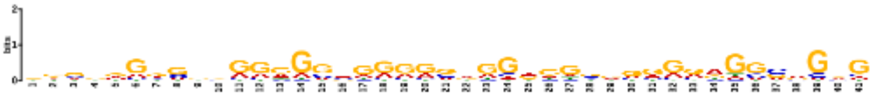	5.1e-254	792	41
5.	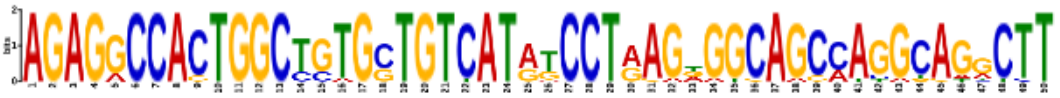	9.0e-247	18	50
6.	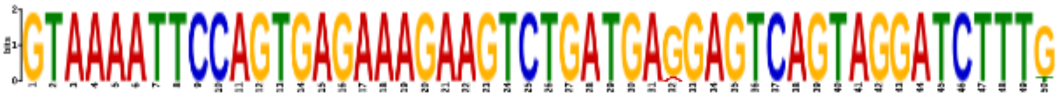	6.9e-228	13	50
7.	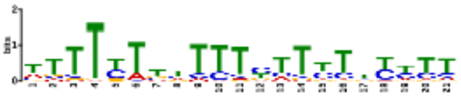	4.3e-162	281	21
8.	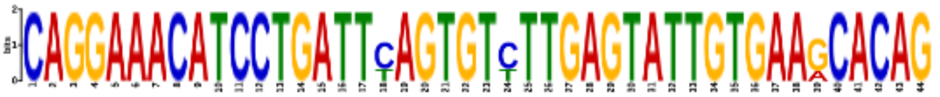	4.4e-184	13	44
9.	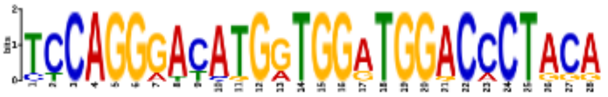	4.5e-064	13	28
10.	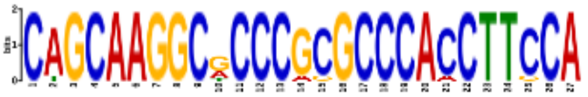	8.5e-046	10	27
11.	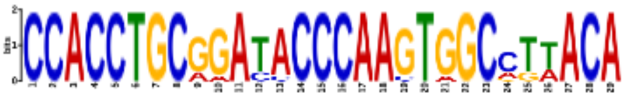	6.3e-049	10	29
12.	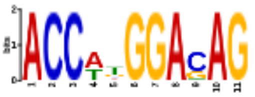	7.0e-042	89	11
13.	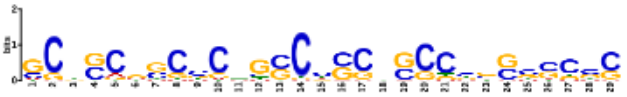	9.0e-050	235	29
14.	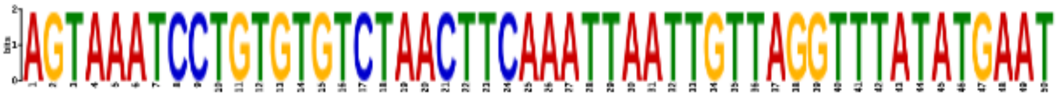	1.6e-029	4	50
15.	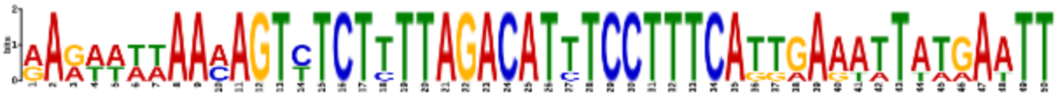	2.6e-028	5	50
16.	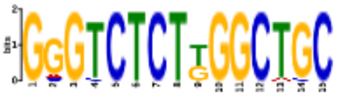	1.3e-024	19	15
17.	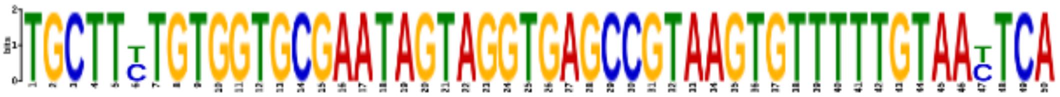	2.5e-024	4	50
18.	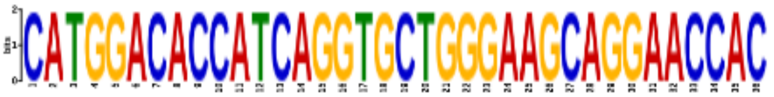	6.6e-024	5	36
19.	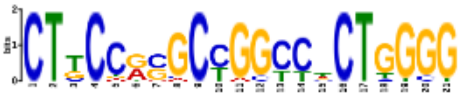	5.6e-023	19	21
20.	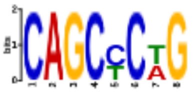	9.9e-017	347	8

Tcell

		Logo	E-value	Sites	Width
1.	+ -	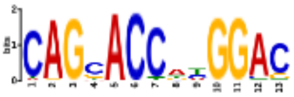	5.3e-1863	1176	13
2.	+ -	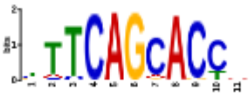	1.7e-453	650	11
3.	+ -	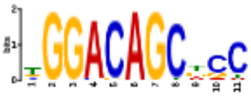	6.3e-368	631	11
4.	+ -	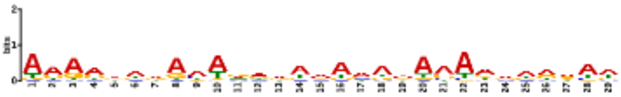	1.3e-364	675	29
5.	+ -	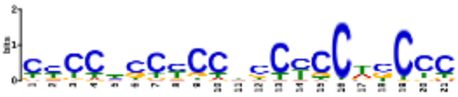	1.4e-156	881	21
6.	+ -	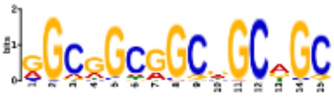	8.3e-088	269	15
7.	+ -	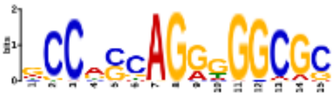	1.6e-094	203	15
8.	+ -	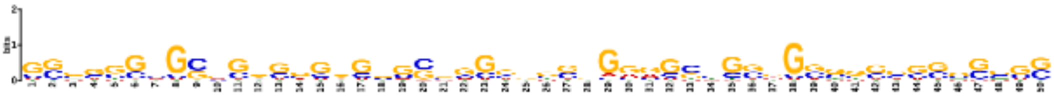	5.4e-186	749	50
9.	+ -	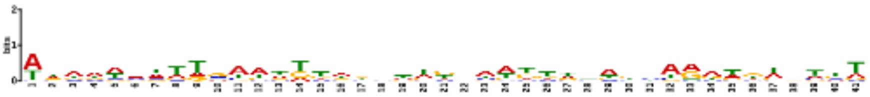	4.3e-075	205	41
10.	+ -	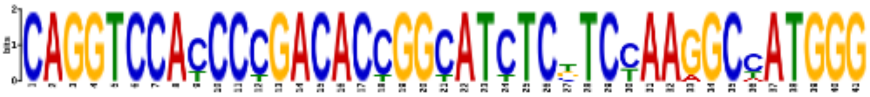	1.8e-072	9	41
11.	+ -	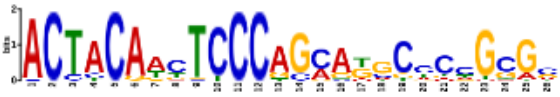	2.1e-065	33	26
12.	+ -	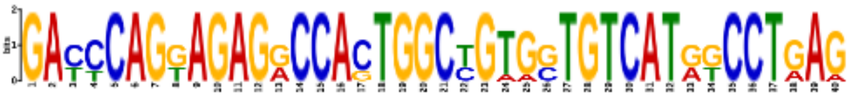	2.7e-051	8	40
13.	+ -	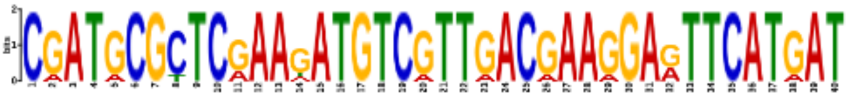	5.8e-067	8	40
14.	+ -	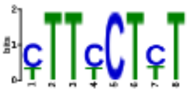	6.8e-033	368	8
15.	+ -	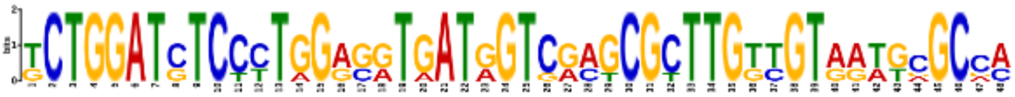	2.0e-027	6	48
16.	+ -	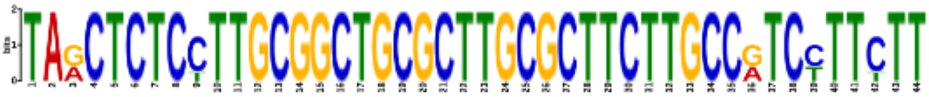	2.1e-031	5	44
17.	+ -	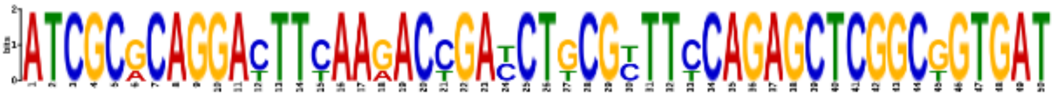	2.1e-017	4	50
18.	+ -	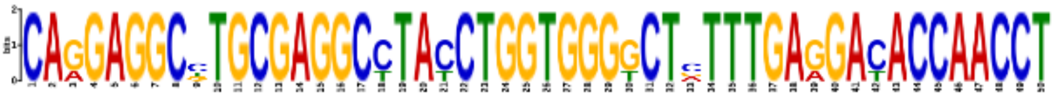	2.2e-017	4	50
19.	+ -	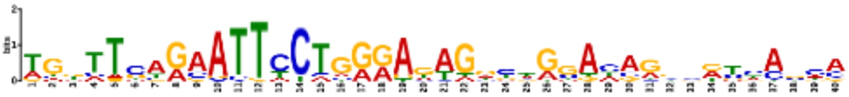	8.1e-013	20	40
20.	+ -	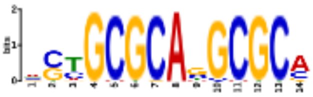	3.4e-016	72	14

U87

		Logo	E-value	Sites	Width
1.	+ -	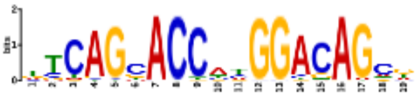	4.6e-5018	1449	19
2.	+ -	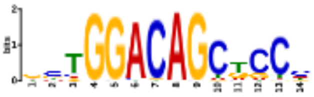	4.4e-533	496	14
3.	+ -	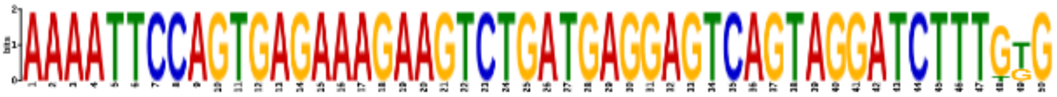	8.1e-233	13	50
4.	+ -	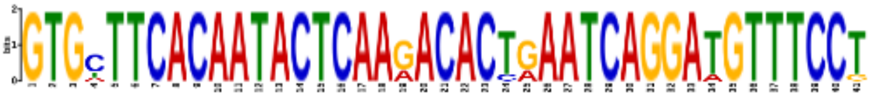	2.6e-199	15	41
5.	+ -	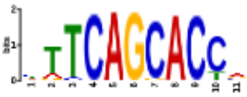	1.5e-167	252	11
6.	+ -	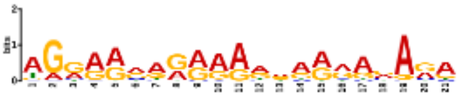	3.5e-144	308	21
7.	+ -	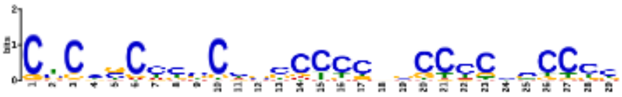	1.7e-101	357	29
8.	+ -	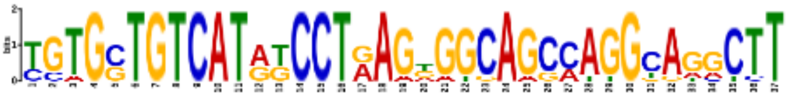	9.1e-052	10	37
9.	+ -	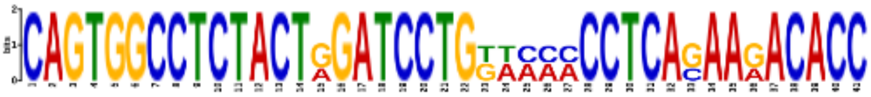	5.5e-036	6	41
10.	+ -	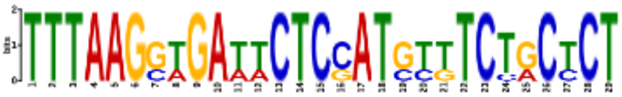	4.1e-021	7	29
11.	+ -	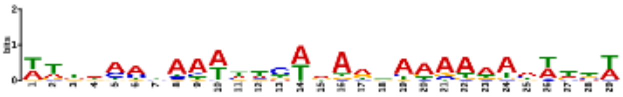	1.1e-021	139	29
12.	+ -	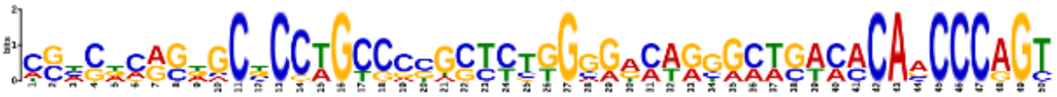	4.3e-021	9	50
13.	+ -	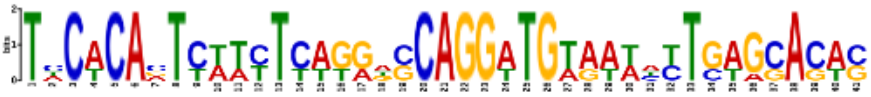	2.8e-018	7	41
14.	+ -	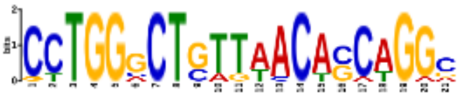	5.5e-018	13	21
15.	+ -	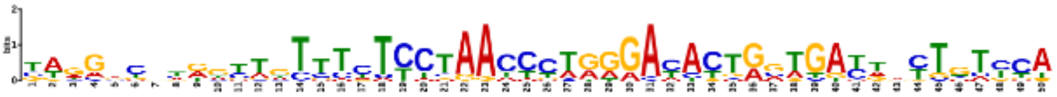	5.0e-012	15	50
16.	+ -	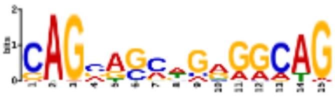	3.3e-006	124	15
17.	+ -	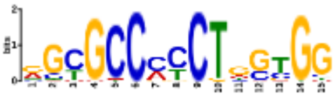	5.4e-022	85	15
18.	+ -	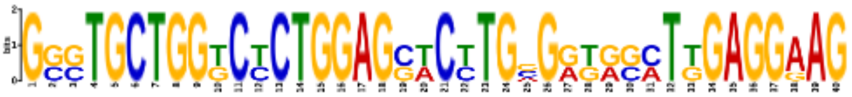	3.4e-009	5	40
19.	+ -	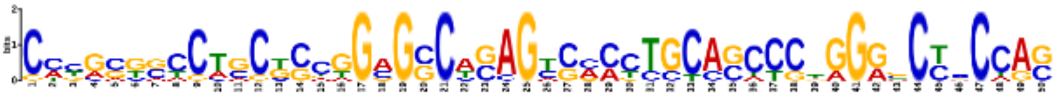	1.1e-007	9	50
20.	+ -	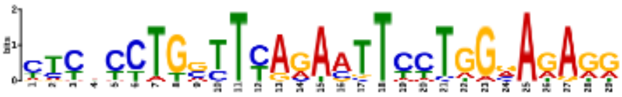	5.7e-005	12	29

(B) MEME output on dm3 (fly)

CTCF

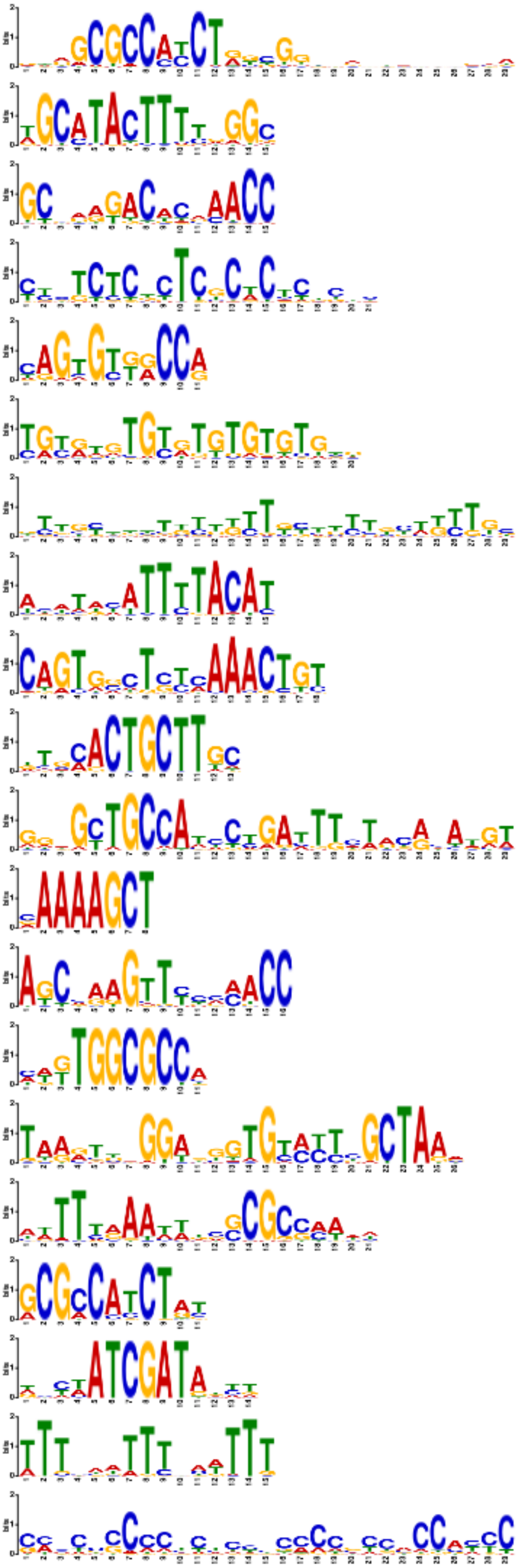

		Logo	E-value	Sites	Width
1.	+ -		2.0e-2298	1012	29
2.	+ -		6.5e-265	176	15
3.	+ -		3.2e-278	319	15
4.	+ -		2.2e-138	169	21
5.	+ -		1.9e-134	287	11
6.	+ -		2.8e-096	109	20
7.	+ -		4.7e-121	284	29
8.	+ -		1.2e-059	177	15
9.	+ -		7.3e-052	48	18
10.	+ -		3.7e-043	85	13
11.	+ -		9.9e-038	29	29
12.	+ -		1.9e-031	214	8
13.	+ -		4.8e-033	68	16
14.	+ -		7.5e-026	80	11
15.	+ -		6.6e-018	23	26
16.	+ -		5.1e-020	72	21
17.	+ -		4.0e-016	86	11
18.	+ -		4.0e-044	168	14
19.	+ -		1.5e-016	216	15
20.	+ -		1.6e-012	55	29

SU(HW)

		Logo	E-value	Sites	Width
1.	+ -	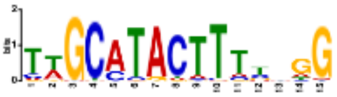	4.4e-3832	1978	15
2.	+ -	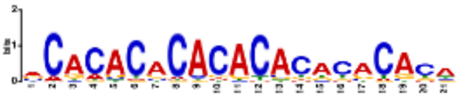	6.2e-796	500	21
3.	+ -	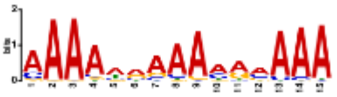	1.9e-262	602	15
4.	+ -	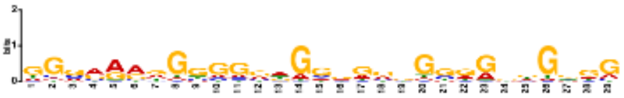	6.7e-271	640	29
5.	+ -	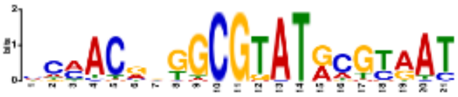	1.7e-146	87	21
6.	+ -	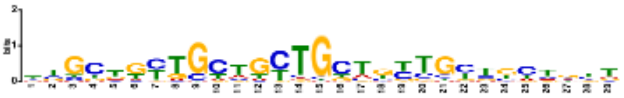	2.1e-136	248	29
7.	+ -	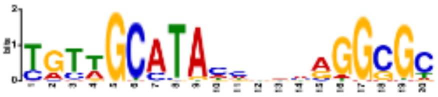	5.9e-118	108	20
8.	+ -	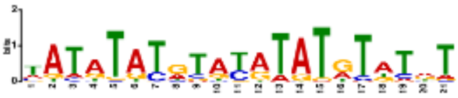	1.0e-064	138	21
9.	+ -	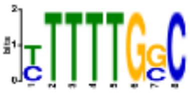	1.9e-034	347	8
10.	+ -	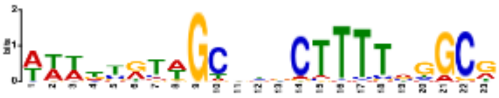	4.4e-042	98	23
11.	+ -	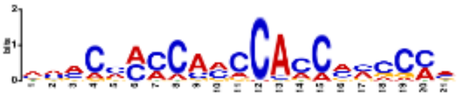	2.6e-034	130	21
12.	+ -	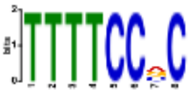	5.2e-025	267	8
13.	+ -	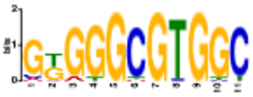	2.2e-021	56	11
14.	+ -	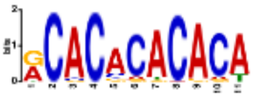	1.1e-021	103	11
15.	+ -	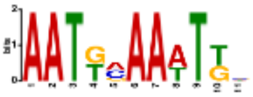	5.6e-023	231	11
16.	+ -	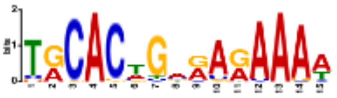	2.6e-021	71	15
17.	+ -	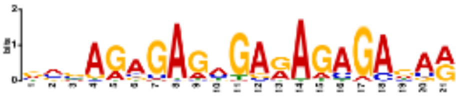	3.6e-016	59	21
18.	+ -	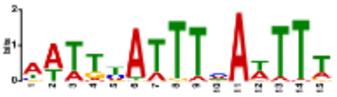	8.0e-017	193	15
19.	+ -	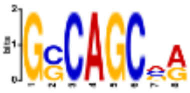	1.4e-009	504	8
20.	+ -	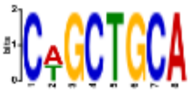	2.1e-003	137	8

Pita

		Logo	E-value	Sites	Width
1.	+ -	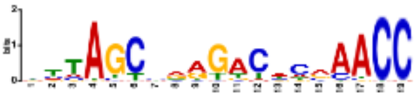	1.2e-1183	759	19
2.	+ -	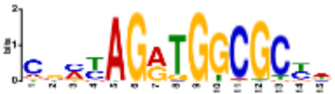	2.7e-339	224	15
3.	+ -	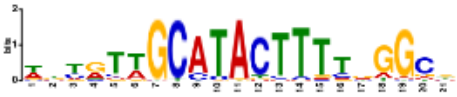	8.6e-323	179	21
4.	+ -	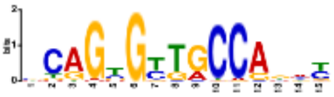	1.3e-085	173	15
5.	+ -	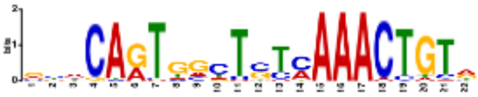	1.6e-069	46	22
6.	+ -	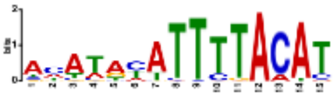	1.3e-066	197	15
7.	+ -	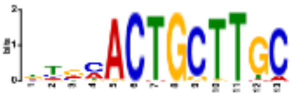	1.3e-041	71	13
8.	+ -	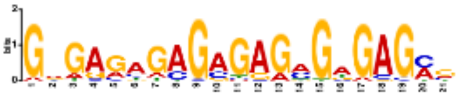	9.0e-034	31	21
9.	+ -	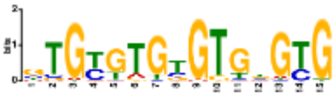	2.3e-031	65	15
10.	+ -	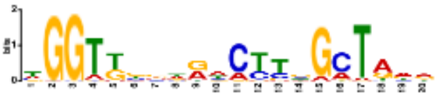	7.9e-086	181	20
11.	+ -	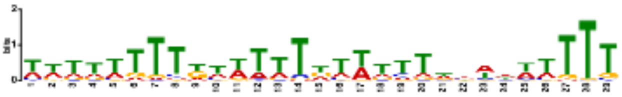	1.9e-029	200	29
12.	+ -	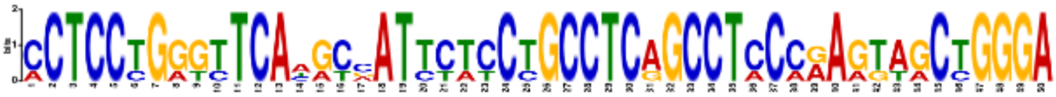	5.2e-012	4	50
13.	+ -	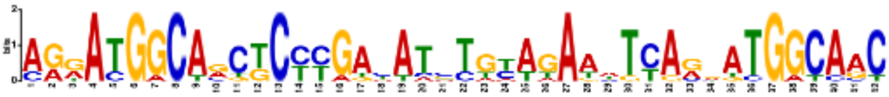	4.7e-019	9	42
14.	+ -	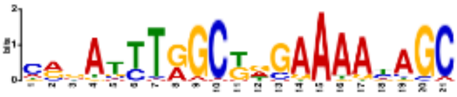	4.9e-014	27	21
15.	+ -	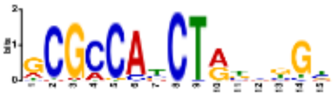	4.6e-014	64	15
16.	+ -	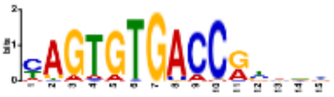	5.5e-009	65	15
17.	+ -	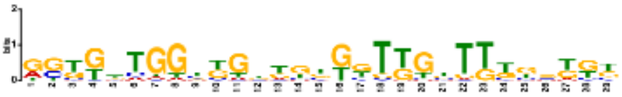	1.3e-005	50	29
18.	+ -	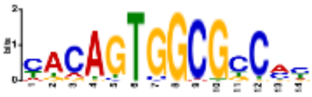	5.6e-004	31	14
19.	+ -	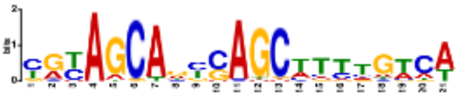	5.7e-004	27	21
20.	+ -	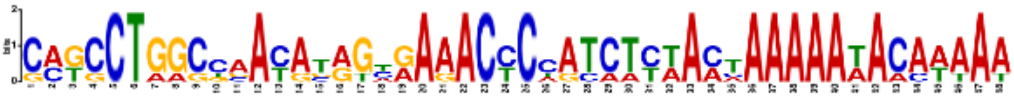	3.9e-002	5	48

(C) DREME output on K562 cell-line

FOSL1

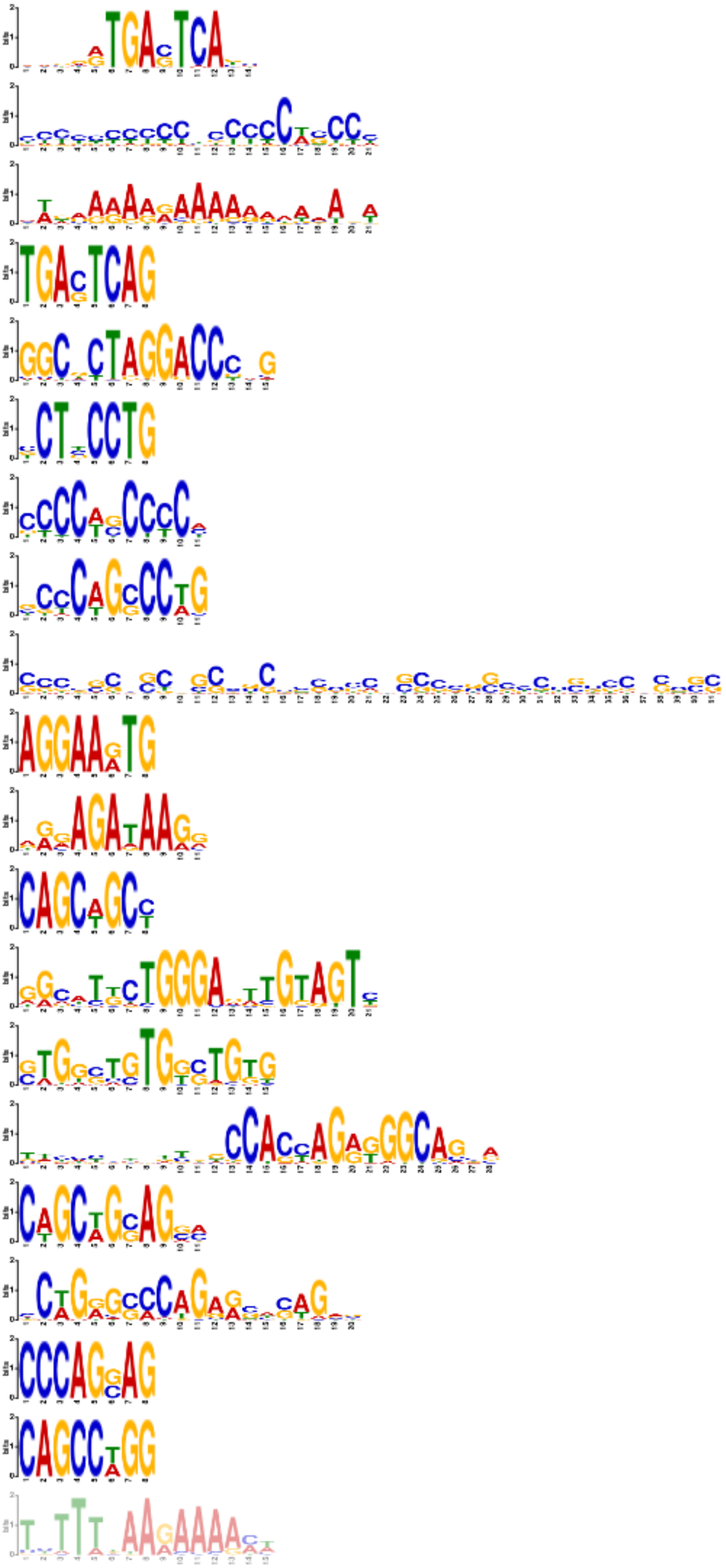

	Logo	E-value	Sites	Width
1.		2.0e-5453	3914	14
2.		4.2e-641	1416	21
3.		8.4e-113	263	21
4.		3.1e-095	314	8
5.		5.3e-088	94	15
6.		1.1e-064	925	8
7.		4.8e-040	372	11
8.		4.2e-049	311	11
9.		4.8e-174	359	41
10.		4.4e-037	238	8
11.		1.3e-026	267	11
12.		9.0e-024	441	8
13.		1.1e-016	24	21
14.		3.1e-008	89	15
15.		4.4e-013	39	28
16.		1.0e-005	135	11
17.		9.9e-009	73	20
18.		4.9e-003	237	8
19.		1.6e-004	239	8
20.		4.2e+002	39	15

FOXA1

		Logo	E-value	Sites	Width
1.	+ -	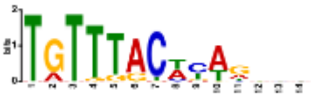	9.2e-330	393	14
2.	+ -	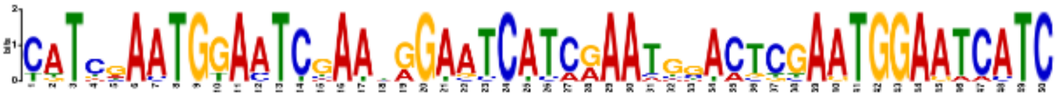	2.6e-083	10	50
3.	+ -	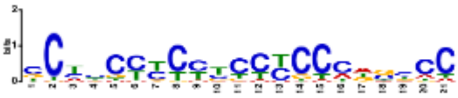	3.2e-035	94	21
4.	+ -	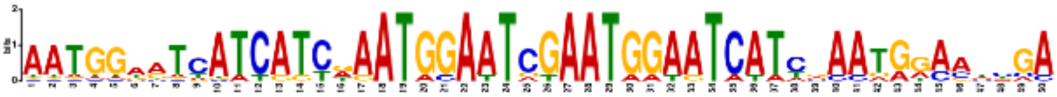	9.3e-028	8	50
5.	+ -	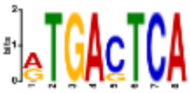	3.7e-008	71	8
6.	+ -	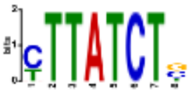	1.8e-011	111	8
7.	+ -	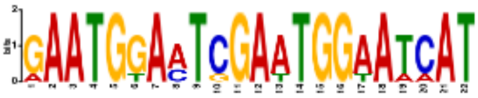	5.6e-006	6	22
8.	+ -	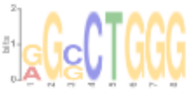	6.8e-002	41	8
9.	+ -	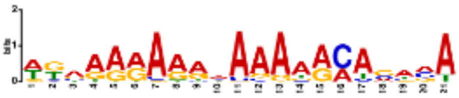	7.9e-006	67	21
10.	+ -	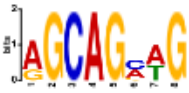	8.6e-004	96	8
11.	+ -	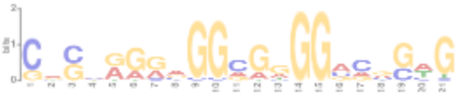	8.6e-001	21	21
12.	+ -	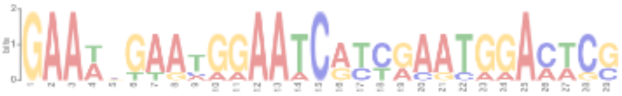	9.8e+000	6	29
13.	+ -	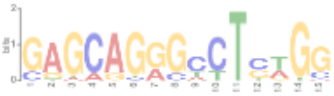	5.2e+001	19	15
14.	+ -	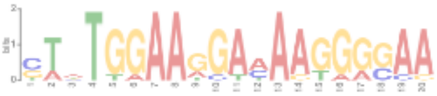	1.9e+002	9	20
15.	+ -	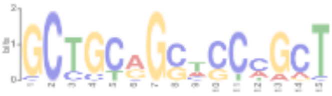	5.9e+003	14	15
16.	+ -	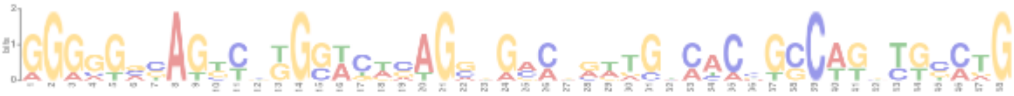	5.7e+003	6	48
17.	+ -	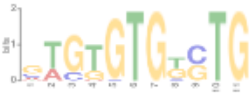	7.9e+004	33	11
18.	+ -	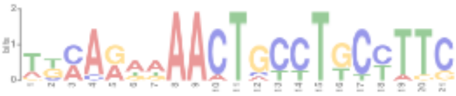	8.3e+004	9	21
19.	+ -	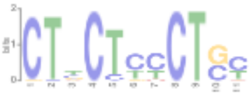	1.1e+005	41	11
20.	+ -	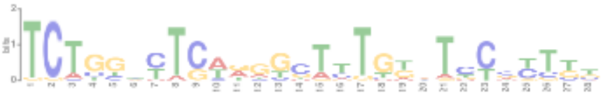	8.2e+002	21	28

GATA1

		Logo	E-value	Sites	Width
1.	+ -	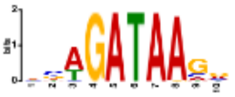	7.1e-1320	1710	10
2.	+ -	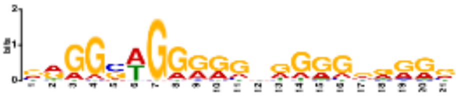	1.3e-257	651	21
3.	+ -	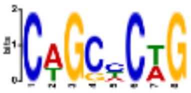	2.7e-107	945	8
4.	+ -	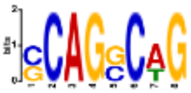	2.4e-069	528	8
5.	+ -	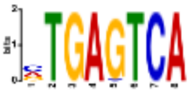	1.7e-036	206	8
6.	+ -	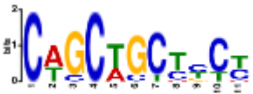	3.7e-024	355	11
7.	+ -	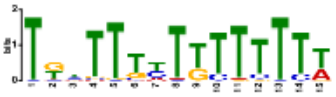	2.6e-023	48	15
8.	+ -	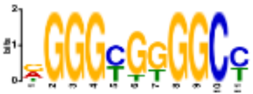	2.2e-012	92	11
9.	+ -	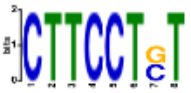	4.1e-008	119	8
10.	+ -	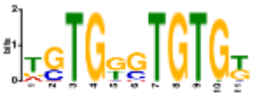	4.6e-006	104	11
11.	+ -	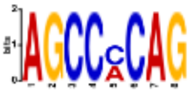	4.5e-011	148	8
12.	+ -	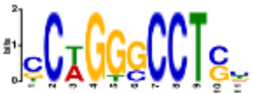	1.2e-005	135	11
13.	+ -	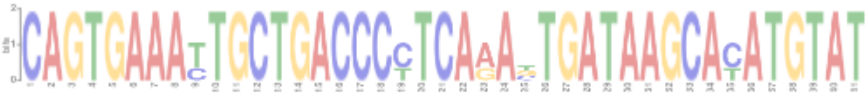	5.3e-002	3	41
14.	+ -	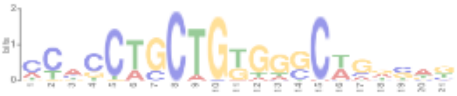	2.6e-001	45	21
15.	+ -	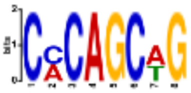	1.9e-007	240	8
16.	+ -	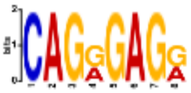	7.0e-016	238	8
17.	+ -	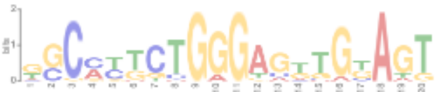	7.3e+000	23	20
18.	+ -	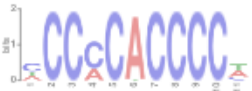	3.1e+001	47	11
19.	+ -	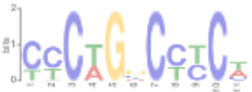	9.0e+001	260	11
20.	+ -	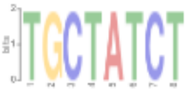	6.2e+004	51	8

GATA2

	Logo	E-value	Sites	Width
1.	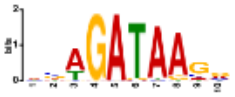	4.0e-1636	3175	10
2.	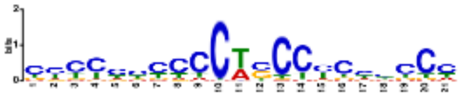	3.6e-448	1083	21
3.	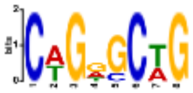	2.3e-141	1227	8
4.	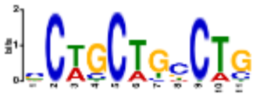	5.1e-041	636	11
5.	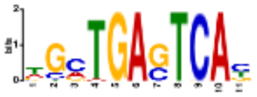	3.5e-052	207	11
6.	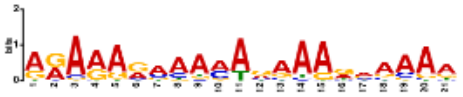	1.8e-039	184	21
7.	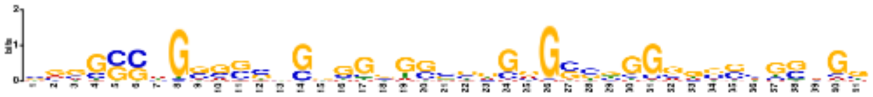	5.3e-030	97	41
8.	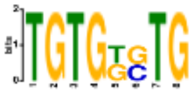	3.1e-023	302	8
9.	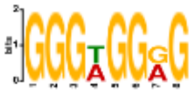	1.3e-032	360	8
10.	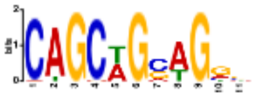	2.9e-015	260	11
11.	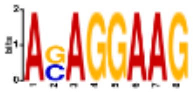	1.2e-012	164	8
12.	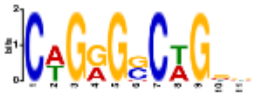	2.1e-051	407	11
13.	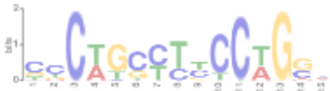	1.0e+001	174	15
14.	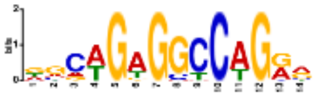	2.5e-003	116	14
15.	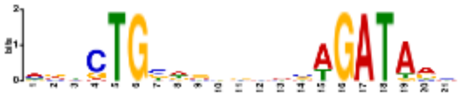	8.2e-028	217	21
16.	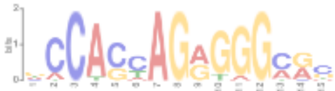	2.3e+001	46	15
17.	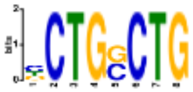	2.6e-003	406	8
18.	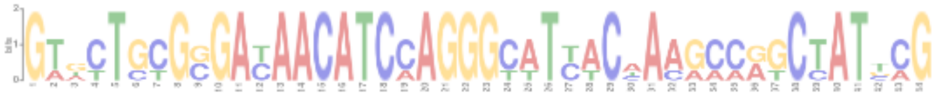	1.7e+002	4	44
19.	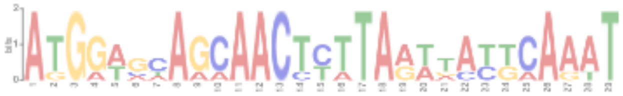	3.2e+006	5	29
20.	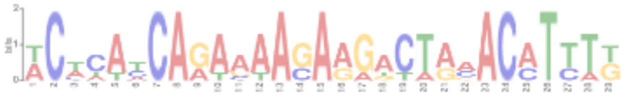	2.5e+006	6	29

IRF2

		Logo	E-value	Sites	Width
1.	+ -	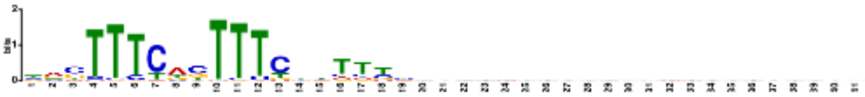	2.0e-3576	2425	41
2.	+ -	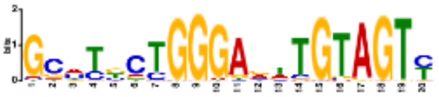	3.4e-1584	562	20
3.	+ -	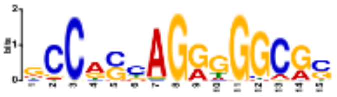	3.8e-615	958	15
4.	+ -	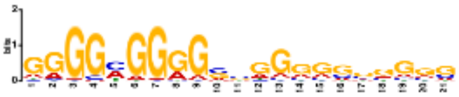	1.0e-545	1484	21
5.	+ -	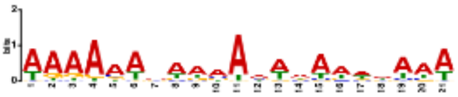	8.7e-201	550	21
6.	+ -	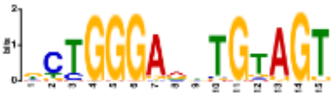	3.9e-190	211	15
7.	+ -	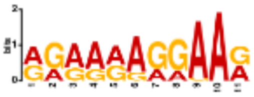	5.0e-122	705	11
8.	+ -	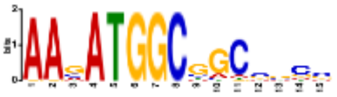	1.0e-091	211	15
9.	+ -	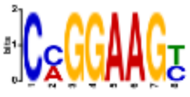	9.7e-087	754	8
10.	+ -	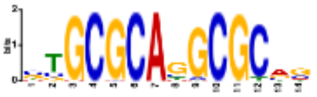	2.1e-081	164	14
11.	+ -	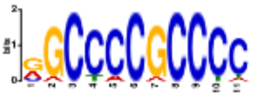	1.7e-055	240	11
12.	+ -	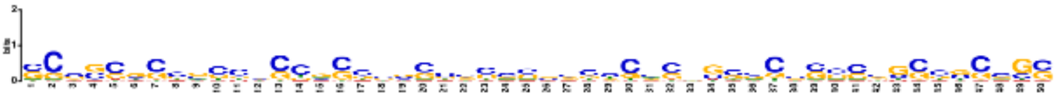	7.6e-321	1097	50
13.	+ -	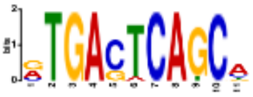	2.4e-024	120	11
14.	+ -	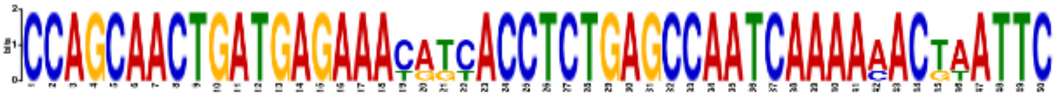	6.8e-022	4	50
15.	+ -	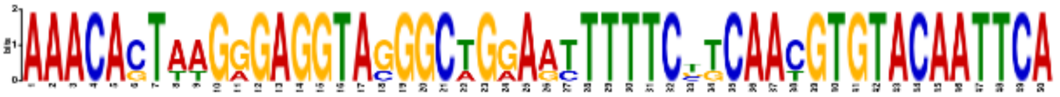	2.4e-017	4	50
16.	+ -	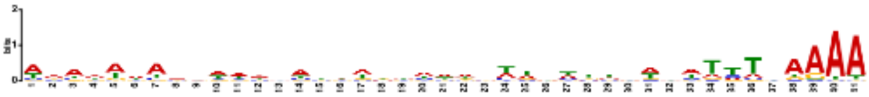	1.9e-118	357	41
17.	+ -	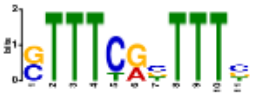	3.2e-036	158	11
18.	+ -	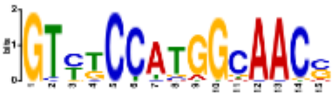	2.7e-010	45	15
19.	+ -	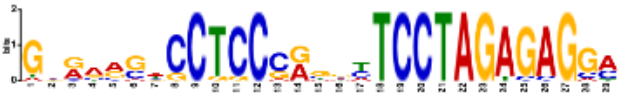	4.3e-007	12	29
20.	+ -	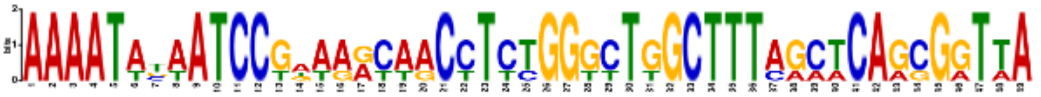	6.7e-005	4	49

JUNB

	Logo	E-value	Sites	Width
1.	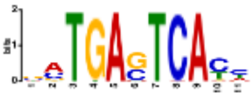	3.3e-1479	1346	11
2.	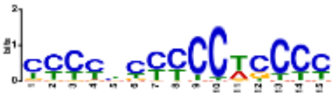	2.4e-308	1030	15
3.	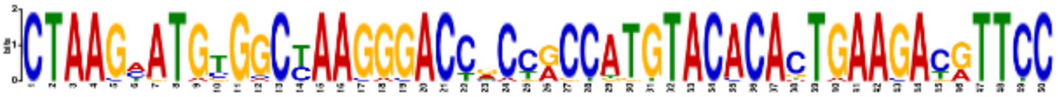	4.6e-235	17	50
4.	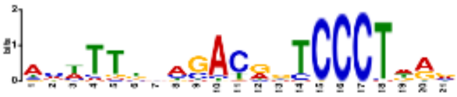	2.5e-196	219	21
5.	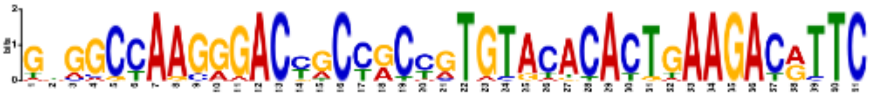	2.4e-087	13	41
6.	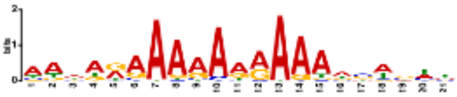	2.5e-063	171	21
7.	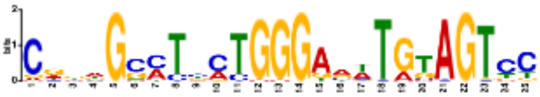	1.9e-050	33	25
8.	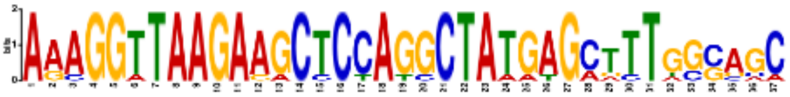	1.7e-038	8	37
9.	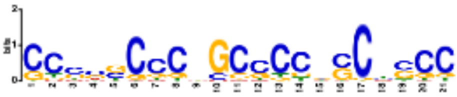	2.8e-037	281	21
10.	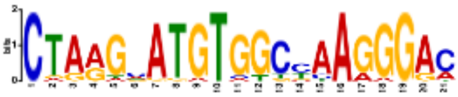	3.1e-022	17	21
11.	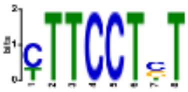	2.6e-018	263	8
12.	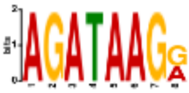	2.9e-020	128	8
13.	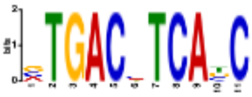	3.0e-015	77	11
14.	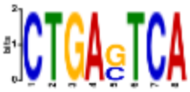	8.1e-013	133	8
15.	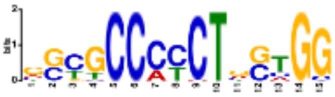	7.1e-017	67	15
16.	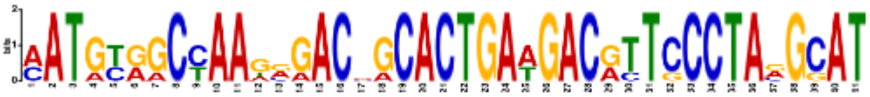	5.5e-010	5	41
17.	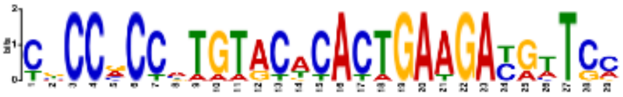	2.4e-007	8	29
18.	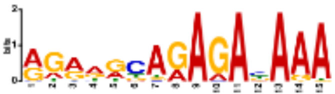	2.2e-004	67	15
19.	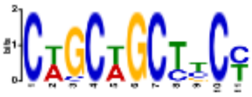	7.6e-013	104	11
20.	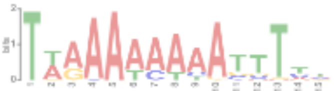	9.3e+000	36	15

RUNX1

		Logo	E-value	Sites	Width
1.	+ -	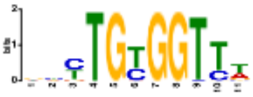	1.9e-1033	1303	11
2.	+ -	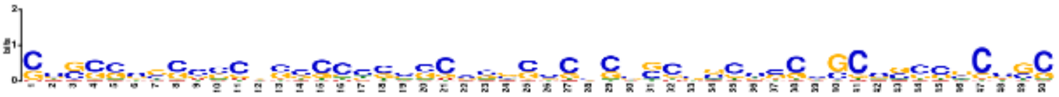	2.9e-507	849	50
3.	+ -	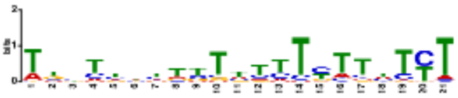	4.4e-151	335	21
4.	+ -	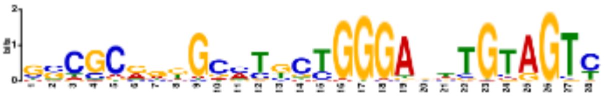	7.1e-148	74	28
5.	+ -	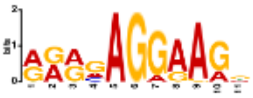	1.2e-074	457	11
6.	+ -	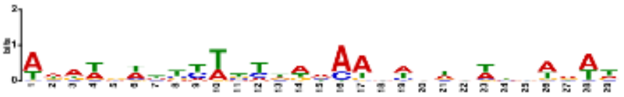	4.5e-056	265	29
7.	+ -	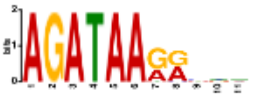	1.5e-019	139	11
8.	+ -	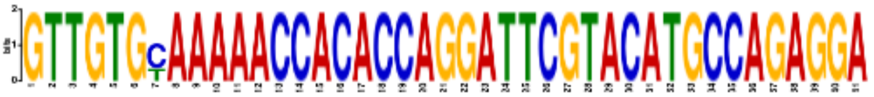	1.0e-005	3	41
9.	+ -	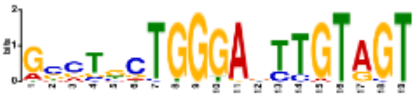	1.8e-005	20	19
10.	+ -	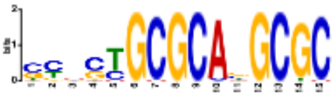	8.0e-008	53	15
11.	+ -	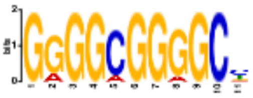	2.6e-008	86	11
12.	+ -	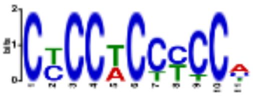	3.0e-008	147	11
13.	+ -	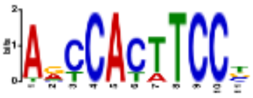	4.3e-004	76	11
14.	+ -	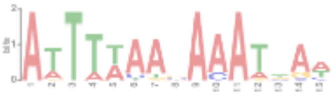	1.5e+003	18	15
15.	+ -	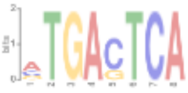	1.0e+004	177	8
16.	+ -	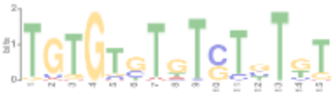	7.2e+000	32	15
17.	+ -	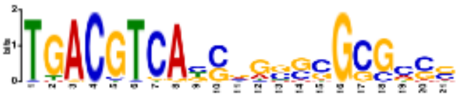	7.6e-004	32	21
18.	+ -	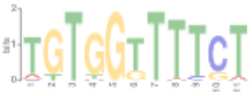	5.5e+004	26	11
19.	+ -	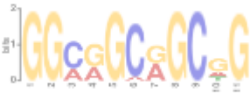	2.4e+004	104	11
20.	+ -	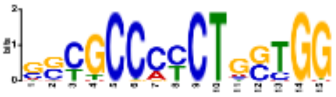	4.7e-002	53	15

P300

		Logo	E-value	Sites	Width
1.	+ -	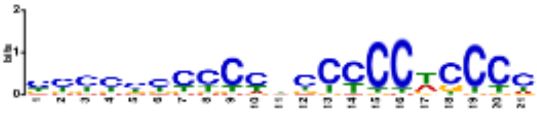	9.0e-2439	5147	21
2.	+ -	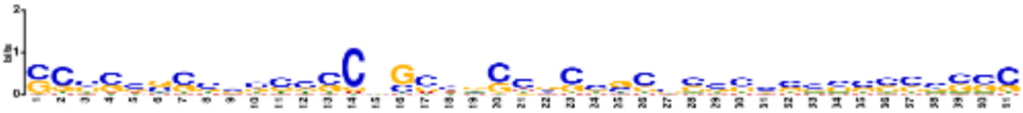	1.0e-1543	2372	41
3.	+ -	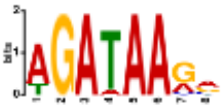	4.5e-1518	6252	8
4.	+ -	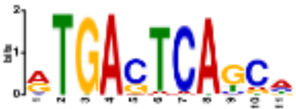	4.0e-1152	2371	11
5.	+ -	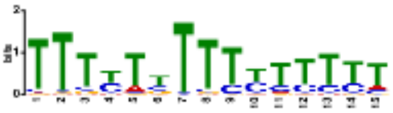	4.8e-569	1230	15
6.	+ -	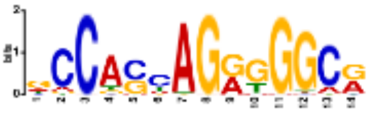	6.7e-447	821	14
7.	+ -	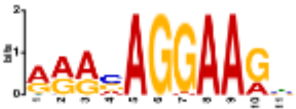	2.6e-396	1667	11
8.	+ -	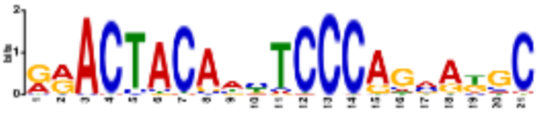	1.1e-294	170	21
9.	+ -	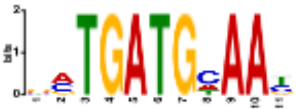	1.1e-260	763	11
10.	+ -	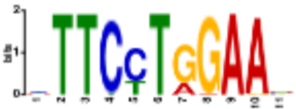	1.5e-263	649	11

MEME would crash for max 20 motifs. These results are with a max of 10 motifs (after about 11 days of execution).

		Logo	E-value	Sites	Width
1.	+ / -	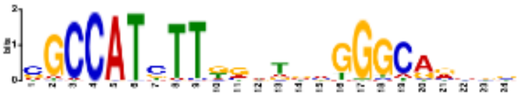	1.3e-513	245	24
2.	+ / -	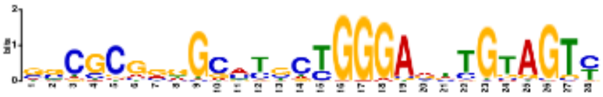	8.8e-466	199	28
3.	+ / -	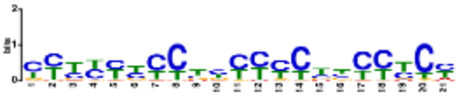	4.9e-286	823	21
4.	+ / -	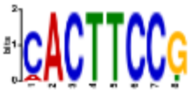	7.2e-169	439	8
5.	+ / -	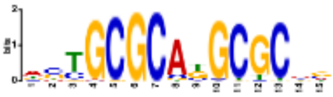	2.2e-214	429	15
6.	+ / -	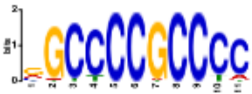	7.6e-197	668	11
7.	+ / -	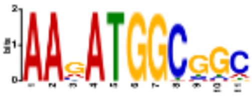	1.6e-141	221	11
8.	+ / -	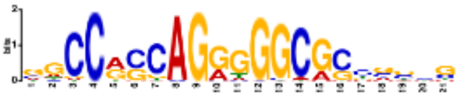	8.2e-117	209	21
9.	+ / -	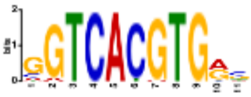	1.1e-105	234	11
10.	+ / -	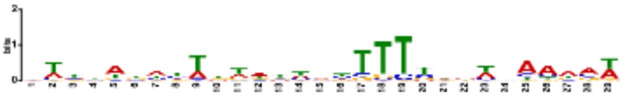	6.4e-062	134	29
11.	+ / -	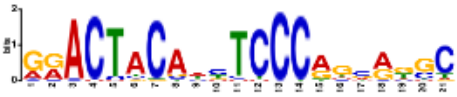	8.3e-060	60	21
12.	+ / -	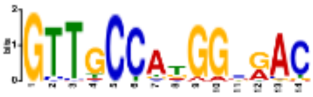	4.2e-084	143	14
13.	+ / -	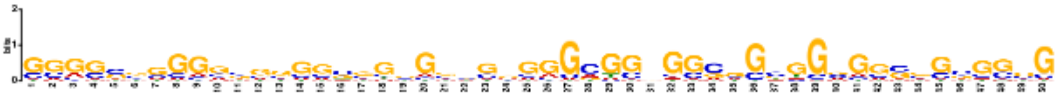	2.0e-054	442	50
14.	+ / -	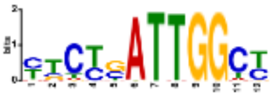	1.6e-055	175	12
15.	+ / -	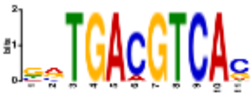	1.2e-029	108	11
16.	+ / -	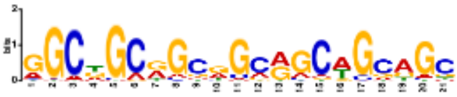	2.6e-033	160	21
17.	+ / -	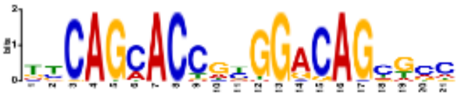	1.1e-028	34	21
18.	+ / -	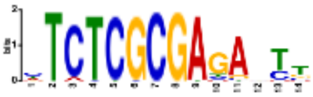	5.4e-022	57	14
19.	+ / -	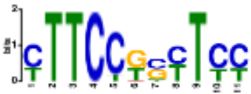	6.0e-026	180	11
20.	+ / -	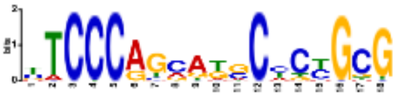	2.1e-018	78	18

		Logo	E-value	Sites	Width
1.	+ -	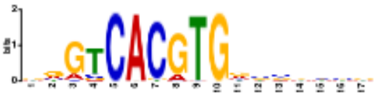	1.0e-1966	1825	17
2.	+ -	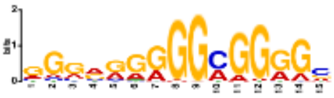	3.2e-186	618	15
3.	+ -	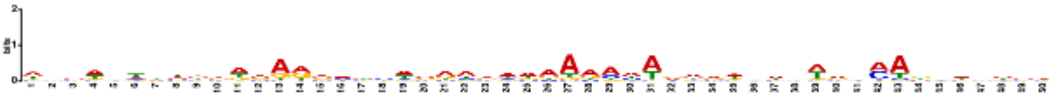	7.6e-258	356	50
4.	+ -	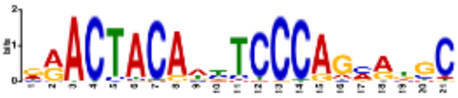	2.4e-114	63	21
5.	+ -	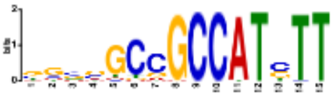	1.8e-065	104	15
6.	+ -	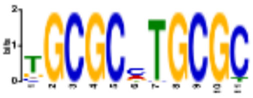	1.1e-058	109	11
7.	+ -	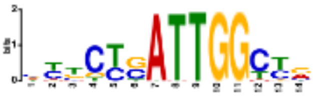	6.5e-070	178	14
8.	+ -	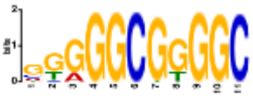	1.1e-044	156	11
9.	+ -	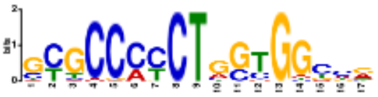	5.4e-028	88	17
10.	+ -	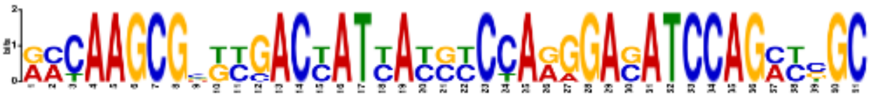	2.5e-017	6	41
11.	+ -	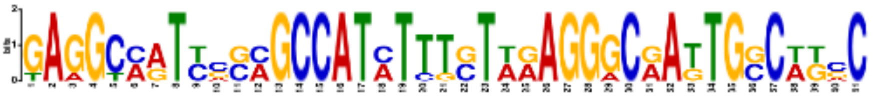	1.0e-015	6	41
12.	+ -	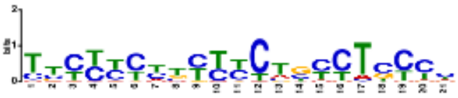	6.1e-013	195	21
13.	+ -	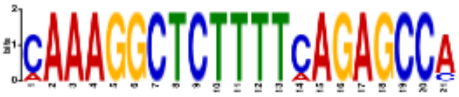	2.5e-009	6	21
14.	+ -	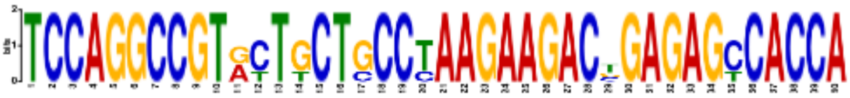	3.1e-010	4	40
15.	+ -	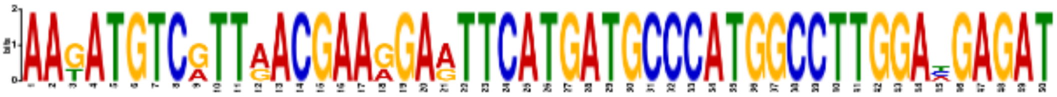	3.3e-007	3	50
16.	+ -	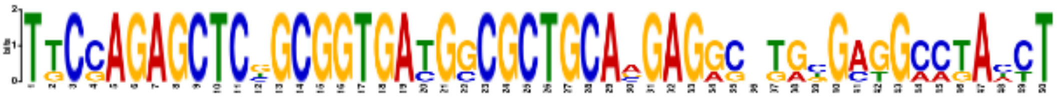	5.6e-006	4	50
17.	+ -	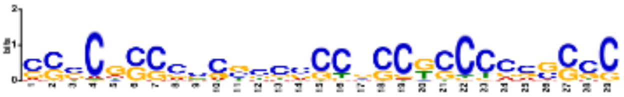	6.9e-019	148	29
18.	+ -	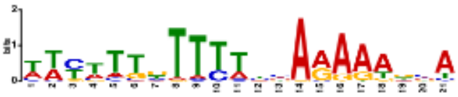	1.5e-004	40	21
19.	+ -	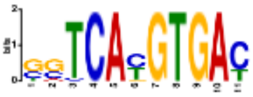	2.0e-002	61	11
20.	+ -	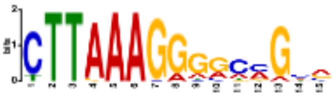	1.9e-005	38	15
