# Supplementary material for: DIVERSITY in binding, regulation, and evolution revealed from high-throughput ChIP
Source: PLoS Comput Biol. 2018 Apr 23;14(4):e1006090. doi: 10.1371/journal.pcbi.1006090 (PMC5933800; doi:10.1371/journal.pcbi.1006090)
Supplement: S7 Fig — (PDF) [file pcbi.1006090.s007.pdf]

(A) DREME output on REST

A549

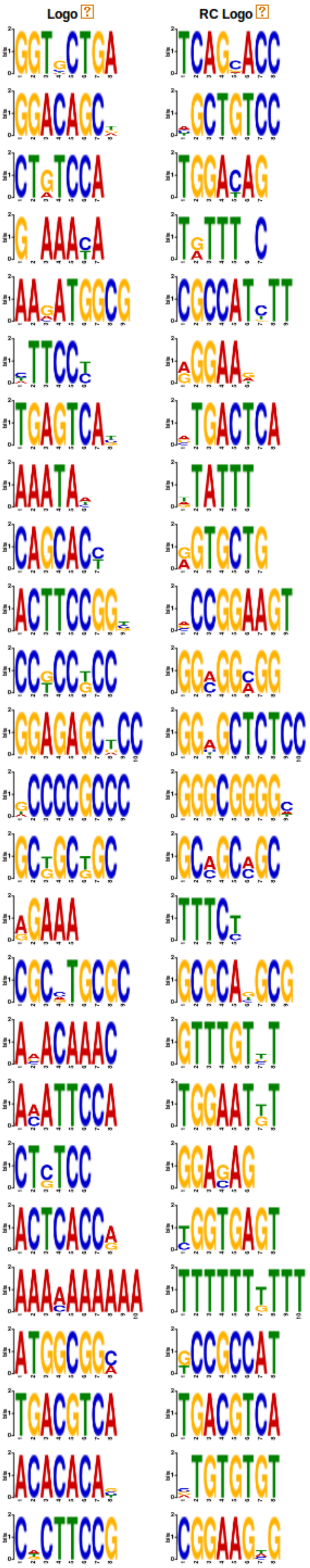

| | Motif | E-value | Unerased E-value |
|---|---|---|---|
| 1. | GGTNCTGA | 2.2e-261 | 2.2e-261 |
| 2. | GGACAGCD | 3.6e-229 | 1.7e-229 |
| 3. | CTRTCCA | 1.5e-037 | 3.1e-163 |
| 4. | GNAAAYA | 3.8e-037 | 5.7e-039 |
| 5. | AAVATGGCG | 7.6e-036 | 6.4e-035 |
| 6. | HTTCCY | 5.3e-029 | 2.3e-033 |
| 7. | TGAGTCAB | 1.7e-027 | 3.5e-030 |
| 8. | AAATAH | 7.7e-019 | 6.7e-033 |
| 9. | CAGCACY | 1.3e-018 | 5.0e-181 |
| 10. | ACTTCCGGB | 4.0e-016 | 7.1e-017 |
| 11. | CCKCCKCC | 3.4e-015 | 1.1e-019 |
| 12. | GGAGAGCDCC | 7.0e-015 | 2.0e-015 |
| 13. | DCCCCGCCC | 1.3e-008 | 2.2e-009 |
| 14. | GCKGCKGC | 3.5e-007 | 1.0e-017 |
| 15. | RGAAA | 3.4e-005 | 1.8e-021 |
| 16. | CGCVTGCGC | 1.1e-003 | 1.6e-004 |
| 17. | AVACAAAC | 2.4e-003 | 8.1e-005 |
| 18. | AMATTCCA | 3.4e-003 | 7.1e-004 |
| 19. | CTSTCC | 3.7e-003 | 3.2e-150 |
| 20. | ACTCACCR | 4.3e-003 | 1.7e-004 |
| 21. | AAAMAAAAAA | 8.6e-003 | 3.0e-003 |
| 22. | ATGGCGGM | 9.7e-003 | 3.2e-028 |
| 23. | TGACGTCA | 2.2e-002 | 3.5e-002 |
| 24. | ACACACAB | 2.2e-002 | 6.9e-004 |
| 25. | CDCTTCCG | 3.2e-002 | 2.1e-009 |

ECC-1

| | Motif | Logo | RC Logo | E-value | Unerased E-value |
|---|---|---|---|---|---|
| 1. | CTGTCCD | 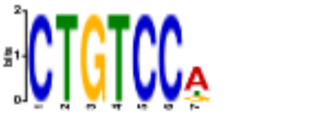 | 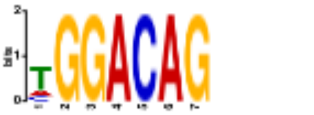 | 3.3e-607 | 3.3e-607 |
| 2. | GGTNCTGA | 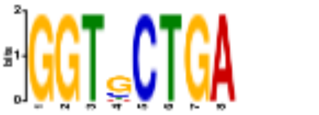 | 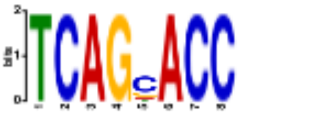 | 7.3e-353 | 1.4e-353 |
| 3. | GGASAGCD | 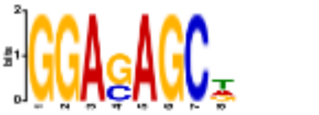 | 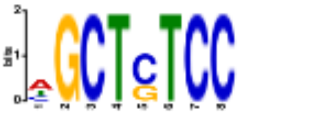 | 2.5e-042 | 2.9e-452 |
| 4. | CAGCACY | 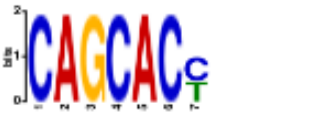 | 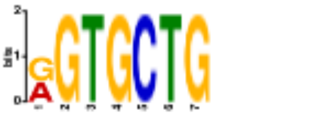 | 1.5e-034 | 1.2e-283 |
| 5. | DAAATR | 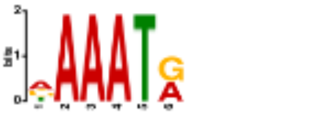 | 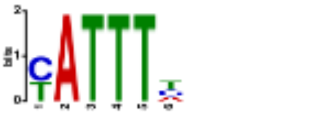 | 1.6e-024 | 3.1e-029 |
| 6. | AGRAA | 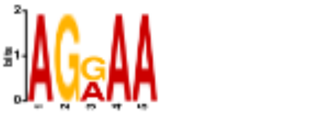 | 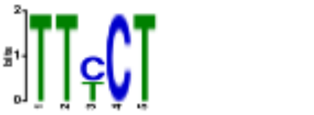 | 3.3e-016 | 2.6e-024 |
| 7. | ACCWTGGAKAG | 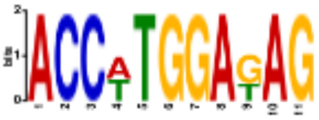 | 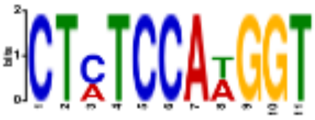 | 1.7e-012 | 3.0e-071 |
| 8. | CCCCRCCC | 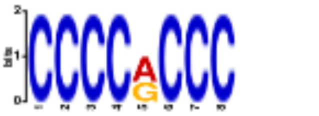 | 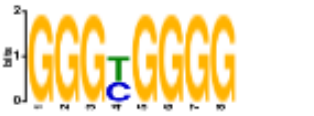 | 2.2e-009 | 9.4e-010 |
| 9. | GGAGCTR | 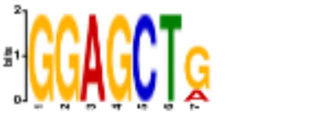 | 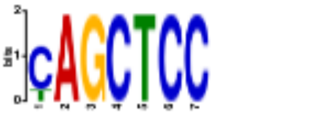 | 2.2e-008 | 1.1e-101 |
| 10. | CCRGGA | 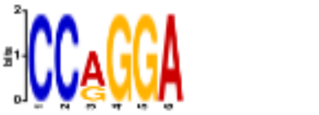 | 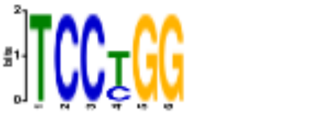 | 7.5e-005 | 3.4e-015 |
| 11. | ACGTGD | 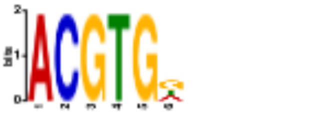 | 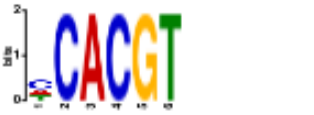 | 2.3e-004 | 5.2e-005 |
| 12. | GGGAAGGSA | 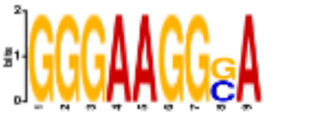 | 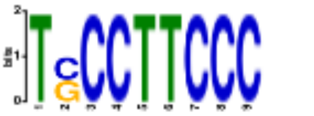 | 2.3e-003 | 3.2e-003 |
| 13. | BACACACA | 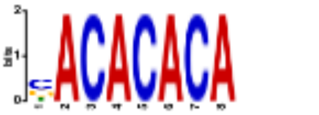 | 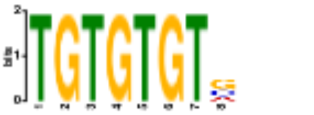 | 2.8e-003 | 5.7e-004 |
| 14. | GTKCTGAA | 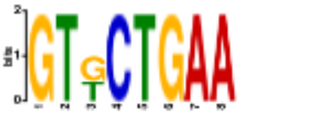 | 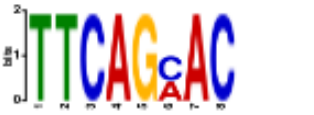 | 1.4e-002 | 8.9e-219 |
| 15. | GCCGSCGCC | 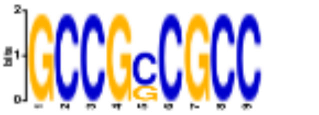 | 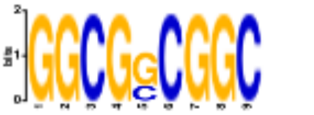 | 4.5e-002 | 5.6e-003 |

GM12878

| | Motif | Logo | RC Logo | E-value | Unerased E-value |
|---|---|---|---|---|---|
| 1. | GGTNCTGA | 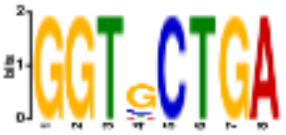 | 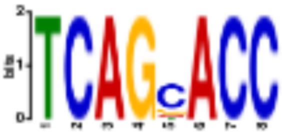 | 1.9e-334 | 1.9e-334 |
| 2. | DCTGTCC | 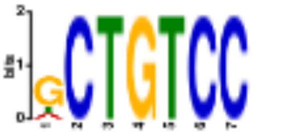 | 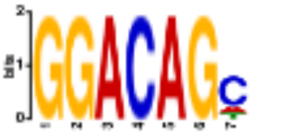 | 2.7e-316 | 2.7e-316 |
| 3. | CAGCACY | 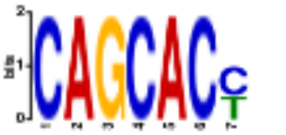 | 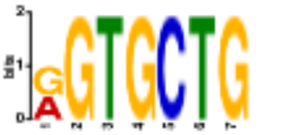 | 6.0e-037 | 1.6e-267 |
| 4. | GGAKAGCK | 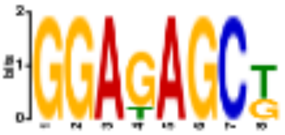 | 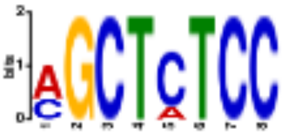 | 2.1e-022 | 9.1e-024 |
| 5. | RGGAAR | 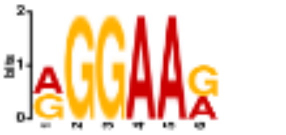 | 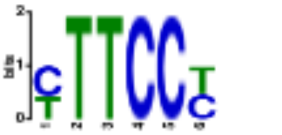 | 1.5e-012 | 3.8e-013 |
| 6. | DAAATA | 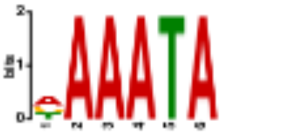 | 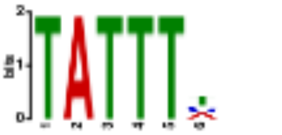 | 8.6e-010 | 8.2e-014 |
| 7. | AKAAA | 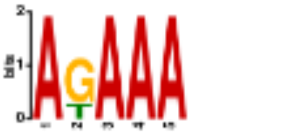 | 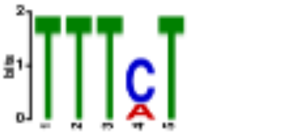 | 6.8e-007 | 1.7e-011 |
| 8. | ATGGABAG | 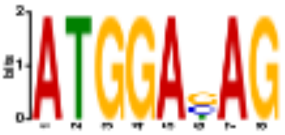 | 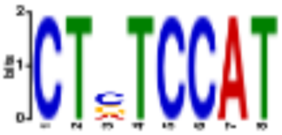 | 1.9e-005 | 1.0e-076 |
| 9. | CCAGKACC | 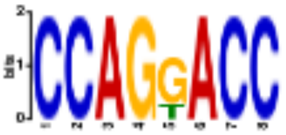 | 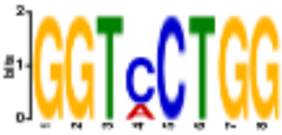 | 6.1e-003 | 1.1e-002 |
| 10. | CCCCRCCC | 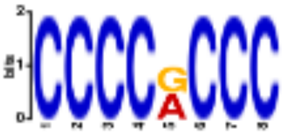 | 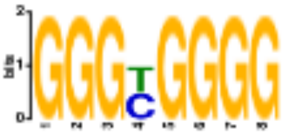 | 7.0e-003 | 8.8e-004 |
| 11. | CCYTGTCC | 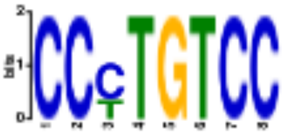 | 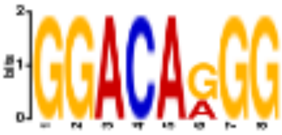 | 7.1e-003 | 4.9e-006 |
| 12. | AAAMAAAAA | 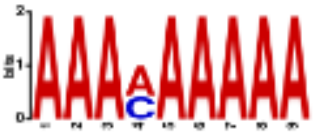 | 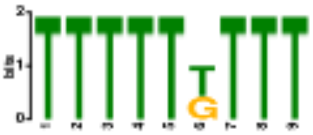 | 2.2e-002 | 1.4e-004 |

# H1ESC

| | Motif | Logo | RC Logo | E-value | Unerased E-value |
|---|---|---|---|---|---|
| **1.** | DCTGTCC | 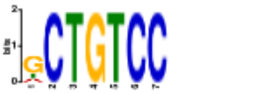 | 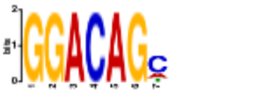 | 4.2e-762 | 4.2e-762 |
| **2.** | RGTNCTGA | 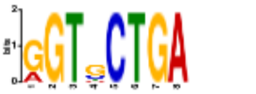 | 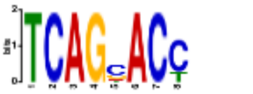 | 3.3e-485 | 6.2e-486 |
| **3.** | ACCWTGGABAG | 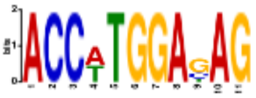 | 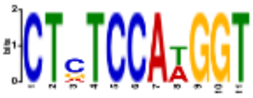 | 1.3e-084 | 6.2e-309 |
| **4.** | CAGBACC | 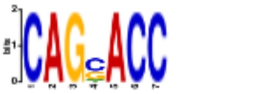 | 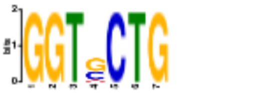 | 4.7e-050 | 4.3e-467 |
| **5.** | RGAAA | 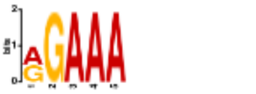 | 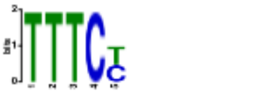 | 7.7e-048 | 9.6e-051 |
| **6.** | AAATAH | 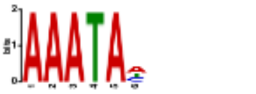 | 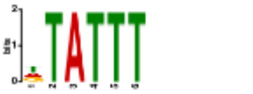 | 3.6e-028 | 1.6e-041 |
| **7.** | GCTMTCCA | 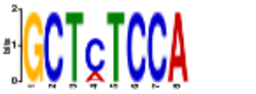 | 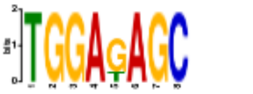 | 2.6e-021 | 5.8e-054 |
| **8.** | CCCCRMCCC | 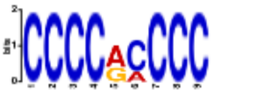 | 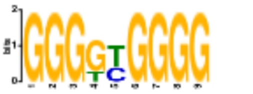 | 5.3e-018 | 6.5e-019 |
| **9.** | GGACAGGD | 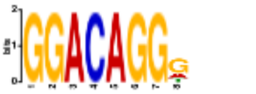 | 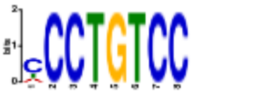 | 3.8e-015 | 2.4e-026 |
| **10.** | CMTTCCY | 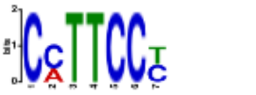 | 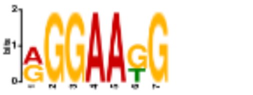 | 3.3e-014 | 4.8e-018 |
| **11.** | ATTTWH | 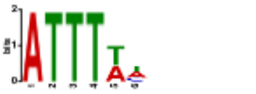 | 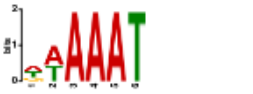 | 1.5e-012 | 2.8e-037 |
| **12.** | SCYGGGA | 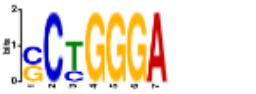 | 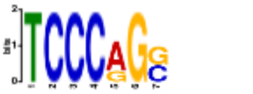 | 5.8e-009 | 1.6e-021 |
| **13.** | CACGTD | 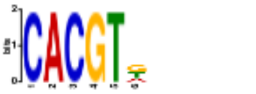 | 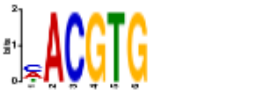 | 4.4e-007 | 1.6e-009 |
| **14.** | GSAGGRA | 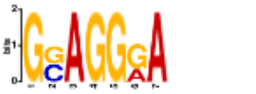 | 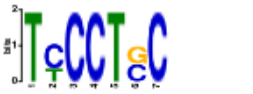 | 2.0e-006 | 1.1e-018 |
| **15.** | AMACRCA | 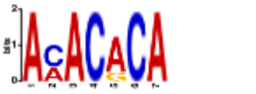 | 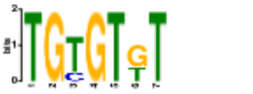 | 1.2e-006 | 8.0e-011 |
| **16.** | TGGYCAGCR | 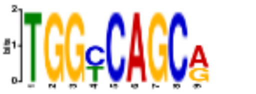 | 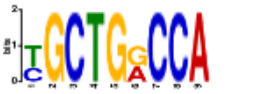 | 1.7e-006 | 4.8e-017 |
| **17.** | CSSCGCC | 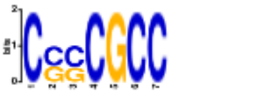 | 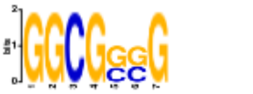 | 2.4e-004 | 1.3e-010 |
| **18.** | CAGCNCTG | 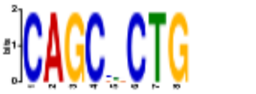 | 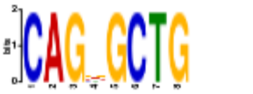 | 1.6e-003 | 2.1e-032 |
| **19.** | CCWGGA | 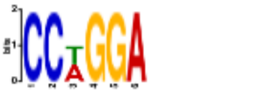 | 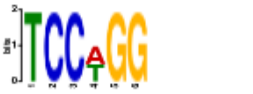 | 4.7e-002 | 1.3e-040 |

## HCT-116

| Motif | Logo | RC Logo | E-value | Unerased E-value |
|---|---|---|---|---|
| **1.** DCTGTCC | 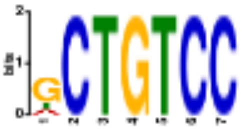 | 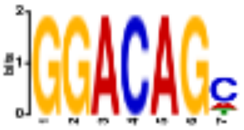 | 5.6e-350 | 5.6e-350 |
| **2.** GGTNCTGA | 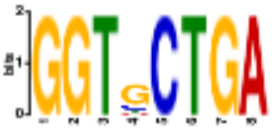 | 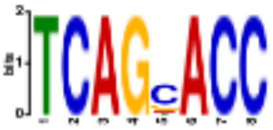 | 9.8e-308 | 9.8e-308 |
| **3.** GGAKAGCKC | 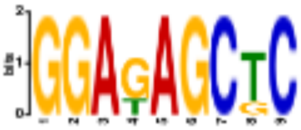 | 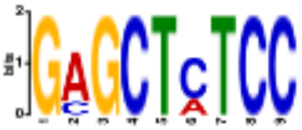 | 4.5e-025 | 1.3e-025 |
| **4.** GTGCTGRA | 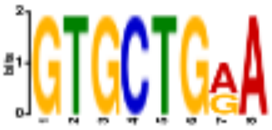 | 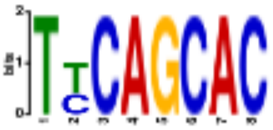 | 3.2e-021 | 2.2e-170 |
| **5.** DAAATA | 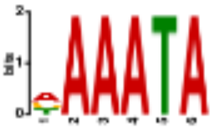 | 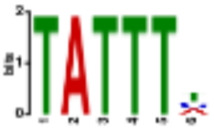 | 4.3e-014 | 4.0e-016 |
| **6.** CTSTCCAW | 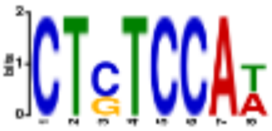 | 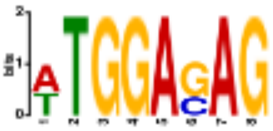 | 2.0e-013 | 1.6e-137 |
| **7.** CAGNACC | 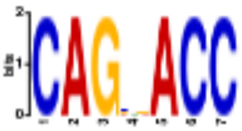 | 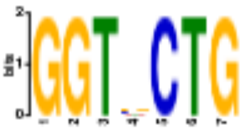 | 5.8e-011 | 1.2e-288 |
| **8.** AGGAAD | 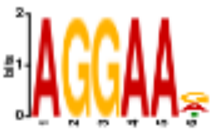 | 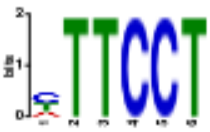 | 1.8e-007 | 3.7e-008 |
| **9.** AAAAARAAAA | 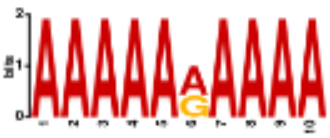 | 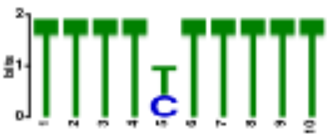 | 2.9e-003 | 7.7e-003 |
| **10.** AKAAA | 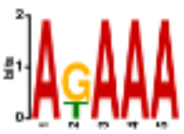 | 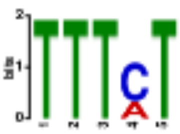 | 1.5e-002 | 1.7e-006 |
| **11.** CCCRSCC | 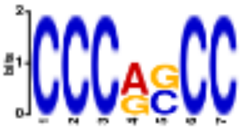 | 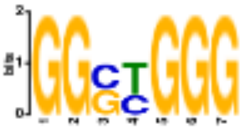 | 3.2e-002 | 1.1e-002 |

# HL-60

| | Motif | Logo | RC Logo | E-value | Unerased E-value |
|---|---|---|---|---|---|
| 1. | DCTGTCC | 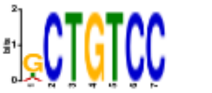 | 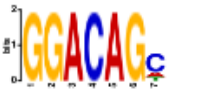 | 1.5e-389 | 1.5e-389 |
| 2. | GGTNCTGA | 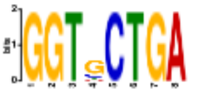 | 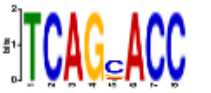 | 2.1e-330 | 2.1e-330 |
| 3. | CAGCACYD | 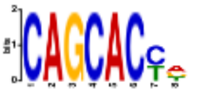 | 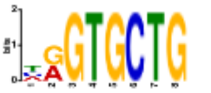 | 2.4e-038 | 6.1e-252 |
| 4. | GCTMTCCA | 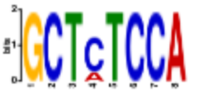 | 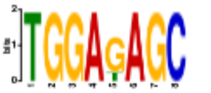 | 1.9e-027 | 2.4e-028 |
| 5. | RDAGAAA | 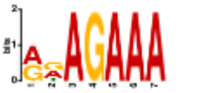 | 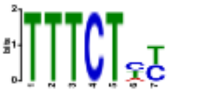 | 3.3e-025 | 7.1e-026 |
| 6. | AARATGGCG | 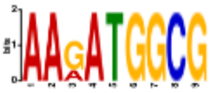 | 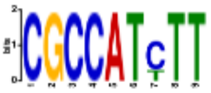 | 1.4e-023 | 9.6e-025 |
| 7. | AWATA | 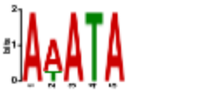 | 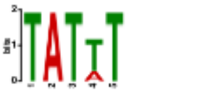 | 1.5e-020 | 6.3e-024 |
| 8. | RGGAAR | 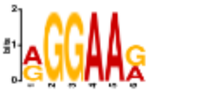 | 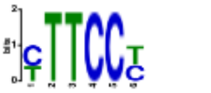 | 1.7e-017 | 1.7e-021 |
| 9. | GCCGCCRY | 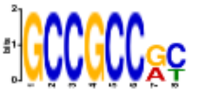 | 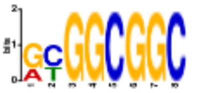 | 1.4e-012 | 1.2e-020 |
| 10. | GGGGCGGGR | 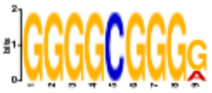 | 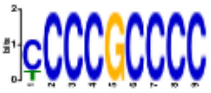 | 3.6e-009 | 1.7e-010 |
| 11. | HCCTGTCCA | 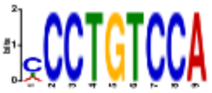 | 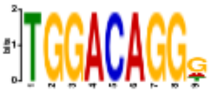 | 1.4e-007 | 8.2e-008 |
| 12. | CAGDACC | 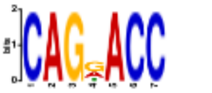 | 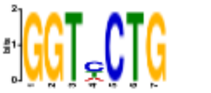 | 4.5e-007 | 2.2e-065 |
| 13. | GTGCTGRA | 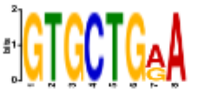 | 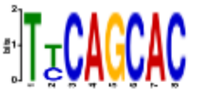 | 4.4e-006 | 2.4e-215 |
| 14. | ACTTCCGS | 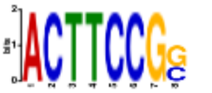 | 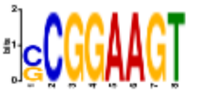 | 1.2e-005 | 7.9e-006 |
| 15. | GAATRAA | 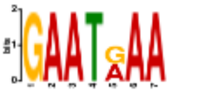 | 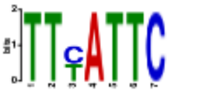 | 3.6e-004 | 6.9e-006 |
| 16. | CMCMGCC | 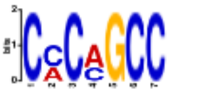 | 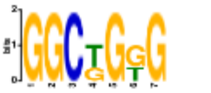 | 3.1e-005 | 2.6e-012 |
| 17. | GGARA | 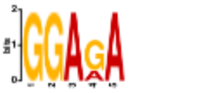 | 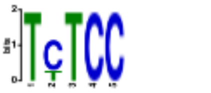 | 8.7e-004 | 7.6e-022 |
| 18. | RTTTTMA | 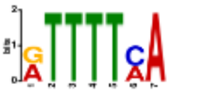 | 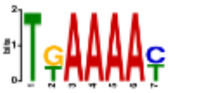 | 1.3e-003 | 3.2e-010 |
| 19. | AAAMAAAAA | 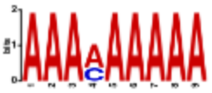 | 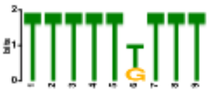 | 1.9e-002 | 2.9e-003 |

HeLaS3

| | Motif | Logo | RC Logo | E-value | Unerased E-value |
|---|---|---|---|---|---|
| 1. | DCTGTCC | 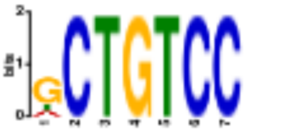 | 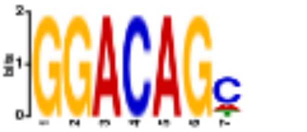 | 1.9e-471 | 1.9e-471 |
| 2. | GGTNCTGA | 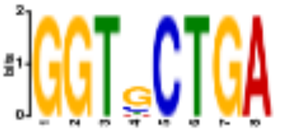 | 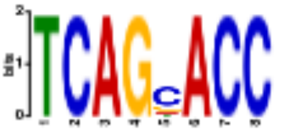 | 3.3e-368 | 3.3e-368 |
| 3. | GTGCTGRA | 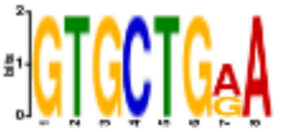 | 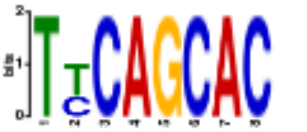 | 1.7e-040 | 1.3e-224 |
| 4. | HGCTMTCCA | 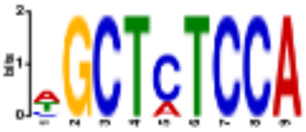 | 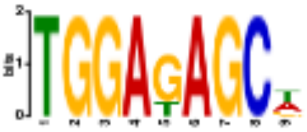 | 1.6e-039 | 2.3e-041 |
| 5. | RKAAA | 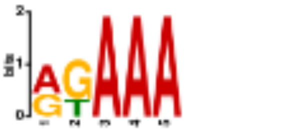 | 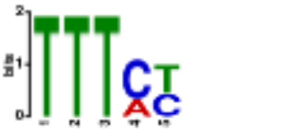 | 2.2e-021 | 1.1e-022 |
| 6. | CAGBACC | 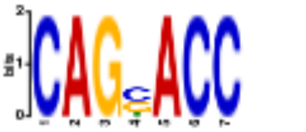 | 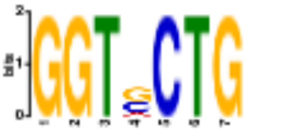 | 2.1e-019 | 1.0e-341 |
| 7. | GGACAGGGM | 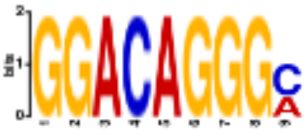 | 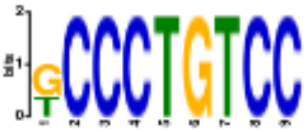 | 4.2e-009 | 4.7e-010 |
| 8. | AGGAAG | 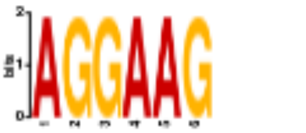 | 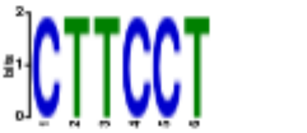 | 6.6e-007 | 1.3e-008 |
| 9. | AAATAY | 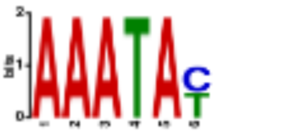 | 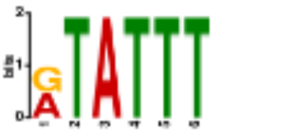 | 1.0e-006 | 5.1e-019 |
| 10. | CCCCRSCC | 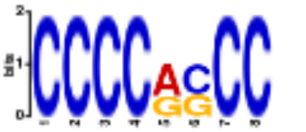 | 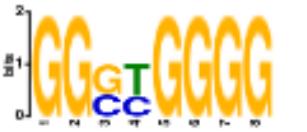 | 3.6e-005 | 8.3e-006 |
| 11. | AYGGAGAG | 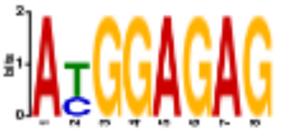 | 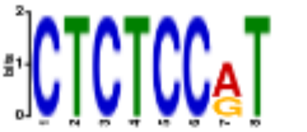 | 1.8e-004 | 1.3e-013 |
| 12. | CAGYTCC | 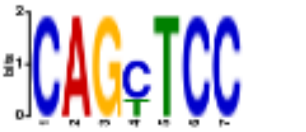 | 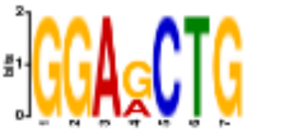 | 4.3e-002 | 4.2e-071 |
| 13. | CGTGK | 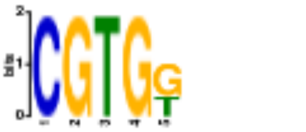 | 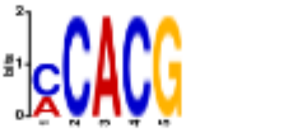 | 3.2e-002 | 1.2e-025 |

HepG2

| Motif | Logo | RC Logo | E-value | Unerased E-value |
|---|---|---|---|---|
| **1.** DCTGTCC | 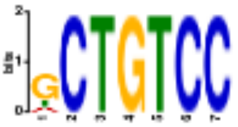 | 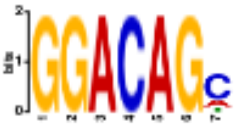 | 1.6e-381 | 1.6e-381 |
| **2.** GGTNCTGA | 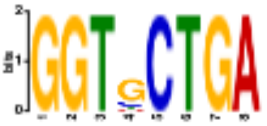 | 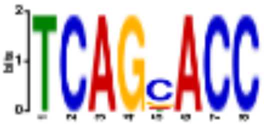 | 2.2e-297 | 2.2e-297 |
| **3.** CAGCACY | 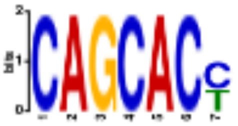 | 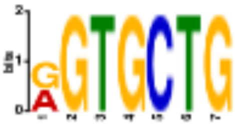 | 8.4e-027 | 1.7e-234 |
| **4.** HGCTMTCCA | 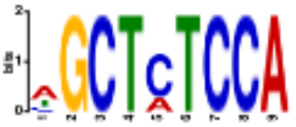 | 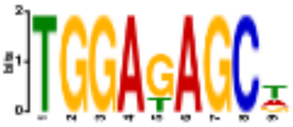 | 8.8e-023 | 6.3e-023 |
| **5.** DRGAAA | 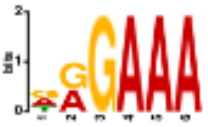 | 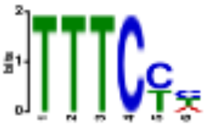 | 6.3e-015 | 1.4e-014 |
| **6.** AAATAH | 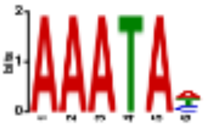 | 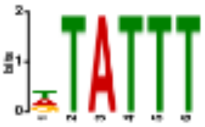 | 5.0e-008 | 1.0e-010 |
| **7.** GGACAGGGMY | 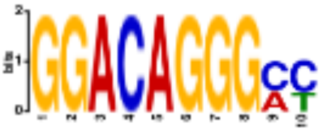 | 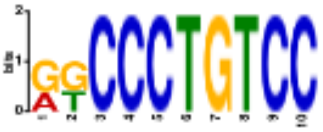 | 1.6e-006 | 9.8e-006 |
| **8.** CTTCCY | 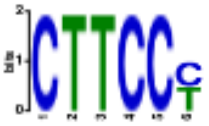 | 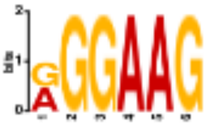 | 5.4e-006 | 5.1e-008 |
| **9.** CCCANCCC | 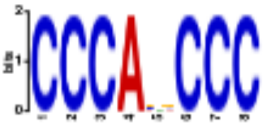 | 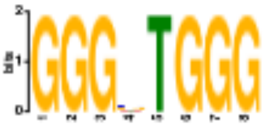 | 1.0e-004 | 1.9e-006 |
| **10.** CAGKACC | 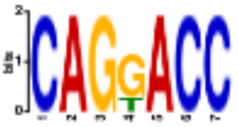 | 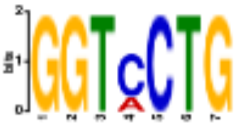 | 5.2e-004 | 3.0e-043 |
| **11.** ATGGASA | 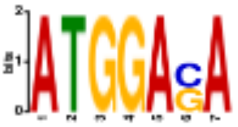 | 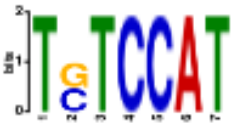 | 4.9e-003 | 2.3e-061 |
| **12.** ATTTH | 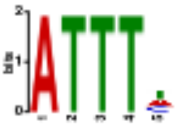 | 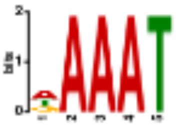 | 3.9e-002 | 1.2e-017 |

# K562

| | Motif | Logo | RC Logo | E-value | Unerased E-value |
|---|---|---|---|---|---|
| **1.** | CTGTCCR | | | 1.4e-403 | 1.4e-403 |
| **2.** | GGTNCTGA | | | 2.4e-322 | 1.1e-322 |
| **3.** | TTATCDB | | | 1.1e-106 | 1.2e-109 |
| **4.** | GGASAGCDCC | | | 3.2e-055 | 1.5e-226 |
| **5.** | AGGAAD | | | 9.3e-042 | 4.1e-045 |
| **6.** | CAGCACY | | | 4.9e-037 | 8.9e-264 |
| **7.** | ADATA | | | 6.1e-024 | 7.9e-058 |
| **8.** | CCCMRCCC | | | 2.4e-022 | 3.8e-024 |
| **9.** | CTGTCYY | | | 8.5e-020 | 5.8e-043 |
| **10.** | RTGACTCA | | | 3.5e-016 | 1.6e-017 |
| **11.** | KTTTCY | | | 1.7e-014 | 2.4e-029 |
| **12.** | CBGGGAA | | | 3.7e-010 | 1.2e-014 |
| **13.** | ACCHTGGAGAG | | | 2.8e-009 | 2.5e-048 |
| **14.** | CTCCCDGC | | | 6.8e-007 | 2.1e-010 |
| **15.** | GCTGTK | | | 2.4e-005 | 3.2e-010 |
| **16.** | AAAAAAAAAM | | | 3.3e-005 | 1.2e-005 |
| **17.** | CACGTK | | | 7.5e-004 | 5.1e-008 |
| **18.** | CCDCCTCC | | | 1.3e-003 | 2.1e-007 |
| **19.** | TGGADA | | | 3.7e-003 | 1.5e-017 |
| **20.** | CACMCRC | | | 1.0e-003 | 2.0e-010 |
| **21.** | GAKGGAGGGA | | | 4.2e-002 | 2.0e+000 |

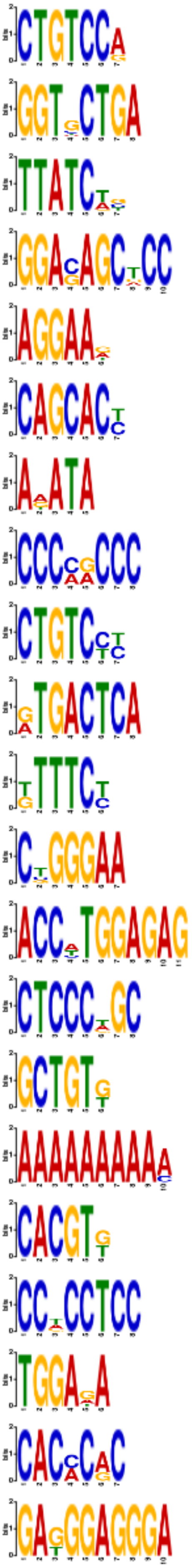

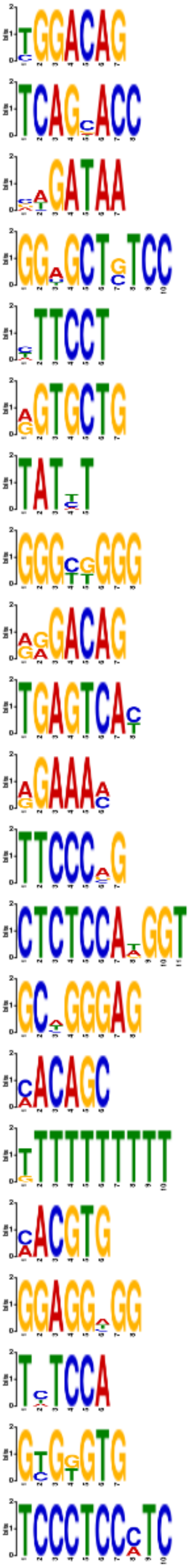

# MCF-7

| | Motif | Logo | RC Logo | E-value | Unerased E-value |
|---|---|---|---|---|---|
| **1.** | DCTGTCC | 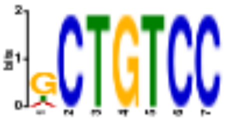 | 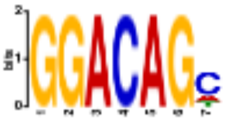 | 1.3e-399 | 1.3e-399 |
| **2.** | GGTNCTGA | 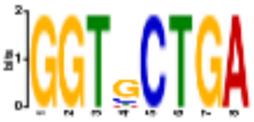 | 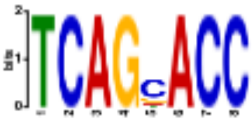 | 2.4e-361 | 2.4e-361 |
| **3.** | ACCWTGGABAG | 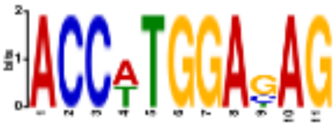 | 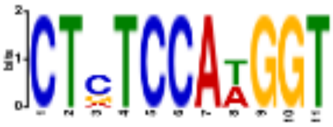 | 1.3e-035 | 5.3e-219 |
| **4.** | DAAATR | 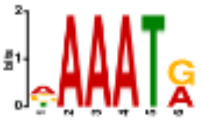 | 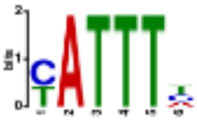 | 9.6e-027 | 8.4e-031 |
| **5.** | CAGCACY | 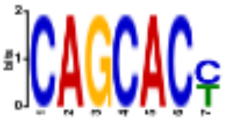 | 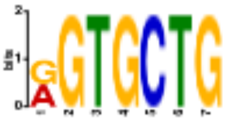 | 3.5e-024 | 2.7e-256 |
| **6.** | CTKTCC | 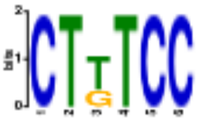 | 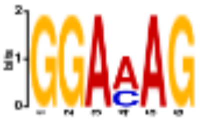 | 9.3e-013 | 1.2e-321 |
| **7.** | GAGCTMTCC | 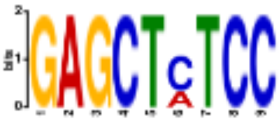 | 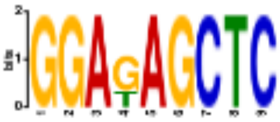 | 1.4e-012 | 1.9e-024 |
| **8.** | GGARRAA | 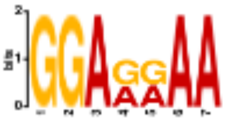 | 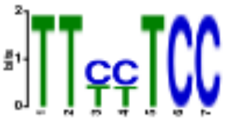 | 8.3e-009 | 1.5e-013 |
| **9.** | CCCHKCCC | 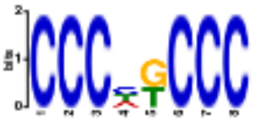 | 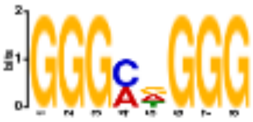 | 2.7e-006 | 6.3e-007 |
| **10.** | CCDGGA | 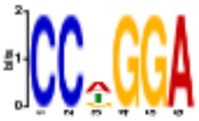 | 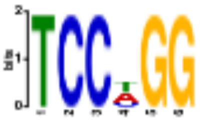 | 7.0e-005 | 2.7e-019 |
| **11.** | AAAYAMA | 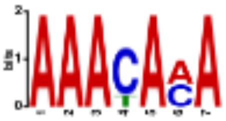 | 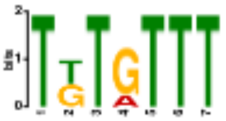 | 8.1e-005 | 3.8e-014 |
| **12.** | CCKCCKCC | 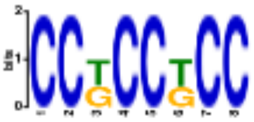 | 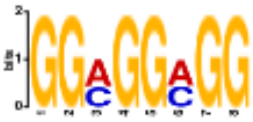 | 8.7e-003 | 7.9e-006 |

# Neuron

| | Motif | Logo / RC Logo | E-value | Unerased E-value |
|---|---|---|---|---|
| 1. | HTTCCY | 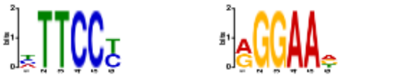 | 3.5e-147 | 3.5e-147 |
| 2. | VTGACAB | 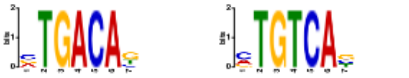 | 1.3e-068 | 8.5e-081 |
| 3. | TGGAAWW | 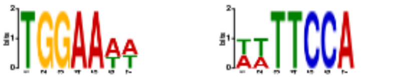 | 7.4e-052 | 1.5e-058 |
| 4. | GCDGCYGC | 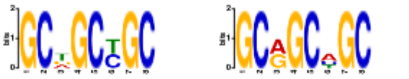 | 6.4e-054 | 4.0e-035 |
| 5. | AAVATGGCG | 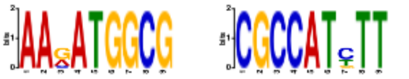 | 1.2e-041 | 3.7e-053 |
| 6. | ACAGC | 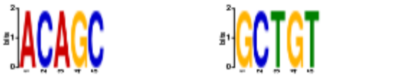 | 1.3e-031 | 1.2e-054 |
| 7. | CHGGGA | 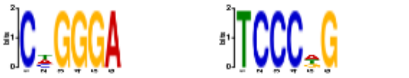 | 1.5e-028 | 5.7e-088 |
| 8. | GGTGCTGAA | 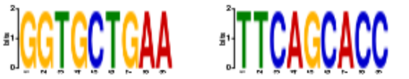 | 1.2e-026 | 8.8e-028 |
| 9. | CYGCMGCC | 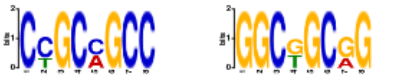 | 6.3e-023 | 2.1e-038 |
| 10. | RGAGAMA | 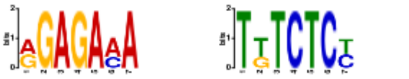 | 8.9e-022 | 7.4e-031 |
| 11. | CCCCDCCCCC | 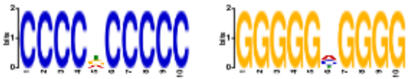 | 8.2e-019 | 4.4e-021 |
| 12. | CCATGGNAAC | 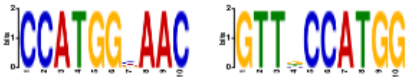 | 1.2e-014 | 9.1e-018 |
| 13. | STGWCA | 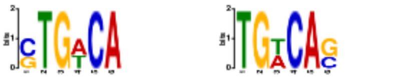 | 3.4e-014 | 1.0e-076 |
| 14. | ATTAY | 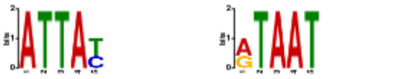 | 5.4e-014 | 5.0e-021 |
| 15. | CDCCKCC | 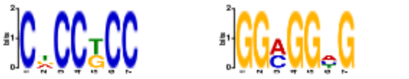 | 1.6e-010 | 2.0e-027 |
| 16. | CASWCAC | 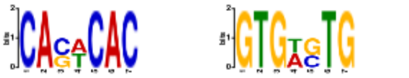 | 2.9e-009 | 1.9e-015 |
| 17. | RKAAA | 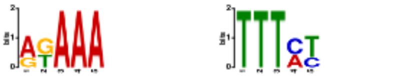 | 1.9e-007 | 3.4e-037 |
| 18. | AAAAAAAAA | 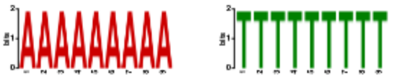 | 5.9e-004 | 2.1e-008 |
| 19. | CGCMTGCGCA | 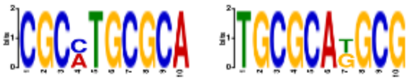 | 1.1e-003 | 2.8e-004 |
| 20. | CCGCKGC | 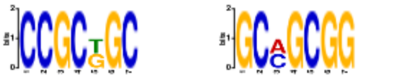 | 3.7e-003 | 3.1e-015 |
| 21. | STTGTCA | 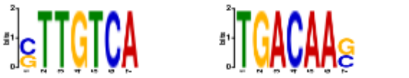 | 9.3e-003 | 9.4e-012 |
| 22. | DGGAA | 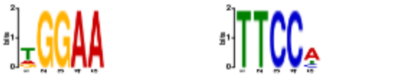 | 1.0e-002 | 3.8e-063 |
| 23. | AGRCAG | 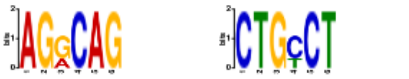 | 1.6e-002 | 8.6e-019 |

PANC-1

| Motif | Logo | RC Logo | E-value | Unerased E-value |
|---|---|---|---|---|
| **1.** DCTGTCC | 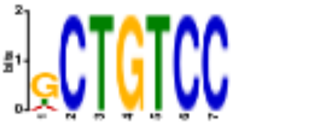 | 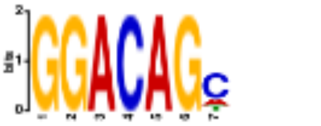 | 1.1e-369 | 1.1e-369 |
| **2.** GGTNCTGA | 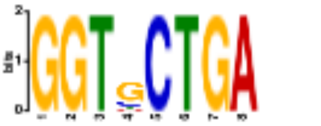 | 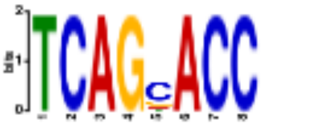 | 4.0e-323 | 4.0e-323 |
| **3.** CTVTCCAW | 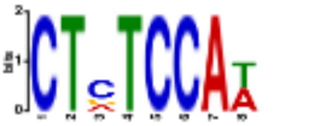 | 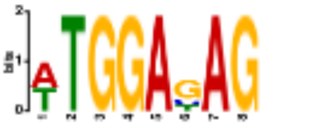 | 1.2e-038 | 1.7e-162 |
| **4.** CAGCACY | 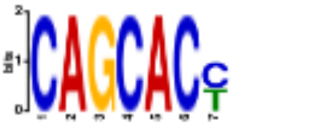 | 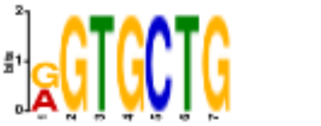 | 1.5e-031 | 9.0e-253 |
| **5.** AAATAH | 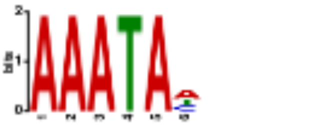 | 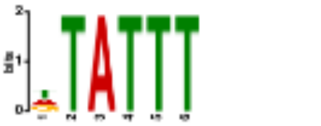 | 1.0e-017 | 2.2e-018 |
| **6.** AGRAAD | 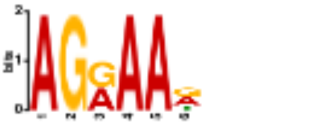 | 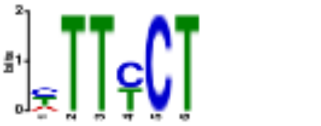 | 1.5e-013 | 3.2e-016 |
| **7.** GGAGCTMTCC | 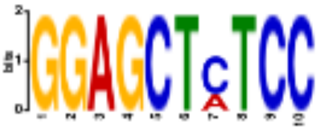 | 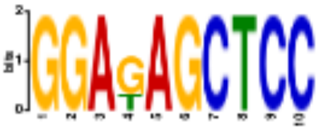 | 9.3e-012 | 8.1e-027 |
| **8.** CCCCDCCCC | 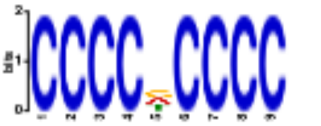 | 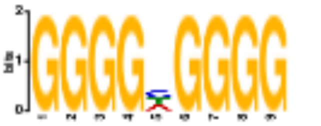 | 3.3e-007 | 1.2e-007 |
| **9.** CAGKACC | 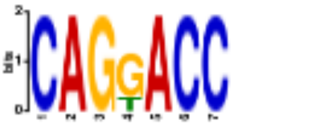 | 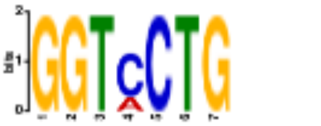 | 7.2e-006 | 4.2e-054 |
| **10.** CNGCCGCCGC | 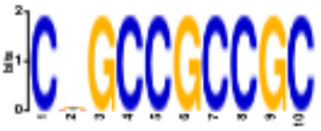 | 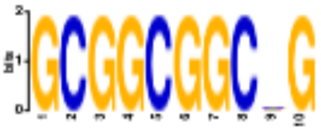 | 1.7e-002 | 3.7e-004 |

# PFSK-1

| | Motif | Logo | RC Logo | E-value | Unerased E-value |
|---|---|---|---|---|---|
| 1. | DCTGTCC | 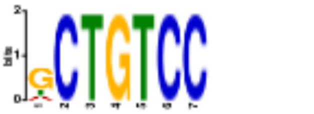 | 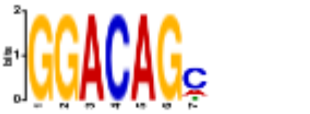 | 7.6e-416 | 7.6e-416 |
| 2. | GGTNCTGA | 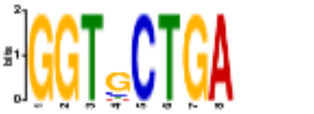 | 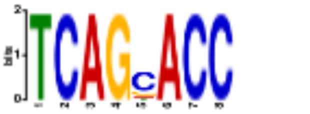 | 5.5e-299 | 5.5e-299 |
| 3. | AGGAAD | 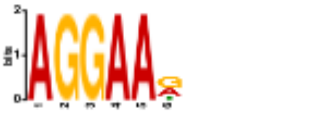 | 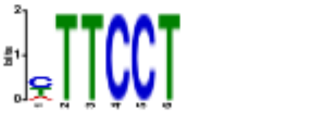 | 2.1e-028 | 2.3e-028 |
| 4. | ACCWTGGABAG | 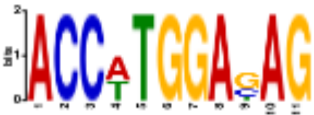 | 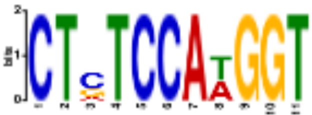 | 1.4e-025 | 1.3e-180 |
| 5. | GTGCTGRA | 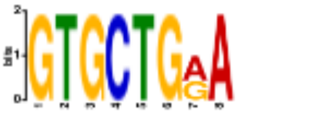 | 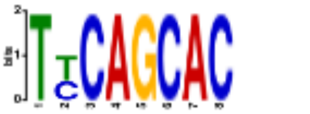 | 3.0e-022 | 4.1e-180 |
| 6. | RKAAA | 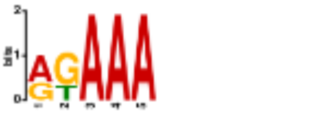 | 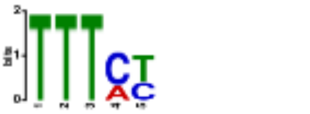 | 1.7e-015 | 8.7e-020 |
| 7. | CCCCRCCC | 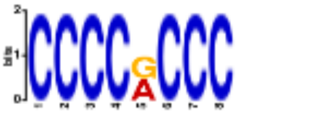 | 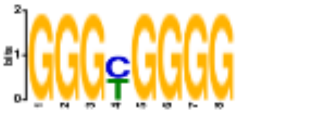 | 2.8e-010 | 2.7e-010 |
| 8. | TGGTGCTR | 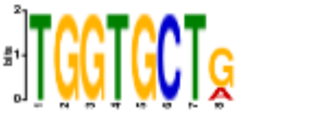 | 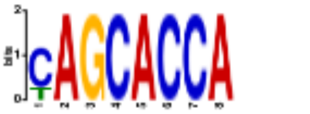 | 2.7e-010 | 2.2e-154 |
| 9. | GGAGCTMTCC | 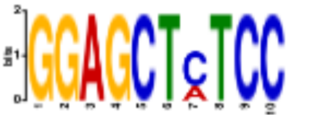 | 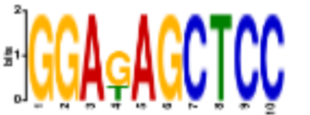 | 1.9e-007 | 1.5e-013 |
| 10. | CCDGGA | 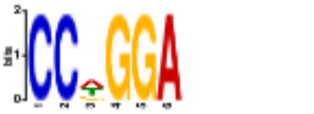 | 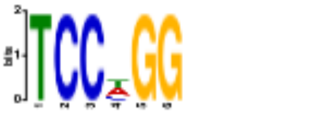 | 6.2e-006 | 1.2e-024 |
| 11. | TCCCGCB | 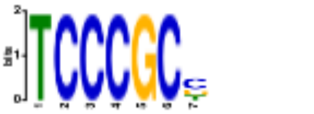 | 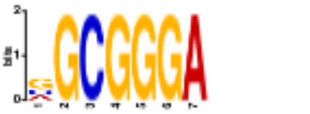 | 8.6e-004 | 3.6e-005 |
| 12. | GACGTCAS | 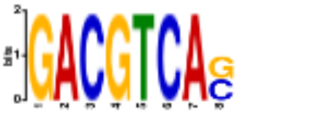 | 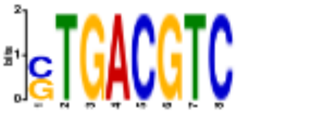 | 4.4e-003 | 1.2e-003 |
| 13. | AGYTCTCAGC | 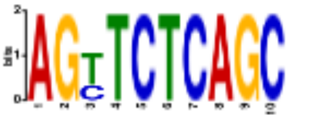 | 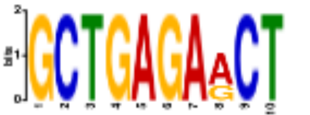 | 1.1e-002 | 1.4e-003 |
| 14. | CKGGGA | 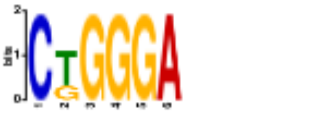 | 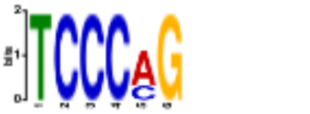 | 1.9e-002 | 1.7e-009 |
| 15. | AGCAGYGGC | 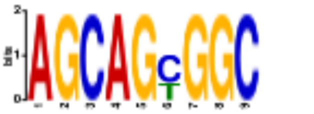 | 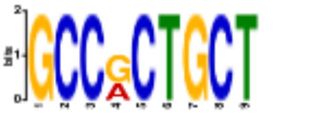 | 2.5e-002 | 2.5e-002 |

# SK-N-SH

| | Motif | Logo | RC Logo | E-value | Unerased E-value |
|---|---|---|---|---|---|
| 1. | DCTGTCC | 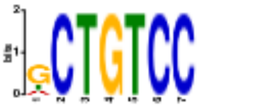 | 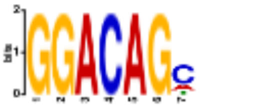 | 2.1e-434 | 2.1e-434 |
| 2. | GGTNCTGA | 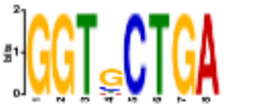 | 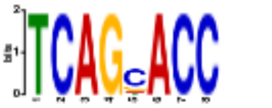 | 3.2e-303 | 3.2e-303 |
| 3. | DTTTCY | 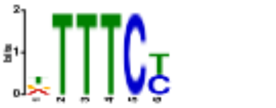 | 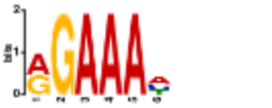 | 9.9e-030 | 1.7e-030 |
| 4. | ACCWTGGABA | 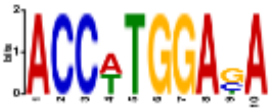 | 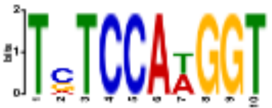 | 5.2e-030 | 7.0e-192 |
| 5. | CAGSACY | 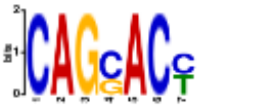 | 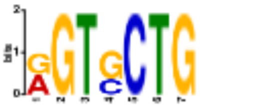 | 7.5e-024 | 6.7e-245 |
| 6. | GGAGCTMTCC | 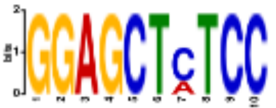 | 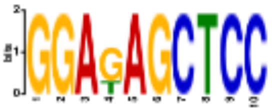 | 2.8e-016 | 1.6e-024 |
| 7. | RGGAAR | 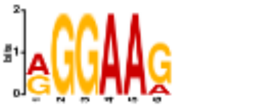 | 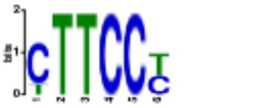 | 4.6e-016 | 5.9e-028 |
| 8. | RTGACTCAB | 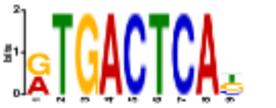 | 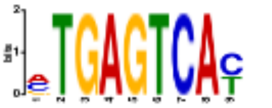 | 5.9e-015 | 2.5e-015 |
| 9. | AWATA | 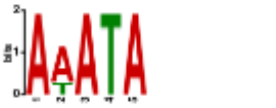 | 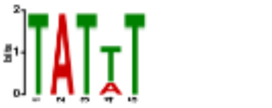 | 2.4e-012 | 1.0e-020 |
| 10. | CCCCDCCCC | 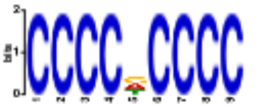 | 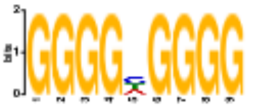 | 1.1e-011 | 3.1e-013 |
| 11. | SCYGGGA | 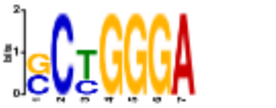 | 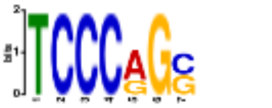 | 1.7e-006 | 2.5e-013 |
| 12. | GCMGCCGCC | 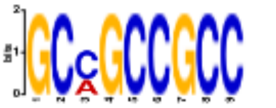 | 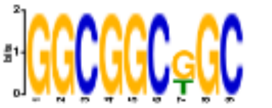 | 1.5e-003 | 1.4e-004 |
| 13. | ACACAMA | 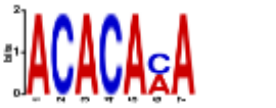 | 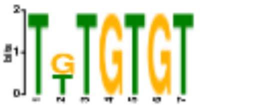 | 1.8e-003 | 1.4e-004 |
| 14. | TGGAGAGCR | 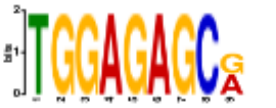 | 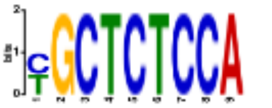 | 5.1e-003 | 1.0e-007 |
| 15. | GGACAGGR | 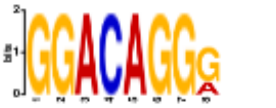 | 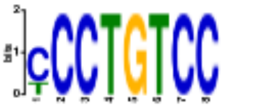 | 5.2e-003 | 1.7e-007 |
| 16. | CASATTCC | 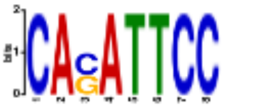 | 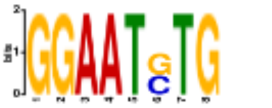 | 4.8e-002 | 2.2e-001 |

## Tcell

| | Motif | Logo | RC Logo | E-value | Unerased E-value |
|---|---|---|---|---|---|
| 1. | GGTNCTGA | 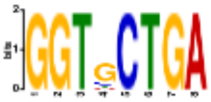 | 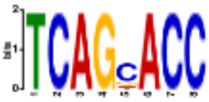 | 2.7e-281 | 2.7e-281 |
| 2. | GGACAGYD | 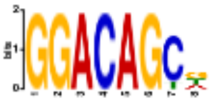 | 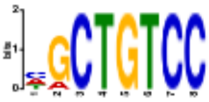 | 3.1e-244 | 1.5e-244 |
| 3. | CDCYTCC | 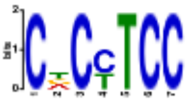 | 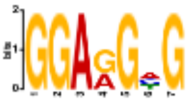 | 6.6e-039 | 6.0e-042 |
| 4. | VGGAAR | 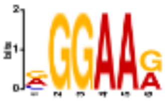 | 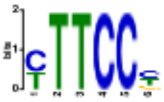 | 2.6e-024 | 6.1e-037 |
| 5. | CAGCACY | 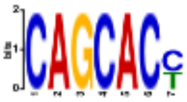 | 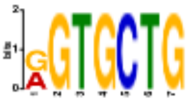 | 1.1e-022 | 3.4e-201 |
| 6. | GGGCGGGR | 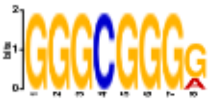 | 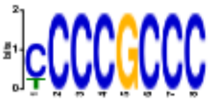 | 2.6e-022 | 6.9e-025 |
| 7. | GCTVTCCA | 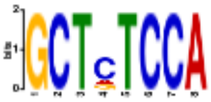 | 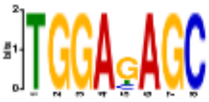 | 1.1e-021 | 1.1e-142 |
| 8. | AKAAA | 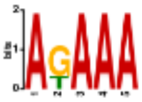 | 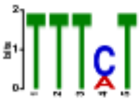 | 6.5e-016 | 2.3e-017 |
| 9. | GCCGCCRYC | 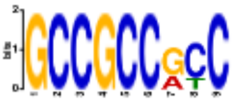 | 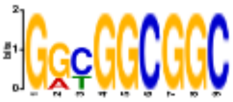 | 6.9e-015 | 2.2e-018 |
| 10. | AHGGACAGA | 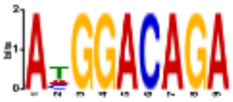 | 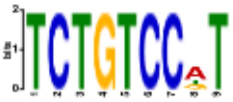 | 6.9e-012 | 4.1e-012 |
| 11. | CGCANGCGCA | 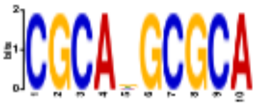 | 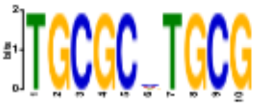 | 2.6e-008 | 1.4e-008 |
| 12. | CRSCTGCAG | 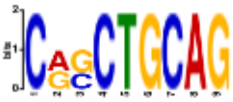 | 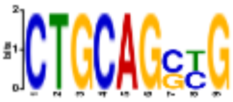 | 2.6e-007 | 1.8e-009 |
| 13. | CDCCGCS | 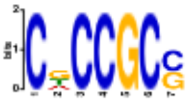 | 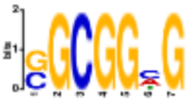 | 1.2e-006 | 8.3e-020 |
| 14. | AAATAH | 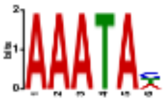 | 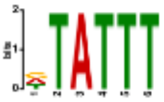 | 8.5e-005 | 1.5e-018 |
| 15. | ACGTGK | 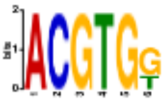 | 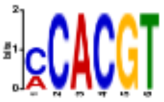 | 1.1e-004 | 2.6e-006 |
| 16. | AGRKGGCG | 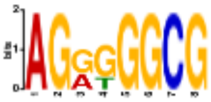 | 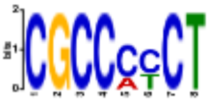 | 1.6e-004 | 4.6e-016 |
| 17. | TGACGTCA | 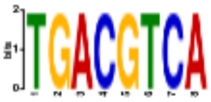 | 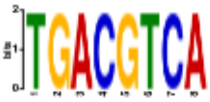 | 2.1e-002 | 5.8e-003 |

U87

| | Motif | Logo | RC Logo | E-value | Unerased E-value |
|---|---|---|---|---|---|
| 1. | DCTGTCC | 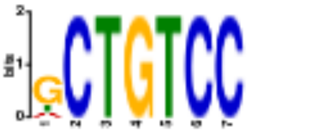 | 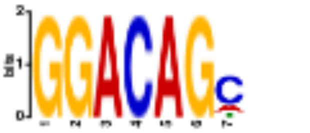 | 8.8e-328 | 8.8e-328 |
| 2. | GGTNCTGA | 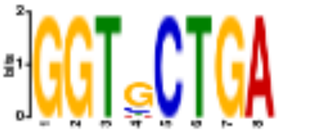 | 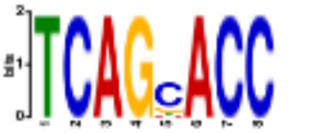 | 3.5e-290 | 3.5e-290 |
| 3. | GGAKAGCKC | 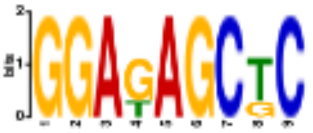 | 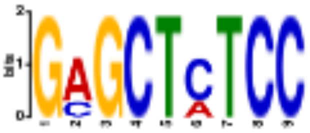 | 1.0e-020 | 5.6e-021 |
| 4. | GTGCTGRA | 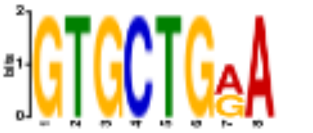 | 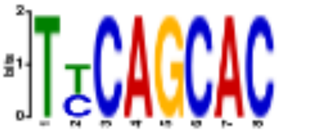 | 4.7e-016 | 1.7e-156 |
| 5. | DAAATA | 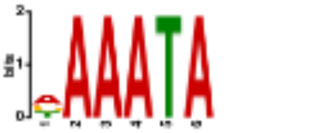 | 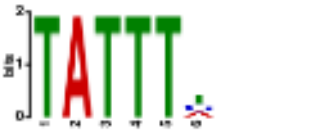 | 2.1e-012 | 2.0e-014 |
| 6. | CHGGRA | 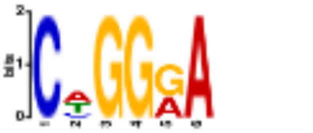 | 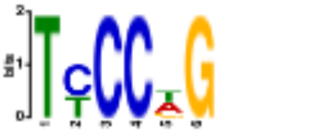 | 7.7e-011 | 1.4e-016 |
| 7. | CAGSACCM | 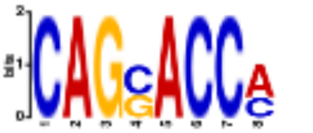 | 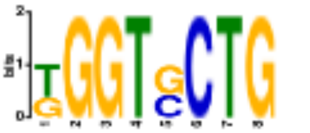 | 3.4e-009 | 1.7e-176 |
| 8. | GGGCGGGR | 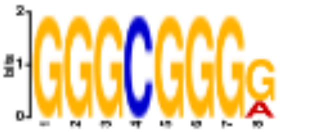 | 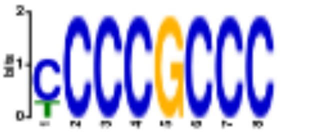 | 1.6e-006 | 6.1e-007 |
| 9. | AGRAA | 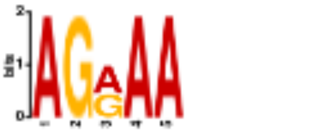 | 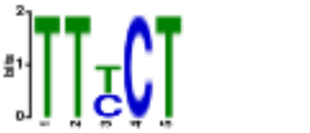 | 6.8e-006 | 1.4e-009 |
| 10. | ATGGAYAG | 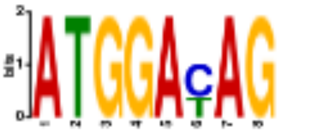 | 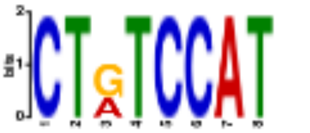 | 5.8e-004 | 8.6e-065 |

## (B) DREME output on dm3 (fly)

### CTCF

| | Motif | Logo | RC Logo | E-value | Unerased E-value |
|---|---|---|---|---|---|
| 1. | AGRKGGCG | | | 3.3e-220 | 3.3e-220 |
| 2. | HATCGATAD | | | 5.9e-038 | 8.1e-039 |
| 3. | CCACY | | | 3.0e-030 | 1.7e-052 |
| 4. | AAAWRTA | | | 2.8e-021 | 7.5e-022 |
| 5. | GTGTGRCCR | | | 1.2e-022 | 1.1e-024 |
| 6. | CAGCTG | | | 1.9e-015 | 6.7e-016 |
| 7. | GAGHGAGA | | | 2.0e-013 | 6.4e-014 |
| 8. | TTCTACAB | | | 1.1e-012 | 3.2e-014 |
| 9. | TCGAWW | | | 4.7e-011 | 1.2e-022 |
| 10. | RAAAGCTY | | | 8.2e-011 | 3.1e-011 |
| 11. | ACTCVAACC | | | 1.8e-009 | 6.8e-009 |
| 12. | AGRTGTCGC | | | 1.8e-009 | 1.4e-010 |
| 13. | GYTGCCA | | | 2.5e-009 | 5.3e-017 |
| 14. | ACACAHAC | | | 5.0e-008 | 5.3e-009 |
| 15. | AWATRT | | | 7.0e-007 | 2.2e-019 |
| 16. | ACTGCTTRC | | | 1.0e-005 | 1.7e-005 |
| 17. | ARCAACAAC | | | 3.2e-005 | 6.0e-005 |
| 18. | WTTTCCR | | | 2.4e-005 | 2.8e-009 |
| 19. | CBCTCTC | | | 2.3e-004 | 6.5e-013 |
| 20. | TTTRTTTW | | | 1.1e-003 | 8.9e-006 |
| 21. | CTGGCBG | | | 3.1e-002 | 1.0e-015 |
| 22. | CCGMTAGA | | | 3.6e-002 | 4.2e-010 |

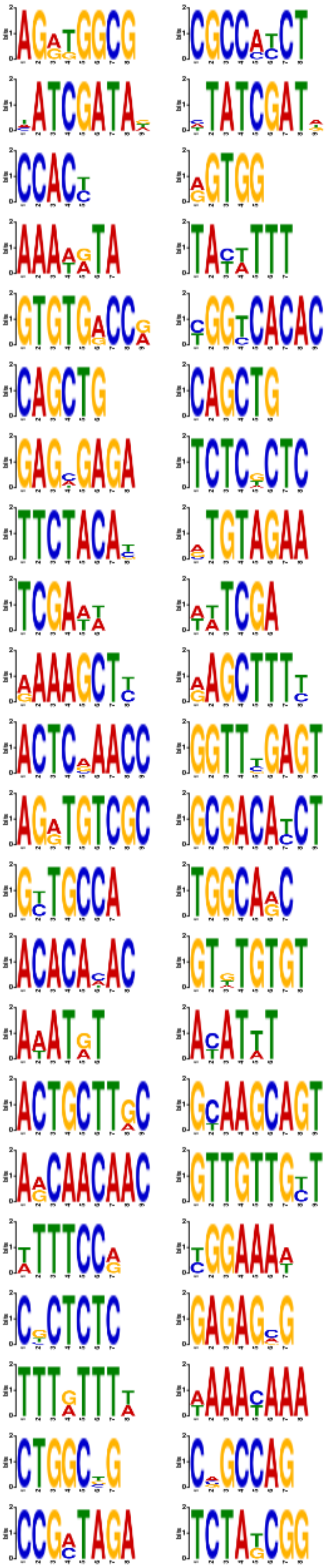

# SU(HW)

| | Motif | Logo | RC Logo | E-value | Unerased E-value |
|---|---|---|---|---|---|
| 1. | AAGTAKRC | | | 7.8e-354 | 7.8e-354 |
| 2. | CRCMCAC | | | 6.3e-076 | 6.3e-076 |
| 3. | ATAHATW | | | 3.5e-045 | 3.5e-045 |
| 4. | SGAAAW | | | 4.5e-031 | 1.8e-034 |
| 5. | CWGCTSC | | | 4.4e-029 | 1.7e-030 |
| 6. | CGASTS | | | 6.5e-021 | 2.0e-027 |
| 7. | AAARTRT | | | 7.8e-021 | 5.4e-136 |
| 8. | CKCCHC | | | 4.1e-012 | 9.4e-019 |
| 9. | GTATGCWA | | | 2.7e-011 | 1.3e-219 |
| 10. | TGGCCA | | | 9.5e-011 | 4.2e-010 |
| 11. | AAATATK | | | 1.1e-008 | 1.1e-028 |
| 12. | CTTTTRGGC | | | 5.0e-008 | 2.0e-033 |
| 13. | CWCTC | | | 9.4e-008 | 1.1e-025 |
| 14. | CMAAAAAAAAAA | | | 6.5e-007 | 1.1e-006 |
| 15. | GCCADCGA | | | 1.4e-006 | 3.0e-009 |
| 16. | GKCGMC | | | 3.6e-006 | 9.5e-018 |
| 17. | ACACAKAY | | | 1.8e-006 | 1.9e-022 |
| 18. | AATKAAA | | | 5.6e-006 | 9.9e-012 |
| 19. | GTCCTKS | | | 5.9e-005 | 1.1e-010 |
| 20. | ACTTTY | | | 1.0e-003 | 1.1e-096 |
| 21. | AAYTATGC | | | 1.2e-003 | 1.2e-005 |
| 22. | AAYAACAA | | | 2.1e-003 | 1.0e-002 |
| 23. | GGGGGGGGGGK | | | 1.4e-002 | 8.3e-004 |
| 24. | AGBCGAA | | | 1.6e-002 | 4.1e-008 |
| 25. | CGYATACGC | | | 1.8e-002 | 5.2e-004 |

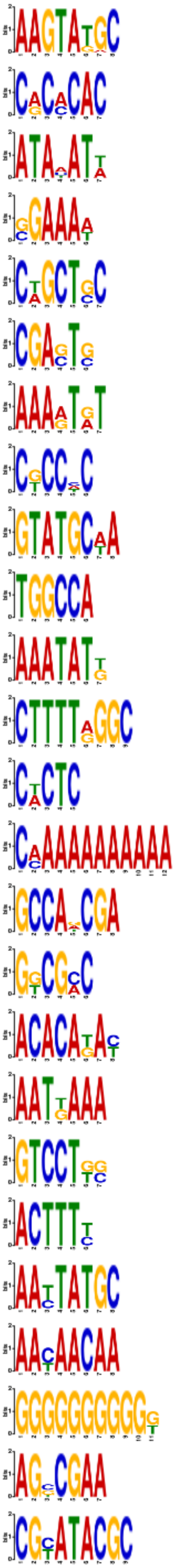

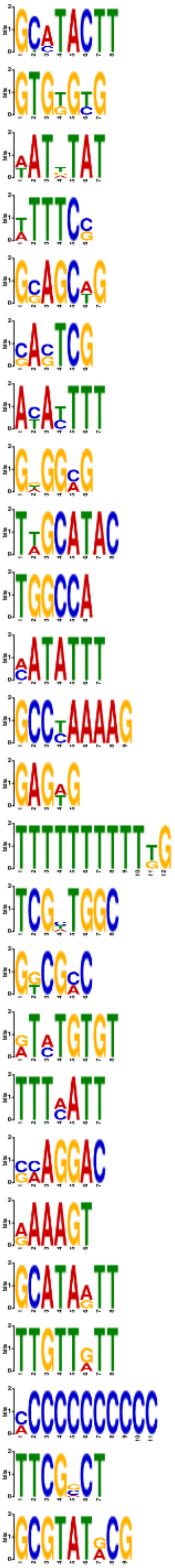

Pita

| | Motif | Logo | RC Logo | E-value | Unerased E-value |
|---|---|---|---|---|---|
| 1. | CWCVAACC | 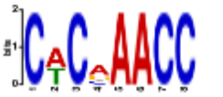 | 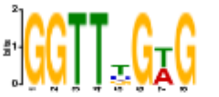 | 2.2e-039 | 2.2e-039 |
| 2. | AGRTGKCG | 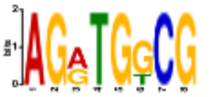 | 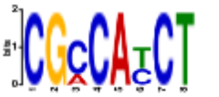 | 2.1e-035 | 2.1e-035 |
| 3. | RTGTARAA | 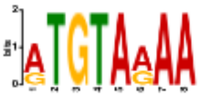 | 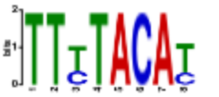 | 1.1e-019 | 1.1e-019 |
| 4. | AAAGTAKGC | 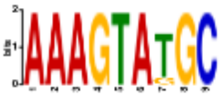 | 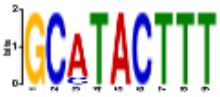 | 2.2e-018 | 2.2e-018 |
| 5. | AGCVRAG | 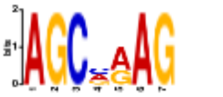 | 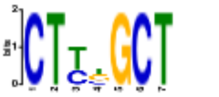 | 3.8e-015 | 3.8e-015 |
| 6. | ABCGATA | 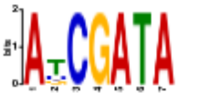 | 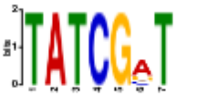 | 4.3e-014 | 4.3e-014 |
| 7. | AAATAWAY | 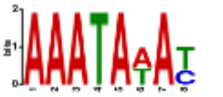 | 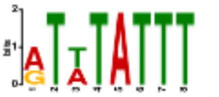 | 5.8e-013 | 4.6e-014 |
| 8. | AGKGYTGC | 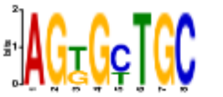 | 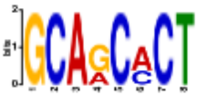 | 1.7e-011 | 2.0e-012 |
| 9. | CAGTGK | 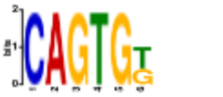 | 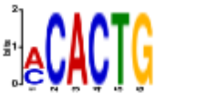 | 5.8e-009 | 4.8e-010 |
| 10. | AHATGTA | 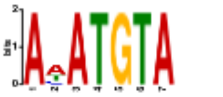 | 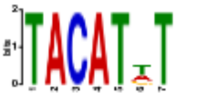 | 2.0e-008 | 3.9e-016 |
| 11. | TSGAAAW | 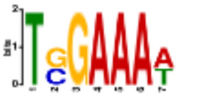 | 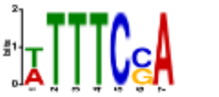 | 1.1e-005 | 1.1e-007 |
| 12. | AGAGRGCG | 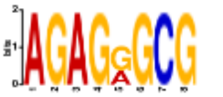 | 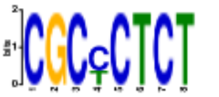 | 2.0e-005 | 7.7e-007 |
| 13. | CACNCAC | 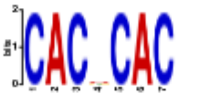 | 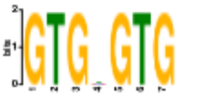 | 3.4e-005 | 9.3e-007 |
| 14. | ACTGCTTGC | 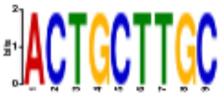 | 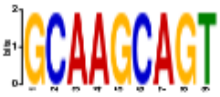 | 1.8e-003 | 1.4e-004 |
| 15. | TGTGACCR | 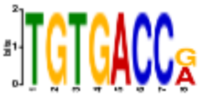 | 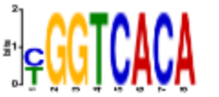 | 1.1e-002 | 3.9e-008 |
| 16. | GCGAGTR | 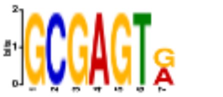 | 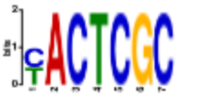 | 1.6e-002 | 1.4e-003 |
| 17. | DAAAATA | 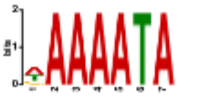 | 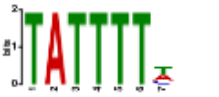 | 3.2e-002 | 1.3e-010 |
| 18. | CAACAACC | 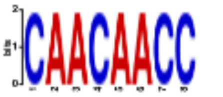 | 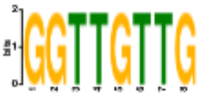 | 3.3e-002 | 1.8e-002 |

(C) DREME output on K562 cell-line

## FOSL1

| | Motif | Logo | RC Logo | E-value | Unerased E-value |
|---|---|---|---|---|---|
| 1. | TKAGTCAB | 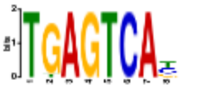 | 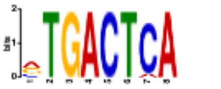 | 3.4e-1400 | 3.4e-1400 |
| 2. | TTATCW | 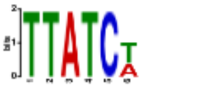 | 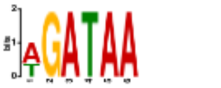 | 2.4e-112 | 5.3e-112 |
| 3. | CCMCDCCC | 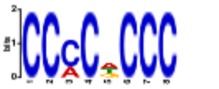 | 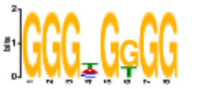 | 1.5e-060 | 4.3e-061 |
| 4. | MGGAAR | 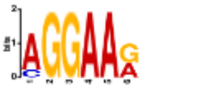 | 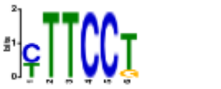 | 8.3e-051 | 1.2e-058 |
| 5. | RTGADTCA | 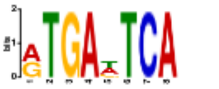 | 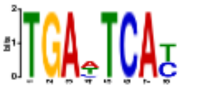 | 7.3e-030 | 1.9e-917 |
| 6. | DAAAYA | 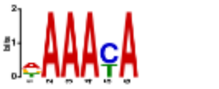 | 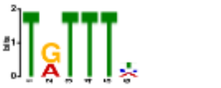 | 5.0e-029 | 1.3e-038 |
| 7. | CVCAGC | 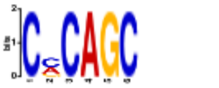 | 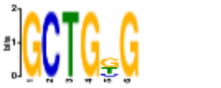 | 2.3e-022 | 6.4e-032 |
| 8. | CCKCCC | 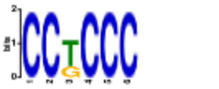 | 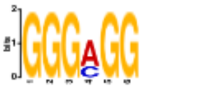 | 1.8e-012 | 1.9e-033 |
| 9. | RTGAGTAA | 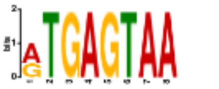 | 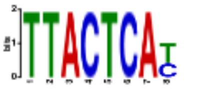 | 6.8e-010 | 6.8e-010 |
| 10. | TTWTAWA | 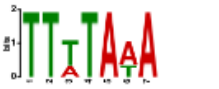 | 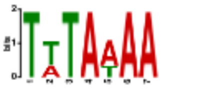 | 3.2e-009 | 2.7e-015 |
| 11. | CDCTAGGACC | 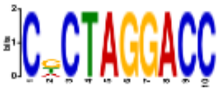 | 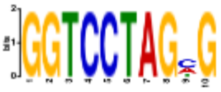 | 9.6e-009 | 7.0e-010 |
| 12. | CRGCAGC | 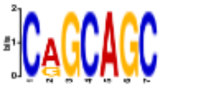 | 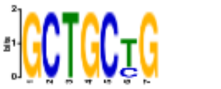 | 2.0e-007 | 8.3e-017 |
| 13. | GGAAA | 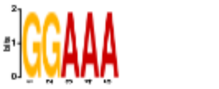 | 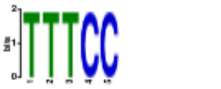 | 2.4e-007 | 1.8e-038 |
| 14. | ACACACVC | 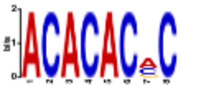 | 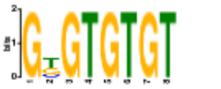 | 1.4e-006 | 1.1e-008 |
| 15. | AAAAARAAA | 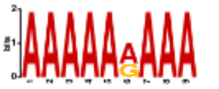 | 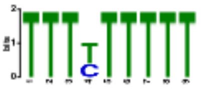 | 1.9e-006 | 3.7e-010 |
| 16. | AGGCNG | 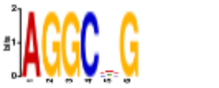 | 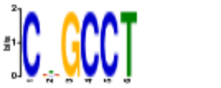 | 9.2e-006 | 3.8e-020 |
| 17. | GRTGACGY | 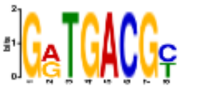 | 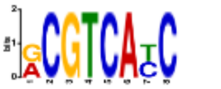 | 8.3e-006 | 6.9e-007 |
| 18. | CACGTRK | 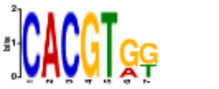 | 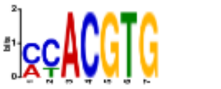 | 3.8e-005 | 1.8e-009 |
| 19. | ARCCACA | 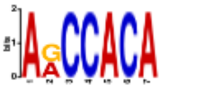 | 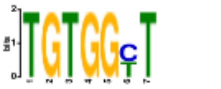 | 7.5e-005 | 1.2e-011 |
| 20. | CCAMCCCCA | 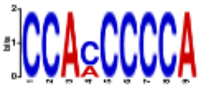 | 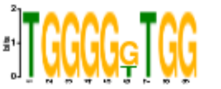 | 4.2e-002 | 8.3e-013 |

FOXA1

| | Motif | Logo | RC Logo | E-value | Unerased E-value |
|---|---|---|---|---|---|
| 1. | GYAAAYA | 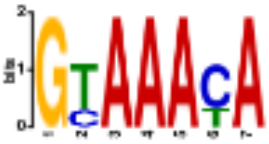 | 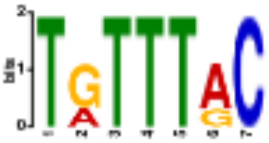 | 4.6e-063 | 4.6e-063 |
| 2. | BTTATCW | 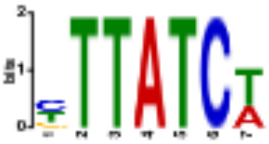 | 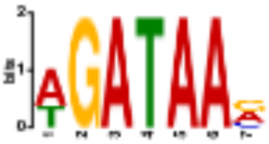 | 7.4e-030 | 1.7e-030 |
| 3. | RTGACTCA | 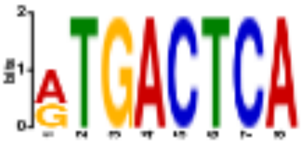 | 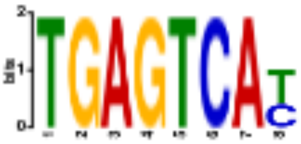 | 2.4e-011 | 2.4e-011 |
| 4. | TGTCAAYA | 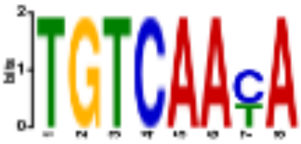 | 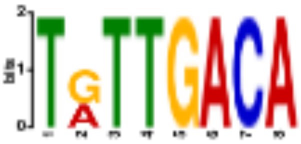 | 5.7e-003 | 3.0e-003 |

# GATA1

| | Motif | Logo | RC Logo | E-value | Unerased E-value |
|---|---|---|---|---|---|
| 1. | HGATAA | 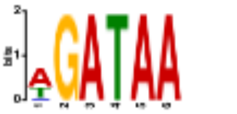 | 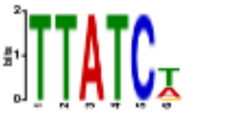 | 3.7e-545 | 3.7e-545 |
| 2. | CCMCRCCC | 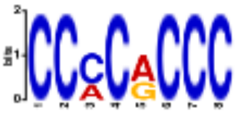 | 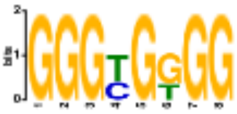 | 8.7e-029 | 8.7e-029 |
| 3. | AGATAGB | 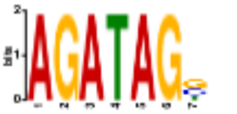 | 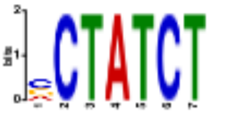 | 1.9e-027 | 2.7e-028 |
| 4. | CMCAGSC | 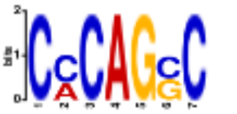 | 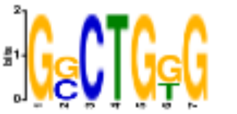 | 5.2e-015 | 3.7e-016 |
| 5. | CWGCWG | 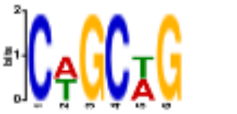 | 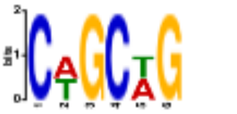 | 6.8e-013 | 7.7e-018 |
| 6. | CAGRAA | 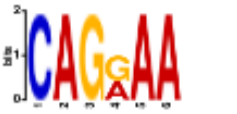 | 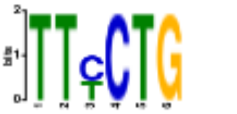 | 1.1e-010 | 7.4e-013 |
| 7. | TGAGTCAB | 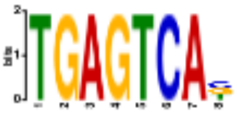 | 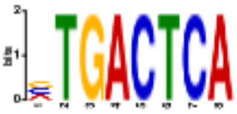 | 3.3e-009 | 2.3e-012 |
| 8. | CASMCAC | 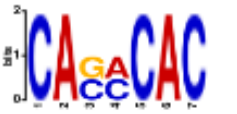 | 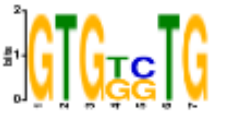 | 1.4e-008 | 1.1e-014 |
| 9. | DAAAYA | 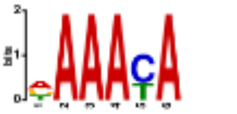 | 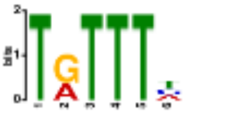 | 9.6e-008 | 2.7e-014 |
| 10. | CTCCYKCC | 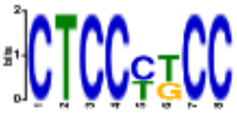 | 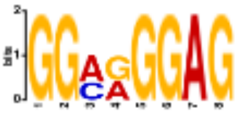 | 2.6e-007 | 1.0e-010 |
| 11. | SWGATTA | 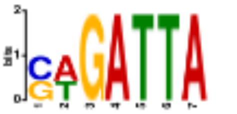 | 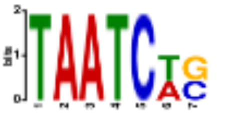 | 8.4e-006 | 1.8e-008 |
| 12. | CGTGK | 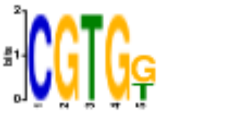 | 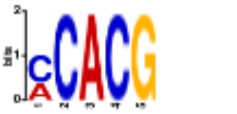 | 8.0e-005 | 8.0e-010 |
| 13. | CWGATA | 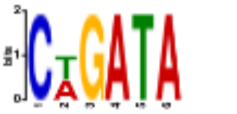 | 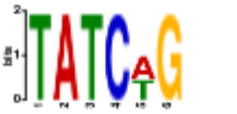 | 3.3e-004 | 2.8e-180 |
| 14. | AAACCRCA | 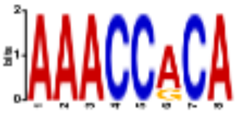 | 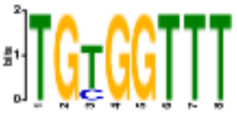 | 7.3e-003 | 7.5e-006 |
| 15. | GTCAC | 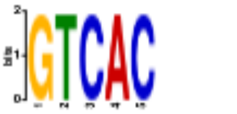 | 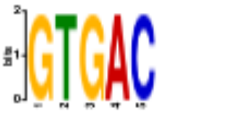 | 4.1e-002 | 1.5e-008 |

# GATA2

| | Motif | Logo | RC Logo | E-value | Unerased E-value |
|---|---|---|---|---|---|
| 1. | HGATAA | 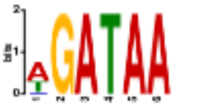 | 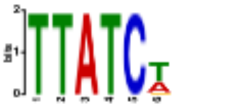 | 4.3e-665 | 4.3e-665 |
| 2. | SWGATA | 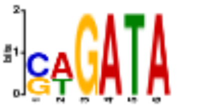 | 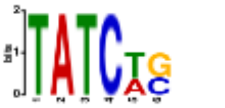 | 2.1e-043 | 1.7e-391 |
| 3. | TGAGTCAB | 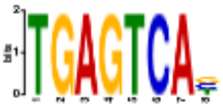 | 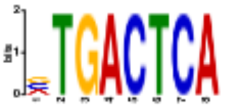 | 2.3e-032 | 8.4e-033 |
| 4. | AMACAS | 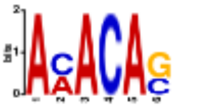 | 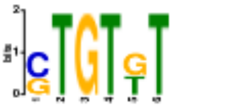 | 2.5e-029 | 1.9e-036 |
| 5. | CCMCRCCC | 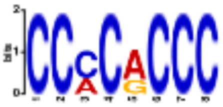 | 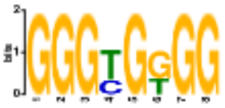 | 1.9e-027 | 1.3e-027 |
| 6. | RVAGGAA | 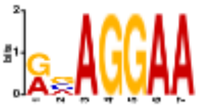 | 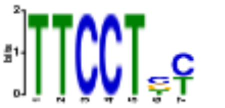 | 2.1e-022 | 1.4e-027 |
| 7. | CHGCWG | 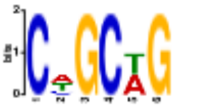 | 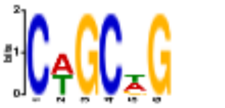 | 2.8e-017 | 2.3e-031 |
| 8. | CMCWGCC | 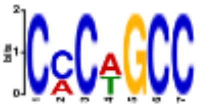 | 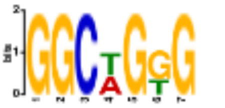 | 4.7e-014 | 7.1e-020 |
| 9. | CCYTYCC | 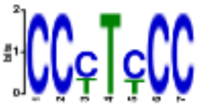 | 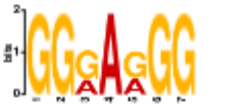 | 2.8e-011 | 6.2e-020 |
| 10. | SWGATTA | 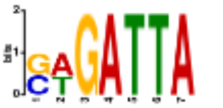 | 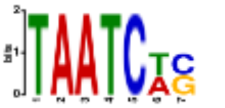 | 6.7e-011 | 9.7e-015 |
| 11. | TATYTW | 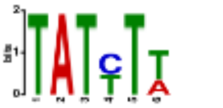 | 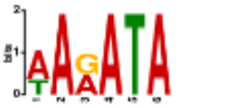 | 1.2e-010 | 9.0e-087 |
| 12. | MCACGY | 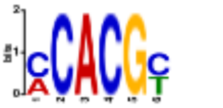 | 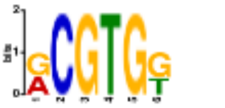 | 5.5e-008 | 5.0e-014 |
| 13. | KTTTCY | 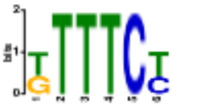 | 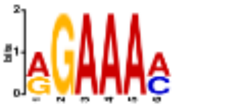 | 2.5e-007 | 9.3e-022 |
| 14. | CTCCWSCC | 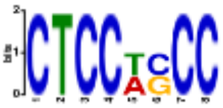 | 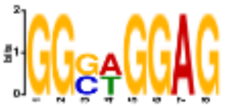 | 9.6e-004 | 4.7e-009 |
| 15. | RRCCACA | 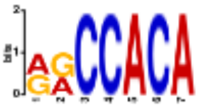 | 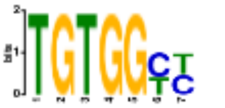 | 1.1e-003 | 2.1e-010 |
| 16. | CATCTGB | 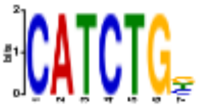 | 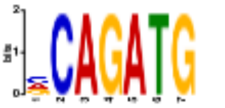 | 6.6e-003 | 5.1e-009 |

# IRF2

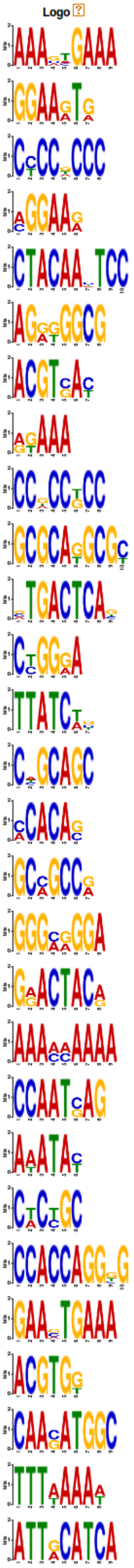

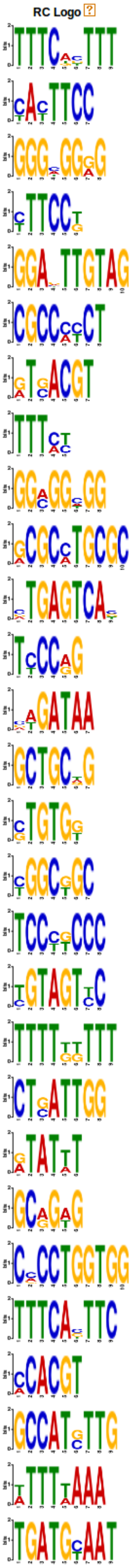

| | Motif | E-value | Unerased E-value |
|---|---|---|---|
| 1. | AAAVHGAAA | 7.3e-318 | 7.3e-318 |
| 2. | GGAARTR | 9.5e-103 | 6.1e-103 |
| 3. | CYCCDCCC | 1.8e-097 | 1.4e-100 |
| 4. | MGGAAR | 5.0e-061 | 6.6e-103 |
| 5. | CTACAANTCC | 1.1e-055 | 1.1e-055 |
| 6. | AGRKGGCG | 4.4e-045 | 1.0e-057 |
| 7. | ACGTSAY | 1.4e-034 | 5.5e-039 |
| 8. | RKAAA | 6.2e-031 | 1.6e-063 |
| 9. | CCDCCKCC | 2.5e-030 | 1.0e-047 |
| 10. | GCGCAKGCGY | 8.2e-030 | 3.5e-031 |
| 11. | VTGACTCAB | 1.4e-028 | 2.1e-030 |
| 12. | CYGGRA | 7.7e-025 | 2.6e-059 |
| 13. | TTATCWB | 2.9e-017 | 1.4e-026 |
| 14. | CHGCAGC | 4.1e-017 | 2.6e-019 |
| 15. | MCACAS | 2.4e-010 | 4.8e-016 |
| 16. | GCMGCCR | 1.7e-009 | 2.1e-028 |
| 17. | GGGMRGGA | 1.3e-009 | 3.3e-025 |
| 18. | GRACTACR | 4.7e-009 | 6.5e-037 |
| 19. | AAAMMAAAA | 2.6e-007 | 3.9e-024 |
| 20. | CCAATSAG | 5.1e-007 | 5.9e-010 |
| 21. | AWATAY | 2.0e-005 | 1.5e-015 |
| 22. | CWCYGC | 4.5e-005 | 1.9e-021 |
| 23. | CCACCAGGBG | 1.0e-003 | 1.4e-007 |
| 24. | GAAVTGAAA | 1.8e-003 | 1.2e-026 |
| 25. | ACGTGK | 1.9e-003 | 5.7e-012 |
| 26. | CAASATGGC | 8.1e-003 | 3.9e-019 |
| 27. | TTTWAAAW | 9.3e-003 | 2.0e-008 |
| 28. | ATTRCATCA | 2.9e-002 | 1.4e-002 |

# JUNB

| | Motif | Logo | RC Logo | E-value | Unerased E-value |
|---|---|---|---|---|---|
| **1.** | RTGASTCAB | 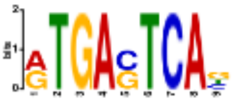 | 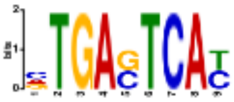 | 4.9e-361 | 4.9e-361 |
| **2.** | BTTATCW | 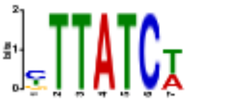 | 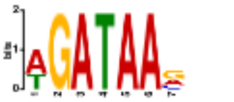 | 2.3e-043 | 1.4e-043 |
| **3.** | AGGAARB | 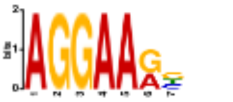 | 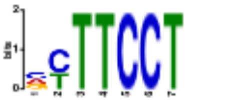 | 1.3e-028 | 8.1e-033 |
| **4.** | CCCCDCCCC | 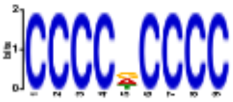 | 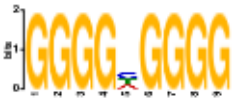 | 2.3e-024 | 1.3e-024 |
| **5.** | BTWAGGGA | 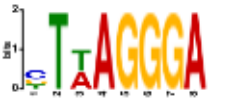 | 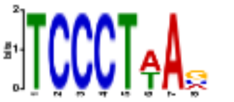 | 1.0e-020 | 2.9e-021 |
| **6.** | CMGGRA | 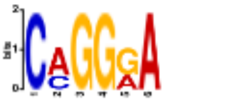 | 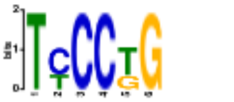 | 4.6e-011 | 1.7e-022 |
| **7.** | TGACTMA | 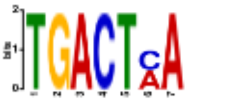 | 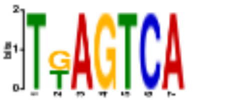 | 8.3e-010 | 3.9e-323 |
| **8.** | CVCMGCC | 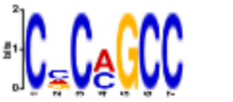 | 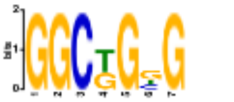 | 2.8e-009 | 6.3e-017 |
| **9.** | AMMCACA | 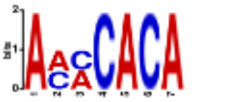 | 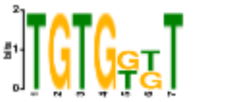 | 1.6e-009 | 1.7e-013 |
| **10.** | DAAATR | 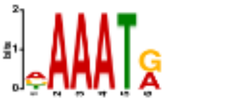 | 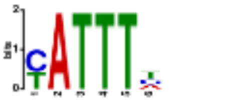 | 1.5e-008 | 4.2e-019 |
| **11.** | CHGCAGC | 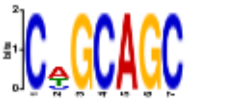 | 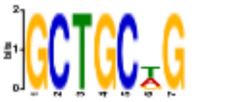 | 3.9e-007 | 1.8e-011 |
| **12.** | TGABGTCA | 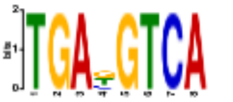 | 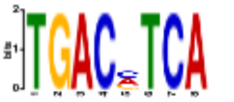 | 2.7e-005 | 3.1e-006 |
| **13.** | CYTCCTCC | 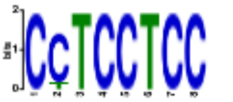 | 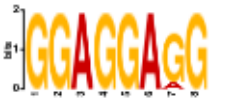 | 1.6e-003 | 6.9e-009 |
| **14.** | ATGAWTCA | 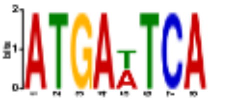 | 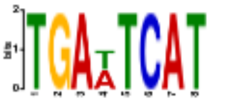 | 9.5e-003 | 7.7e-004 |
| **15.** | AGGGACYG | 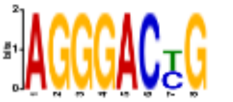 | 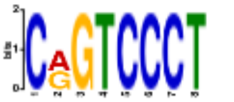 | 1.1e-002 | 1.8e-014 |
| **16.** | CAGCYCTG | 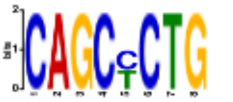 | 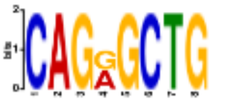 | 1.2e-002 | 2.3e-005 |
| **17.** | TGTAMACA | 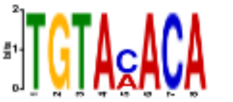 | 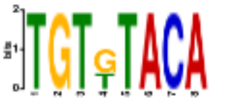 | 1.5e-002 | 7.2e-002 |

## RUNX1

| Motif | | Logo | RC Logo | E-value | Unerased E-value |
|---|---|---|---|---|---|
| **1.** | RACCRCA | 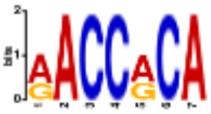 | 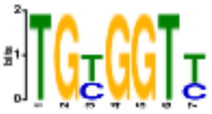 | 1.6e-344 | 1.6e-344 |
| **2.** | TTATCW | 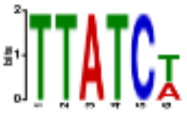 | 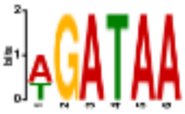 | 2.4e-061 | 4.9e-064 |
| **3.** | VGGAAR | 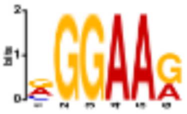 | 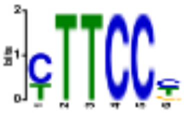 | 1.7e-039 | 3.0e-043 |
| **4.** | CHCCKCCC | 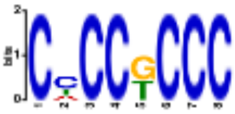 | 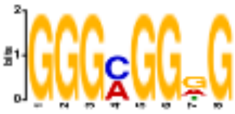 | 1.8e-028 | 9.4e-032 |
| **5.** | TGAGTCAB | 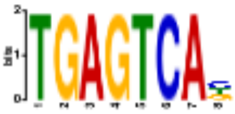 | 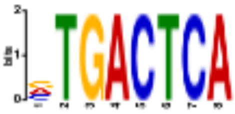 | 7.1e-016 | 4.3e-016 |
| **6.** | CCGCCKCY | 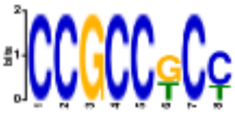 | 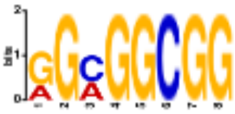 | 9.5e-012 | 2.4e-014 |
| **7.** | CCRCAGM | 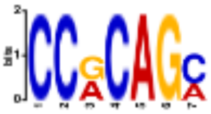 | 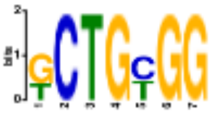 | 2.8e-013 | 1.1e-071 |
| **8.** | GCGCABGCGC | 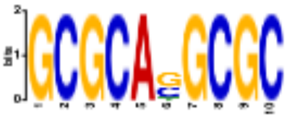 | 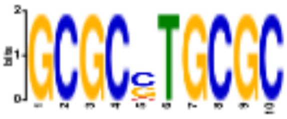 | 9.0e-009 | 3.4e-009 |
| **9.** | TGACBTCA | 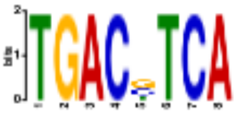 | 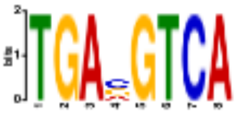 | 3.1e-008 | 3.0e-008 |
| **10.** | CCMCACCC | 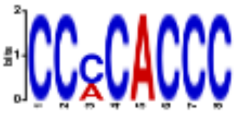 | 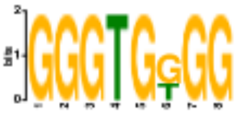 | 6.9e-006 | 1.6e-008 |
| **11.** | BCACGTGAC | 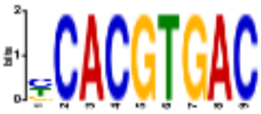 | 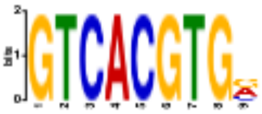 | 9.7e-005 | 1.1e-006 |
| **12.** | GTGGTTW | 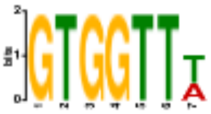 | 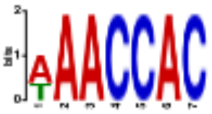 | 2.3e-004 | 5.2e-129 |
| **13.** | ARATA | 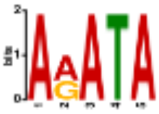 | 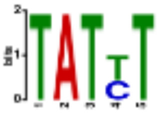 | 3.0e-004 | 5.6e-026 |
| **14.** | GSCGGGA | 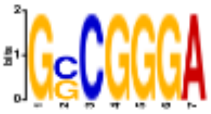 | 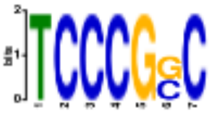 | 5.2e-003 | 1.4e-008 |
| **15.** | ACCRCA | 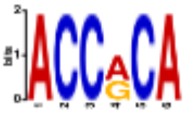 | 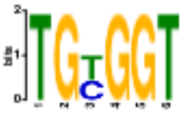 | 2.3e-002 | 1.6e-317 |

# P300

| | Motif | Logo | RC Logo | E-value | Unerased E-value |
|---|---|---|---|---|---|
| 1. | BTTATCW | | | 3.1e-1034 | 3.1e-1034 |
| 2. | TGAGTCAB | | | 3.6e-408 | 1.2e-409 |
| 3. | CHCCDCCC | | | 2.3e-296 | 1.6e-300 |
| 4. | MGGAAR | | | 6.9e-287 | 1.4e-313 |
| 5. | ARATA | | | 4.1e-110 | 2.1e-398 |
| 6. | AMACAS | | | 1.4e-080 | 9.1e-115 |
| 7. | RTTDCATCA | | | 1.3e-081 | 1.8e-084 |
| 8. | ACGTSAY | | | 1.4e-071 | 3.3e-086 |
| 9. | CWGCWG | | | 4.0e-058 | 5.4e-094 |
| 10. | DTTTCY | | | 2.8e-057 | 6.0e-173 |
| 11. | CCACRYCC | | | 5.7e-050 | 6.5e-059 |
| 12. | GGGMGGGA | | | 1.5e-039 | 5.9e-059 |
| 13. | HGTCAY | | | 8.2e-038 | 7.1e-204 |
| 14. | CCKCCKCC | | | 1.5e-035 | 3.7e-082 |
| 15. | TTATCR | | | 4.6e-032 | 2.8e-316 |
| 16. | GCGCANGCGC | | | 4.3e-031 | 1.5e-032 |
| 17. | AVCCACA | | | 1.1e-029 | 2.1e-069 |
| 18. | AAAAAAAAAAR | | | 3.2e-027 | 6.7e-041 |
| 19. | AGRKGGCG | | | 5.0e-028 | 7.3e-069 |
| 20. | CYTYCC | | | 6.0e-027 | 1.3e-141 |
| 21. | TTCYARGAA | | | 4.0e-022 | 1.1e-080 |
| 22. | ACGTGK | | | 3.8e-018 | 3.4e-045 |
| 23. | CMCAGSC | | | 1.2e-016 | 7.9e-060 |
| 24. | TTTWAAAW | | | 1.8e-010 | 2.5e-024 |
| 25. | CHGCS | | | 1.5e-011 | 1.5e-048 |
| 26. | GCTGADTCA | | | 1.4e-008 | 7.6e-120 |
| 27. | GWAAACA | | | 5.9e-008 | 3.3e-060 |
| 28. | AGATTAB | | | 7.0e-008 | 4.9e-019 |
| 29. | STGACCWCA | | | 6.1e-008 | 4.4e-018 |
| 30. | TTCTGRGAA | | | 4.3e-005 | 1.6e-026 |
| 31. | TTACTCAB | | | 7.6e-005 | 2.4e-011 |
| 32. | CTACAABTCCC | | | 8.6e-005 | 5.1e-008 |
| 33. | GGAAA | | | 1.7e-004 | 4.2e-160 |
| 34. | CCACYAGGKGG | | | 9.5e-008 | 8.2e-025 |
| 35. | CTGATAGS | | | 3.0e-004 | 2.9e-008 |
| 36. | AAAYGT | | | 3.3e-004 | 4.5e-043 |
| 37. | CASATG | | | 4.8e-004 | 1.3e-024 |
| 38. | CTGTGGCCW | | | 2.2e-004 | 1.5e-013 |
| 39. | AAAACAARA | | | 1.5e-003 | 1.1e-009 |
| 40. | GTGGTTW | | | 1.8e-003 | 7.7e-053 |
| 41. | CAGAGGYCA | | | 6.0e-003 | 9.7e-009 |
| 42. | RCCGTTA | | | 2.0e-002 | 5.6e-010 |
| 43. | CTCCCACDC | | | 4.1e-002 | 1.0e-026 |

# THAP1

| | Motif | Logo | RC Logo | E-value | Unerased E-value |
|---|---|---|---|---|---|
| 1. | ACTTCCGB | 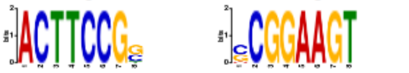 | | 2.9e-091 | 2.9e-091 |
| 2. | AANATGGC | 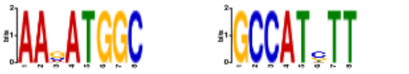 | | 3.8e-080 | 3.8e-080 |
| 3. | GGGCGGRR | 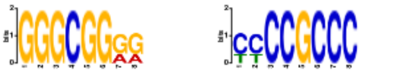 | | 1.3e-076 | 3.8e-087 |
| 4. | ACGTSAC | 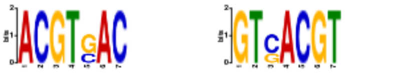 | | 7.2e-058 | 1.3e-060 |
| 5. | VGGAAR | 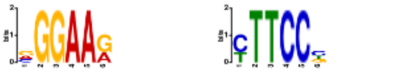 | | 5.5e-054 | 4.6e-080 |
| 6. | GCGCABGCGCR | 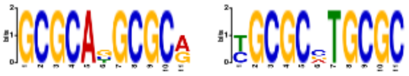 | | 4.9e-055 | 1.3e-055 |
| 7. | CDCCKCC | 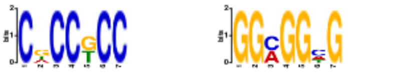 | | 6.0e-032 | 3.1e-046 |
| 8. | CYGGRA | 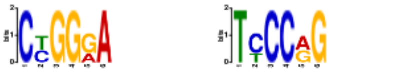 | | 1.0e-021 | 2.2e-066 |
| 9. | ARCCAAT | 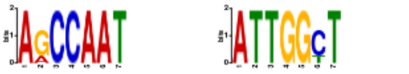 | | 8.8e-021 | 2.7e-021 |
| 10. | RACTACA | 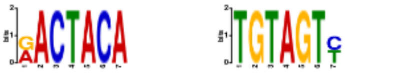 | | 7.1e-014 | 1.5e-017 |
| 11. | GCGCATGCK | 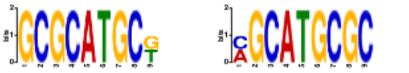 | | 7.6e-011 | 3.0e-027 |
| 12. | CHGCRGC | 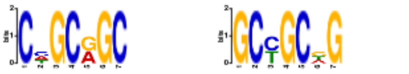 | | 5.2e-011 | 7.9e-018 |
| 13. | GAMGCCR | 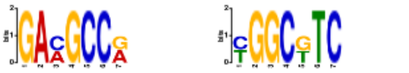 | | 3.0e-010 | 1.0e-028 |
| 14. | DATAAA | 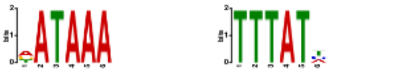 | | 4.1e-008 | 1.1e-009 |
| 15. | CCACGTR | 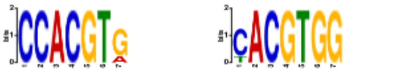 | | 2.4e-007 | 2.4e-024 |
| 16. | ATRGCAAC | 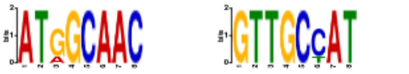 | | 1.8e-006 | 2.8e-008 |
| 17. | TCTCGCGAKA | 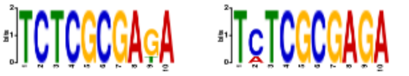 | | 3.6e-006 | 9.0e-007 |
| 18. | RTGACKCA | 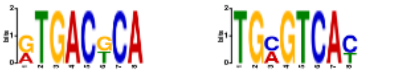 | | 7.3e-006 | 9.5e-012 |
| 19. | CCCCWCCC | 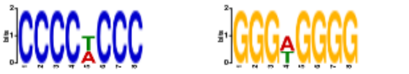 | | 1.6e-004 | 4.9e-010 |
| 20. | GCGCBTGCGCA | 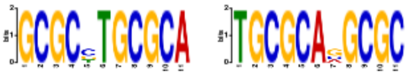 | | 5.4e-003 | 6.3e-023 |
| 21. | GGGCGTGGCB | 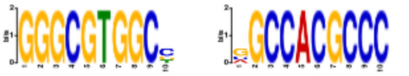 | | 8.5e-003 | 7.9e-006 |
| 22. | TCABGTGA | 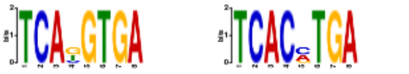 | | 2.6e-002 | 4.3e-017 |
| 23. | ATTGGYCG | 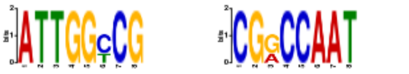 | | 2.9e-002 | 5.6e-003 |
| 24. | AGAAAM | 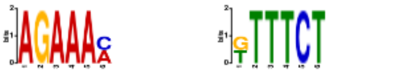 | | 3.5e-002 | 1.2e-005 |
| 25. | GGTGCTGAM | 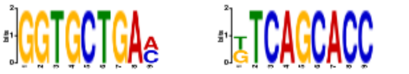 | | 3.5e-002 | 5.3e-005 |

USF1

| | Motif | Logo | RC Logo | E-value | Unerased E-value |
|---|---|---|---|---|---|
| 1. | CACRTGD | 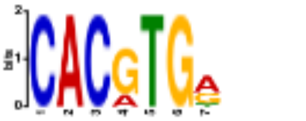 | 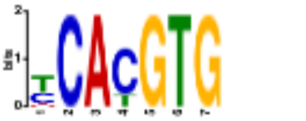 | 1.2e-531 | 1.2e-531 |
| 2. | CYCCGCCCH | 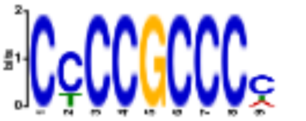 | 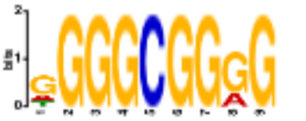 | 2.7e-049 | 8.0e-051 |
| 3. | VGGAAR | 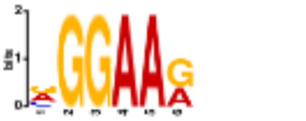 | 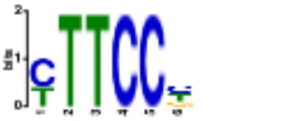 | 7.2e-027 | 7.7e-029 |
| 4. | AAVATGGCG | 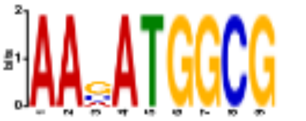 | 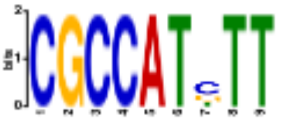 | 7.5e-017 | 2.1e-018 |
| 5. | GCGCANGCGC | 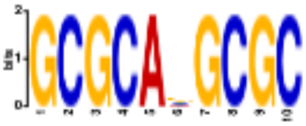 | 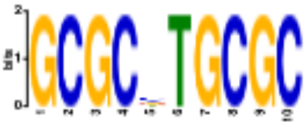 | 4.6e-015 | 2.5e-015 |
| 6. | CCKCCTCY | 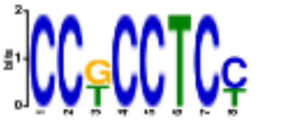 | 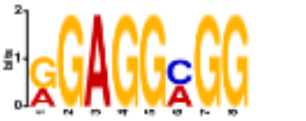 | 3.1e-013 | 1.9e-017 |
| 7. | RGCCAATS | 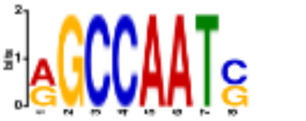 | 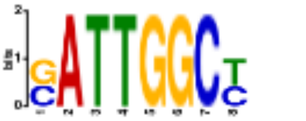 | 1.0e-012 | 2.5e-012 |
| 8. | DAAATA | 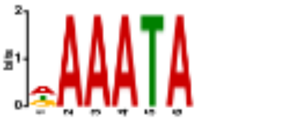 | 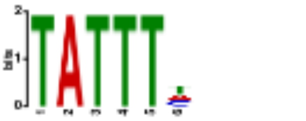 | 5.0e-008 | 3.3e-010 |
| 9. | CCCDCCCC | 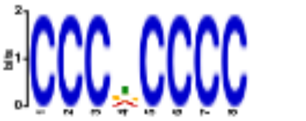 | 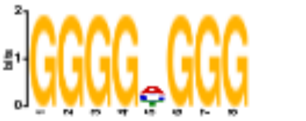 | 9.0e-007 | 3.0e-030 |
| 10. | GMCGTCAC | 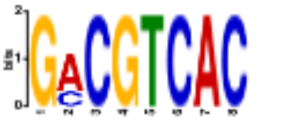 | 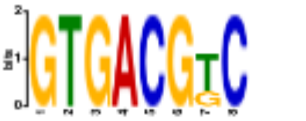 | 4.4e-006 | 7.3e-016 |
| 11. | CAGCCW | 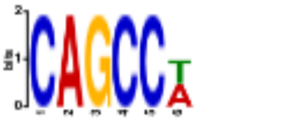 | 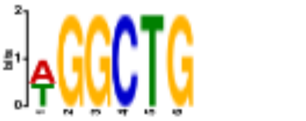 | 2.7e-004 | 6.6e-009 |
| 12. | RGAAA | 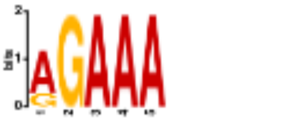 | 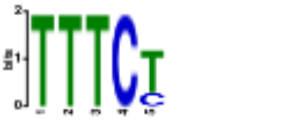 | 1.6e-003 | 1.8e-013 |
| 13. | CCTCCCDC | 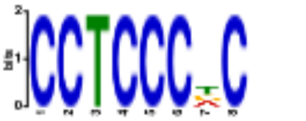 | 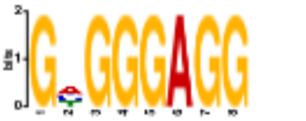 | 2.2e-002 | 1.5e-011 |
| 14. | CAKGTGAC | 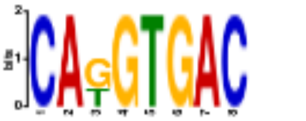 | 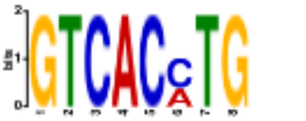 | 4.1e-002 | 8.8e-043 |
