# Supplementary material for: DIVERSITY in binding, regulation, and evolution revealed from high-throughput ChIP
Source: PLoS Comput Biol. 2018 Apr 23;14(4):e1006090. doi: 10.1371/journal.pcbi.1006090 (PMC5933800; doi:10.1371/journal.pcbi.1006090)

A549 (4243 sequences): Best Model with 11 components

InMoDe	DIVERSITY

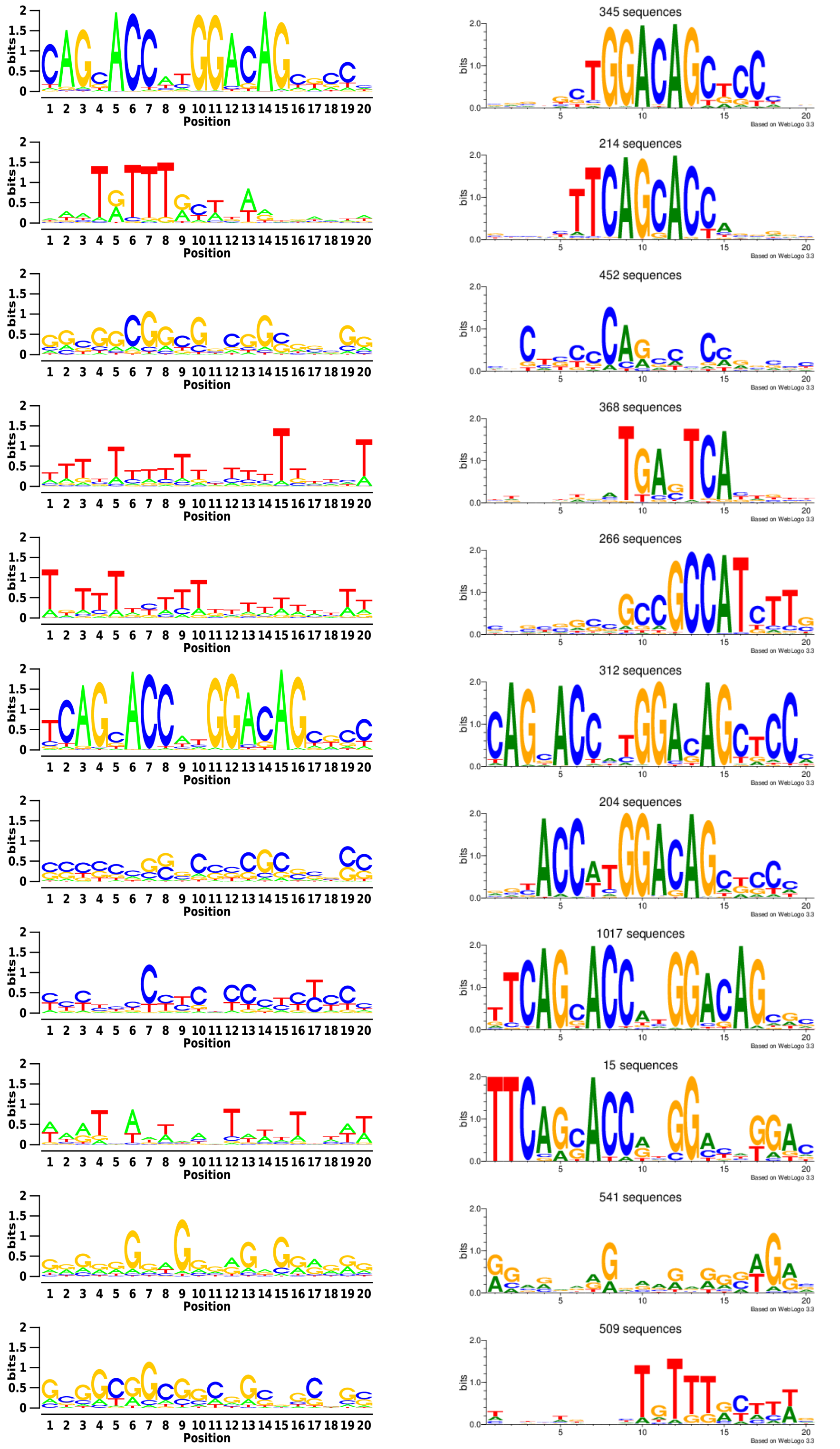

ECC-1 (4410 sequences): Best Model with 7 components

InMoDe	DIVERSITY

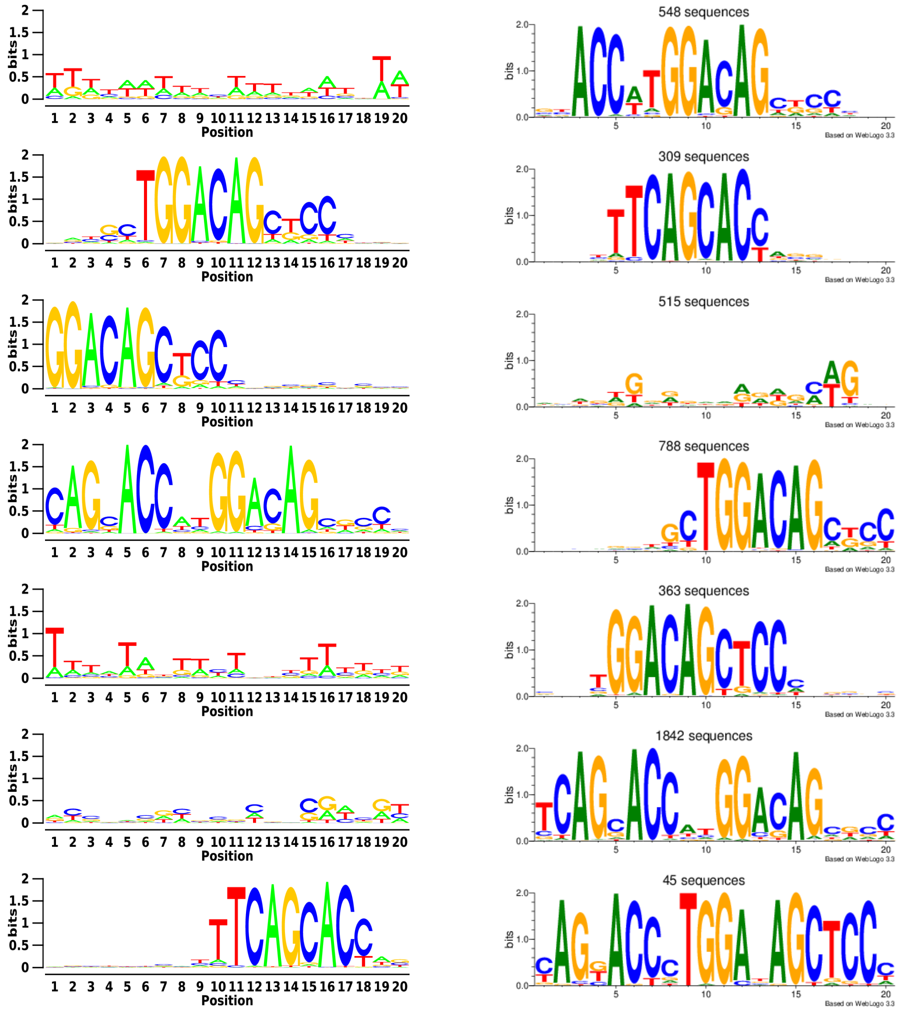

GM12878 (2292 sequences): Best Model with 6 components

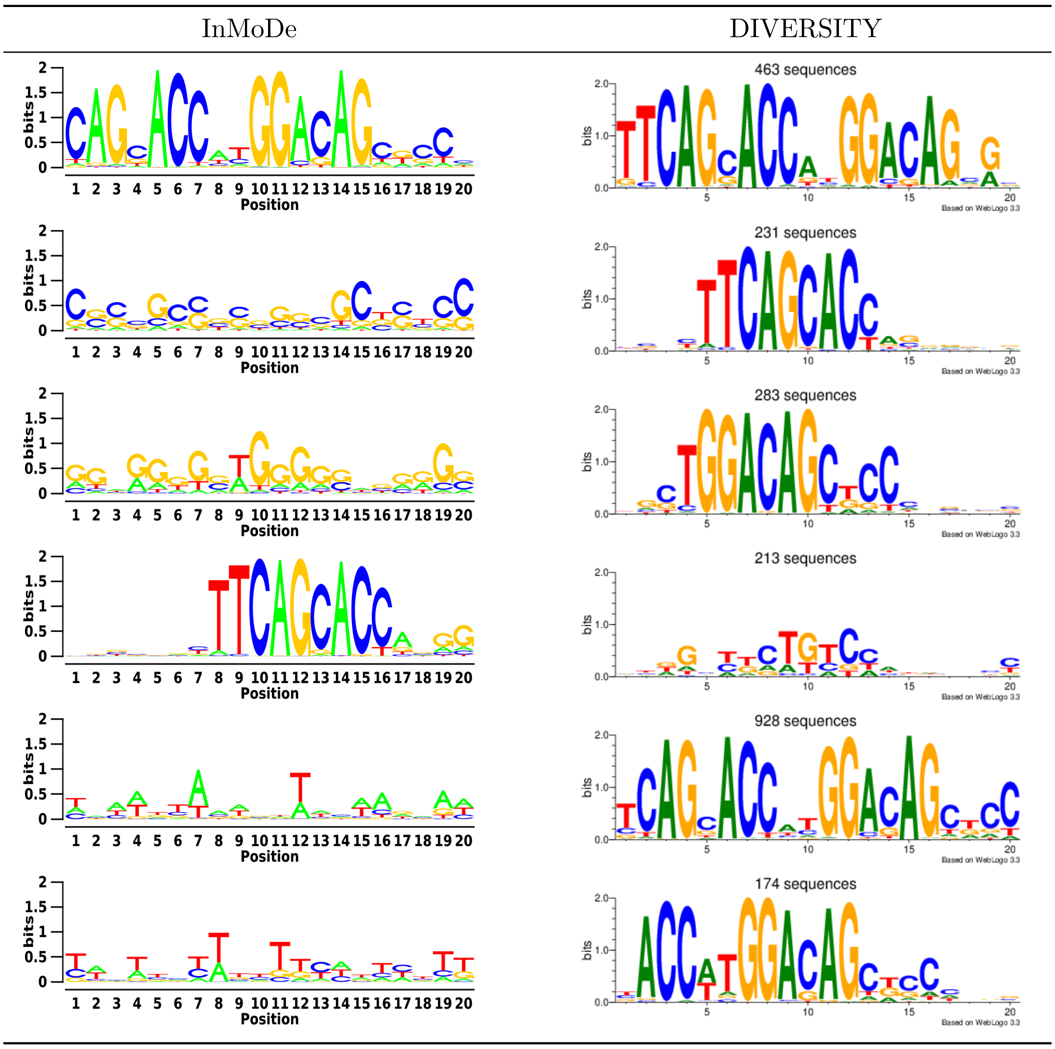

H1ESC (6882 sequences): Best Model with 11 components

InMoDe	DIVERSITY

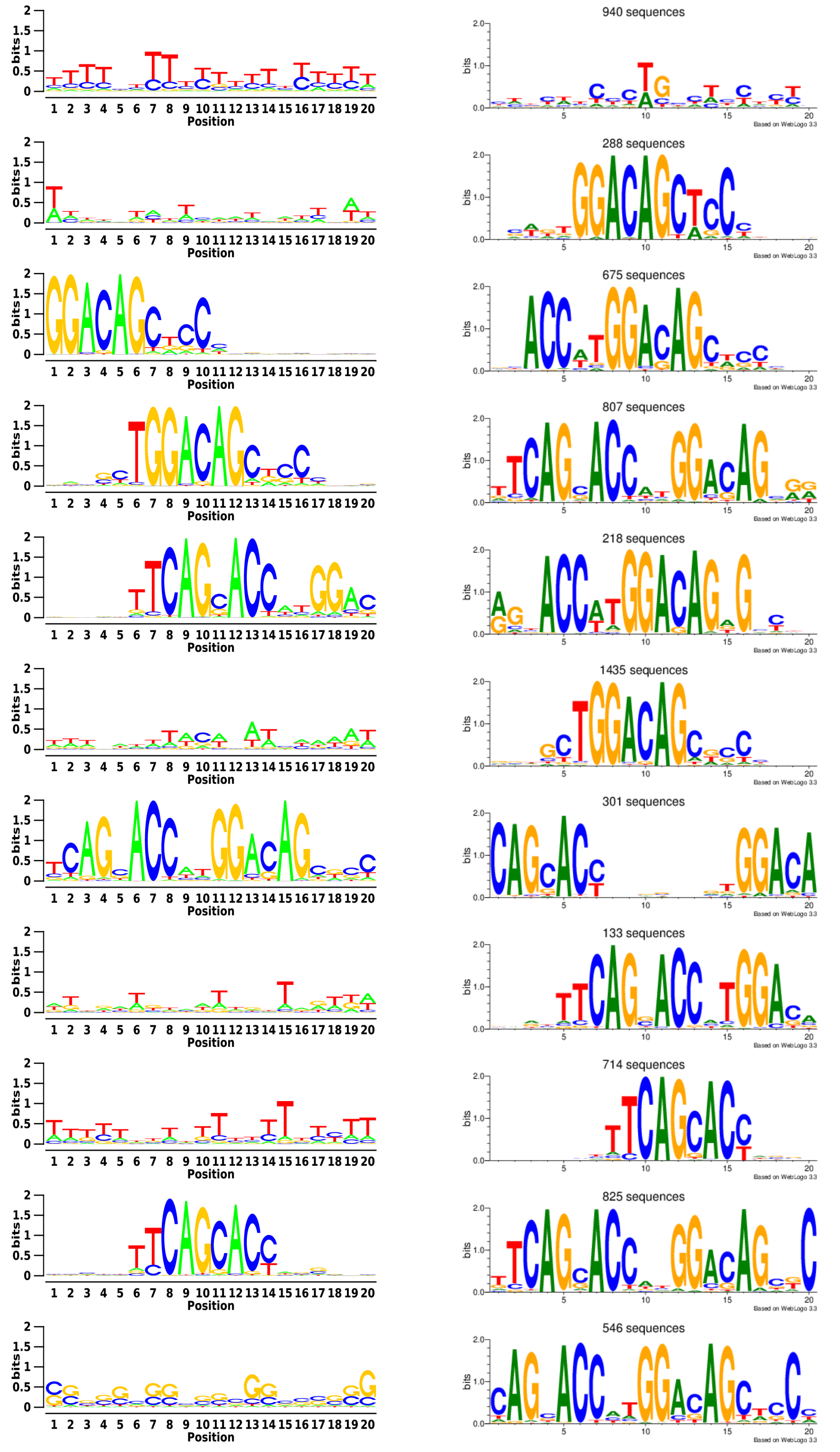

HCT-116 (2436 sequences): Best Model with 6 components

InMoDe	DIVERSITY
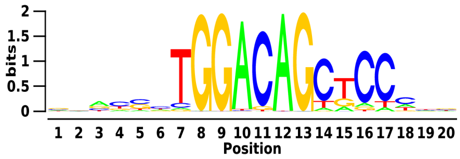	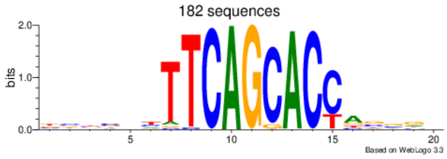
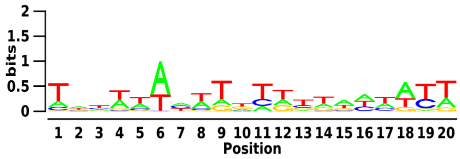	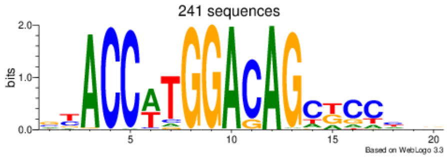
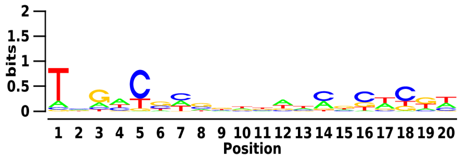	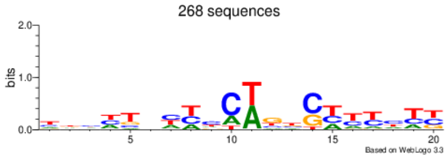
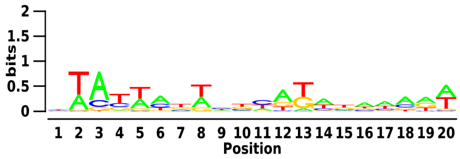	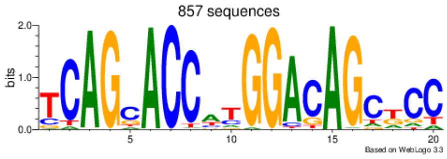
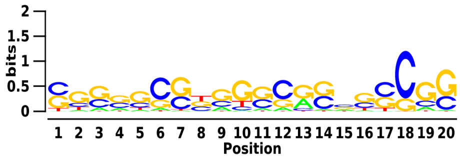	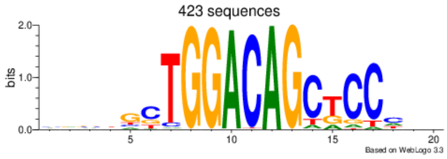
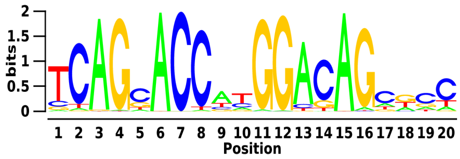	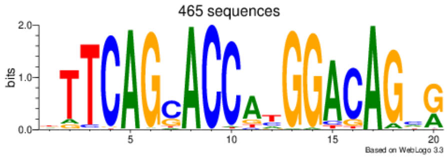

HL-60 (3683 sequences): Best Model with 8 components

InMoDe	DIVERSITY

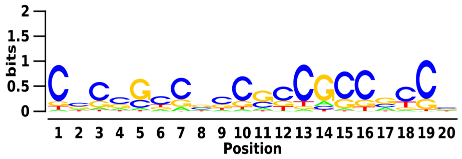

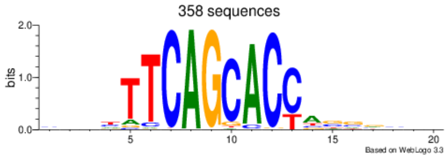

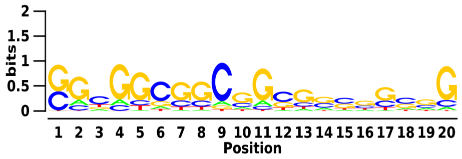

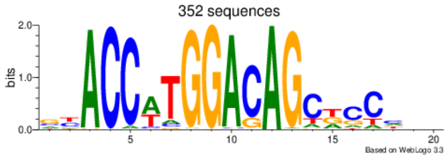

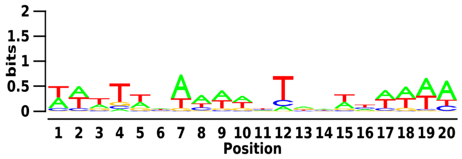

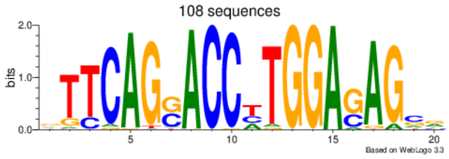

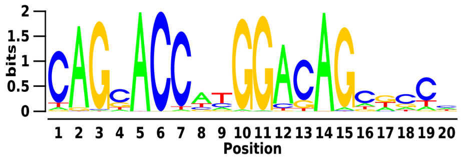

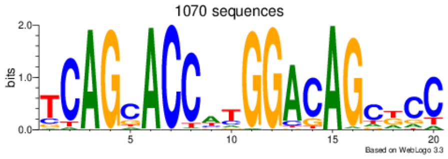

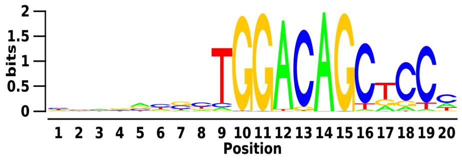

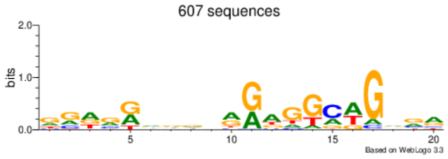

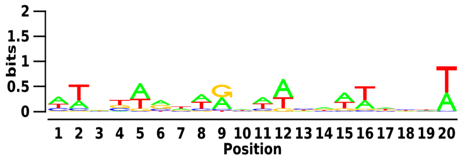

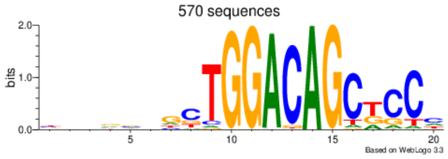

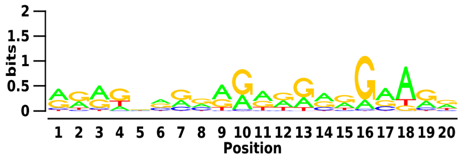

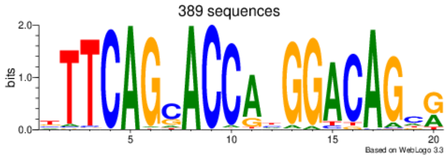

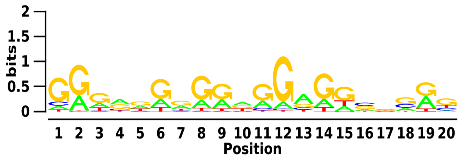

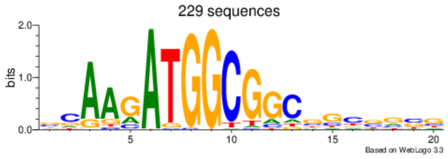

HeLaS3 (3526 sequences): Best Model with 7 components

InMoDe	DIVERSITY

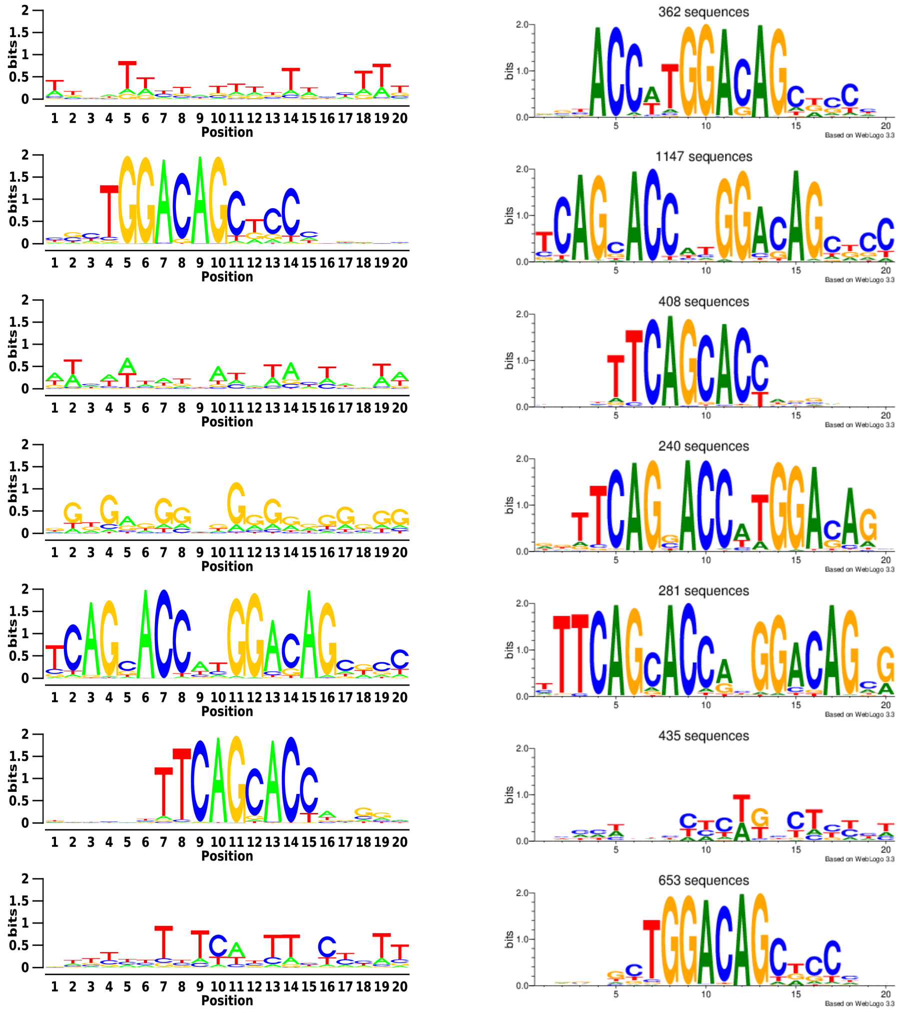

HepG2 (2706 sequences): Best Model with 7 components

InMoDe	DIVERSITY
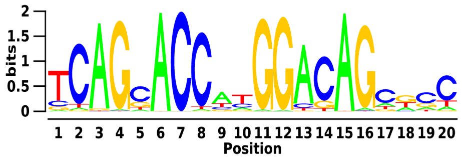	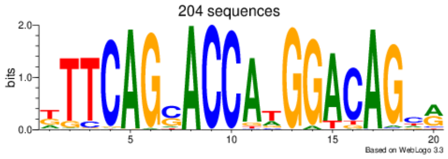
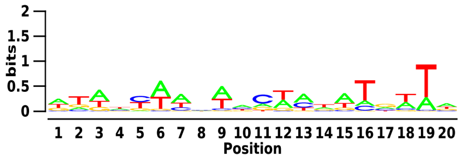	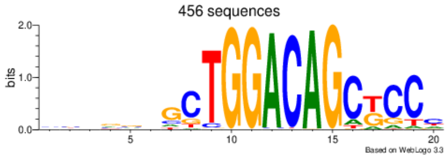
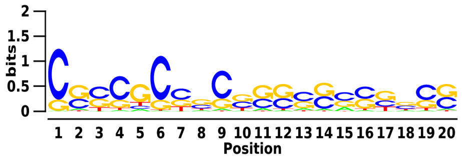	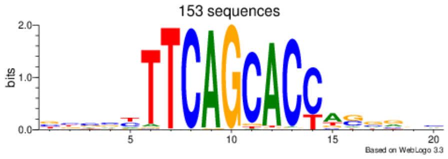
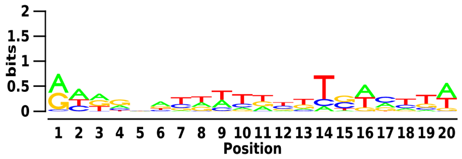	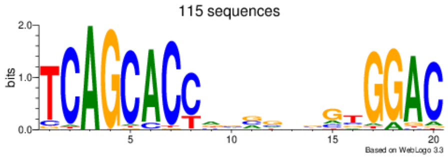
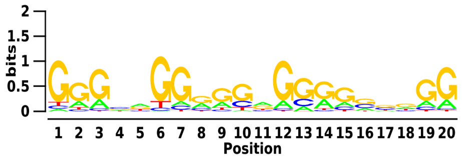	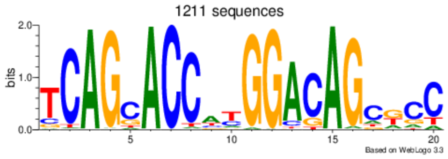
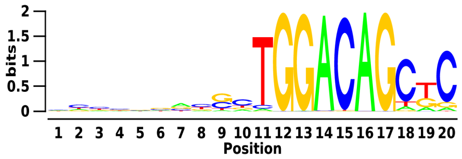	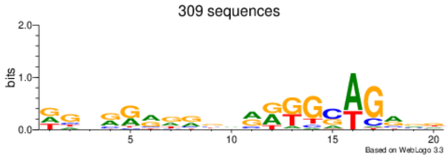
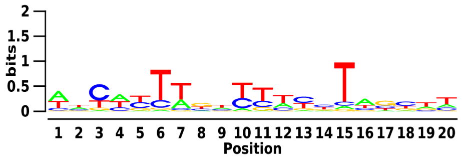	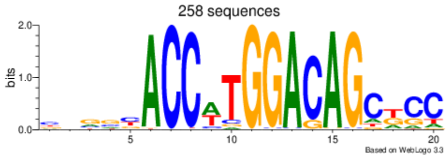

K562 (4810 sequences): Best Model with 9 components

InMoDe	DIVERSITY

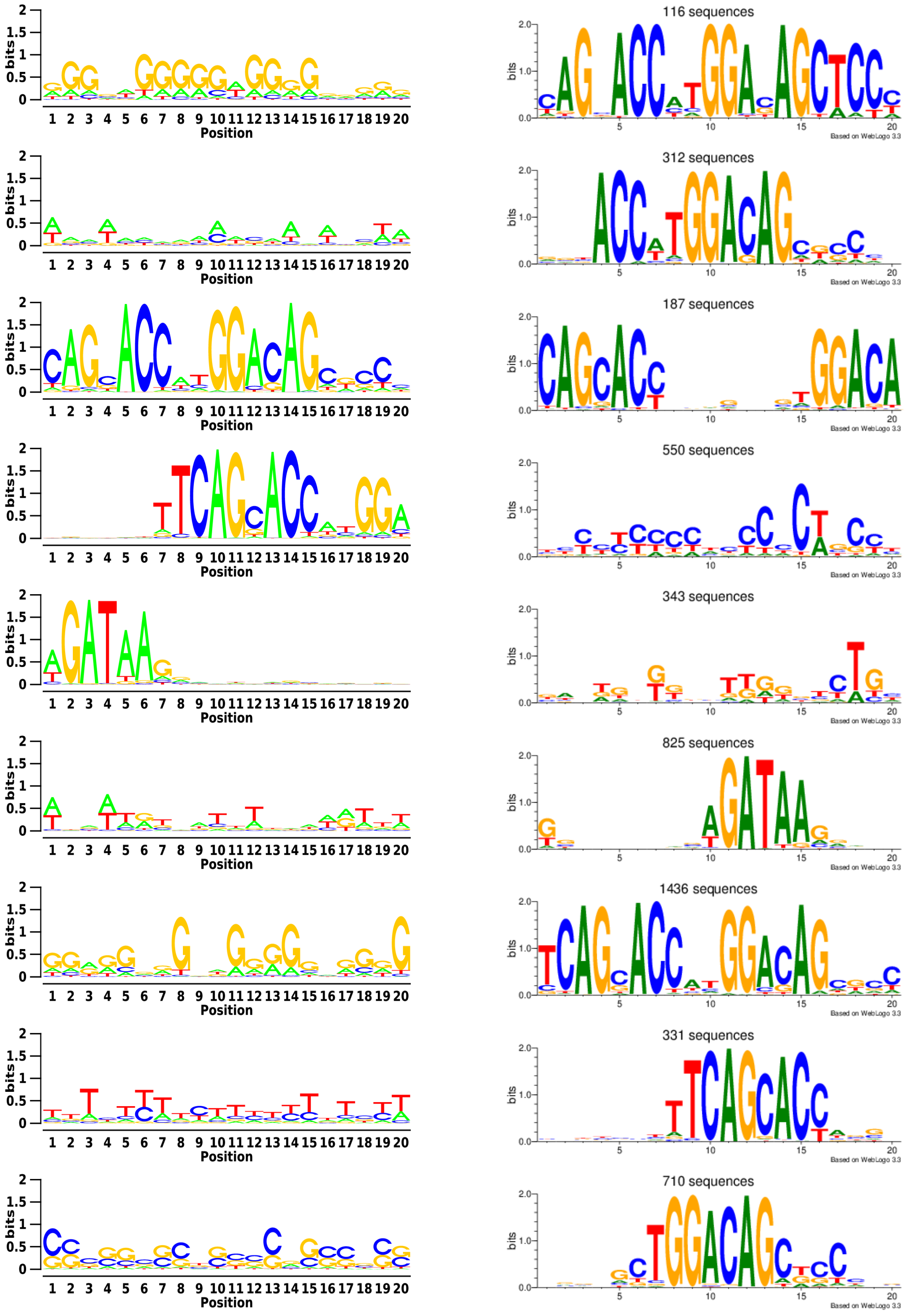

MCF-7 (3431 sequences): Best Model with 8 components

InMoDe	DIVERSITY

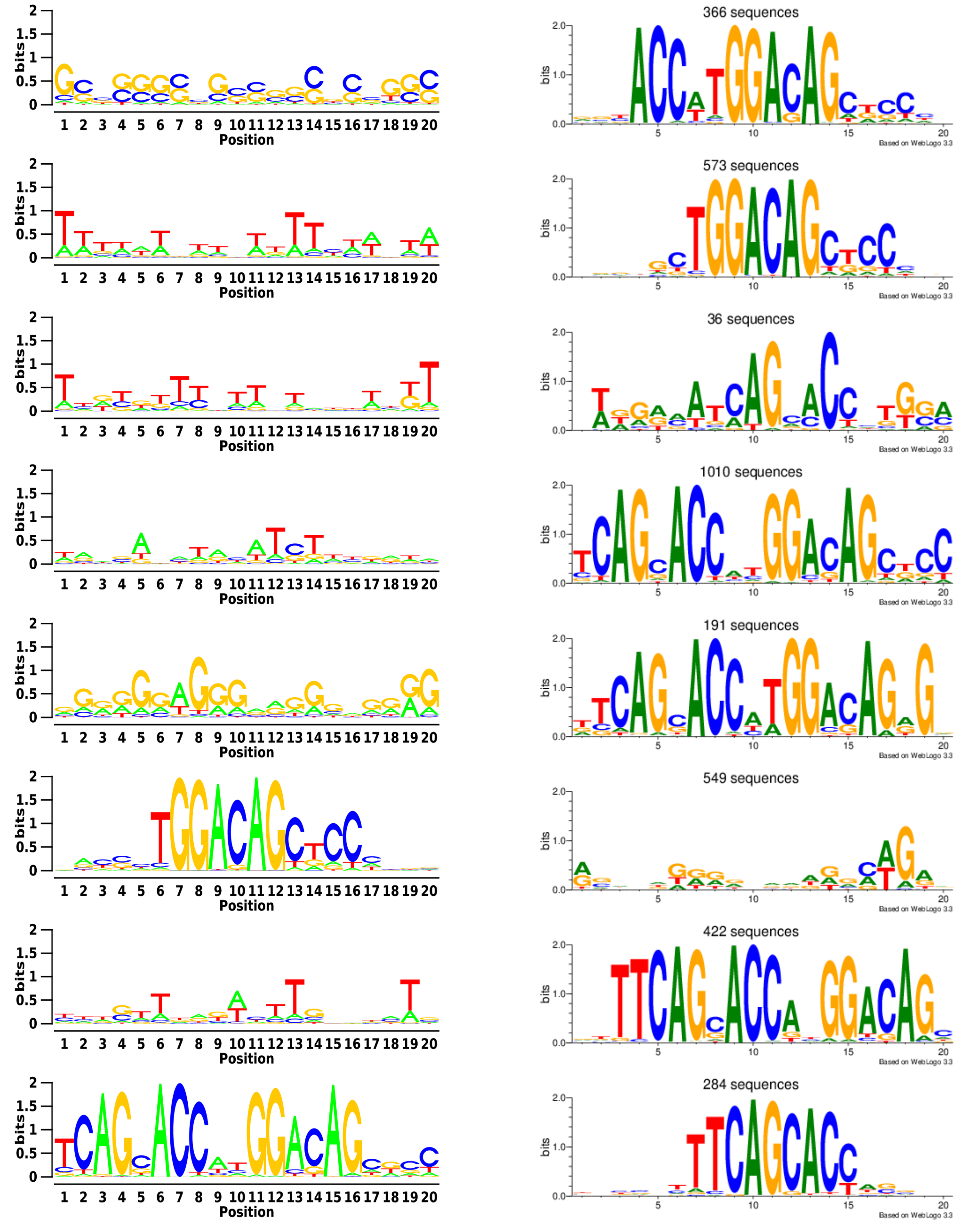

Neuron (5123 sequences): Best Model with 9 components

InMoDe	DIVERSITY

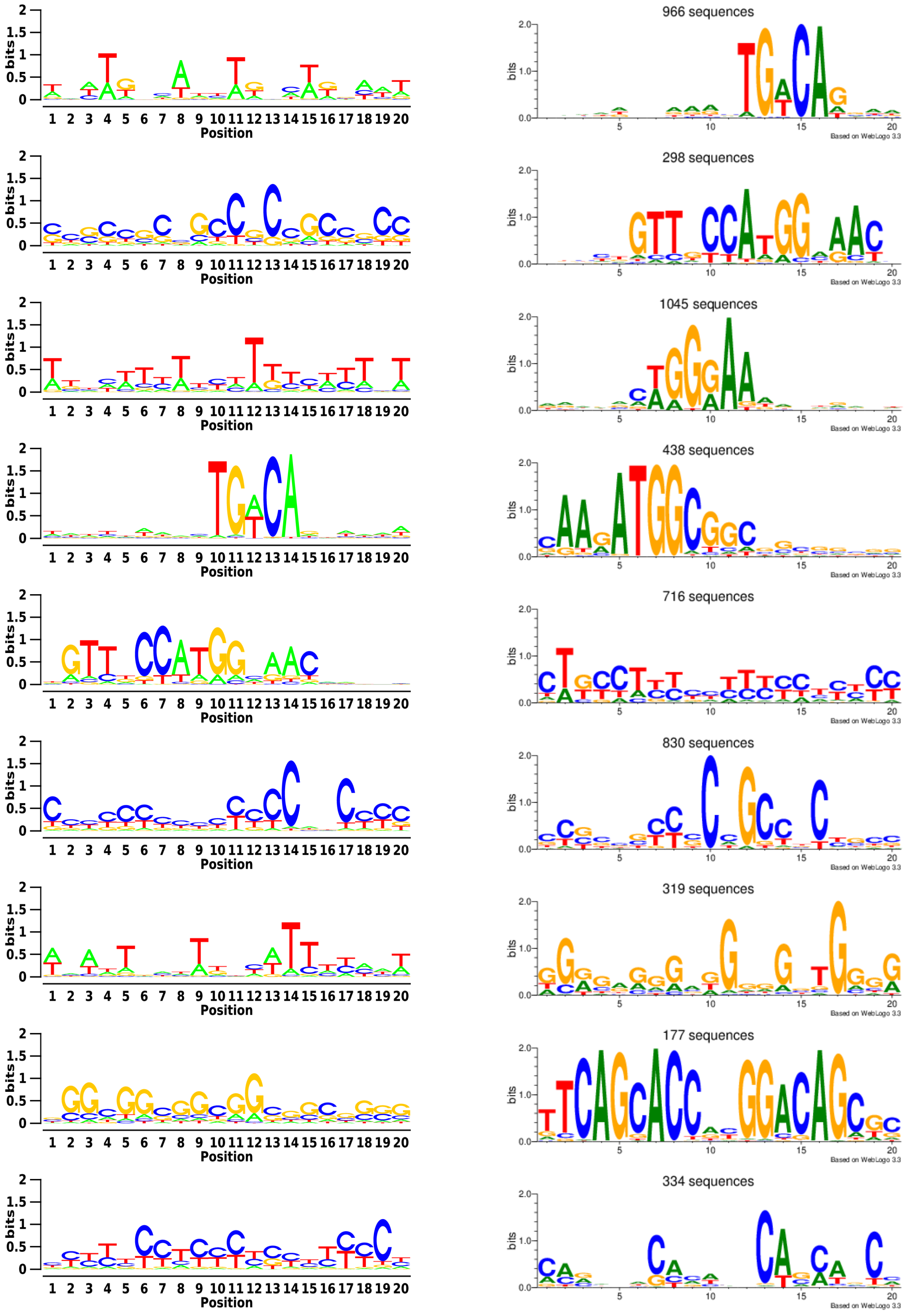

PANC-1 (2885 sequences): Best Model with 6 components

InMoDe	DIVERSITY
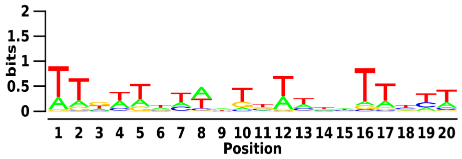	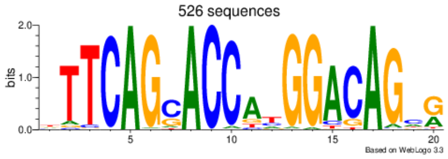
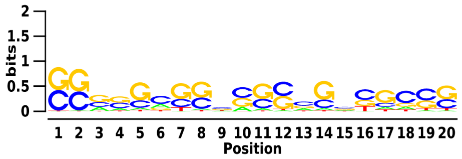	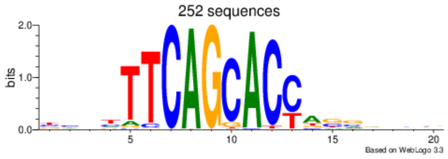
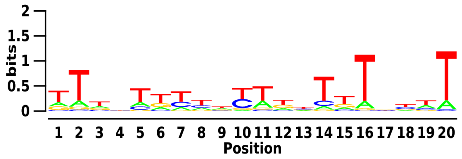	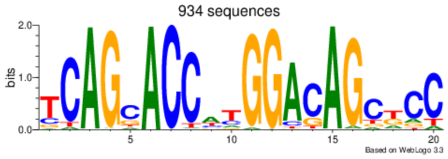
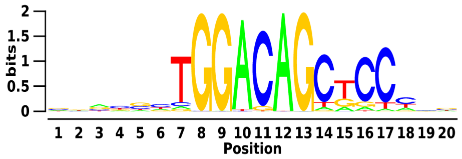	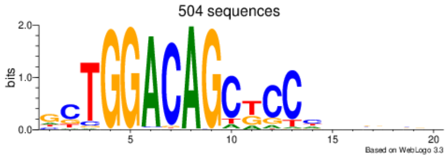
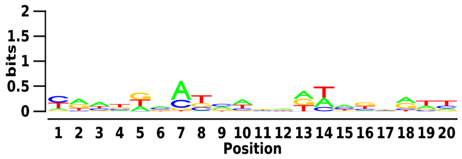	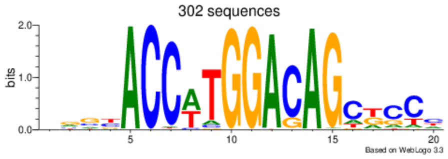
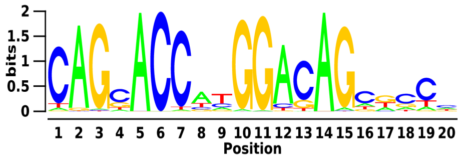	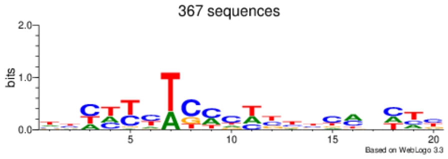

PFSK-1 (3167 sequences): Best Model with 7 components

InMoDe	DIVERSITY

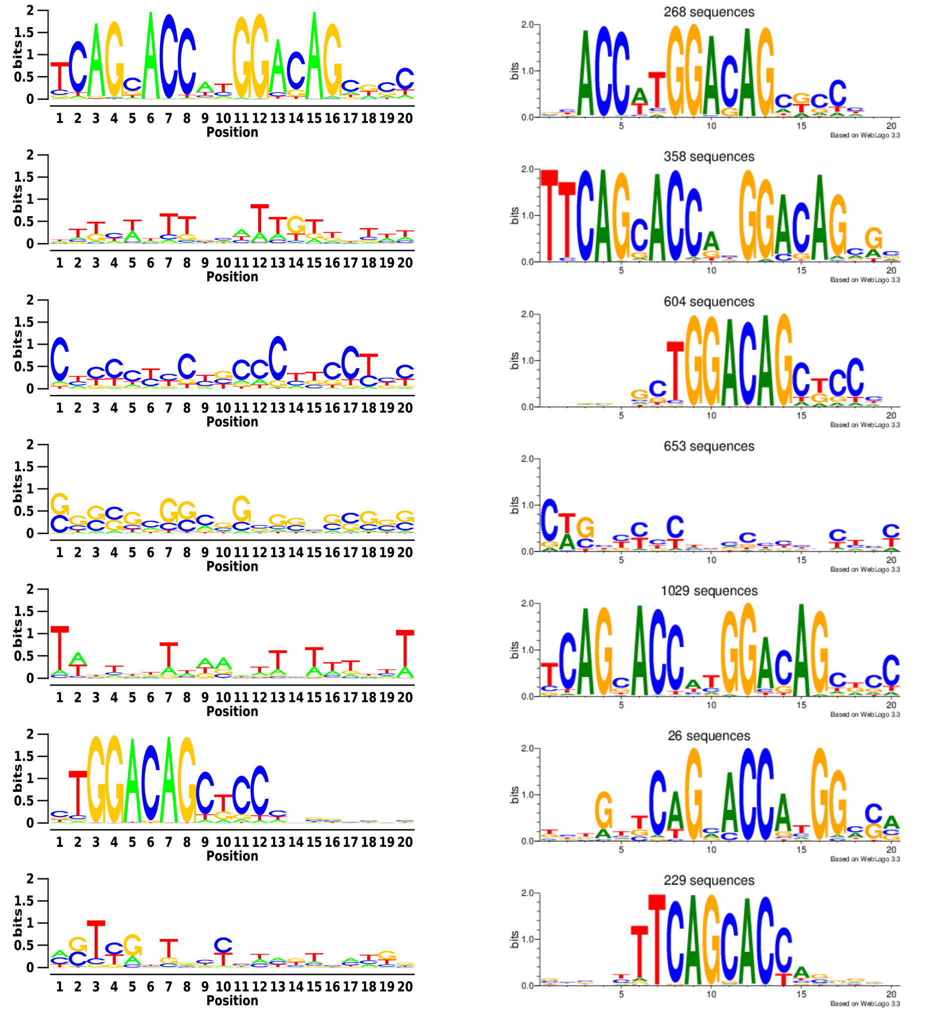

SK-N-SH (3610 sequences): Best Model with 6 components

InMoDe	DIVERSITY

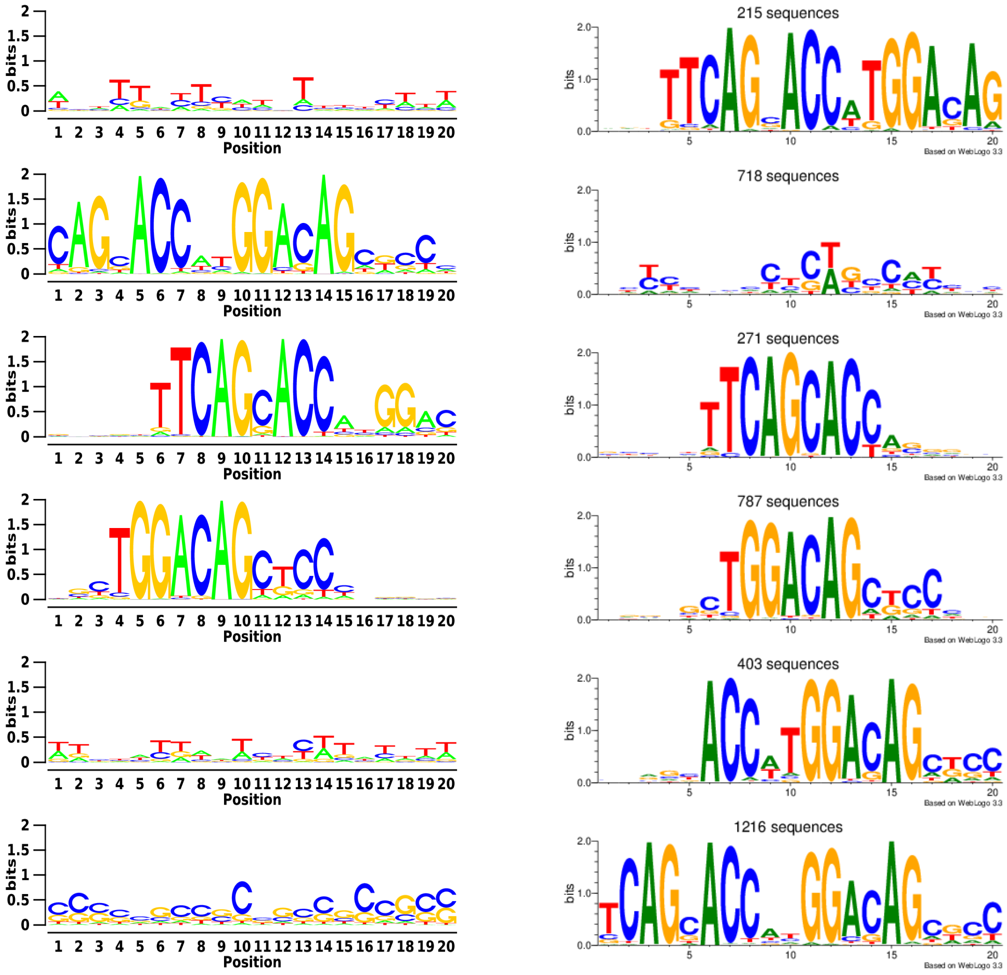

Tcell (4177 sequences): Best Model with 6 components

InMoDe	DIVERSITY
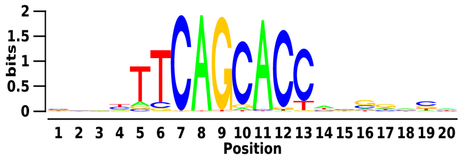	1282 sequences 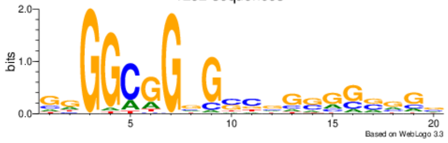
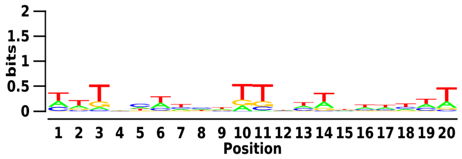	774 sequences 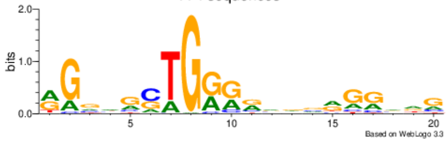
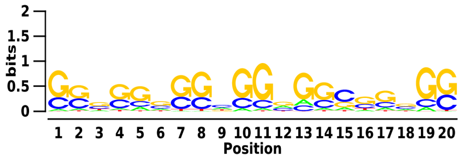	449 sequences 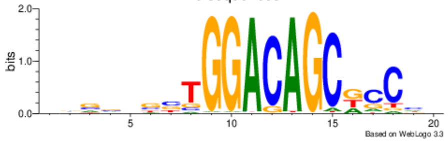
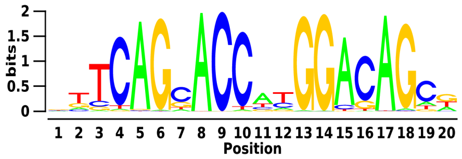	115 sequences 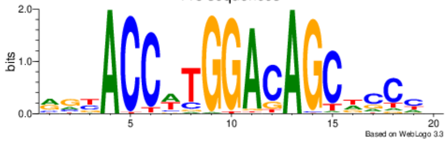
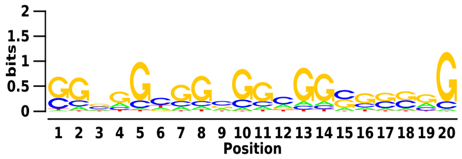	1058 sequences 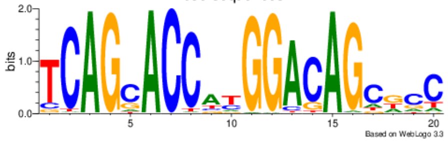
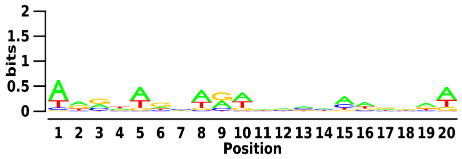	499 sequences 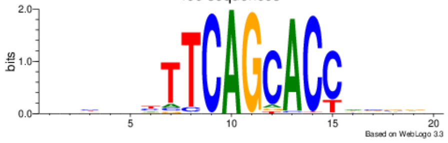

U87 (2238 sequences): Best Model with 7 components

InMoDe	DIVERSITY

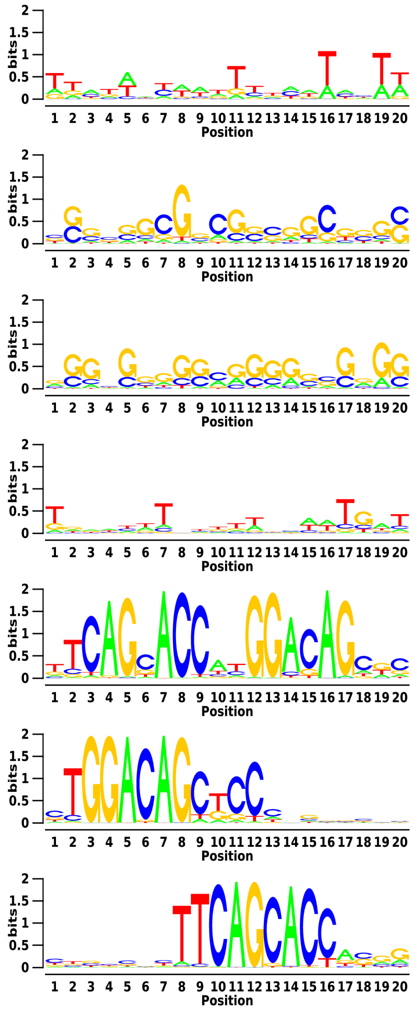

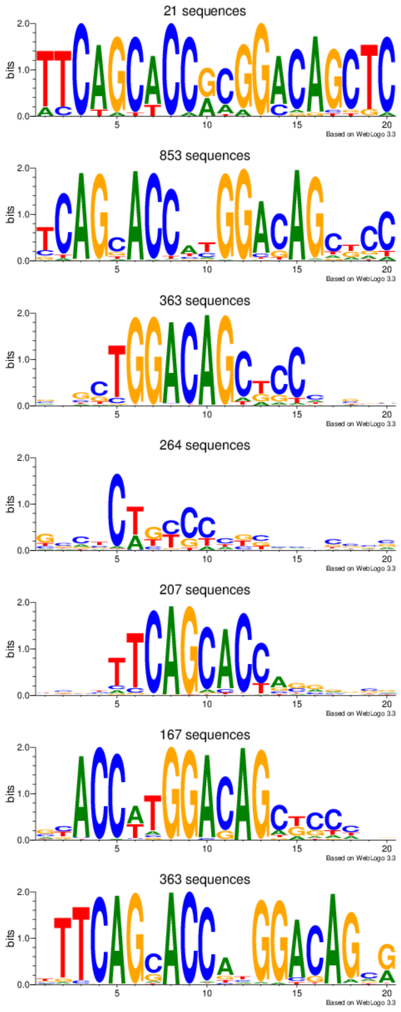

Supplement: S8 Fig — (PDF) [file pcbi.1006090.s008.pdf]
