# Supplementary material for: DIVERSITY in binding, regulation, and evolution revealed from high-throughput ChIP
Source: PLoS Comput Biol. 2018 Apr 23;14(4):e1006090. doi: 10.1371/journal.pcbi.1006090 (PMC5933800; doi:10.1371/journal.pcbi.1006090)
Supplement: S1 File — (GZ) [file pcbi.1006090.s009.tar.gz › DIVERSITY-master/weblogoMod/weblogolib/htdocs/create_html_template.html]

WebLogo 3 - Create 


|  |  |
| --- | --- |
| WebLogo 3 : Create | · about · create · examples · manual ·   $version |
| Sequence data |  |
|  | (or paste sequence data below) |
| ${sequences} | |
|  | Download |
| ${error\_message} | |
| Output format | PNG (low res.) PNG (high res.) JPEG (low res.) EPS (vector) PDF (vector) SVG (vector) Data (plain text) |
| Logo size | small medium large |
| Stacks per line |  |
| Sequence type | auto protein dna rna |
| Ignore lower case |  |
| Units | probability bits nats kT kJ/mol kcal/mol |
| First position number |  |
| Logo range | - |
| Composition | No adjustment for composition auto equiprobable Percentage CG ⇒ C. elegans (36%)  D. melanogaster (43%) E. coli (50.5%)  H. sapiens (40%)  M. musculus (42%) S. cerevisiae (38%)  or  % CG |
| Scale stack widths |  |
| Error bars |  |
| Title |  |
| Figure label |  |
| X-axis | Label: |
| Y-axis | Label: |
| Y-axis scale: |  |
| Y-axis tic spacing: |  |
| Sequence end labels |  |
| Version fineprint |  |
| Color scheme | Auto Monochrome Base pairing (NA default) Classic (NA) Hydrophobicity (AA default) Chemistry (AA) Charge (AA) Custom (Specify below) |
|  | |  |  | | --- | --- | | Symbols | Color | |  |  | |  |  | |  |  | |  |  | |  |  | |
|  |  |
|  |  |
