# Supplementary material for: DIVERSITY in binding, regulation, and evolution revealed from high-throughput ChIP
Source: PLoS Comput Biol. 2018 Apr 23;14(4):e1006090. doi: 10.1371/journal.pcbi.1006090 (PMC5933800; doi:10.1371/journal.pcbi.1006090)
Supplement: S1 File — (GZ) [file pcbi.1006090.s009.tar.gz › DIVERSITY-master/weblogoMod/weblogolib/htdocs/examples.html]

WebLogo 3 - Examples


|  |  |
| --- | --- |
| WebLogo 3: Examples | · about · create · examples · manual · |
| - CAP HTH motif - Transcription Factor Binding Sites - *E. coli* Promoters - Globins - HTH motif - Human Splice Sites   The **Edit Logo** buttons will transfer the relevant sequence data to the Logo creation form. There you can examine the sequence data and recreate the logo for yourself. Additional examples can be found at the Sequence Logo Gallery.   ---   Catobolite Activator Protein (CAP) The helix-turn-helix motif from the CAP family of homodimeric DNA binding proteins. CAP (Catabolite Activator Protein, also known as CRP for cAMP Receptor Protein) is a transcription promoter that binds at more than 100 sites within the *E. coli* genome. Residues 1-7 form the first helix, 8-11 the turn and 12-20 the DNA recognition helix. The glycine at position 9 appears to be critical in forming the turn. Positions 4, 8, 10, 15 and 19 are partially or completely buried, and therefore tend to be populated by hydrophobic amino acids, which are colored black. Positions 11-14, 17 and 20 interact directly with bases in the major groove and are critical to the sequence specific binding of the protein. The data for this logo consists of 100 sequences from the full Pfam alignment of this family (Accession number PF00325). A few sequences with rare insertions were removed for convenience.                 The two DNA recognition helixes of the CAP homodimer insert themselves into consecutive turns of the major groove. Several consequences can be observed in this CAP binding site logo. The logo is approximately palindromic, which provides two very similar recognition sites, one for each subunit of the dimer. However, the binding site is not perfectly symmetric, possible due to the inherent asymmetry of the operon promoter region. The displacement of the two parts is 11 base pairs, or approximately one full turn of the DNA helix. Additional interactions between the protein and the first and last two bases occur within the DNA minor groove, where it is difficult for the protein to distinguish A from T, or G from C. The data for this logo consists of 59 binding sites determined by DNA footprinting: Robison, K., McGuire, A. M., Church, G. M. A comprehensive library of DNA-binding site matrices for 55 proteins applied to the complete *Escherichia coli* K12 genome. Journal of Molecular Biology (1998) 284, 241-254.               ---  *E. coli* Transcription Factor Binding Sites The following logos (along with the CAP logo above) display a selection of *E. coli* transcription factor binding sites determined by DNA footprinting. This data has been collated in the DPInteract database and has been used to search for additional binding sites within the *E. coli* genome.  Robison, K., McGuire, A. M., Church, G. M. A comprehensive library of DNA-binding site matrices for 55 proteins applied to the complete *Escherichia coli* K12 genome. Journal of Molecular Biology (1998) 284, 241-254.      LexA repressor is closely related to CAP, and has similar DNA protein interactions.      H-NS: Histone like, nucleoid-associated DNA-binding protein.      DNA biosynthesis initiation binding protein.      Arginine Repressor.    ---  *E. coli* Promoters (Transcription Start Signals) In prokaryotes the DNA sequence just upstream of the transcription start point contains two important conserved regions. The first such region is centered at around 35bp upstream and is involved in the initial recognition of the gene by RNA polymerase. The second region, sometimes referred to as the Pribnow box, is centered at about 10bp upstream. The typical separation between the -35 and -10 sites is 15-18 bp. See baseflip: Strong Minor Groove Base Conservation in Sequence Logos implies DNA Distortion or Base Flipping during Replication and Transcription Initiation for more information. This sequence data was kindly provided by Prof. Julia Brettschneider <juliab@stat.berkeley.edu>   The -10 region of 350 E. coli promoters    ---  Globins  The end of the B helix through the beginning of the D helix of 34 globins. This sequence data was taken from Sequence Logos: A New Way to Display Consensus Sequences.                    ---  HTH Proteins   Helix-Turn-Helix DNA binding motifs found by the Gibbs sampling system. Compared to the CAP HTH logo there is much less sequence conservation within the DNA binding helix (11-17), as might be expected for a diverse sample of proteins.        ---  Human Splice Sites     These logos show a small sample of Human intron-exon splice boundaries. Sequences of experimentally confirmed genes were extracted from EID: the Exon-Intron database. Additional discussion of the features in this logo can be found in the paper Features of spliceosome evolution...   Exon-Intron (Donor) Sites    Intron-Exon (Acceptor) Sites | |
