# Supplementary material for: DIVERSITY in binding, regulation, and evolution revealed from high-throughput ChIP
Source: PLoS Comput Biol. 2018 Apr 23;14(4):e1006090. doi: 10.1371/journal.pcbi.1006090 (PMC5933800; doi:10.1371/journal.pcbi.1006090)
Supplement: S1 File — (GZ) [file pcbi.1006090.s009.tar.gz › DIVERSITY-master/weblogoMod/weblogolib/htdocs/index.html]

WebLogo 3 - About


|  |  |
| --- | --- |
| WebLogo 3 | · about · create · examples · manual · |
| Introduction **WebLogo** is a web based application designed to make the generation of sequence logos easy and painless. WebLogo has featured in over 2000 scientific publications.  A sequence logo is a graphical representation of an amino acid or nucleic acid multiple sequence alignment developed by Tom Schneider and Mike Stephens. Each logo consists of stacks of symbols, one stack for each position in the sequence. The overall height of the stack indicates the sequence conservation at that position, while the height of symbols within the stack indicates the relative frequency of each amino or nucleic acid at that position. In general, a sequence logo provides a richer and more precise description of, for example, a binding site, than would a consensus sequence.   - Create your own logos - View example sequence logos and input data. - Read the release notes for latest changes and updates. - Read the User's Manual - Download WebLogo source code - WebLogo discussion group  References Crooks GE, Hon G, Chandonia JM, Brenner SE WebLogo: A sequence logo generator,   *Genome Research*, 14:1188-1190, (2004) [Full Text ]  Schneider TD, Stephens RM. 1990. Sequence Logos: A New Way to Display Consensus Sequences. *Nucleic Acids Res.* *18*:6097-6100 Warning Please do not abuse our server. If you need to create large numbers of logos then you are welcome to download the code and run WebLogo on your own machine. If a request flood compromises the WebLogo server then your IP address will be unceremoniously blocked. Disclaimer While no permanent records are kept of submitted sequences, we cannot undertake to guarantee that data sent to WebLogo remains secure. Moreover, no guarantees whatsoever are provided about data generated by WebLogo. Feedback Suggestions on how to improve WebLogo are heartily welcomed! Please direct questions to the WebLogo discussion group. | |
