# Supplementary material for: DIVERSITY in binding, regulation, and evolution revealed from high-throughput ChIP
Source: PLoS Comput Biol. 2018 Apr 23;14(4):e1006090. doi: 10.1371/journal.pcbi.1006090 (PMC5933800; doi:10.1371/journal.pcbi.1006090)
Supplement: S1 File — (GZ) [file pcbi.1006090.s009.tar.gz › DIVERSITY-master/weblogoMod/weblogolib/htdocs/manual.html]

WebLogo 3 - User's Manual


|  |  |
| --- | --- |
| WebLogo 3 : User's Manual | · about · create · examples · manual · |
| Contents   - Introduction - Creating Sequences Logos using the Web interface - Downloading and Installing WebLogo - Command Line Interface (CLI) - Application Programmer Interface (API) - Development and Future Features - Miscellanea  Release Notes and Known BugsThe WebLogo release notes detail changes to WebLogo and known issues with particular versions. Introduction **WebLogo** is a web based application designed to make the generation of sequence logos as easy and painless as possible.  A sequence logo is a graphical representation of an amino acid or nucleic acid multiple sequence alignment. Each logo consists of stacks of symbols, one stack for each position in the sequence. The overall height of the stack indicates the sequence conservation at that position, while the height of symbols within the stack indicates the relative frequency of each amino or nucleic acid at that position. The width of the stack is proportional to the fraction of valid symbols in that position. (Positions with many gaps have thin stacks.) In general, a sequence logo provides a richer and more precise description of, for example,a binding site, than would a consensus sequence. References Crooks GE, Hon G, Chandonia JM, Brenner SE WebLogo: A sequence logo generator, *Genome Research*, 14:1188-1190, (2004) [Full Text ]  Schneider TD, Stephens RM. 1990. Sequence Logos: A New Way to Display Consensus Sequences. *Nucleic Acids Res.* *18*:6097-6100 Creating Sequences Logos using the Web interfaceSequence DataEnter your multiple sequence alignment or position weight matrix file, or select a file to upload. Supported file formats include CLUSTALW, FASTA, plain flatfile, MSF, NBRF, PIR, NEXUS and PHYLIP for multiple sequence alignments, and transfac for position weight matrices. All sequences must be the same length, else WebLogo will return an error and report the first sequence that differed in length from previous sequences.Output format  - PNG : (600 DPI) Print resolution bitmap - PNG : (low res, 96 DPI) Screen resolution bitmap - JPEG :Screen resolution bitmap - EPS : Encapsulated postscript - PDF : Portable Document Format - SVG : Scalable Vector Graphics  Generally speaking, vector formats (EPS and PDF) are better for printing, while bitmaps (JPEG and PNG) are more suitable for displaying on the screen or embedding into a web page.Logo sizeThe physical dimensions of the generated logo. Specifically, controls the size of individual symbols stacks.  - small : 5.4 points wide (same as 9pt Courier), aspect ratio 5:1 - medium : Double the width and height of small. - large : Triple the width and height of small.  The choices have been limited to promote inter-logo consistency. Small logos can fit 80 stacks across a printed page, or 40 across a half page column. The command line interface provides greater control, if so desired.Stacks per lineIf the length of the sequences is greater than this maximum number of stacks per line, then the logo will be split across multiple lines.Sequence typeThe type of biological molecule.  - auto: Automatically guess sequence type from the data - protein - dna - rna  Ignore lower caseDisregard lower case letters and only count upper case letters in sequences?UnitsThe units used for the y-axis.  - probability: Show residue probabilities, rather than information content. If compositional adjustment is disabled, then these are the raw residue frequencies. - bits: Information content in bits - nats: Natural units, 1 bit = ln 2 (0.69) nats - kT : Thermal energy units in natural units (Numerically the same as nats) - kJ/mol : Thermal energy (assuming T = 300 K) - kcal/mol : Thermal energy (assuming T = 300 K)  First position number The numerical label of the first position in the sequence data in the input file. The label must be an integer. Residue labels for the logo will be relative to this number. (See also: Logo range.)Logo range By default, all sequence data from the input file is displayed in the Sequence Logo. With this option, you can instead show a subrange of the sequence data. The numbering of Start and End Positions is relative to the First Position Number. Thus, if the First Position Number is "2", Start is "5" and End is "10", then the 4th through 9th (inclusive) sequence positions of the input file will be displayed, and they will be numbered "5", "6", "7", "8", "9" and "10". Composition The background composition of the genome or proteome from which the sequences have been drawn. The default, automatic option is to use equiprobable background for nucleic acids and a typical amino acid usage pattern for proteins. However, you may also explicitly set the expected CG content for nucleic acid sequences, insist on equiprobable background distributions, or turn off composition adjustment altogether.  Compositional adjustment has two effects. First, the information content of a site is defined as the relative entropy of the monomers at that site to the background distribution. Consequentially, rare monomers have higher information content (when they occur) than relatively common monomers.  Secondly, the background composition is used in the small sample correction of information content. Briefly, if only a few sequences are available in the multiple sequence alignment, then sites typically appear more conserved than they really are. Small samples bias the relative entropy upwards. To compensate, we add pseudocounts to the actual counts, proportional to the expected background composition. These pseudocounts smooth the data for small samples, but become irrelevant for large samples. The proportionality constant is set to 4 for nucleic acid sequences, and 20 for proteins (these numbers have been found to give reasonable results in practice).  Behind the scenes, things are more complex. We do a full Bayesian calculation, starting with explicit Dirichlet priors based on the background composition, to which we add the data and then calculate both the posterior mean relative entropy (the stack height) and Bayesian 95% confidence intervals for error bars. These interesting details will be explained elsewhere. Scale stack widthScale the visible stack width by the fraction of symbols in the column? (I.e. columns with many gaps or unknown residues are narrow.)Error barsDisplay error bars. These indicate an approximate, Bayesian 95% confidence interval.TitleGive your logo a title.Figure labelAn optional figure label, added to the top left (e.g. '(a)').X-axisAdd a label to the x-axis, or hide axis altogether.Y-axisThe vertical axis indicates the information content of a sequence position. Use this option to toggle the y-axis and override the default axis label.Y-axis scaleThe height of the y-axis in designated units. The automatic option will pick reasonable defaults based on the sequence type and axis unit.Y-axis tic spacingThe distance between major tic marks on the y-axis.Sequence end labelsChoose this option to label the 5' & 3' ends of nucleic acid or the N & C termini of amino acid sequences.Version fineprintToggle display of the WebLogo version information in the lower right corner. Optional, but we appreciate the acknowledgment.Color Scheme  - auto : use Base Pairing for nucleic acids (NA), Hydrophobicity for amino acids (AA). - monochrome : All symbols black - Base Pairing (NA default) :    |  |  |  |   | --- | --- | --- |   | 2 Watson-Crick hydrogen bonds | TAU | dark orange |   | 3 Watson-Crick hydrogen bonds | GC | blue | - Classic (NA) : WebLogo (version 1 and 2) and makelogo default color scheme for nucleic acids: G, orange; T & U, red; C, blue; and A, green.    |  |  |   | --- | --- |   | G | orange |   | TU | red |   | C | blue |   | A | green | - Hydrophobicity (AA default) :    |  |  |  |   | --- | --- | --- |   | Hydrophilic | RKDENQ | blue |   | Neutral | SGHTAP | green |   | Hydrophobic | YVMCLFIW | black | - Chemistry (AA) : Color amino acids according to chemical properties. WebLogo (version 1 and 2) and makelogo default color. (Note that the WebLogo 2 documentation erroneously lists Q and N under green.)    |  |  |  |   | --- | --- | --- |   | Polar | G,S,T,Y,C | green |   | Neutral | Q,N | purple |   | Basic | K,R,H | blue |   | Acidic | D,E | red |   | Hydrophobic | A,V,L,I,P,W,F,M | black | - Charge (AA) :    |  |  |  |   | --- | --- | --- |   | Positive | KRH | blue |   | Negative | DE | red | - Custom : A custom color scheme can be specified in the input field below. Specify colors on the left and associated symbols on the right. Colors are entered using CSS2 (Cascading Style Sheet) syntax. (E.g. 'red', '#F00', '#FF0000', 'rgb(255, 0, 0)', 'rgb(100%, 0%, 0%)' or 'hsl(0, 100%, 50%)' for the color red.)  More OptionsThe weblogo command line client, `weblogo`, provides many more options and greater control over the final logo appearance. Installing WebLogoDependencies WebLogo version 3 is written in python. It is necessary to have python 2.5, 2.6 or 2.7 and the extension package numpy installed before WebLogo will run. WebLogo also requires a recent version of ghostscript to create PNG and PDF output, and pdf2svg to generate SVG output. Download and Installation If the python package `setuptools` has been installed, then WebLogo and its dependencies can be downloaded and installed with a single command:   ``` sudo easy_install weblogo ```   Alternatively, weblogo and its dependencies can be installed manually. The WebLogo source code can be downloaded from http://code.google.com/p/weblogo/. This code is distributed under various open source licenses. Please consult the `LICENSE.txt` file in the source distribution for details.  After unpacking the WebLogo tarfile, it should be possible to immediately create logos using the command line client (provided that python, numpy and ghostscript have already been installed).   ``` ./weblogo --format PNG < cap.fa > cap.png ```   Please consult the file `build_examples.sh` for more examples.  To run WebLogo as a stand alone web service, run the logo server command :   ``` ./weblogo --serve ```   It should now be possible to access WebLogo at http://localhost:8080/.  The command line client and WebLogo libraries can be permanently installed using the supplied `setup.py` script.   ``` sudo python setup.py install ```   Run `python setup.py help` for more installation options. For example, to specifically install the weblogo script to /usr/local/bin   ``` sudo python setup.py install_scripts --install-dir /usr/local/bin ```  Web App To use WebLogo as a web application, first install the weblogo dependencies and libraries as above, then place (or link) the `weblogolib/htdocs` directory somewhere within the document root of your webserver. The webserver must be able to execute the CGI script `create.cgi`. For Apache, you may have to add an `ExecCGI` option and add a cgi handler in the `httpd.conf` configuration file. Something like this:   ``` <Directory "/home/httpd/htdocs/weblogo/">     Options FollowSymLinks MultiViews ExecCGI     AllowOverride None     Order allow,deny     Allow from all </Directory> ... # To use CGI scripts outside of ScriptAliased directories: # (You will also need to add "ExecCGI" to the "Options" directive.) # AddHandler cgi-script .cgi ```  It may also be necessary to set the `PATH` and `PYTHONPATH` environment variables.  ``` SetEnv PYTHONPATH /path/to/weblogo/libraries ```  The cgi script also has to be able to find the '`gs`' ghostscript executable. If ghostscript is installed in a non-standard location add the following environment variable.  ``` SetEnv COREBIOPATH /path/to/gs ```  The maximum bytes of uploaded sequence data can be controlled with the `WEBLOGO_MAX_FILE_SIZE` environment variable.  ``` SetEnv WEBLOGO_MAX_FILE_SIZE 1000000 ```  `weblogo`, The WebLogo Command Line Interface (CLI)The command line client has many options not available through the web interface. Please consult the bundled `build_examples.sh` script for inspiration.  ``` Usage: weblogo [options]  < sequence_data.fa > sequence_logo.eps  Create sequence logos from biological sequence alignments.  Options:      --version                  show program's version number and exit   -h --help                     show this help message and exit    Input/Output Options:     -f --fin FILENAME           Sequence input file (default: stdin)     -D --datatype FORMAT        Type of multiple sequence alignment or                                 position weight matrix file: (clustal, fasta,                                 plain, msf, genbank, nbrf, nexus, phylip,                                 stockholm, intelligenetics, table, array,                                 transfac)     -o --fout FILENAME          Output file (default: stdout)     -F --format FORMAT          Format of output: eps (default), png,                                 png_print, pdf, jpeg, svg, logodata    Logo Data Options:     -A --sequence-type TYPE     The type of sequence data: 'protein', 'rna' or                                 'dna'.     -a --alphabet ALPHABET      The set of symbols to count, e.g. 'AGTC'. All                                 characters not in the alphabet are ignored. If                                 neither the alphabet nor sequence-type are                                 specified then weblogo will examine the input                                 data and make an educated guess. See also                                 --sequence-type, --ignore-lower-case     -U --units NUMBER           A unit of entropy ('bits' (default), 'nats',                                 'digits'), or a unit of free energy ('kT',                                 'kJ/mol', 'kcal/mol'), or 'probability' for                                 probabilities        --composition COMP.      The expected composition of the sequences:                                 'auto' (default), 'equiprobable', 'none' (do                                 not perform any compositional adjustment), a                                 CG percentage, a species name (e.g. 'E. coli',                                 'H. sapiens'), or an explicit distribution                                 (e.g. "{'A':10, 'C':40, 'G':40, 'T':10}"). The                                 automatic option uses a typical distribution                                 for proteins and equiprobable distribution for                                 everything else.        --weight NUMBER          The weight of prior data.  Default depends on                                 alphabet length     -i --first-index INDEX      Index of first position in sequence data                                 (default: 1)     -l --lower INDEX            Lower bound of sequence to display     -u --upper INDEX            Upper bound of sequence to display    Transformations:     Optional transformations of the sequence data.         --ignore-lower-case      Disregard lower case letters and only count                                 upper case letters in sequences.        --reverse                reverse sequences        --complement             complement DNA sequences    Logo Format Options:     These options control the format and display of the logo.      -s --size LOGOSIZE          Specify a standard logo size (small, medium                                 (default), large)     -n --stacks-per-line COUNT  Maximum number of logo stacks per logo line.                                 (default: 40)     -t --title TEXT             Logo title text.        --label TEXT             A figure label, e.g. '2a'     -X --show-xaxis YES/NO      Display sequence numbers along x-axis?                                 (default: True)     -x --xlabel TEXT            X-axis label        --annotate TEXT          A comma separated list of custom stack                                 annotations, e.g. '1,3,4,5,6,7'.  Annotation                                 list must be same length as sequences.     -S --yaxis UNIT             Height of yaxis in units. (Default: Maximum                                 value with uninformative prior.)     -Y --show-yaxis YES/NO      Display entropy scale along y-axis? (default:                                 True)     -y --ylabel TEXT            Y-axis label (default depends on plot type and                                 units)     -E --show-ends YES/NO       Label the ends of the sequence? (default:                                 False)     -P --fineprint TEXT         The fine print (default: weblogo version)        --ticmarks NUMBER        Distance between ticmarks (default: 1.0)        --errorbars YES/NO       Display error bars? (default: True)        --reverse-stacks YES/NO  Draw stacks with largest letters on top?                                 (default: True)    Color Options:     Colors can be specified using CSS2 syntax. e.g. 'red', '#FF0000', etc.      -c --color-scheme SCHEME    Specify a standard color scheme (auto, base                                 pairing, charge, chemistry, classic,                                 hydrophobicity, monochrome)     -C --color COLOR SYMBOLS DESCRIPTION                                  Specify symbol colors, e.g. --color black AG                                 'Purine' --color red TC 'Pyrimidine'        --default-color COLOR    Symbol color if not otherwise specified.    Advanced Format Options:     These options provide fine control over the display of the logo.      -W --stack-width POINTS     Width of a logo stack (default: 10.8)        --aspect-ratio POINTS    Ratio of stack height to width (default: 5)        --box YES/NO             Draw boxes around symbols? (default: no)        --resolution DPI         Bitmap resolution in dots per inch (DPI).                                 (Default: 96 DPI, except png_print, 600 DPI)                                 Low resolution bitmaps (DPI<300) are                                 antialiased.        --scale-width YES/NO     Scale the visible stack width by the fraction                                 of symbols in the column?  (I.e. columns with                                 many gaps of unknowns are narrow.)  (Default:                                 yes)        --debug YES/NO           Output additional diagnostic information.                                 (Default: False)    WebLogo Server:     Run a standalone webserver on a local port.         --serve                  Start a standalone WebLogo server for creating                                 sequence logos.        --port PORT              Listen to this local port. (Default: 8080) ```  WebLogo Application Programmer Interface (API)The WebLogo python libraries provide even greater flexibility than the command line client. The code is split between two principle packages, `weblogo` itself, which contains specialized sequence logo generation code, and `corebio`, a package that contains code of more general utility. Please consult the WebLogo and CoreBio API documentation. WebLogo Development and Future Features The development project is hosted at http://code.google.com/p/weblogo. If you wish to extend WebLogo or to contribute code, then you should download the full source code development package directly from the subversion repository.   ``` > svn checkout http://weblogo.googlecode.com/svn/trunk/ weblogo >  cd weblogo > ./weblogo < cap.fa > cap.eps ```   Please consult the developer notes, `DEVELOPERS.txt` and software license `LICENSE.txt`  Outstanding bugs and feature requests are listed on the WebLogo issue tracker. MiscellaneaWebLogo 2The legacy WebLogo 2 sever can be found here.Acknowledgments WebLogo was created by Gavin E. Crooks, Liana Lareau, Gary Hon, John-Marc Chandonia and Steven E. Brenner. Many others have provided suggestions, bug fixes and moral support.  WebLogo was originally based upon the programs alpro and makelogo, both of which are part of Tom Schneider's delila package. Many thanks are due to him for making this software freely available and for encouraging its use. Disclaimer While no permanent records are kept of submitted sequences, we cannot undertake to guarantee that data sent to WebLogo remains secure. Moreover, no guarantees whatsoever are provided about data generated by WebLogo. Feedback Suggestions on how to improve WebLogo are heartily welcomed! Please direct questions to the WebLogo discussion group. | |
