# Supplementary material for: DIVERSITY in binding, regulation, and evolution revealed from high-throughput ChIP
Source: PLoS Comput Biol. 2018 Apr 23;14(4):e1006090. doi: 10.1371/journal.pcbi.1006090 (PMC5933800; doi:10.1371/journal.pcbi.1006090)
Supplement: S1 File — (GZ) [file pcbi.1006090.s009.tar.gz › DIVERSITY-master/weblogoMod/weblogolib/htdocs/test.html]

WebLogo 3 - Tests 


|  |  |
| --- | --- |
| WebLogo 3: Tests | · about · create · examples · manual · |
| Various tests of the WebLogo webapp.     ---   Relative Entropy Test  The entropy should be 2 bits, 1 bit, 0 bits (The small sample correction should be turned off.)  Title And Labels Test  Replace and display x-label, y-label and title.   Same, but do not show axes  Format Test  Ensure that this logo can be created in each of the available formats  Format:  PNG PNG (low res.) JPEG (low res.) EPS (vector) PDF (vector) data  Test Alphabetic Order  Each character in each stack has the same entropy. The letters should be alphabetized, top down.  (NA)         (AA)  One Single Column  Should not die just because there is only 1 stack.  Small Sample Correction Test  The samples per column decrease from 32 (left) to 1 (right). Before the small sample correction the relative entropy of each stack is 2 bits.  No adjustment for composition auto  Test Graceful Failure  Each of these tests should result in a polite and informative error message.  Logo Sizes  Same sequences, three different sizes.  Size: small medium large  Transfac test | |
