# Supplementary material for: DIVERSITY in binding, regulation, and evolution revealed from high-throughput ChIP
Source: PLoS Comput Biol. 2018 Apr 23;14(4):e1006090. doi: 10.1371/journal.pcbi.1006090 (PMC5933800; doi:10.1371/journal.pcbi.1006090)
Supplement: S1 File — (GZ) [file pcbi.1006090.s009.tar.gz › DIVERSITY-master/weblogoMod/apidocs/class-tree.html]

xml version="1.0" encoding="ascii"?


Class Hierarchy


| Home | Trees | Indices | Help | WebLogo |
| --- | --- | --- | --- | --- |

 **[ Module Hierarchy
| Class Hierarchy ]**   

# Class Hierarchy

- **weblogolib.color.Color**:
  *Color specifications using CSS2 (Cascading Style Sheet) syntax.*
- **object**:
  *The most base type*
  - **weblogolib.colorscheme.ColorGroup**:
    *Associate a group of symbols with a color*
  - **weblogolib.colorscheme.ColorScheme**:
    *A coloring of an alphabet.*
  - **weblogolib.logomath.Dirichlet**:
    *The Dirichlet probability distribution.*
  - **weblogolib.\_cgi.Field**:
    *A representation of an HTML form field.*
  - **weblogolib.logomath.Gamma**:
    *The gamma probability distribution.*
  - **weblogolib.GhostscriptAPI**:
    *Interface to the command line program Ghostscript ('gs')*
  - **weblogolib.LogoData**:
    *The data needed to generate a sequence logo.*
  - **weblogolib.LogoOptions**:
    *A container for all logo formatting options.*

| Home | Trees | Indices | Help | WebLogo |
| --- | --- | --- | --- | --- |

|  |  |
| --- | --- |
| Generated by Epydoc 3.0.1 on Mon Jan 30 17:27:18 2012 | http://epydoc.sourceforge.net |
