# Supplementary material for: DIVERSITY in binding, regulation, and evolution revealed from high-throughput ChIP
Source: PLoS Comput Biol. 2018 Apr 23;14(4):e1006090. doi: 10.1371/journal.pcbi.1006090 (PMC5933800; doi:10.1371/journal.pcbi.1006090)
Supplement: S1 File — (GZ) [file pcbi.1006090.s009.tar.gz › DIVERSITY-master/weblogoMod/apidocs/identifier-index.html]

xml version="1.0" encoding="ascii"?


Identifier Index


| Home | Trees | Indices | Help | WebLogo |
| --- | --- | --- | --- | --- |

|  |  |
| --- | --- |
| Identifier Index | [ A B C D E F G H I J K L M N O P Q R S T U V W X Y Z \_ ] |

|  |  |
| --- | --- |
| A | aa\_composition (in weblogolib) \| alphabets (in weblogolib.\_cgi) \| |
| B | base\_distribution() (in weblogolib) \| base\_pairing (in weblogolib.colorscheme) \| by\_name() (in Color) |
| C | cdf() (in Gamma) \| Color (in weblogolib.color) \| ColorScheme (in weblogolib.colorscheme); charge (in weblogolib.colorscheme) \| color() (in ColorScheme) \| composition (in weblogolib.\_cgi); chemistry (in weblogolib.colorscheme) \| color\_schemes (in weblogolib.\_cgi) \| convert() (in GhostscriptAPI); classic (in weblogolib) \| ColorGroup (in weblogolib.colorscheme) \| covariance() (in Dirichlet); color (in weblogolib) \| colorscheme (in weblogolib) \| |
| D | default\_color\_schemes (in weblogolib) \| description (in weblogolib) \| Dirichlet (in weblogolib.logomath) |
| E | eps\_formatter() (in weblogolib) \| equiprobable\_distribution() (in weblogolib) \| extension (in weblogolib.\_cgi) |
| F | Field (in weblogolib.\_cgi) \| formatters (in weblogolib) \| from\_rgb() (in Color); find\_root() (in weblogolib.logomath) \| from\_counts() (in LogoData) \| from\_seqs() (in LogoData); float\_or\_none() (in weblogolib.\_cgi) \| from\_hsl() (in Color) \| from\_shape\_scale() (in Gamma); formats (in GhostscriptAPI) \| from\_mean\_variance() (in Gamma) \| from\_string() (in Color) |
| G | Gamma (in weblogolib.logomath) \| get\_value() (in Field) \| GhostscriptAPI (in weblogolib) |
| H | httpd\_serve\_forever() (in weblogolib.\_cli) \| hydrophobicity (in weblogolib.colorscheme) \| |
| I | int\_or\_none() (in weblogolib.\_cgi) \| interval\_relative\_entropy() (in Dirichlet) \| inverse\_cdf() (in Gamma) |
| J | jpeg\_formatter() (in weblogolib) \| \| |
| L | LogoData (in weblogolib) \| logomath (in weblogolib) \|; LogoFormat (in weblogolib) \| LogoOptions (in weblogolib) \| |
| M | main() (in weblogolib.\_cgi) \| mean() (in Gamma) \| mean\_x() (in Dirichlet); main() (in weblogolib.\_cli) \| mean\_entropy() (in Dirichlet) \| mime\_type (in weblogolib.\_cgi); mean() (in Dirichlet) \| mean\_relative\_entropy() (in Dirichlet) \| monochrome (in weblogolib.colorscheme) |
| N | names() (in Color) \| nucleotide (in weblogolib.colorscheme) \| |
| P | pdf() (in Gamma) \| png\_formatter() (in weblogolib) \|; pdf\_formatter() (in weblogolib) \| png\_print\_formatter() (in weblogolib) \| |
| R | read\_seq\_data() (in weblogolib) \| release\_date (in weblogolib) \| resource\_string() (in weblogolib.\_cgi); release\_build (in weblogolib) \| release\_description (in weblogolib) \| |
| S | sample() (in Dirichlet) \| std\_alphabets (in weblogolib) \| std\_sizes (in weblogolib); sample() (in Gamma) \| std\_color\_schemes (in weblogolib) \| std\_units (in weblogolib); send\_form() (in weblogolib.\_cgi) \| std\_percentCG (in weblogolib) \| string\_or\_none() (in weblogolib.\_cgi) |
| T | taylor (in weblogolib.colorscheme) \| truth() (in weblogolib.\_cgi) \| txt\_formatter() (in weblogolib) |
| V | variance() (in Gamma) \| variance\_relative\_entropy() (in Dirichlet) \| version() (in GhostscriptAPI); variance\_entropy() (in Dirichlet) \| variance\_x() (in Dirichlet) \| |
| W | weblogolib \| \| |
| \_ | \_\_eq\_\_() (in Color) \| \_\_init\_\_() (in Color) \| \_\_repr\_\_() (in Color); \_\_init\_\_() (in GhostscriptAPI) \| \_\_init\_\_() (in ColorGroup) \| \_\_str\_\_() (in LogoData); \_\_init\_\_() (in LogoData) \| \_\_init\_\_() (in ColorScheme) \| \_cgi (in weblogolib); \_\_init\_\_() (in LogoFormat) \| \_\_init\_\_() (in Dirichlet) \| \_cli (in weblogolib); \_\_init\_\_() (in LogoOptions) \| \_\_init\_\_() (in Gamma) \| \_std\_colors (in weblogolib.color); \_\_init\_\_() (in Field) \| \_\_repr\_\_() (in LogoOptions) \| |

  
  

| Home | Trees | Indices | Help | WebLogo |
| --- | --- | --- | --- | --- |

|  |  |
| --- | --- |
| Generated by Epydoc 3.0.1 on Mon Jan 30 17:27:18 2012 | http://epydoc.sourceforge.net |
