# Supplementary material for: DIVERSITY in binding, regulation, and evolution revealed from high-throughput ChIP
Source: PLoS Comput Biol. 2018 Apr 23;14(4):e1006090. doi: 10.1371/journal.pcbi.1006090 (PMC5933800; doi:10.1371/journal.pcbi.1006090)
Supplement: S1 File — (GZ) [file pcbi.1006090.s009.tar.gz › DIVERSITY-master/weblogoMod/apidocs/index.html]

xml version="1.0" encoding="ascii"?


weblogolib


| Home | Trees | Indices | Help | WebLogo |
| --- | --- | --- | --- | --- |

Package weblogolib

# Package weblogolib

source code

```
WebLogo (http://code.google.com/p/weblogo/) is a tool for creating sequence 
logos from biological sequence alignments.  It can be run on the command line,
as a standalone webserver, as a CGI webapp, or as a python library.

The main WebLogo webserver is located at http://weblogo.threeplusone.com

Please consult the manual for installation instructions and more information:
(Also located in the weblogolib/htdocs subdirectory.)

    http://weblogo.threeplusone.com/manual.html

For help on the command line interface run
    ./weblogo --help

To build a simple logo run
    ./weblogo  < cap.fa > logo0.eps
    
To run as a standalone webserver at localhost:8080 
    ./weblogo --serve

To create a logo in python code:
    >>> from weblogolib import *
    >>> fin = open('cap.fa')
    >>> seqs = read_seq_data(fin) 
    >>> data = LogoData.from_seqs(seqs)
    >>> options = LogoOptions()
    >>> options.title = "A Logo Title"
    >>> format = LogoFormat(data, options)
    >>> fout = open('cap.eps', 'w') 
    >>> eps_formatter( data, format, fout)


-- Distribution and Modification --
This package is distributed under the new BSD Open Source License. 
Please see the LICENSE.txt file for details on copyright and licensing.
The WebLogo source code can be downloaded from 
http://code.google.com/p/weblogo/

WebLogo requires Python 2.5, 2.6 or 2.7, and the python
array package 'numpy' (http://www.scipy.org/Download)

Generating logos in PDF or bitmap graphics formats require that the ghostscript
program 'gs' be installed. Scalable Vector Graphics (SVG) format also requires 
the program 'pdf2svg'.
```

---

**Version:**
3.2


Submodules

- **weblogolib.\_cgi**
- **weblogolib.\_cli**
- **weblogolib.color**: *Color specifications using CSS2 (Cascading Style Sheet) syntax.*
- **weblogolib.colorscheme**: *Popular color codings for nucleic and amino acids.*
- **weblogolib.logomath**

  


|  |  |
| --- | --- |
| Classes | |
|  | GhostscriptAPI  Interface to the command line program Ghostscript ('gs') |
|  | LogoOptions  A container for all logo formatting options. |
|  | LogoFormat  Specifies the format of the logo. |
|  | LogoData  The data needed to generate a sequence logo. |


Functions

|  |  |
| --- | --- |
| pdf\_formatter(data, format, fout)  Generate a logo in PDF format. | source code |

|  |  |
| --- | --- |
| jpeg\_formatter(data, format, fout)  Generate a logo in JPEG format. | source code |

|  |  |
| --- | --- |
| png\_formatter(data, format, fout)  Generate a logo in PNG format. | source code |

|  |  |
| --- | --- |
| png\_print\_formatter(data, format, fout)  Generate a logo in PNG format with print quality (600 DPI) resolution. | source code |

|  |  |
| --- | --- |
| txt\_formatter(logodata, format, fout)  Create a text representation of the logo data. | source code |

|  |  |
| --- | --- |
| eps\_formatter(logodata, format, fout)  Generate a logo in Encapsulated Postscript (EPS) | source code |

|  |  |
| --- | --- |
| default\_formatter(logodata, format, fout)  Generate a logo in Encapsulated Postscript (EPS) | source code |

|  |  |
| --- | --- |
| base\_distribution(percentCG) | source code |

|  |  |
| --- | --- |
| equiprobable\_distribution(length) | source code |

|  |  |
| --- | --- |
| read\_seq\_data(fin, input\_parser=seq\_io.read, alphabet=None, ignore\_lower\_case=False, max\_file\_size=0)  Read sequence data from the input stream and return a seqs object. | source code |


|  |  |
| --- | --- |
| Variables | |
|  | description = `"Create sequence logos from biological sequence ...` |
|  | classic = `ColorScheme([ColorGroup("G", "orange"), ColorGroup("...` |
|  | std\_color\_schemes = `{"auto": None, "monochrome": monochrome, "...` |
|  | default\_color\_schemes = `{unambiguous_protein_alphabet: hydroph...` |
|  | std\_units = `{"bits": 1./ log(2), "nats": 1., "digits": 1./ log...` |
|  | std\_sizes = `{"small": 5.4, "medium": 5.4* 2, "large": 5.4* 3}` |
|  | std\_alphabets = `{'protein': unambiguous_protein_alphabet, 'rna...` |
|  | std\_percentCG = `{'H. sapiens': 40., 'E. coli': 50.5, 'S. cerev...` |
|  | formatters = `{'eps': eps_formatter, 'pdf': pdf_formatter, 'png...` |


Function Details

|  |  |
| --- | --- |
| read\_seq\_data(fin, input\_parser=seq\_io.read, alphabet=None, ignore\_lower\_case=False, max\_file\_size=0) | source code |

```
Read sequence data from the input stream and return a seqs object. 

The environment variable WEBLOGO_MAX_FILE_SIZE overides the max_file_size argument.
Used to limit the load on the WebLogo webserver.
```

  


Variables Details

### description

Value:
:   ```
    "Create sequence logos from biological sequence alignments."
    ```

### classic

Value:
:   ```
    ColorScheme([ColorGroup("G", "orange"), ColorGroup("TU", "red"), Color
    Group("C", "blue"), ColorGroup("A", "green")])
    ```

### std\_color\_schemes

Value:
:   ```
    {"auto": None, "monochrome": monochrome, "base pairing": base_pairing,
     "classic": classic, "hydrophobicity": hydrophobicity, "chemistry": ch
    emistry, "charge": charge,}
    ```

### default\_color\_schemes

Value:
:   ```
    {unambiguous_protein_alphabet: hydrophobicity, unambiguous_rna_alphabe
    t: base_pairing, unambiguous_dna_alphabet: base_pairing}
    ```

### std\_units

Value:
:   ```
    {"bits": 1./ log(2), "nats": 1., "digits": 1./ log(10), "kT": 1., "kJ/
    mol": 8.314472* 298.15/ 1000., "kcal/mol": 1.987* 298.15/ 1000., "prob
    ability": None,}
    ```

### std\_alphabets

Value:
:   ```
    {'protein': unambiguous_protein_alphabet, 'rna': unambiguous_rna_alpha
    bet, 'dna': unambiguous_dna_alphabet}
    ```

### std\_percentCG

Value:
:   ```
    {'H. sapiens': 40., 'E. coli': 50.5, 'S. cerevisiae': 38., 'C. elegans
    ': 36., 'D. melanogaster': 43., 'M. musculus': 42., 'T. thermophilus':
     69.4,}
    ```

### formatters

Value:
:   ```
    {'eps': eps_formatter, 'pdf': pdf_formatter, 'png': png_formatter, 'pn
    g_print': png_print_formatter, 'jpeg': jpeg_formatter, 'svg': svg_form
    atter, 'logodata': txt_formatter,}
    ```

  


| Home | Trees | Indices | Help | WebLogo |
| --- | --- | --- | --- | --- |

|  |  |
| --- | --- |
| Generated by Epydoc 3.0.1 on Mon Jan 30 17:27:18 2012 | http://epydoc.sourceforge.net |
