# Supplementary material for: DIVERSITY in binding, regulation, and evolution revealed from high-throughput ChIP
Source: PLoS Comput Biol. 2018 Apr 23;14(4):e1006090. doi: 10.1371/journal.pcbi.1006090 (PMC5933800; doi:10.1371/journal.pcbi.1006090)
Supplement: S1 File — (GZ) [file pcbi.1006090.s009.tar.gz › DIVERSITY-master/weblogoMod/apidocs/module-tree.html]

xml version="1.0" encoding="ascii"?


Module Hierarchy


| Home | Trees | Indices | Help | WebLogo |
| --- | --- | --- | --- | --- |

 **[ Module Hierarchy
| Class Hierarchy ]**   

# Module Hierarchy

- **weblogolib**: *WebLogo (http://code.google.com/p/weblogo/) is a tool for creating sequence
  logos from biological sequence alignments.*
  - **weblogolib.\_cgi**
  - **weblogolib.\_cli**
  - **weblogolib.color**: *Color specifications using CSS2 (Cascading Style Sheet) syntax.*
  - **weblogolib.colorscheme**: *Popular color codings for nucleic and amino acids.*
  - **weblogolib.logomath**

| Home | Trees | Indices | Help | WebLogo |
| --- | --- | --- | --- | --- |

|  |  |
| --- | --- |
| Generated by Epydoc 3.0.1 on Mon Jan 30 17:27:18 2012 | http://epydoc.sourceforge.net |
