# Supplementary material for: DIVERSITY in binding, regulation, and evolution revealed from high-throughput ChIP
Source: PLoS Comput Biol. 2018 Apr 23;14(4):e1006090. doi: 10.1371/journal.pcbi.1006090 (PMC5933800; doi:10.1371/journal.pcbi.1006090)
Supplement: S1 File — (GZ) [file pcbi.1006090.s009.tar.gz › DIVERSITY-master/weblogoMod/apidocs/weblogolib-pysrc.html]

xml version="1.0" encoding="ascii"?


weblogolib


| Home | Trees | Indices | Help | WebLogo |
| --- | --- | --- | --- | --- |

Package weblogolib

# Source Code for Package weblogolib

```
   1  #!/usr/bin/env python 
   2   
   3  # -------------------------------- WebLogo -------------------------------- 
   4   
   5  #  Copyright (c) 2003-2004 The Regents of the University of California. 
   6  #  Copyright (c) 2005 Gavin E. Crooks 
   7  #  Copyright (c) 2006-2011, The Regents of the University of California, through  
   8  #  Lawrence Berkeley National Laboratory (subject to receipt of any required 
   9  #  approvals from the U.S. Dept. of Energy).  All rights reserved. 
  10   
  11  #  This software is distributed under the new BSD Open Source License. 
  12  #  <http://www.opensource.org/licenses/bsd-license.html> 
  13  # 
  14  #  Redistribution and use in source and binary forms, with or without  
  15  #  modification, are permitted provided that the following conditions are met:  
  16  # 
  17  #  (1) Redistributions of source code must retain the above copyright notice,  
  18  #  this list of conditions and the following disclaimer.  
  19  # 
  20  #  (2) Redistributions in binary form must reproduce the above copyright  
  21  #  notice, this list of conditions and the following disclaimer in the  
  22  #  documentation and or other materials provided with the distribution.  
  23  # 
  24  #  (3) Neither the name of the University of California, Lawrence Berkeley  
  25  #  National Laboratory, U.S. Dept. of Energy nor the names of its contributors  
  26  #  may be used to endorse or promote products derived from this software  
  27  #  without specific prior written permission.  
  28  # 
  29  #  THIS SOFTWARE IS PROVIDED BY THE COPYRIGHT HOLDERS AND CONTRIBUTORS "AS IS"  
  30  #  AND ANY EXPRESS OR IMPLIED WARRANTIES, INCLUDING, BUT NOT LIMITED TO, THE  
  31  #  IMPLIED WARRANTIES OF MERCHANTABILITY AND FITNESS FOR A PARTICULAR PURPOSE  
  32  #  ARE DISCLAIMED. IN NO EVENT SHALL THE COPYRIGHT OWNER OR CONTRIBUTORS BE  
  33  #  LIABLE FOR ANY DIRECT, INDIRECT, INCIDENTAL, SPECIAL, EXEMPLARY, OR  
  34  #  CONSEQUENTIAL DAMAGES (INCLUDING, BUT NOT LIMITED TO, PROCUREMENT OF  
  35  #  SUBSTITUTE GOODS OR SERVICES; LOSS OF USE, DATA, OR PROFITS; OR BUSINESS  
  36  #  INTERRUPTION) HOWEVER CAUSED AND ON ANY THEORY OF LIABILITY, WHETHER IN  
  37  #  CONTRACT, STRICT LIABILITY, OR TORT (INCLUDING NEGLIGENCE OR OTHERWISE)  
  38  #  ARISING IN ANY WAY OUT OF THE USE OF THIS SOFTWARE, EVEN IF ADVISED OF THE  
  39  #  POSSIBILITY OF SUCH DAMAGE.  
  40   
  41  # Replicates README.txt 
  42   
  43  """ 
  44  WebLogo (http://code.google.com/p/weblogo/) is a tool for creating sequence  
  45  logos from biological sequence alignments.  It can be run on the command line, 
  46  as a standalone webserver, as a CGI webapp, or as a python library. 
  47   
  48  The main WebLogo webserver is located at http://weblogo.threeplusone.com 
  49   
  50  Please consult the manual for installation instructions and more information: 
  51  (Also located in the weblogolib/htdocs subdirectory.) 
  52   
  53      http://weblogo.threeplusone.com/manual.html 
  54   
  55  For help on the command line interface run 
  56      ./weblogo --help 
  57   
  58  To build a simple logo run 
  59      ./weblogo  < cap.fa > logo0.eps 
  60       
  61  To run as a standalone webserver at localhost:8080  
  62      ./weblogo --serve 
  63   
  64  To create a logo in python code: 
  65      >>> from weblogolib import * 
  66      >>> fin = open('cap.fa') 
  67      >>> seqs = read_seq_data(fin)  
  68      >>> data = LogoData.from_seqs(seqs) 
  69      >>> options = LogoOptions() 
  70      >>> options.title = "A Logo Title" 
  71      >>> format = LogoFormat(data, options) 
  72      >>> fout = open('cap.eps', 'w')  
  73      >>> eps_formatter( data, format, fout) 
  74   
  75   
  76  -- Distribution and Modification -- 
  77  This package is distributed under the new BSD Open Source License.  
  78  Please see the LICENSE.txt file for details on copyright and licensing. 
  79  The WebLogo source code can be downloaded from  
  80  http://code.google.com/p/weblogo/ 
  81   
  82  WebLogo requires Python 2.5, 2.6 or 2.7, and the python 
  83  array package 'numpy' (http://www.scipy.org/Download) 
  84   
  85  Generating logos in PDF or bitmap graphics formats require that the ghostscript 
  86  program 'gs' be installed. Scalable Vector Graphics (SVG) format also requires  
  87  the program 'pdf2svg'. 
  88   
  89  """ 
  90   
  91  import sys 
  92  import copy 
  93  import os 
  94  from datetime import datetime 
  95  from StringIO import StringIO 
  96   
  97  from  corebio.data import rna_letters, dna_letters, amino_acid_letters 
  98   
  99   
 100  from string import Template 
 101  from subprocess import * 
 102  from corebio.utils import resource_string, resource_filename 
 103   
 104  from math import log, sqrt, exp 
 105   
 106  # Avoid 'from numpy import *' since numpy has lots of names defined 
 107  from numpy import array, asarray, float64, ones, zeros, int32,all,any, shape 
 108  import numpy as na 
 109   
 110   
 111  from color import * 
 112  from colorscheme import * 
 113  from corebio.seq import Alphabet, Seq, SeqList 
 114  from corebio import seq_io 
 115  from corebio.utils import isfloat, find_command, ArgumentError, stdrepr 
 116  from corebio.moremath import * 
 117  from corebio.data import amino_acid_composition 
 118  from corebio.seq import unambiguous_rna_alphabet, unambiguous_dna_alphabet, unambiguous_protein_alphabet 
 119   
 120  from logomath import Dirichlet 
 121   
 122   
 123  # ------ META DATA ------ 
 124   
 125  __all__ = [ 'LogoOptions',  
 126              'description',  
 127              '__version__',  
 128              'LogoFormat', 
 129              'LogoData', 
 130              'GhostscriptAPI', 
 131              'std_color_schemes', 
 132              'default_color_schemes', 
 133              'classic', 
 134              'std_units', 
 135              'std_sizes', 
 136              'std_alphabets', 
 137              'std_percentCG', 
 138              'pdf_formatter', 
 139              'jpeg_formatter', 
 140              'png_formatter', 
 141              'png_print_formatter', 
 142              'txt_formatter', 
 143              'eps_formatter', 
 144              'formatters', 
 145              'default_formatter', 
 146              'base_distribution', 
 147              'equiprobable_distribution', 
 148              'read_seq_data', 
 149              'color', 
 150              'colorscheme', 
 151              'logomath', 
 152              ] 
 153   
 154  description  = "Create sequence logos from biological sequence alignments."  
 155   
 156  __version__ = "3.2" 
 157   
 158  # These keywords are substituted by subversion. 
 159  # The date and revision will only tell the truth after a branch or tag, 
 160  # since different files in trunk will have been changed at different times 
 161  release_date ="$Date: 2011-11-07 19:14:18 -0800 (Mon, 07 Nov 2011) $".split()[1] 
 162  release_build = "$Revision: 118 $".split()[1] 
 163  release_description = "WebLogo %s (%s)" % (__version__,  release_date) 


164   
 165   
 166   
 167 -def cgi(htdocs_directory) :


168      import weblogolib._cgi 
 169      weblogolib._cgi.main(htdocs_directory)

 170           


171 -class GhostscriptAPI(object) :


172      """Interface to the command line program Ghostscript ('gs')""" 
 173       
 174      formats = ('png', 'pdf', 'jpeg') 
 175       


176 -    def __init__(self, path=None) :


177          try: 
 178              command = find_command('gs', path=path) 
 179          except EnvironmentError: 
 180              try: 
 181                  command = find_command('gswin32c.exe', path=path) 
 182              except EnvironmentError: 
 183                  raise EnvironmentError("Could not find Ghostscript on path." 
 184                  " There should be either a gs executable or a gswin32c.exe on your system's path") 
 185           
 186          self.command = command

 187       


188 -    def version(self) :


189          args = [self.command, '--version'] 
 190          try : 
 191              p = Popen(args, stdout=PIPE) 
 192              (out,err) = p.communicate()  
 193          except OSError : 
 194              raise RuntimeError("Cannot communicate with ghostscript.")   
 195          return out.strip()

 196          


197 -    def convert(self, format, fin, fout,  width,  height, resolution=300) :


198          device_map = { 'png':'png16m',  'pdf':'pdfwrite', 'jpeg':'jpeg'} 
 199          
 200          try : 
 201              device = device_map[format] 
 202          except KeyError: 
 203              raise ValueError("Unsupported format.") 
 204           
 205          args = [self.command,  
 206              "-sDEVICE=%s" % device,  
 207              "-dPDFSETTINGS=/printer", 
 208              #"-q",   # Quite: Do not dump messages to stdout. 
 209              "-sstdout=%stderr", # Redirect messages and errors to stderr 
 210              "-sOutputFile=-", # Stdout 
 211              "-dDEVICEWIDTHPOINTS=%s" % str(width),   
 212              "-dDEVICEHEIGHTPOINTS=%s" % str(height),   
 213              "-dSAFER",  # For added security 
 214              "-dNOPAUSE",] 
 215               
 216          if device != 'pdf' : 
 217              args.append("-r%s" % str(resolution) )  
 218              if resolution < 300 : # Antialias if resolution is Less than 300 DPI 
 219                  args.append("-dGraphicsAlphaBits=4") 
 220                  args.append("-dTextAlphaBits=4") 
 221                  args.append("-dAlignToPixels=0") 
 222           
 223          args.append("-")  # Read from stdin. Must be last argument. 
 224           
 225          error_msg = "Unrecoverable error : Ghostscript conversion failed " \ 
 226                      "(Invalid postscript?). %s" % " ".join(args)  
 227   
 228          source = fin.read() 
 229          try : 
 230              p = Popen(args, stdin=PIPE, stdout = PIPE, stderr= PIPE)  
 231              (out,err) = p.communicate(source)  
 232          except OSError : 
 233              raise RuntimeError(error_msg) 
 234       
 235          if p.returncode != 0 :  
 236              error_msg += '\nReturn code: %i\n' % p.returncode  
 237              if err is not None : error_msg += err 
 238              raise RuntimeError(error_msg) 
 239           
 240          print >>fout, out

 241  # end class Ghostscript 
 242   
 243   
 244  aa_composition = [ amino_acid_composition[_k] for _k in 
 245                      unambiguous_protein_alphabet] 
 246   
 247   
 248   
 249  # ------  DATA ------ 
 250   
 251  classic = ColorScheme([ 
 252      ColorGroup("G",  "orange" ), 
 253      ColorGroup("TU", "red"), 
 254      ColorGroup("C",  "blue"), 
 255      ColorGroup("A",  "green") 
 256      ] ) 
 257       
 258  std_color_schemes = {"auto":            None,   # Depends on sequence type 
 259                       "monochrome":      monochrome, 
 260                       "base pairing":    base_pairing, 
 261                       "classic":         classic, 
 262                       "hydrophobicity" : hydrophobicity,   
 263                       "chemistry" :      chemistry, 
 264                       "charge" :         charge, 
 265                       }# 
 266   
 267  default_color_schemes = { 
 268      unambiguous_protein_alphabet: hydrophobicity,  
 269      unambiguous_rna_alphabet: base_pairing, 
 270      unambiguous_dna_alphabet: base_pairing  
 271  } 
 272   
 273  std_units = { 
 274      "bits"    : 1./log(2), 
 275      "nats"    : 1., 
 276      "digits"  : 1./log(10), 
 277      "kT"      : 1., 
 278      "kJ/mol"  : 8.314472 *298.15 /1000., 
 279      "kcal/mol": 1.987 *298.15  /1000., 
 280      "probability" : None, 
 281  } 
 282   
 283  # The base stack width is set equal to 9pt Courier.  
 284  # (Courier has a width equal to 3/5 of the point size.) 
 285  # Check that can get 80 characters in journal page @small 
 286  # 40 characters in a journal column 
 287  std_sizes = { 
 288      "small" : 5.4 ,  
 289      "medium" : 5.4*2, 
 290      "large"  : 5.4*3 
 291  } 
 292               
 293  std_alphabets = { 
 294      'protein': unambiguous_protein_alphabet,  
 295      'rna': unambiguous_rna_alphabet, 
 296      'dna': unambiguous_dna_alphabet} 
 297   
 298  std_percentCG = { 
 299      'H. sapiens'    : 40., 
 300      'E. coli'       : 50.5, 
 301      'S. cerevisiae' : 38., 
 302      'C. elegans'    : 36., 
 303      'D. melanogaster': 43., 
 304      'M. musculus'   :  42., 
 305      'T. thermophilus' : 69.4, 
 306  } 


307   
 308  # Thermus thermophilus: Henne A, Bruggemann H, Raasch C, Wiezer A, Hartsch T, 
 309  # Liesegang H, Johann A, Lienard T, Gohl O, Martinez-Arias R, Jacobi C,  
 310  # Starkuviene V, Schlenczeck S, Dencker S, Huber R, Klenk HP, Kramer W,  
 311  # Merkl R, Gottschalk G, Fritz HJ: The genome sequence of the extreme  
 312  # thermophile Thermus thermophilus. 
 313  # Nat Biotechnol 2004, 22:547-53 
 314               
 315   
 316     
 317 -class LogoOptions(object) :


318      """ A container for all logo formatting options. Not all of these 
 319      are directly accessible through the CLI or web interfaces.  
 320       
 321      To display LogoOption defaults: 
 322      >>> from weblogolib import * 
 323      >>> LogoOptions() 
 324       
 325      All physical lengths are measured in points. (72 points per inch, 28.3 points per cm) 
 326         
 327      String attributes: 
 328          o creator_text      -- Embedded as comment in figures. 
 329          o logo_title              
 330          o logo_label 
 331          o unit_name         -- See std_units for options. (Default 'bits')  
 332          o yaxis_label       -- Defaults to unit_name       
 333          o xaxis_label 
 334          o fineprint         -- Defaults to WebLogo name and version 
 335           
 336      Boolean attributes: 
 337          o show_yaxis  
 338          o show_xaxis          
 339          o show_ends  
 340          o show_fineprint         
 341          o show_errorbars    -- Draw errorbars (default: False) 
 342          o show_boxes        -- Draw boxes around stack characters (default: True) 
 343          o debug             -- Draw extra graphics debugging information.  
 344          o rotate_numbers    -- Draw xaxis numbers with vertical orientation?  
 345          o scale_width       -- boolean, scale width of characters proportional to ungaps 
 346          o pad_right         -- Make a single line logo the same width as multiline logos (default: False)                            
 347                                   
 348      Other attributes: 
 349          o stacks_per_line 
 350           
 351          o yaxis_tic_interval  
 352          o yaxis_minor_tic_ratio  
 353          o yaxis_scale 
 354          o xaxis_tic_interval  
 355          o number_interval 
 356   
 357          o shrink_fraction       -- Proportional shrinkage of characters if show_boxes is true. 
 358   
 359          o errorbar_fraction  
 360          o errorbar_width_fraction  
 361          o errorbar_gray  
 362   
 363          o resolution             -- Dots per inch (default: 96). Used for bitmapped output formats 
 364           
 365          o default_color  
 366          o color_scheme  
 367           
 368          o stack_width           -- 
 369          o stack_aspect_ratio    -- Ratio of stack height to width (default: 5) 
 370   
 371          o logo_margin           -- Default: 2 pts 
 372          o stroke_width          -- Default: 0.5 pts 
 373          o tic_length            -- Default: 5 pts 
 374          o stack_margin          -- Default: 0.5 pts 
 375           
 376          o small_fontsize        -- Small text font size in points 
 377          o fontsize              -- Regular text font size in points 
 378          o title_fontsize        -- Title text font size in points 
 379          o number_fontsize       -- Font size for axis-numbers, in points. 
 380           
 381          o text_font 
 382          o logo_font 
 383          o title_font 
 384           
 385          o first_index 
 386          o logo_start 
 387          o logo_end 
 388   
 389      """        
 390   


391 -    def __init__(self, **kwargs) :


392          """ Create a new LogoOptions instance. 
 393           
 394          >>> L = LogoOptions(logo_title = "Some Title String") 
 395          >>> L.show_yaxis = False 
 396          >>> repr(L) 
 397          """ 
 398   
 399          self.alphabet = None 
 400   
 401          self.creator_text = release_description 
 402           
 403          self.logo_title = "" 
 404          self.logo_label = "" 
 405          self.stacks_per_line = 40 
 406           
 407          self.unit_name = "bits" 
 408        
 409          self.show_yaxis = True 
 410          # yaxis_lable default depends on other settings. See LogoFormat 
 411          self.yaxis_label = None 
 412          self.yaxis_tic_interval = 1. 
 413          self.yaxis_minor_tic_ratio = 5 
 414          self.yaxis_scale = None 
 415   
 416          self.show_xaxis = True 
 417          self.xaxis_label = "" 
 418          self.xaxis_tic_interval =1 
 419          self.rotate_numbers = False 
 420          self.number_interval = 5             
 421          self.show_ends = False 
 422          self.annotate = None 
 423           
 424             
 425          self.show_fineprint = True 
 426          self.fineprint =  "WebLogo "+__version__  
 427       
 428          self.show_boxes = False 
 429          self.shrink_fraction = 0.5 
 430     
 431          self.show_errorbars = True 
 432          self.errorbar_fraction = 0.90 
 433          self.errorbar_width_fraction = 0.25       
 434          self.errorbar_gray = 0.75 
 435    
 436          self.resolution  = 96.     # Dots per inch 
 437              
 438          self.default_color = Color.by_name("black")     
 439          self.color_scheme = None 
 440          #self.show_color_key = False # NOT yet implemented 
 441           
 442          self.debug = False 
 443           
 444          self.logo_margin = 2 
 445          self.stroke_width = 0.5 
 446          self.tic_length = 5 
 447           
 448          self.stack_width = std_sizes["medium"]         
 449          self.stack_aspect_ratio = 5 
 450           
 451          self.stack_margin = 0.5 
 452          self.pad_right = False   
 453   
 454          self.small_fontsize = 6 
 455          self.fontsize = 10 
 456          self.title_fontsize = 12 
 457          self.number_fontsize = 8 
 458   
 459          self.text_font    = "ArialMT" 
 460          self.logo_font    = "Arial-BoldMT" 
 461          self.title_font   = "ArialMT" 
 462   
 463          self.first_index = 1         
 464          self.logo_start = None       
 465          self.logo_end=None           
 466          self.scale_width = True 
 467   
 468          from corebio.utils import update 
 469          update(self, **kwargs)

 470   


471 -    def __repr__(self) :


472          from corebio.util import stdrepr 
 473          return stdrepr( self)

 474   


475 -    def __repr__(self) :


476          attributes = vars(self).keys() 
 477          attributes.sort() 
 478          return stdrepr(self, attributes )

 479    


480  # End class LogoOptions 
 481   
 482   
 483           
 484   
 485 -class LogoFormat(LogoOptions) :

 486      """ Specifies the format of the logo. Requires LogoData and LogoOptions  
 487      objects. 
 488       
 489      >>> data = LogoData.from_seqs(seqs ) 
 490      >>> options = LogoOptions() 
 491      >>> options.title = "A Logo Title" 
 492      >>> format = LogoFormat(data, options)  
 493       
 494      Raises an ArgumentError if arguments are invalid. 
 495      """ 


496 -    def __init__(self, data, options= None) :


497          """ Create a new LogoFormat instance. 
 498           
 499          """ 
 500          LogoOptions.__init__(self) 
 501           
 502          if options is not None : 
 503              self.__dict__.update(options.__dict__) 
 504                    
 505          self.alphabet = data.alphabet 
 506          self.seqlen = data.length 
 507           
 508           
 509          # Derived parameters. 
 510          self.show_title = False 
 511          self.show_xaxis_label = False 
 512          self.yaxis_minor_tic_interval = None 
 513          self.lines_per_logo       = None 
 514          self.char_width       = None        # Maximum character width. Stack width minus margins.  
 515          self.line_margin_left = None 
 516          self.line_margin_right    = None 
 517          self.line_margin_bottom = None 
 518          self.line_margin_top = None 
 519          self.title_height = None 
 520          self.xaxis_label_height = None 
 521          self.line_height = None 
 522          self.line_width = None 
 523          self.logo_height = None 
 524          self.logo_width = None 
 525          self.creation_date = None 
 526          self.end_type = None 
 527   
 528          self.stack_height = self.stack_width * self.stack_aspect_ratio 
 529           
 530          # Attribute to test, test, error message 
 531          arg_conditions = ( 
 532              ("stacks_per_line",     lambda x: x>0 ,     "Stacks per line must be positive."), 
 533              ("stack_width",         lambda x: x>0.0,    "Stack width must be greater than zero."), 
 534              ("stack_aspect_ratio" , lambda x: x>0,    "Stack aspect ratio must be greater than zero."), 
 535              ("fontsize" ,           lambda x: x>0 ,     "Font sizes must be positive."), 
 536              ("small_fontsize" ,     lambda x: x>0 ,     "Font sizes must be positive."), 
 537              ("title_fontsize" ,     lambda x: x>0 ,     "Font sizes must be positive."), 
 538              ("errorbar_fraction" ,  lambda x: x>=0.0 and x<=1.0,  
 539                  "The visible fraction of the error bar must be between zero and one."), 
 540              ("yaxis_tic_interval" , lambda x: x>=0.0 , "The yaxis tic interval cannot be negative."),                                     
 541              ("yaxis_minor_tic_interval" , lambda x: not (x and x<0.0) , "Distances cannot be negative."), 
 542              ("xaxis_tic_interval" , lambda x: x>0.0 ,   "Tic interval must be greater than zero."), 
 543              ("number_interval" ,    lambda x: x>0.0 ,      "Invalid interval between numbers."), 
 544              ("shrink_fraction" ,    lambda x: x>=0.0 and x<=1.0 , "Invalid shrink fraction."), 
 545              ("stack_margin" ,       lambda x: x>0.0 , "Invalid stack margin."), 
 546              ("logo_margin" ,        lambda x: x>0.0 , "Invalid logo margin."), 
 547              ("stroke_width",       lambda x: x>0.0 , "Invalid stroke width."), 
 548              ("tic_length" ,         lambda x: x>0.0 , "Invalid tic length."), 
 549          ) 
 550           
 551          # Run arguments tests. The second, attribute argument to the ArgumentError is  
 552          # used by the UI to provide user feedback. 
 553          # FIXME: More validation         
 554          for test in arg_conditions : 
 555              if not test[1]( getattr(self,test[0]) ) : raise ArgumentError(test[2], test[0]) 
 556      
 557            
 558          # Inclusive upper and lower bounds 
 559          # FIXME: Validate here. Move from eps_formatter         
 560          if self.logo_start is  None: self.logo_start = self.first_index 
 561           
 562          if self.logo_end is  None :  
 563              self.logo_end = self.seqlen + self.first_index -1  
 564           
 565          self.total_stacks = self.logo_end - self.logo_start +1 
 566   
 567          if self.logo_start - self.first_index <0 : 
 568              raise ArgumentError( 
 569                  "Logo range extends before start of available sequence.", 
 570                  'logo_range') 
 571           
 572          if self.logo_end - self.first_index  >= self.seqlen  :  
 573              raise ArgumentError( 
 574                  "Logo range extends beyond end of available sequence.", 
 575                  'logo_range') 
 576       
 577          if self.logo_title      : self.show_title = True 
 578          if not self.fineprint   : self.show_fineprint = False 
 579          if self.xaxis_label     : self.show_xaxis_label = True 
 580   
 581          if self.yaxis_label is None :  
 582              self.yaxis_label = self.unit_name 
 583           
 584          if self.yaxis_label :  
 585              self.show_yaxis_label = True 
 586          else : 
 587              self.show_yaxis_label = False 
 588              self.show_ends = False 
 589                 
 590          if not self.yaxis_scale : 
 591              conversion_factor = std_units[self.unit_name] 
 592              if conversion_factor : 
 593                  self.yaxis_scale=log(len(self.alphabet))*conversion_factor 
 594              else : 
 595                  self.yaxis_scale=1.0    # probability units 
 596   
 597          if self.yaxis_scale<=0.0 :  
 598              raise ArgumentError("Invalid yaxis scale", 'yaxis_scale',) 
 599   
 600          if self.yaxis_tic_interval >= self.yaxis_scale: 
 601              self.yaxis_tic_interval /= 2.  
 602   
 603          self.yaxis_minor_tic_interval \ 
 604              = float(self.yaxis_tic_interval)/self.yaxis_minor_tic_ratio 
 605                         
 606          if self.color_scheme is None : 
 607              if self.alphabet in default_color_schemes : 
 608                  self.color_scheme = default_color_schemes[self.alphabet] 
 609              else : 
 610                  self.color_scheme = monochrome 
 611   
 612          self.lines_per_logo = 1+ ( (self.total_stacks-1) / self.stacks_per_line) 
 613       
 614          if self.lines_per_logo==1 and not self.pad_right: 
 615              self.stacks_per_line = min(self.stacks_per_line, self.total_stacks) 
 616   
 617          self.char_width = self.stack_width - 2* self.stack_margin 
 618       
 619       
 620          if self.show_yaxis : 
 621              self.line_margin_left = self.fontsize * 3.0 
 622          else : 
 623              self.line_margin_left = 0 
 624   
 625          if self.show_ends : 
 626              self.line_margin_right = self.fontsize *1.5  
 627          else : 
 628              self.line_margin_right = self.fontsize              
 629   
 630          if self.show_xaxis : 
 631              if self.rotate_numbers : 
 632                  self.line_margin_bottom = self.number_fontsize *2.5 
 633              else: 
 634                  self.line_margin_bottom = self.number_fontsize *1.5 
 635          else : 
 636              self.line_margin_bottom = 4 
 637   
 638          self.line_margin_top = 4 
 639       
 640          if self.show_title : 
 641              self.title_height = self.title_fontsize  
 642          else : 
 643              self.title_height = 0 
 644   
 645          self.xaxis_label_height =0. 
 646          if self.show_xaxis_label : 
 647              self.xaxis_label_height += self.fontsize 
 648          if self.show_fineprint : 
 649              self.xaxis_label_height += self.small_fontsize 
 650   
 651          self.line_height = (self.stack_height + self.line_margin_top +     
 652                              self.line_margin_bottom ) 
 653          self.line_width  = (self.stack_width*self.stacks_per_line +  
 654                              self.line_margin_left + self.line_margin_right ) 
 655   
 656          self.logo_height = int(2*self.logo_margin + self.title_height \ 
 657              + self.xaxis_label_height + self.line_height*self.lines_per_logo)  
 658          self.logo_width = int(2*self.logo_margin + self.line_width ) 
 659   
 660   
 661          self.creation_date = datetime.now().isoformat(' ')  
 662   
 663          end_type = '-' 
 664          end_types = { 
 665              unambiguous_protein_alphabet: 'p',  
 666              unambiguous_rna_alphabet: '-', 
 667              unambiguous_dna_alphabet: 'd'  
 668          } 
 669          if self.show_ends and self.alphabet in end_types: 
 670              end_type = end_types[self.alphabet] 
 671          self.end_type = end_type 
 672   
 673           
 674          if self.annotate is None : 
 675              self.annotate = [] 
 676              for i in range(self.seqlen): 
 677                  index = i + self.first_index 
 678                  if index % self.number_interval == 0 :  
 679                      self.annotate.append( "%d"%index) 
 680                  else : 
 681                      self.annotate.append("") 
 682                       
 683          if len(self.annotate)!=self.seqlen  : 
 684              raise ArgumentError( 
 685                    "Annotations must be same length as sequences.", 
 686                    'annotate')

 687               


688   
 689      # End __init__ 
 690  # End class LogoFormat 
 691   
 692   
 693   
 694  # ------ Logo Formaters ------ 
 695  # Each formatter is a function f(LogoData, LogoFormat, output file). 
 696  # that draws a representation of the logo into the given file. 
 697  # The main graphical formatter is eps_formatter. A mapping 'formatters' 
 698  # containing all available formatters is located after the formatter 
 699  # definitions.  
 700   
 701 -def pdf_formatter(data, format, fout) :


702      """ Generate a logo in PDF format.""" 
 703       
 704      feps = StringIO() 
 705      eps_formatter(data, format, feps) 
 706      feps.seek(0) 
 707       
 708      gs = GhostscriptAPI()     
 709      gs.convert('pdf', feps, fout, format.logo_width, format.logo_height)

 710   


711   
 712 -def _bitmap_formatter(data, format, fout, device) :


713      feps = StringIO() 
 714      eps_formatter(data, format, feps) 
 715      feps.seek(0) 
 716       
 717      gs = GhostscriptAPI()     
 718      gs.convert(device, feps, fout,  
 719          format.logo_width, format.logo_height, format.resolution)

 720   


721   
 722 -def jpeg_formatter(data, format, fout) :

 723      """ Generate a logo in JPEG format.""" 
 724      _bitmap_formatter(data, format, fout, device="jpeg") 
 725   


726 -def svg_formatter(data, format, fout) :

 727      """ Generate a logo in Scalable Vector Graphics (SVG) format. 
 728      Requires the program 'pdf2svg' be installed. 
 729      """ 
 730   
 731      fpdf = StringIO() 
 732      pdf_formatter(data, format, fpdf) 
 733      fpdf.seek(0) 
 734       
 735      try: 
 736              command = find_command('pdf2svg')  
 737      except EnvironmentError: 
 738          raise EnvironmentError("Scalable Vector Graphics (SVG) format requires the program 'pdf2svg'. " 
 739                                  "Cannot find 'pdf2svg' on search path.") 
 740   
 741      import tempfile, os 
 742      fpdfi, fname_pdf = tempfile.mkstemp(suffix=".pdf") 
 743      fsvgi, fname_svg = tempfile.mkstemp(suffix=".svg") 
 744      try: 
 745           
 746          fpdf2 = open(fname_pdf, 'w') 
 747          fpdf2.write(fpdf.getvalue() ) 
 748          fpdf2.seek(0) 
 749     
 750          args = [command, fname_pdf, fname_svg] 
 751          p = Popen(args) 
 752          (out,err) = p.communicate()  
 753   
 754          fsvg = open(fname_svg) 
 755          fout.write(fsvg.read()) 
 756      finally: 
 757          os.remove(fname_svg) 
 758          os.remove(fname_pdf) 
 759   


760   
 761 -def png_formatter(data, format, fout) :

 762      """ Generate a logo in PNG format.""" 
 763      _bitmap_formatter(data, format, fout, device="png") 
 764   


765   
 766 -def png_print_formatter(data, format, fout) :

 767      """ Generate a logo in PNG format with print quality (600 DPI) resolution.""" 
 768      format.resolution = 600 
 769      _bitmap_formatter(data, format, fout, device="png") 
 770   


771   
 772 -def txt_formatter( logodata, format, fout) :


773      """ Create a text representation of the logo data.  
 774      """ 
 775      print >>fout, str(logodata)

 776   


777      
 778   
 779       
 780 -def eps_formatter( logodata, format, fout) :


781      """ Generate a logo in Encapsulated Postscript (EPS)""" 
 782       
 783      substitutions = {} 
 784      from_format =[ 
 785          "creation_date",    "logo_width",           "logo_height",       
 786          "lines_per_logo",   "line_width",           "line_height", 
 787          "line_margin_right","line_margin_left",     "line_margin_bottom", 
 788          "line_margin_top",  "title_height",         "xaxis_label_height", 
 789          "creator_text",     "logo_title",           "logo_margin", 
 790          "stroke_width",     "tic_length",            
 791          "stacks_per_line",  "stack_margin", 
 792          "yaxis_label",      "yaxis_tic_interval",   "yaxis_minor_tic_interval", 
 793          "xaxis_label",      "xaxis_tic_interval",   "number_interval", 
 794          "fineprint",        "shrink_fraction",      "errorbar_fraction", 
 795          "errorbar_width_fraction", 
 796          "errorbar_gray",    "small_fontsize",       "fontsize", 
 797          "title_fontsize",   "number_fontsize",      "text_font", 
 798          "logo_font",        "title_font",           
 799          "logo_label",       "yaxis_scale",          "end_type", 
 800          "debug",            "show_title",           "show_xaxis", 
 801          "show_xaxis_label", "show_yaxis",           "show_yaxis_label", 
 802          "show_boxes",       "show_errorbars",       "show_fineprint", 
 803          "rotate_numbers",   "show_ends",            "stack_height", 
 804          "stack_width" 
 805          ] 
 806      
 807      for s in from_format : 
 808          substitutions[s] = getattr(format,s) 
 809   
 810      substitutions["shrink"] = str(format.show_boxes).lower() 
 811   
 812   
 813      # --------- COLORS -------------- 
 814      def format_color(color): 
 815          return  " ".join( ("[",str(color.red) , str(color.green),  
 816              str(color.blue), "]"))

 817   
 818      substitutions["default_color"] = format_color(format.default_color) 
 819   
 820      colors = []   
 821      for group in format.color_scheme.groups : 
 822          cf = format_color(group.color) 
 823          for s in group.symbols : 
 824              colors.append( "  ("+s+") " + cf ) 
 825      substitutions["color_dict"] = "\n".join(colors) 
 826           
 827      data = [] 
 828       
 829      # Unit conversion. 'None' for probability units 
 830      conv_factor = std_units[format.unit_name] 
 831       
 832      data.append("StartLine") 
 833       
 834   
 835      seq_from = format.logo_start- format.first_index 
 836      seq_to = format.logo_end - format.first_index +1 
 837   
 838      # seq_index : zero based index into sequence data 
 839      # logo_index : User visible coordinate, first_index based 
 840      # stack_index : zero based index of visible stacks 
 841      for seq_index in range(seq_from, seq_to) : 
 842          logo_index = seq_index + format.first_index  
 843          stack_index = seq_index - seq_from 
 844           
 845          if stack_index!=0 and (stack_index % format.stacks_per_line) ==0 : 
 846              data.append("") 
 847              data.append("EndLine") 
 848              data.append("StartLine") 
 849              data.append("") 
 850           
 851          data.append("(%s) StartStack" % format.annotate[seq_index] ) 
 852   
 853          if conv_factor:  
 854              stack_height = logodata.entropy[seq_index] * std_units[format.unit_name] 
 855          else : 
 856              stack_height = 1.0 # Probability 
 857   
 858          s = zip(logodata.counts[seq_index], logodata.alphabet) 
 859          def mycmp( c1, c2 ) : 
 860              # Sort by frequency. If equal frequency then reverse alphabetic 
 861              if c1[0] == c2[0] : return cmp(c2[1], c1[1]) 
 862              return cmp(c1[0], c2[0]) 
 863           
 864          s.sort(mycmp) 
 865   
 866          C = float(sum(logodata.counts[seq_index]))  
 867          if C > 0.0 : 
 868              fraction_width = 1.0 
 869              if format.scale_width : 
 870                  fraction_width = logodata.weight[seq_index]  
 871              # print >>sys.stderr, fraction_width 
 872              for c in s: 
 873                  data.append(" %f %f (%s) ShowSymbol" % (fraction_width, c[0]*stack_height/C, c[1]) ) 
 874   
 875          # Draw error bar on top of logo. Replaced by DrawErrorbarFirst above. 
 876          if logodata.entropy_interval is not None and conv_factor: 
 877              low, high = logodata.entropy_interval[seq_index] 
 878              center = logodata.entropy[seq_index] 
 879   
 880              down = (center - low) * conv_factor 
 881              up   = (high - center) * conv_factor 
 882              data.append(" %f %f DrawErrorbar" % (down, up) ) 
 883               
 884          data.append("EndStack") 
 885          data.append("") 
 886                  
 887      data.append("EndLine") 
 888      substitutions["logo_data"] = "\n".join(data)   
 889   
 890   
 891      # Create and output logo 
 892      template = resource_string( __name__, 'template.eps', __file__) 
 893      logo = Template(template).substitute(substitutions) 
 894      print >>fout, logo 
 895    
 896   
 897  # map between output format names and logo   
 898  formatters = { 
 899      'eps': eps_formatter,  
 900      'pdf': pdf_formatter, 
 901      'png': png_formatter, 
 902      'png_print' : png_print_formatter, 
 903      'jpeg'  : jpeg_formatter, 
 904      'svg'   : svg_formatter, 
 905      'logodata' : txt_formatter,           
 906      }      
 907       
 908  default_formatter = eps_formatter 


909   
 910   
 911   
 912   
 913   
 914 -def parse_prior(composition, alphabet, weight=None) :


915      """ Parse a description of the expected monomer distribution of a sequence. 
 916       
 917      Valid compositions: 
 918       
 919      - None or 'none' :      No composition sepecified  
 920      - 'auto' or 'automatic': Use the typical average distribution 
 921                              for proteins and an equiprobable distribution for 
 922                              everything else.     
 923      - 'equiprobable' :      All monomers have the same probability. 
 924      - a percentage, e.g. '45%' or a fraction '0.45': 
 925                              The fraction of CG bases for nucleotide alphabets 
 926      - a species name, e.g. 'E. coli', 'H. sapiens' : 
 927                              Use the average CG percentage for the specie's       
 928                              genome. 
 929      - An explicit distribution,  e.g. {'A':10, 'C':40, 'G':40, 'T':10} 
 930      """ 
 931      if composition is None: return None 
 932      comp = composition.strip() 
 933       
 934      if comp.lower() == 'none': return None 
 935       
 936      if weight is None and alphabet is not None: weight = float(len(alphabet)) 
 937       
 938      if comp.lower() == 'equiprobable' : 
 939          prior = weight * equiprobable_distribution(len(alphabet))  
 940      elif comp.lower() == 'auto' or comp.lower() == 'automatic': 
 941          if alphabet == unambiguous_protein_alphabet : 
 942              prior =  weight * asarray(aa_composition, float64) 
 943          else : 
 944              prior = weight * equiprobable_distribution(len(alphabet))  
 945       
 946      elif comp in std_percentCG : 
 947          prior = weight * base_distribution(std_percentCG[comp]) 
 948   
 949      elif comp[-1] == '%' : 
 950          prior = weight * base_distribution( float(comp[:-1])) 
 951   
 952      elif isfloat(comp) : 
 953          prior = weight * base_distribution( float(comp)*100. ) 
 954   
 955      elif composition[0] == '{' and composition[-1] == '}' :  
 956          explicit = composition[1: -1] 
 957          explicit = explicit.replace(',',' ').replace("'", ' ').replace('"',' ').replace(':', ' ').split() 
 958           
 959          if len(explicit) != len(alphabet)*2 : 
 960              #print explicit 
 961              raise ValueError("Explicit prior does not match length of alphabet") 
 962          prior = - ones(len(alphabet), float64)  
 963          try : 
 964              for r in range(len(explicit)/2) : 
 965                  letter = explicit[r*2] 
 966                  index = alphabet.ord(letter) 
 967                  value = float(explicit[r*2 +1]) 
 968                  prior[index] = value 
 969          except ValueError : 
 970              raise ValueError("Cannot parse explicit composition") 
 971       
 972          if any(prior==-1.) : 
 973              raise ValueError("Explicit prior does not match alphabet")  
 974          prior/= sum(prior) 
 975          prior *= weight 
 976           
 977           
 978      else :  
 979          raise ValueError("Unknown or malformed composition: %s"%composition) 
 980       
 981      if len(prior) != len(alphabet) : 
 982          raise ValueError( 
 983              "The sequence alphabet and composition are incompatible.") 
 984      return prior

 985   


986       
 987 -def base_distribution(percentCG) :


988      A = (1. - (percentCG/100.))/2. 
 989      C = (percentCG/100.)/2. 
 990      G = (percentCG/100.)/2. 
 991      T = (1. - (percentCG/100))/2. 
 992      return asarray((A,C,G,T), float64)

 993   


994 -def equiprobable_distribution( length) :


995      return ones( (length), float64) /length

 996     


997   
 998 -def read_seq_data(fin,  
 999                  input_parser=seq_io.read,  
1000                  alphabet=None,  
1001                  ignore_lower_case=False,  
1002                  max_file_size=0):


1003      """ Read sequence data from the input stream and return a seqs object.  
1004       
1005      The environment variable WEBLOGO_MAX_FILE_SIZE overides the max_file_size argument. 
1006      Used to limit the load on the WebLogo webserver. 
1007      """ 
1008   
1009      max_file_size =int(os.environ.get("WEBLOGO_MAX_FILE_SIZE", max_file_size)) 
1010   
1011      # If max_file_size is set, or if fin==stdin (which is non-seekable), we 
1012      # read the data and replace fin with a StringIO object.  
1013      if(max_file_size>0) : 
1014          data = fin.read(max_file_size) 
1015          more_data = fin.read(2) 
1016          if more_data != "" : 
1017              raise IOError("File exceeds maximum allowed size: %d bytes"  % max_file_size)  
1018          fin = StringIO(data) 
1019      elif fin == sys.stdin: 
1020          fin = StringIO(fin.read()) 
1021           
1022      seqs = input_parser(fin) 
1023   
1024      if seqs is None or len(seqs) ==0 : 
1025          raise ValueError("Please provide a multiple sequence alignment") 
1026       
1027      if ignore_lower_case : 
1028          # Case is significant. Do not count lower case letters. 
1029          for i,s in enumerate(seqs) : 
1030              seqs[i] = s.mask() 
1031   
1032      # Add alphabet to seqs. 
1033      if alphabet : 
1034          seqs.alphabet = Alphabet(alphabet)  
1035      else : 
1036          seqs.alphabet = Alphabet.which(seqs) 
1037      return seqs

1038   


1039   
1040       
1041 -class LogoData(object) :


1042      """The data needed to generate a sequence logo. 
1043          
1044      - alphabet  
1045      - length 
1046      - counts  -- An array of character counts 
1047      - entropy -- The relative entropy of each column 
1048      - entropy_interval -- entropy confidence interval 
1049       """ 
1050        


1051 -    def __init__(self, length=None, alphabet = None, counts =None,  
1052              entropy =None, entropy_interval = None, weight=None) :


1053          """Creates a new LogoData object""" 
1054          self.length = length 
1055          self.alphabet = alphabet 
1056          self.counts = counts 
1057          self.entropy = entropy 
1058          self.entropy_interval = entropy_interval 
1059          self.weight = weight

1060           
1061   
1062      @classmethod     


1063 -    def from_counts(cls, alphabet, counts, prior= None):


1064          """Build a LogoData object from counts.""" 
1065          # Counts is a Motif object? 
1066          #counts = counts.array 
1067           
1068          seq_length, A = counts.shape 
1069           
1070          if prior is not None: prior = array(prior, float64) 
1071           
1072          if prior is None : 
1073              R = log(A) 
1074              ent = zeros(  seq_length, float64) 
1075              entropy_interval = None     
1076              for i in range (0, seq_length) : 
1077                  C = sum(counts[i])  
1078                  #FIXME: fixup corebio.moremath.entropy()? 
1079                  if C == 0 : 
1080                      ent[i] = 0.0 
1081                  else : 
1082                      ent[i] = R - entropy(counts[i]) 
1083          else : 
1084              ent = zeros(  seq_length, float64) 
1085              entropy_interval = zeros( (seq_length,2) , float64) 
1086           
1087              R = log(A) 
1088               
1089              for i in range (0, seq_length) : 
1090                  alpha = array(counts[i] , float64) 
1091                  alpha += prior 
1092                   
1093                  posterior = Dirichlet(alpha) 
1094                  ent[i] = posterior.mean_relative_entropy(prior/sum(prior))  
1095                  entropy_interval[i][0], entropy_interval[i][1] = \ 
1096                      posterior.interval_relative_entropy(prior/sum(prior), 0.95)  
1097    
1098          weight = array( na.sum(counts,axis=1) , float)  
1099          weight /= max(weight) 
1100    
1101          return cls(seq_length, alphabet, counts, ent, entropy_interval, weight)

1102   
1103   
1104      @classmethod     


1105 -    def from_seqs(cls, seqs, prior= None):


1106          """Build a LogoData object from a SeqList, a list of sequences.""" 
1107          # --- VALIDATE DATA --- 
1108          # check that at least one sequence of length at least 1 long 
1109          if len(seqs)==0 or len(seqs[0]) ==0: 
1110              raise ValueError("No sequence data found.")    
1111       
1112          # Check sequence lengths     
1113          seq_length = len(seqs[0]) 
1114          for i,s in enumerate(seqs) : 
1115              #print i,s, len(s) 
1116              #TODO: Redundant? Should be checked in SeqList? 
1117              if seq_length != len(s) : 
1118                  raise ArgumentError( 
1119      "Sequence number %d differs in length from the previous sequences" % (i+1) , 
1120                          'sequences') 
1121   
1122          # FIXME: Check seqs.alphabet? 
1123           
1124          counts = seqs.profile() 
1125          return cls.from_counts(seqs.alphabet, counts, prior)

1126   
1127   


1128 -    def __str__(self) :


1129          out = StringIO() 
1130          print >>out, '## LogoData' 
1131          print >>out, '# First column is position number, counting from zero' 
1132          print >>out, '# Subsequent columns are raw symbol counts' 
1133          print >>out, '# Entropy is mean entropy measured in nats.'  
1134          print >>out, '# Low and High are the 95% confidence limits.' 
1135          print >>out, '# Weight is the fraction of non-gap symbols in the column.' 
1136          print >>out, '#\t' 
1137          print >>out, '#\t', 
1138          for a in self.alphabet : 
1139              print >>out, a, '\t', 
1140          print >>out, 'Entropy\tLow\tHigh\tWeight' 
1141           
1142          for i in range(self.length) : 
1143              print >>out, i+1, '\t', 
1144              for c in self.counts[i] : print >>out, c, '\t', 
1145              print >>out, "%6.4f" % self.entropy[i], '\t', 
1146              if self.entropy_interval is not None: 
1147                  print >>out, "%6.4f" % self.entropy_interval[i][0], '\t',  
1148                  print >>out, "%6.4f" % self.entropy_interval[i][1], '\t', 
1149              else : 
1150                  print >>out, '\t','\t', 
1151              if self.weight is not None : 
1152                  print >>out, "%6.4f" % self.weight[i], 
1153              print >>out, '' 
1154          print >>out, '# End LogoData' 
1155           
1156          return out.getvalue()

1157
```

  


| Home | Trees | Indices | Help | WebLogo |
| --- | --- | --- | --- | --- |

|  |  |
| --- | --- |
| Generated by Epydoc 3.0.1 on Mon Jan 30 17:27:19 2012 | http://epydoc.sourceforge.net |
