# Supplementary material for: DIVERSITY in binding, regulation, and evolution revealed from high-throughput ChIP
Source: PLoS Comput Biol. 2018 Apr 23;14(4):e1006090. doi: 10.1371/journal.pcbi.1006090 (PMC5933800; doi:10.1371/journal.pcbi.1006090)
Supplement: S1 File — (GZ) [file pcbi.1006090.s009.tar.gz › DIVERSITY-master/weblogoMod/apidocs/weblogolib._cgi-module.html]

xml version="1.0" encoding="ascii"?


weblogolib.\_cgi


| Home | Trees | Indices | Help | WebLogo |
| --- | --- | --- | --- | --- |

Package weblogolib ::
Module \_cgi

# Module \_cgi

source code


|  |  |
| --- | --- |
| Classes | |
|  | Field  A representation of an HTML form field. |


Functions

|  |  |
| --- | --- |
| resource\_string(resource, basefilename) | source code |

|  |  |
| --- | --- |
| string\_or\_none(value) | source code |

|  |  |
| --- | --- |
| truth(value) | source code |

|  |  |
| --- | --- |
| int\_or\_none(value) | source code |

|  |  |
| --- | --- |
| float\_or\_none(value) | source code |

|  |  |
| --- | --- |
| main(htdocs\_directory=None) | source code |

|  |  |
| --- | --- |
| send\_form(controls, errors=[], htdocs\_directory=None) | source code |


|  |  |
| --- | --- |
| Variables | |
|  | mime\_type = `{'eps': 'application/postscript', 'pdf': 'applicat...` |
|  | extension = `{'eps': 'eps', 'pdf': 'pdf', 'png': 'png', 'svg': ...` |
|  | alphabets = `{'alphabet_auto': None, 'alphabet_protein': weblog...` |
|  | color\_schemes = `{}` |
|  | composition = `{'comp_none': 'none', 'comp_auto': 'auto', 'comp...` |


Variables Details

### mime\_type

Value:
:   ```
    {'eps': 'application/postscript', 'pdf': 'application/pdf', 'svg': 'im
    age/svg+xml', 'png': 'image/png', 'png_print': 'image/png', 'logodata'
    : 'text/plain', 'jpeg': 'image/jpeg',}
    ```

### extension

Value:
:   ```
    {'eps': 'eps', 'pdf': 'pdf', 'png': 'png', 'svg': 'svg', 'png_print': 
    'png', 'logodata': 'txt', 'jpeg': 'jpeg'}
    ```

### alphabets

Value:
:   ```
    {'alphabet_auto': None, 'alphabet_protein': weblogolib.unambiguous_pro
    tein_alphabet, 'alphabet_rna': weblogolib.unambiguous_rna_alphabet, 'a
    lphabet_dna': weblogolib.unambiguous_dna_alphabet}
    ```

### composition

Value:
:   ```
    {'comp_none': 'none', 'comp_auto': 'auto', 'comp_equiprobable': 'equip
    robable', 'comp_CG': 'percentCG', 'comp_Celegans': 'C. elegans', 'comp
    _Dmelanogaster': 'D. melanogaster', 'comp_Ecoli': 'E. coli', 'comp_Hsa
    piens': 'H. sapiens', 'comp_Mmusculus': 'M. musculus', 'comp_Scerevisi
    ae': 'S. cerevisiae'}
    ```

  


| Home | Trees | Indices | Help | WebLogo |
| --- | --- | --- | --- | --- |

|  |  |
| --- | --- |
| Generated by Epydoc 3.0.1 on Mon Jan 30 17:27:18 2012 | http://epydoc.sourceforge.net |
