# Supplementary material for: DIVERSITY in binding, regulation, and evolution revealed from high-throughput ChIP
Source: PLoS Comput Biol. 2018 Apr 23;14(4):e1006090. doi: 10.1371/journal.pcbi.1006090 (PMC5933800; doi:10.1371/journal.pcbi.1006090)
Supplement: S1 File — (GZ) [file pcbi.1006090.s009.tar.gz › DIVERSITY-master/weblogoMod/apidocs/weblogolib._cgi-pysrc.html]

xml version="1.0" encoding="ascii"?


weblogolib.\_cgi


| Home | Trees | Indices | Help | WebLogo |
| --- | --- | --- | --- | --- |

Package weblogolib ::
Module \_cgi

# Source Code for Module weblogolib.\_cgi

```
  1  #!/usr/bin/env python 
  2   
  3  #  Copyright (c) 2003-2004 The Regents of the University of California. 
  4  #  Copyright (c) 2005 Gavin E. Crooks 
  5  #  Copyright (c) 2006, The Regents of the University of California, through  
  6  #  Lawrence Berkeley National Laboratory (subject to receipt of any required 
  7  #  approvals from the U.S. Dept. of Energy).  All rights reserved. 
  8   
  9  #  This software is distributed under the new BSD Open Source License. 
 10  #  <http://www.opensource.org/licenses/bsd-license.html> 
 11  # 
 12  #  Redistribution and use in source and binary forms, with or without  
 13  #  modification, are permitted provided that the following conditions are met:  
 14  # 
 15  #  (1) Redistributions of source code must retain the above copyright notice,  
 16  #  this list of conditions and the following disclaimer.  
 17  # 
 18  #  (2) Redistributions in binary form must reproduce the above copyright  
 19  #  notice, this list of conditions and the following disclaimer in the  
 20  #  documentation and or other materials provided with the distribution.  
 21  # 
 22  #  (3) Neither the name of the University of California, Lawrence Berkeley  
 23  #  National Laboratory, U.S. Dept. of Energy nor the names of its contributors  
 24  #  may be used to endorse or promote products derived from this software  
 25  #  without specific prior written permission.  
 26  # 
 27  #  THIS SOFTWARE IS PROVIDED BY THE COPYRIGHT HOLDERS AND CONTRIBUTORS "AS IS"  
 28  #  AND ANY EXPRESS OR IMPLIED WARRANTIES, INCLUDING, BUT NOT LIMITED TO, THE  
 29  #  IMPLIED WARRANTIES OF MERCHANTABILITY AND FITNESS FOR A PARTICULAR PURPOSE  
 30  #  ARE DISCLAIMED. IN NO EVENT SHALL THE COPYRIGHT OWNER OR CONTRIBUTORS BE  
 31  #  LIABLE FOR ANY DIRECT, INDIRECT, INCIDENTAL, SPECIAL, EXEMPLARY, OR  
 32  #  CONSEQUENTIAL DAMAGES (INCLUDING, BUT NOT LIMITED TO, PROCUREMENT OF  
 33  #  SUBSTITUTE GOODS OR SERVICES; LOSS OF USE, DATA, OR PROFITS; OR BUSINESS  
 34  #  INTERRUPTION) HOWEVER CAUSED AND ON ANY THEORY OF LIABILITY, WHETHER IN  
 35  #  CONTRACT, STRICT LIABILITY, OR TORT (INCLUDING NEGLIGENCE OR OTHERWISE)  
 36  #  ARISING IN ANY WAY OUT OF THE USE OF THIS SOFTWARE, EVEN IF ADVISED OF THE  
 37  #  POSSIBILITY OF SUCH DAMAGE.  
 38   
 39  import sys 
 40  import cgi as cgilib 
 41  import cgitb; cgitb.enable() 
 42   
 43  from StringIO import StringIO 
 44  from color import * 
 45  from colorscheme import ColorScheme, ColorGroup 
 46   
 47  import weblogolib 
 48  from corebio.utils import * 
 49  from string import Template 
 50   
 51   
 52  # TODO: Check units 
 53   
 54  # TODO: In WebLogo2: why slash create.cgi? I think this was a workaround 
 55  # for some browser quirk 
 56  #<form method="post" action="/create.cgi" enctype="multipart/form-data"> 
 57   
 58   
 59  # Should replace with corebio.utils?     


60 -def resource_string(resource, basefilename) :


61      import os 
 62      fn =  os.path.join(os.path.dirname(basefilename), resource) 
 63      return open( fn ).read()

 64   
 65  mime_type = { 
 66      'eps': 'application/postscript',  
 67      'pdf': 'application/pdf', 
 68      'svg': 'image/svg+xml', 
 69      'png': 'image/png', 
 70      'png_print': 'image/png',     
 71      'logodata' : 'text/plain',        
 72      'jpeg'  : 'image/jpeg', 
 73  } 
 74   
 75  extension = { 
 76      'eps': 'eps', 
 77      'pdf': 'pdf', 
 78      'png': 'png', 
 79      'svg': 'svg', 
 80      'png_print': 'png', 
 81      'logodata' : 'txt',         
 82      'jpeg'  : 'jpeg' 
 83  } 
 84   
 85   
 86  alphabets = { 
 87      'alphabet_auto': None,  
 88      'alphabet_protein': weblogolib.unambiguous_protein_alphabet,  
 89      'alphabet_rna': weblogolib.unambiguous_rna_alphabet, 
 90      'alphabet_dna': weblogolib.unambiguous_dna_alphabet} 
 91   
 92  color_schemes = {} 
 93  for k in weblogolib.std_color_schemes.keys(): 
 94      color_schemes[ 'color_'+k.replace(' ', '_')] = weblogolib.std_color_schemes[k] 
 95       
 96   
 97  composition = {'comp_none' : 'none', 
 98      'comp_auto' : 'auto', 
 99      'comp_equiprobable':'equiprobable', 
100      'comp_CG': 'percentCG', 
101      'comp_Celegans' : 'C. elegans', 
102      'comp_Dmelanogaster' : 'D. melanogaster', 
103      'comp_Ecoli' : 'E. coli', 
104      'comp_Hsapiens': 'H. sapiens', 
105      'comp_Mmusculus' : 'M. musculus', 
106      'comp_Scerevisiae': 'S. cerevisiae' 
107  } 
108   


109 -class Field(object) :


110      """ A representation of an HTML form field.""" 


111 -    def __init__(self, name, default=None, conversion= None, options=None, errmsg="Illegal value.") :


112          self.name = name 
113          self.default = default 
114          self.value = default 
115          self.conversion = conversion         
116          self.options = options 
117          self.errmsg = errmsg

118   


119 -    def get_value(self) :


120          if self.options : 
121              if not self.value in self.options : 
122                  raise ValueError, (self.name, self.errmsg) 
123                   
124          if self.conversion : 
125              try : 
126                  return self.conversion(self.value) 
127              except ValueError, e : 
128                  raise ValueError, (self.name, self.errmsg) 
129          else: 
130              return self.value

131       
132       


133 -def string_or_none(value) :


134      if value is None or value == 'auto': 
135          return None 
136      return str(value)

137       


138 -def truth(value) :


139      if value== "true" : return True 
140      return bool(value)

141   


142 -def int_or_none(value) :


143      if value =='' or value is None or value == 'auto': 
144          return None 
145      return int(value)

146       


147 -def float_or_none(value) :


148      if value =='' or value is None or value == 'auto': 
149          return None 
150      return float(value)

151           
152   


153 -def main(htdocs_directory = None) :


154    
155      logooptions = weblogolib.LogoOptions()  
156         
157      # A list of form fields. 
158      # The default for checkbox values must be False (irrespective of 
159      # the default in logooptions) since a checked checkbox returns 'true' 
160      # but an unchecked checkbox returns nothing. 
161      controls = [ 
162          Field( 'sequences', ''), 
163          Field( 'format', 'png', weblogolib.formatters.get , 
164              options=['png_print', 'png', 'jpeg', 'eps', 'pdf', 'svg', 'logodata'] , #TODO: Should copy list from __init__.formatters 
165              errmsg="Unknown format option."), 
166          Field( 'stacks_per_line', logooptions.stacks_per_line , int,  
167              errmsg='Invalid number of stacks per line.'), 
168          Field( 'stack_width','medium', weblogolib.std_sizes.get, 
169              options=['small', 'medium', 'large'], errmsg='Invalid logo size.'), 
170          Field( 'alphabet','alphabet_auto', alphabets.get, 
171              options=['alphabet_auto', 'alphabet_protein', 'alphabet_dna',  
172                          'alphabet_rna'], 
173              errmsg="Unknown sequence type."), 
174          Field( 'unit_name', 'bits',  
175              options=[ 'probability', 'bits', 'nats', 'kT', 'kJ/mol',  
176                          'kcal/mol']), 
177          Field( 'first_index', 1, int_or_none), 
178          Field( 'logo_start', '', int_or_none), 
179          Field( 'logo_end', '', int_or_none), 
180          Field( 'composition', 'comp_auto', composition.get, 
181              options=['comp_none','comp_auto','comp_equiprobable','comp_CG', 
182              'comp_Celegans','comp_Dmelanogaster','comp_Ecoli', 
183              'comp_Hsapiens','comp_Mmusculus','comp_Scerevisiae'],  
184              errmsg= "Illegal sequence composition."), 
185          Field( 'percentCG', '', float_or_none, errmsg="Invalid CG percentage."), 
186          Field( 'show_errorbars', False , truth), 
187          Field( 'logo_title', logooptions.logo_title ), 
188          Field( 'logo_label', logooptions.logo_label ), 
189          Field( 'show_xaxis', False, truth), 
190          Field( 'xaxis_label', logooptions.xaxis_label ), 
191          Field( 'show_yaxis', False, truth),   
192          Field( 'yaxis_label', logooptions.yaxis_label, string_or_none ), 
193          Field( 'yaxis_scale', logooptions.yaxis_scale , float_or_none, 
194              errmsg="The yaxis scale must be a positive number." ), 
195          Field( 'yaxis_tic_interval', logooptions.yaxis_tic_interval ,  
196                  float_or_none), 
197          Field( 'show_ends', False, truth),  
198          Field( 'show_fineprint', False , truth),  
199          Field( 'color_scheme', 'color_auto', color_schemes.get, 
200              options=color_schemes.keys() , 
201              errmsg = 'Unknown color scheme'), 
202          Field( 'color0', ''), 
203          Field( 'symbols0', ''), 
204          Field( 'desc0', ''), 
205          Field( 'color1', ''), 
206          Field( 'symbols1', ''), 
207          Field( 'desc1', ''), 
208          Field( 'color2', ''), 
209          Field( 'symbols2', ''), 
210          Field( 'desc2', ''), 
211          Field( 'color3', ''), 
212          Field( 'symbols3', ''), 
213          Field( 'desc3', ''), 
214          Field( 'color4', ''), 
215          Field( 'symbols4', ''), 
216          Field( 'desc4', ''), 
217          Field( 'ignore_lower_case', False, truth),  
218          Field( 'scale_width', False, truth),  
219          ] 
220       
221      form = {} 
222      for c in controls : 
223          form[c.name] = c 
224   
225   
226      form_values = cgilib.FieldStorage() 
227       
228      # Send default form? 
229      if len(form_values) ==0 or form_values.has_key("cmd_reset"): 
230          # Load default truth values now. 
231          form['show_errorbars'].value = logooptions.show_errorbars 
232          form['show_xaxis'].value = logooptions.show_xaxis 
233          form['show_yaxis'].value = logooptions.show_yaxis 
234          form['show_ends'].value = logooptions.show_ends 
235          form['show_fineprint'].value = logooptions.show_fineprint 
236          form['scale_width'].value = logooptions.scale_width 
237           
238          send_form(controls, htdocs_directory = htdocs_directory)  
239          return 
240       
241      # Get form content 
242      for c in controls : 
243          c.value = form_values.getfirst( c.name, c.default)  
244          
245          
246      options_from_form = ['format', 'stacks_per_line', 'stack_width',  
247          'alphabet', 'unit_name', 'first_index', 'logo_start','logo_end', 
248           'composition',  
249          'show_errorbars', 'logo_title', 'logo_label', 'show_xaxis',  
250          'xaxis_label', 
251          'show_yaxis', 'yaxis_label', 'yaxis_scale', 'yaxis_tic_interval', 
252          'show_ends', 'show_fineprint', 'scale_width'] 
253       
254       
255      errors = [] 
256      for optname in options_from_form : 
257          try : 
258              value =  form[optname].get_value() 
259              if value!=None : setattr(logooptions, optname, value) 
260          except ValueError, err : 
261              errors.append(err.args)             
262   
263       
264      # Construct custom color scheme 
265      custom = ColorScheme() 
266      for i in range(0,5) : 
267          color = form["color%d"%i].get_value() 
268          symbols = form["symbols%d"%i].get_value() 
269          desc = form["desc%d"%i].get_value()  
270   
271          if color : 
272              try : 
273                  custom.groups.append(weblogolib.ColorGroup(symbols, color, desc)) 
274              except ValueError, e :  
275                  errors.append( ('color%d'%i, "Invalid color: %s" % color) ) 
276       
277      if form["color_scheme"].value == 'color_custom' : 
278          logooptions.color_scheme =  custom 
279      else : 
280          try : 
281              logooptions.color_scheme = form["color_scheme"].get_value() 
282          except ValueError, err : 
283              errors.append(err.args)             
284   
285      sequences = None 
286   
287      # FIXME: Ugly fix: Must check that sequence_file key exists 
288      # FIXME: Sending malformed or missing form keys should not cause a crash 
289      # sequences_file = form["sequences_file"] 
290      if form_values.has_key("sequences_file") : 
291          sequences = form_values.getvalue("sequences_file")  
292          assert type(sequences) == str 
293   
294      if not sequences or len(sequences)  ==0: 
295          sequences = form["sequences"].get_value() 
296       
297      if not sequences or len(sequences)  ==0: 
298          errors.append( ("sequences", "Please enter a multiple-sequence alignment in the box above, or select a file to upload.")) 
299     
300   
301   
302      # If we have uncovered errors or we want the chance to edit the logo  
303      # ("cmd_edit" command from examples page) then we return the form now. 
304      # We do not proceed to the time consuming logo creation step unless 
305      # required by a 'create' or 'validate' command, and no errors have been 
306      # found yet. 
307      if form_values.has_key("cmd_edit") or errors : 
308          send_form(controls, errors, htdocs_directory) 
309          return     
310    
311      # We write the logo into a local buffer so that we can catch and 
312      # handle any errors. Once the "Content-Type:" header has been sent 
313      # we can't send any useful feedback 
314      logo = StringIO() 
315      try : 
316          comp = form["composition"].get_value() 
317          percentCG = form["percentCG"].get_value() 
318          ignore_lower_case = form_values.has_key("ignore_lower_case")  
319          if comp=='percentCG': comp = str(percentCG/100)      
320           
321          from corebio.matrix import Motif 
322            
323          try: 
324              # Try reading data in transfac format first.  
325              # TODO Refactor this code  
326              motif = Motif.read_transfac(StringIO( sequences), alphabet=logooptions.alphabet) 
327              prior = weblogolib.parse_prior( comp,motif.alphabet)   
328              data = weblogolib.LogoData.from_counts(motif.alphabet, motif, prior)           
329          except ValueError, motif_err : 
330              seqs = weblogolib.read_seq_data(StringIO( sequences),  
331                                          alphabet=logooptions.alphabet, 
332                                          ignore_lower_case=ignore_lower_case 
333                                          ) 
334              prior = weblogolib.parse_prior(comp, seqs.alphabet)                         
335              data = weblogolib.LogoData.from_seqs(seqs, prior)  
336               
337          logoformat =  weblogolib.LogoFormat(data, logooptions) 
338          format = form["format"].value 
339          weblogolib.formatters[format](data, logoformat, logo)             
340      except ValueError, err : 
341          errors.append( err.args ) 
342      except IOError, err : 
343          errors.append( err.args) 
344      except RuntimeError, err : 
345          errors.append( err.args ) 
346                 
347      if form_values.has_key("cmd_validate") or errors : 
348          send_form(controls, errors, htdocs_directory)  
349          return     
350    
351    
352      # 
353      #  RETURN LOGO OVER HTTP 
354      # 
355   
356      print "Content-Type:", mime_type[format] 
357      # Content-Disposition: inline       Open logo in browser window 
358      # Content-Disposition: attachment   Download logo   
359      if form_values.has_key("download") : 
360          print 'Content-Disposition: attachment; ' \ 
361              'filename="logo.%s"' % extension[format]  
362      else :        
363          print 'Content-Disposition: inline; ' \ 
364              'filename="logo.%s"' % extension[format]         
365       
366           
367      # Separate header from data 
368      print  
369           
370      # Finally, and at last, send the logo. 
371      print logo.getvalue()

372   
373     


374 -def send_form(controls, errors=[], htdocs_directory=None) :


375      if htdocs_directory is None : 
376          htdocs_directory = os.path.join( 
377              os.path.dirname(__file__, "htdocs") ) 
378   
379      substitutions = {} 
380      substitutions["version"] = weblogolib.release_description  
381      # Bug fix. Not sure why this default substitution isn't added automatically like everything else 
382      substitutions['color_custom'] = ''   
383      for c in controls : 
384          if c.options : 
385              for opt in c.options : 
386                  substitutions[opt.replace('/','_')] = '' 
387              substitutions[c.value.replace('/','_')] = 'selected' 
388          else : 
389              value = c.value 
390              if value == None : value = 'auto' 
391              if value=='true': 
392                  substitutions[c.name] = 'checked' 
393              elif type(value)==bool : 
394                  if value : 
395                      substitutions[c.name] = 'checked' 
396                  else : 
397                      substitutions[c.name] = '' 
398              else : 
399                  substitutions[c.name] = str(value)             
400          substitutions[c.name+'_err']  = '' 
401      substitutions['logo_range_err'] = '' 
402      
403      # Disable graphics options if necessary auxiliary programs are not installed. 
404      try: 
405          command = find_command('gs') 
406      except EnvironmentError: 
407          try: 
408              command = find_command('gswin32c.exe') 
409          except EnvironmentError: 
410              substitutions['png_print'] = 'disabled="disabled"' 
411              substitutions['png'] = 'disabled="disabled"' 
412              substitutions['jpeg'] = 'disabled="disabled"' 
413              substitutions['pdf'] = 'disabled="disabled"' 
414              substitutions['svg'] = 'disabled="disabled"' 
415              substitutions['eps'] = 'selected="selected"' 
416      try: 
417          command = find_command('pdf2svg') 
418      except EnvironmentError: 
419          substitutions['svg'] = 'disabled="disabled"' 
420          
421       
422      if errors : 
423          print >>sys.stderr, errors 
424          error_message = [] 
425          for e in errors : 
426              if type(e) is str : 
427                  msg = e 
428              elif len(e)==2: 
429                  substitutions[e[0]+"_err"] = "class='error'"     
430                  msg = e[1] 
431              else : 
432                  msg = e[0] 
433   
434   
435              error_message +=  "ERROR: "  
436              error_message +=  msg 
437              error_message += ' <br />' 
438               
439          error_message += \ 
440              "<input style='float:right; font-size:small' type='submit' name='cmd_validate' value='Clear Error' /> " 
441          substitutions["error_message"] = ''.join(error_message) 
442      else : 
443          substitutions["error_message"] = "" 
444       
445           
446      template = resource_string( "create_html_template.html", htdocs_directory) 
447      html = Template(template).safe_substitute(substitutions) #FIXME 
448   
449      print "Content-Type: text/html\n\n" 
450      print html

451   
452      # DEBUG 
453      #keys = substitutions.keys() 
454      #keys.sort() 
455      #for k in keys : 
456      #    print k,"=", substitutions[k], " <br />"  
457   
458      #print " <br />" 
459      #print " <br />" 
460      #for k in controls : 
461      #    print k.name,"=", k.get_value(), " <br />"  
462   
463   
464   
465  if __name__=="__main__" : 
466      import _cli 
467      _cli.main() 
468
```

  


| Home | Trees | Indices | Help | WebLogo |
| --- | --- | --- | --- | --- |

|  |  |
| --- | --- |
| Generated by Epydoc 3.0.1 on Mon Jan 30 17:27:18 2012 | http://epydoc.sourceforge.net |
