# Supplementary material for: DIVERSITY in binding, regulation, and evolution revealed from high-throughput ChIP
Source: PLoS Comput Biol. 2018 Apr 23;14(4):e1006090. doi: 10.1371/journal.pcbi.1006090 (PMC5933800; doi:10.1371/journal.pcbi.1006090)
Supplement: S1 File — (GZ) [file pcbi.1006090.s009.tar.gz › DIVERSITY-master/weblogoMod/apidocs/weblogolib._cli-pysrc.html]

xml version="1.0" encoding="ascii"?


weblogolib.\_cli


| Home | Trees | Indices | Help | WebLogo |
| --- | --- | --- | --- | --- |

Package weblogolib ::
Module \_cli

# Source Code for Module weblogolib.\_cli

```
  1  #!/usr/bin/env python 
  2   
  3  # -------------------------------- WebLogo -------------------------------- 
  4   
  5  #  Copyright (c) 2003-2004 The Regents of the University of California. 
  6  #  Copyright (c) 2005 Gavin E. Crooks 
  7  #  Copyright (c) 2006-2011, The Regents of the University of California, through  
  8  #  Lawrence Berkeley National Laboratory (subject to receipt of any required 
  9  #  approvals from the U.S. Dept. of Energy).  All rights reserved. 
 10   
 11  #  This software is distributed under the new BSD Open Source License. 
 12  #  <http://www.opensource.org/licenses/bsd-license.html> 
 13  # 
 14  #  Redistribution and use in source and binary forms, with or without  
 15  #  modification, are permitted provided that the following conditions are met:  
 16  # 
 17  #  (1) Redistributions of source code must retain the above copyright notice,  
 18  #  this list of conditions and the following disclaimer.  
 19  # 
 20  #  (2) Redistributions in binary form must reproduce the above copyright  
 21  #  notice, this list of conditions and the following disclaimer in the  
 22  #  documentation and or other materials provided with the distribution.  
 23  # 
 24  #  (3) Neither the name of the University of California, Lawrence Berkeley  
 25  #  National Laboratory, U.S. Dept. of Energy nor the names of its contributors  
 26  #  may be used to endorse or promote products derived from this software  
 27  #  without specific prior written permission.  
 28  # 
 29  #  THIS SOFTWARE IS PROVIDED BY THE COPYRIGHT HOLDERS AND CONTRIBUTORS "AS IS"  
 30  #  AND ANY EXPRESS OR IMPLIED WARRANTIES, INCLUDING, BUT NOT LIMITED TO, THE  
 31  #  IMPLIED WARRANTIES OF MERCHANTABILITY AND FITNESS FOR A PARTICULAR PURPOSE  
 32  #  ARE DISCLAIMED. IN NO EVENT SHALL THE COPYRIGHT OWNER OR CONTRIBUTORS BE  
 33  #  LIABLE FOR ANY DIRECT, INDIRECT, INCIDENTAL, SPECIAL, EXEMPLARY, OR  
 34  #  CONSEQUENTIAL DAMAGES (INCLUDING, BUT NOT LIMITED TO, PROCUREMENT OF  
 35  #  SUBSTITUTE GOODS OR SERVICES; LOSS OF USE, DATA, OR PROFITS; OR BUSINESS  
 36  #  INTERRUPTION) HOWEVER CAUSED AND ON ANY THEORY OF LIABILITY, WHETHER IN  
 37  #  CONTRACT, STRICT LIABILITY, OR TORT (INCLUDING NEGLIGENCE OR OTHERWISE)  
 38  #  ARISING IN ANY WAY OUT OF THE USE OF THIS SOFTWARE, EVEN IF ADVISED OF THE  
 39  #  POSSIBILITY OF SUCH DAMAGE.  
 40   
 41  # WebLogo Command Line Interface 
 42   
 43  import sys 
 44   
 45  from color import * 
 46  from colorscheme import ColorScheme, ColorGroup 
 47  from corebio.utils import * 
 48  from string import Template 
 49   
 50  from corebio import seq_io 
 51   
 52   
 53   
 54  import os  
 55  from corebio.utils.deoptparse import DeOptionParser 
 56  from optparse import OptionGroup 
 57   
 58  from weblogolib import LogoOptions, LogoData, LogoFormat 
 59  from weblogolib import parse_prior, description, release_description, formatters, default_formatter 
 60  from weblogolib import std_alphabets, std_units, std_sizes, std_color_schemes 
 61  from weblogolib import read_seq_data 
 62   
 63  # ====================== Main: Parse Command line ============================= 


64 -def main():

 65      """WebLogo command line interface """ 
 66       
 67      # ------ Parse Command line ------ 
 68      parser = _build_option_parser()                 
 69      (opts, args) = parser.parse_args(sys.argv[1:]) 
 70      if args : parser.error("Unparsable arguments: %s " % args) 
 71       
 72      if opts.serve: 
 73          httpd_serve_forever(opts.port) # Never returns? 
 74          sys.exit(0)  
 75     
 76               
 77      # ------ Create Logo ------ 
 78      try: 
 79          data = _build_logodata(opts) 
 80          format = _build_logoformat(data, opts) 
 81           
 82          formatter = opts.formatter 
 83          formatter(data, format, opts.fout) 
 84   
 85      except ValueError, err : 
 86          print >>sys.stderr, 'Error:', err 
 87          sys.exit(2) 
 88      except KeyboardInterrupt, err: 
 89          sys.exit(0) 
 90  # End main()             
 91           
 92   


93 -def httpd_serve_forever(port=8080) :


94      """ Start a webserver on a local port.""" 
 95      import BaseHTTPServer 
 96      import CGIHTTPServer  
 97       
 98      class __HTTPRequestHandler(CGIHTTPServer.CGIHTTPRequestHandler): 
 99          def is_cgi(self) : 
100              if self.path == "/create.cgi":  
101                  self.cgi_info = '', 'create.cgi' 
102                  return True 
103              return False

104       
105      # Add current directory to PYTHONPATH. This is 
106      # so that we can run the standalone server 
107      # without having to run the install script.       
108      pythonpath = os.getenv("PYTHONPATH", '') 
109      pythonpath += ":" + os.path.abspath(sys.path[0]).split()[0] 
110      os.environ["PYTHONPATH"] = pythonpath 
111   
112      htdocs = resource_filename(__name__, 'htdocs', __file__) 
113      os.chdir(htdocs)  
114   
115      HandlerClass = __HTTPRequestHandler 
116      ServerClass = BaseHTTPServer.HTTPServer 
117      httpd = ServerClass(('', port), HandlerClass) 
118      print "WebLogo server running at http://localhost:%d/" % port 
119       
120      try : 
121          httpd.serve_forever() 
122      except KeyboardInterrupt: 
123          sys.exit(0) 
124  # end httpd_serve_forever()   
125       
126       
127   


128 -def _build_logodata(options) :


129       
130      if options.input_parser != "transfac": 
131          seqs = read_seq_data(options.fin,  
132              options.input_parser.read, 
133              alphabet=options.alphabet, 
134              ignore_lower_case = options.ignore_lower_case)    
135               
136          if options.reverse:  
137              seqs = SeqList([s.reverse() for s in seqs], seqs.alphabet) 
138           
139          if options.complement : 
140              seqs= SeqList( [Seq(s,seqs.alphabet).complement() for s in seqs], seqs.alphabet) 
141   
142             
143           
144          prior = parse_prior( options.composition,seqs.alphabet, options.weight) 
145          data = LogoData.from_seqs(seqs, prior) 
146   
147      else : 
148          from corebio.matrix import Motif 
149       
150          if options.ignore_lower_case: 
151              raise ValueError("error: option --ignore-lower-case incompatible with matrix input") 
152           
153          #FIXME : implement 
154          if options.reverse: 
155              raise ValueError("error: option --reverse incompatible with matrix input") 
156           
157          #FIXME : implement 
158          if options.complement: 
159              raise ValueError("error: option --complement incompatible with matrix input") 
160                         
161          motif = Motif.read_transfac(options.fin, alphabet=options.alphabet) 
162          prior = parse_prior( options.composition,motif.alphabet, options.weight) 
163          data = LogoData.from_counts(motif.alphabet, motif, prior) 
164           
165      return data

166        
167                


168 -def _build_logoformat( logodata, opts) :


169      """ Extract and process relevant option values and return a  
170      LogoFormat object."""  
171   
172      args = {}   
173      direct_from_opts = [ 
174          "stacks_per_line",  
175          "logo_title", 
176          "yaxis_label",  
177          "show_xaxis", 
178          "show_yaxis",         
179          "xaxis_label",  
180          "show_ends", 
181          "fineprint",   
182          "show_errorbars",  
183          "show_boxes",   
184          "yaxis_tic_interval",  
185          "resolution",       
186          "alphabet", 
187          "debug", 
188          "show_ends", 
189          "default_color", 
190          #"show_color_key", 
191          "color_scheme", 
192          "unit_name", 
193          "logo_label", 
194          "yaxis_scale", 
195          "first_index", 
196          "logo_start", 
197          "logo_end", 
198          "scale_width",  
199          "annotate", 
200          "stack_width", 
201          "stack_aspect_ratio" 
202          ] 
203     
204      for k in direct_from_opts: 
205          args[k] = opts.__dict__[k] 
206   
207  #    logo_size = copy.copy(opts.__dict__['logo_size']) 
208  #    size_from_opts = ["stack_width", "stack_height"] 
209  #    for k in size_from_opts : 
210  #        length = getattr(opts, k) 
211  #        if length : setattr( logo_size, k, length ) 
212  #   args["size"] = logo_size     
213   
214   
215      if opts.colors: 
216          color_scheme = ColorScheme() 
217          for color, symbols, desc in opts.colors: 
218              try : 
219                  #c = Color.from_string(color) 
220                  color_scheme.groups.append( ColorGroup(symbols, color, desc)  ) 
221              except ValueError :  
222                  raise ValueError( 
223                       "error: option --color: invalid value: '%s'" % color )           
224                    
225          args["color_scheme"] = color_scheme 
226    
227    
228      if opts.annotate: 
229          args["annotate"] = opts.annotate.split(',') 
230    
231       
232      logooptions = LogoOptions()  
233      for a, v in args.iteritems() : 
234          setattr(logooptions,a,v) 
235   
236       
237      theformat =  LogoFormat(logodata, logooptions ) 
238      return theformat

239   
240       
241      
242       
243      
244       
245  # ========================== OPTIONS ========================== 


246 -def _build_option_parser() :


247      defaults = LogoOptions() 
248      parser = DeOptionParser(usage="%prog [options]  < sequence_data.fa > sequence_logo.eps", 
249          description = description, 
250          version     = release_description, 
251          add_verbose_options = False 
252          )     
253           
254      io_grp = OptionGroup(parser, "Input/Output Options",) 
255      data_grp = OptionGroup(parser, "Logo Data Options",) 
256      trans_grp = OptionGroup(parser, "Transformations", "Optional transformations of the sequence data.") 
257   
258    
259      format_grp = OptionGroup(parser, "Logo Format Options",  
260          "These options control the format and display of the logo.") 
261      color_grp = OptionGroup(parser, "Color Options",  
262          "Colors can be specified using CSS2 syntax. e.g. 'red', '#FF0000', etc.") 
263      advanced_grp = OptionGroup(parser, "Advanced Format Options",  
264          "These options provide fine control over the display of the logo. ") 
265      server_grp = OptionGroup(parser, "WebLogo Server", 
266          "Run a standalone webserver on a local port.") 
267   
268   
269      parser.add_option_group(io_grp) 
270      parser.add_option_group(data_grp) 
271      parser.add_option_group(trans_grp)       
272      parser.add_option_group(format_grp)   
273      parser.add_option_group(color_grp) 
274      parser.add_option_group(advanced_grp) 
275      parser.add_option_group(server_grp) 
276       
277      # ========================== IO OPTIONS ========================== 
278       
279      io_grp.add_option( "-f", "--fin", 
280          dest="fin", 
281          action="store", 
282          type="file_in", 
283          default=sys.stdin, 
284          help="Sequence input file (default: stdin)", 
285          metavar="FILENAME") 
286   
287      # Add position weight matrix formats to input parsers by hand 
288      fin_choices = dict(seq_io.format_names()) 
289      fin_choices['transfac'] = 'transfac' 
290       
291      io_grp.add_option("-D", "--datatype",  
292          dest="input_parser", 
293          action="store", type ="dict", 
294          default = seq_io, 
295          choices = fin_choices,       # seq_io.format_names(), 
296          help="Type of multiple sequence alignment or position weight matrix file: (%s, transfac)" %  
297             ', '.join([ f.names[0] for f in seq_io.formats]), 
298          metavar="FORMAT") 
299   
300      io_grp.add_option("-o", "--fout", dest="fout", 
301          type="file_out", 
302          default=sys.stdout, 
303          help="Output file (default: stdout)", 
304          metavar="FILENAME") 
305   
306      io_grp.add_option( "-F", "--format", 
307          dest="formatter", 
308          action="store", 
309          type="dict", 
310          choices = formatters, 
311          metavar= "FORMAT", 
312          help="Format of output: eps (default), png, png_print, pdf, jpeg, svg, logodata", 
313          default = default_formatter) 
314   
315   
316      # ========================== Data OPTIONS ========================== 
317   
318   
319    
320      data_grp.add_option( "-A", "--sequence-type", 
321          dest="alphabet", 
322          action="store", 
323          type="dict", 
324          choices = std_alphabets, 
325          help="The type of sequence data: 'protein', 'rna' or 'dna'.", 
326          metavar="TYPE") 
327                          
328      data_grp.add_option( "-a", "--alphabet", 
329          dest="alphabet", 
330          action="store", 
331          help="The set of symbols to count, e.g. 'AGTC'. " 
332               "All characters not in the alphabet are ignored. " 
333               "If neither the alphabet nor sequence-type are specified then weblogo will examine the input data and make an educated guess. " 
334               "See also --sequence-type, --ignore-lower-case" )                        
335       
336          
337      data_grp.add_option( "-U", "--units", 
338          dest="unit_name", 
339          action="store", 
340          choices = std_units.keys(),     
341          type="choice", 
342          default = defaults.unit_name, 
343          help="A unit of entropy ('bits' (default), 'nats', 'digits'), or a unit of free energy ('kT', 'kJ/mol', 'kcal/mol'), or 'probability' for probabilities", 
344          metavar = "NUMBER")  
345   
346   
347      data_grp.add_option( "", "--composition", 
348          dest="composition", 
349          action="store", 
350          type="string", 
351          default = "auto", 
352          help="The expected composition of the sequences: 'auto' (default), 'equiprobable', 'none' (do not perform any compositional adjustment), a CG percentage, a species name (e.g. 'E. coli', 'H. sapiens'), or an explicit distribution (e.g. \"{'A':10, 'C':40, 'G':40, 'T':10}\"). The automatic option uses a typical distribution for proteins and equiprobable distribution for everything else. ", 
353          metavar="COMP.") 
354   
355      data_grp.add_option( "", "--weight", 
356          dest="weight", 
357          action="store", 
358          type="float", 
359          default = None, 
360          help="The weight of prior data.  Default: total pseudocounts equal to the number of monomer types.", 
361          metavar="NUMBER") 
362   
363      data_grp.add_option( "-i", "--first-index", 
364          dest="first_index", 
365          action="store", 
366          type="int", 
367          default = 1, 
368          help="Index of first position in sequence data (default: 1)", 
369          metavar="INDEX") 
370   
371      data_grp.add_option( "-l", "--lower", 
372          dest="logo_start", 
373          action="store", 
374          type="int", 
375          help="Lower bound of sequence to display", 
376          metavar="INDEX") 
377       
378      data_grp.add_option( "-u", "--upper", 
379          dest="logo_end", 
380          action="store", 
381          type="int", 
382          help="Upper bound of sequence to display", 
383          metavar="INDEX") 
384   
385      # ========================== Transformation OPTIONS ========================== 
386       
387   
388      # FIXME Add test?  
389      trans_grp.add_option( "", "--ignore-lower-case", 
390          dest="ignore_lower_case", 
391          action="store_true", 
392          default=False, 
393          help="Disregard lower case letters and only count upper case letters in sequences." 
394         ) 
395   
396      trans_grp.add_option( "", "--reverse", 
397          dest="reverse", 
398          action="store_true", 
399          default=False, 
400          help="reverse sequences", 
401          ) 
402   
403      trans_grp.add_option( "", "--complement", 
404          dest="complement", 
405          action="store_true", 
406          default=False, 
407          help="complement DNA sequences", 
408          ) 
409       
410   
411   
412      # ========================== FORMAT OPTIONS ========================== 
413   
414      format_grp.add_option( "-s", "--size", 
415          dest="stack_width", 
416          action="store", 
417          type ="dict", 
418          choices = std_sizes, 
419          metavar = "LOGOSIZE", 
420          default = defaults.stack_width, 
421          help="Specify a standard logo size (small, medium (default), large)" ) 
422               
423      format_grp.add_option( "-n", "--stacks-per-line", 
424          dest="stacks_per_line", 
425          action="store", 
426          type="int", 
427          help="Maximum number of logo stacks per logo line. (default: %default)", 
428          default = defaults.stacks_per_line, 
429          metavar="COUNT") 
430   
431      format_grp.add_option( "-t", "--title", 
432          dest="logo_title", 
433          action="store", 
434          type="string", 
435          help="Logo title text.", 
436          default = defaults.logo_title, 
437          metavar="TEXT") 
438   
439      format_grp.add_option( "", "--label", 
440          dest="logo_label", 
441          action="store", 
442          type="string", 
443          help="A figure label, e.g. '2a'", 
444          default = defaults.logo_label, 
445          metavar="TEXT") 
446   
447      format_grp.add_option( "-X", "--show-xaxis", 
448          action="store", 
449          type = "boolean", 
450          default= defaults.show_xaxis, 
451          metavar = "YES/NO", 
452          help="Display sequence numbers along x-axis? (default: %default)") 
453                          
454      format_grp.add_option( "-x", "--xlabel", 
455          dest="xaxis_label", 
456          action="store", 
457          type="string", 
458          default = defaults.xaxis_label, 
459          help="X-axis label", 
460          metavar="TEXT") 
461   
462      format_grp.add_option( "", "--annotate", 
463              dest="annotate", 
464              action="store", 
465              type="string", 
466              default = None, 
467              help="A comma separated list of custom stack annotations, e.g. '1,3,4,5,6,7'.  Annotation list must be same length as sequences.", 
468              metavar="TEXT") 
469   
470      format_grp.add_option( "-S", "--yaxis", 
471          dest="yaxis_scale", 
472          action="store", 
473          type="float", 
474          help="Height of yaxis in units. (Default: Maximum value with uninformative prior.)", 
475          metavar = "UNIT")  
476   
477      format_grp.add_option( "-Y", "--show-yaxis", 
478          action="store", 
479          type = "boolean", 
480          dest = "show_yaxis", 
481          default= defaults.show_yaxis, 
482          metavar = "YES/NO", 
483          help="Display entropy scale along y-axis? (default: %default)") 
484   
485      format_grp.add_option( "-y", "--ylabel", 
486          dest="yaxis_label", 
487          action="store", 
488          type="string", 
489          help="Y-axis label (default depends on plot type and units)", 
490          metavar="TEXT") 
491   
492      format_grp.add_option( "-E", "--show-ends", 
493          action="store", 
494          type = "boolean", 
495          default= defaults.show_ends, 
496          metavar = "YES/NO", 
497          help="Label the ends of the sequence? (default: %default)") 
498           
499      format_grp.add_option( "-P", "--fineprint", 
500          dest="fineprint", 
501          action="store", 
502          type="string", 
503          default= defaults.fineprint, 
504          help="The fine print (default: weblogo version)", 
505          metavar="TEXT") 
506   
507      format_grp.add_option( "", "--ticmarks", 
508          dest="yaxis_tic_interval", 
509          action="store", 
510          type="float", 
511          default= defaults.yaxis_tic_interval, 
512          help="Distance between ticmarks (default: %default)", 
513          metavar = "NUMBER") 
514   
515           
516      format_grp.add_option( "", "--errorbars", 
517          dest = "show_errorbars", 
518          action="store", 
519          type = "boolean", 
520          default= defaults.show_errorbars, 
521          metavar = "YES/NO", 
522          help="Display error bars? (default: %default)") 
523        
524          
525           
526      # ========================== Color OPTIONS ========================== 
527      # TODO: Future Feature 
528      # color_grp.add_option( "-K", "--color-key", 
529      #    dest= "show_color_key", 
530      #    action="store", 
531      #    type = "boolean", 
532      #    default= defaults.show_color_key, 
533      #    metavar = "YES/NO", 
534      #    help="Display a color key (default: %default)") 
535           
536           
537      color_scheme_choices = std_color_schemes.keys()     
538      color_scheme_choices.sort()     
539      color_grp.add_option( "-c", "--color-scheme", 
540          dest="color_scheme", 
541          action="store", 
542          type ="dict", 
543          choices = std_color_schemes, 
544          metavar = "SCHEME", 
545          default = None, # Auto 
546          help="Specify a standard color scheme (%s)" % \ 
547              ", ".join(color_scheme_choices) ) 
548               
549      color_grp.add_option( "-C", "--color", 
550          dest="colors", 
551          action="append", 
552          metavar="COLOR SYMBOLS DESCRIPTION ", 
553          nargs = 3, 
554          default=[], 
555          help="Specify symbol colors, e.g. --color black AG 'Purine' --color red TC 'Pyrimidine' ")     
556   
557      color_grp.add_option( "", "--default-color", 
558          dest="default_color", 
559          action="store", 
560          metavar="COLOR", 
561          default= defaults.default_color, 
562          help="Symbol color if not otherwise specified.") 
563   
564   
565      # ========================== Advanced options =========================    
566                   
567      advanced_grp.add_option( "-W", "--stack-width", 
568          dest="stack_width", 
569          action="store", 
570          type="float", 
571          default= defaults.stack_width, 
572          help="Width of a logo stack (default: %s)"% defaults.stack_width, 
573          metavar="POINTS" ) 
574   
575      advanced_grp.add_option( "", "--aspect-ratio", 
576          dest="stack_aspect_ratio", 
577          action="store", 
578          type="float", 
579          default= defaults.stack_aspect_ratio , 
580          help="Ratio of stack height to width (default: %s)"%defaults.stack_aspect_ratio, 
581          metavar="POINTS" )     
582   
583      advanced_grp.add_option( "", "--box", 
584          dest="show_boxes", 
585          action="store", 
586          type = "boolean", 
587          default=False,  
588          metavar = "YES/NO", 
589          help="Draw boxes around symbols? (default: no)") 
590   
591      advanced_grp.add_option( "", "--resolution", 
592          dest="resolution", 
593          action="store", 
594          type="float", 
595          default=96, 
596          help="Bitmap resolution in dots per inch (DPI).  (Default: 96 DPI, except png_print, 600 DPI) Low resolution bitmaps (DPI<300) are antialiased.", 
597          metavar="DPI")   
598   
599      advanced_grp.add_option( "", "--scale-width", 
600          dest="scale_width", 
601          action="store", 
602          type = "boolean", 
603          default= True,  
604          metavar = "YES/NO", 
605          help="Scale the visible stack width by the fraction of symbols in the column?  (I.e. columns with many gaps of unknowns are narrow.)  (Default: yes)") 
606      
607      advanced_grp.add_option( "", "--debug", 
608          action="store", 
609          type = "boolean", 
610          default= defaults.debug, 
611          metavar = "YES/NO", 
612          help="Output additional diagnostic information. (Default: %default)") 
613   
614   
615      # ========================== Server options =========================    
616      server_grp.add_option( "", "--serve", 
617          dest="serve", 
618          action="store_true", 
619          default= False, 
620          help="Start a standalone WebLogo server for creating sequence logos.")     
621       
622      server_grp.add_option( "", "--port", 
623          dest="port", 
624          action="store", 
625          type="int", 
626          default= 8080, 
627          help="Listen to this local port. (Default: %default)", 
628          metavar="PORT") 
629   
630      return parser

631       
632      # END _build_option_parser 
633   
634   
635  ############################################################## 
636
```

  


| Home | Trees | Indices | Help | WebLogo |
| --- | --- | --- | --- | --- |

|  |  |
| --- | --- |
| Generated by Epydoc 3.0.1 on Mon Jan 30 17:27:19 2012 | http://epydoc.sourceforge.net |
