# Supplementary material for: DIVERSITY in binding, regulation, and evolution revealed from high-throughput ChIP
Source: PLoS Comput Biol. 2018 Apr 23;14(4):e1006090. doi: 10.1371/journal.pcbi.1006090 (PMC5933800; doi:10.1371/journal.pcbi.1006090)
Supplement: S1 File — (GZ) [file pcbi.1006090.s009.tar.gz › DIVERSITY-master/weblogoMod/apidocs/weblogolib.color-pysrc.html]

xml version="1.0" encoding="ascii"?


weblogolib.color


| Home | Trees | Indices | Help | WebLogo |
| --- | --- | --- | --- | --- |

Package weblogolib ::
Module color

# Source Code for Module weblogolib.color

```
  1   
  2   
  3  #  Copyright (c) 2005 Gavin E. Crooks 
  4  # 
  5  #  This software is distributed under the MIT Open Source License. 
  6  #  <http://www.opensource.org/licenses/mit-license.html> 
  7  # 
  8  #  Permission is hereby granted, free of charge, to any person obtaining a  
  9  #  copy of this software and associated documentation files (the "Software"), 
 10  #  to deal in the Software without restriction, including without limitation 
 11  #  the rights to use, copy, modify, merge, publish, distribute, sublicense, 
 12  #  and/or sell copies of the Software, and to permit persons to whom the 
 13  #  Software is furnished to do so, subject to the following conditions: 
 14  # 
 15  #  The above copyright notice and this permission notice shall be included 
 16  #  in all copies or substantial portions of the Software. 
 17  # 
 18  #  THE SOFTWARE IS PROVIDED "AS IS", WITHOUT WARRANTY OF ANY KIND, EXPRESS OR  
 19  #  IMPLIED, INCLUDING BUT NOT LIMITED TO THE WARRANTIES OF MERCHANTABILITY,  
 20  #  FITNESS FOR A PARTICULAR PURPOSE AND NONINFRINGEMENT. IN NO EVENT SHALL THE 
 21  #  AUTHORS OR COPYRIGHT HOLDERS BE LIABLE FOR ANY CLAIM, DAMAGES OR OTHER  
 22  #  LIABILITY, WHETHER IN AN ACTION OF CONTRACT, TORT OR OTHERWISE, ARISING FROM, 
 23  #  OUT OF OR IN CONNECTION WITH THE SOFTWARE OR THE USE OR OTHER DEALINGS IN  
 24  #  THE SOFTWARE. 
 25   
 26  """ Color specifications using CSS2 (Cascading Style Sheet) syntax.""" 
 27   


28 -class Color:

 29      """ Color specifications using CSS2 (Cascading Style Sheet) syntax. 
 30       
 31      http://www.w3.org/TR/REC-CSS2/syndata.html#color-units 
 32   
 33      Usage: 
 34       
 35      red = Color(255,0,0) 
 36      red = Color(1., 0., 0.) 
 37      red = Color.by_name("red") 
 38      red = Color.from_rgb(1.,0.,0.) 
 39      red = Color.from_rgb(255,0,0) 
 40      red = Color.from_hsl(0.,1., 0.5) 
 41       
 42      red = Color.from_string("red")  
 43      red = Color.from_string("RED")  
 44      red = Color.from_string("#F00")  
 45      red = Color.from_string("#FF0000")  
 46      red = Color.from_string("rgb(255, 0, 0)") 
 47      red = Color.from_string("rgb(100%, 0%, 0%)") 
 48      red = Color.from_string("hsl(0, 100%, 50%)") 
 49   
 50      """ 


51 -    def __init__(self, red, green, blue) :


52           
 53          if ( type(red) is not type(green) ) or (type(red) is not type(blue)): 
 54              raise TypeError("Mixed floats and integers?") 
 55   
 56          if type(red) is type(1) : red = float(red)/255. 
 57          if type(green) is type(1) : green = float(green)/255.                   
 58          if type(blue) is type(1) : blue = float(blue)/255. 
 59   
 60          self.red = max(0., min(float(red), 1.0)) 
 61          self.green = max(0., min(float(green), 1.0)) 
 62          self.blue = max(0., min(float(blue), 1.0))

 63   
 64      #@staticmethod 


65 -    def names():


66          "Return a list of standard color names." 
 67          return _std_colors.keys()

 68      names = staticmethod(names) 
 69   
 70      #@classmethod 


71 -    def from_rgb(cls, r, g, b):


72          return cls(r,g,b)

 73      from_rgb = classmethod(from_rgb) 
 74    
 75      #@classmethod 


76 -    def from_hsl(cls, hue_angle, saturation, lightness ):


77          def hue_to_rgb( v1, v2, vH) : 
 78              if vH < 0.0 : vH += 1.0 
 79              if vH > 1.0 : vH -= 1.0 
 80              if vH*6.0 < 1.0 : return (v1 + (v2 - v1) * 6.0 * vH) 
 81              if vH*2.0 < 1.0 : return v2 
 82              if vH*3.0 < 2.0 : return (v1 + (v2 - v1) * ((2.0/3.0) - vH) * 6.0) 
 83              return v1

 84       
 85          hue =  (((hue_angle % 360.) + 360.) % 360.)/360. 
 86           
 87          if not (saturation >= 0.0 and saturation <=1.0) : 
 88              raise ValueError("Out-of-range saturation %f"% saturation) 
 89          if not (lightness >= 0.0 and lightness <=1.0) : 
 90              raise ValueError("Out-of-range lightness %f"% lightness) 
 91                   
 92          if saturation == 0 : 
 93              # greyscale 
 94              return cls.from_rgb( lightness, lightness, lightness) 
 95       
 96          if lightness < 0.5 : 
 97              v2 = lightness * (1.0+ saturation) 
 98          else : 
 99              v2 = (lightness + saturation) - (saturation* lightness)        
100   
101          v1 = 2.0 * lightness - v2 
102          r = hue_to_rgb( v1, v2, hue + (1./3.) ) 
103          g = hue_to_rgb( v1, v2, hue ) 
104          b = hue_to_rgb( v1, v2, hue - (1./3.) ) 
105   
106          return cls(r,g,b) 
107      from_hsl = classmethod(from_hsl)   
108    
109       
110      #@staticmethod 


111 -    def by_name(string):


112          s = string.strip().lower().replace(' ', '') 
113           
114          try: 
115              return _std_colors[s] 
116          except KeyError: 
117              raise ValueError("Unknown color name: %s"%  s)

118      by_name = staticmethod(by_name) 
119          
120      #@classmethod 


121 -    def from_string(cls, string):


122          def to_frac(string) : 
123              # string can be "255" or "100%" 
124              if string[-1]=='%':  
125                  return float(string[0:-1])/100. 
126              else: 
127                  return float(string)/255.

128                   
129          s = string.strip().lower().replace(' ', '').replace('_', '') 
130           
131          if s in _std_colors : # "red" 
132              return _std_colors[s] 
133   
134          if s[0] == "#" :    # "#fef" 
135              if len(s) == 4 : 
136                  r = int(s[1]+s[1],16) 
137                  g = int(s[2]+s[2],16) 
138                  b = int(s[3]+s[3],16) 
139                  return cls(r,g,b) 
140              elif len(s) ==7 :   # "#ff00aa" 
141                  r = int(s[1:3],16) 
142                  g = int(s[3:5],16) 
143                  b = int(s[5:7],16) 
144                  return cls(r,g,b) 
145              else : 
146                  raise ValueError("Cannot parse string: %s" % s) 
147   
148          if s[0:4] == 'rgb(' and s[-1] == ')' : 
149              rgb = s[4:-1].split(",") 
150              if len(rgb) != 3 : 
151                  raise ValueError("Cannot parse string a: %s" % s) 
152              return cls( to_frac(rgb[0]), to_frac(rgb[1]), to_frac(rgb[2])) 
153   
154          if s[0:4] == 'hsl(' and s[-1] == ')' : 
155              hsl = s[4:-1].split(",") 
156              if len(hsl) != 3 : 
157                  raise ValueError("Cannot parse string a: %s" % s) 
158              return cls.from_hsl( int(hsl[0]), to_frac(hsl[1]), to_frac(hsl[2])) 
159           
160          raise ValueError("Cannot parse string: %s" % s) 
161      from_string = classmethod(from_string) 
162   


163 -    def __eq__(self, other) :


164          req = int(0.5+255.*self.red) == int(0.5+255.*other.red) 
165          beq = int(0.5+255.*self.blue) == int(0.5+255.*other.blue) 
166          geq = int(0.5+255.*self.green) == int(0.5+255.*other.green) 
167           
168          return req and beq and geq

169   


170 -    def __repr__(self):


171          return "Color(%f,%f,%f)" % (self.red, self.green, self.blue)

172           
173       
174  _std_colors = dict( 
175      aliceblue = Color(240,248,255),     #f0f8ff 
176      antiquewhite = Color(250,235,215),     #faebd7 
177      aqua = Color(0,255,255),     #00ffff 
178      aquamarine = Color(127,255,212),     #7fffd4 
179      azure = Color(240,255,255),     #f0ffff 
180      beige = Color(245,245,220),     #f5f5dc 
181      bisque = Color(255,228,196),     #ffe4c4 
182      black = Color(0,0,0),     #000000 
183      blanchedalmond = Color(255,235,205),     #ffebcd 
184      blue = Color(0,0,255),     #0000ff 
185      blueviolet = Color(138,43,226),     #8a2be2 
186      brown = Color(165,42,42),     #a52a2a 
187      burlywood = Color(222,184,135),     #deb887 
188      cadetblue = Color(95,158,160),     #5f9ea0 
189      chartreuse = Color(127,255,0),     #7fff00 
190      chocolate = Color(210,105,30),     #d2691e 
191      coral = Color(255,127,80),     #ff7f50 
192      cornflowerblue = Color(100,149,237),     #6495ed 
193      cornsilk = Color(255,248,220),     #fff8dc 
194      crimson = Color(220,20,60),     #dc143c 
195      cyan = Color(0,255,255),     #00ffff 
196      darkblue = Color(0,0,139),     #00008b 
197      darkcyan = Color(0,139,139),     #008b8b 
198      darkgoldenrod = Color(184,134,11),     #b8860b 
199      darkgray = Color(169,169,169),     #a9a9a9 
200      darkgreen = Color(0,100,0),     #006400 
201      darkgrey = Color(169,169,169),     #a9a9a9 
202      darkkhaki = Color(189,183,107),     #bdb76b 
203      darkmagenta = Color(139,0,139),     #8b008b 
204      darkolivegreen = Color(85,107,47),     #556b2f 
205      darkorange = Color(255,140,0),     #ff8c00 
206      darkorchid = Color(153,50,204),     #9932cc 
207      darkred = Color(139,0,0),     #8b0000 
208      darksalmon = Color(233,150,122),     #e9967a 
209      darkseagreen = Color(143,188,143),     #8fbc8f 
210      darkslateblue = Color(72,61,139),     #483d8b 
211      darkslategray = Color(47,79,79),     #2f4f4f 
212      darkslategrey = Color(47,79,79),     #2f4f4f 
213      darkturquoise = Color(0,206,209),     #00ced1 
214      darkviolet = Color(148,0,211),     #9400d3 
215      deeppink = Color(255,20,147),     #ff1493 
216      deepskyblue = Color(0,191,255),     #00bfff 
217      dimgray = Color(105,105,105),     #696969 
218      dimgrey = Color(105,105,105),     #696969     
219      dodgerblue = Color(30,144,255),     #1e90ff 
220      firebrick = Color(178,34,34),     #b22222 
221      floralwhite = Color(255,250,240),     #fffaf0 
222      forestgreen = Color(34,139,34),     #228b22 
223      fuchsia = Color(255,0,255),     #ff00ff 
224      gainsboro = Color(220,220,220),     #dcdcdc 
225      ghostwhite = Color(248,248,255),     #f8f8ff 
226      gold = Color(255,215,0),     #ffd700 
227      goldenrod = Color(218,165,32),     #daa520 
228      gray = Color(128,128,128),     #808080 
229      green = Color(0,128,0),     #008000 
230      greenyellow = Color(173,255,47),     #adff2f 
231      grey = Color(128,128,128),     #808080 
232      honeydew = Color(240,255,240),     #f0fff0 
233      hotpink = Color(255,105,180),     #ff69b4 
234      indianred = Color(205,92,92),     #cd5c5c 
235      indigo = Color(75,0,130),     #4b0082 
236      ivory = Color(255,255,240),     #fffff0 
237      khaki = Color(240,230,140),     #f0e68c 
238      lavender = Color(230,230,250),     #e6e6fa 
239      lavenderblush = Color(255,240,245),     #fff0f5 
240      lawngreen = Color(124,252,0),     #7cfc00 
241      lemonchiffon = Color(255,250,205),     #fffacd 
242      lightblue = Color(173,216,230),     #add8e6 
243      lightcoral = Color(240,128,128),     #f08080 
244      lightcyan = Color(224,255,255),     #e0ffff 
245      lightgoldenrodyellow = Color(250,250,210),     #fafad2 
246      lightgray = Color(211,211,211),     #d3d3d3 
247      lightgreen = Color(144,238,144),     #90ee90 
248      lightgrey = Color(211,211,211),     #d3d3d3 
249      lightpink = Color(255,182,193),     #ffb6c1 
250      lightsalmon = Color(255,160,122),     #ffa07a 
251      lightseagreen = Color(32,178,170),     #20b2aa 
252      lightskyblue = Color(135,206,250),     #87cefa 
253      lightslategray = Color(119,136,153),     #778899 
254      lightslategrey = Color(119,136,153),     #778899 
255      lightsteelblue = Color(176,196,222),     #b0c4de 
256      lightyellow = Color(255,255,224),     #ffffe0 
257      lime = Color(0,255,0),     #00ff00 
258      limegreen = Color(50,205,50),     #32cd32 
259      linen = Color(250,240,230),     #faf0e6 
260      magenta = Color(255,0,255),     #ff00ff 
261      maroon = Color(128,0,0),     #800000 
262      mediumaquamarine = Color(102,205,170),     #66cdaa 
263      mediumblue = Color(0,0,205),     #0000cd 
264      mediumorchid = Color(186,85,211),     #ba55d3 
265      mediumpurple = Color(147,112,219),     #9370db 
266      mediumseagreen = Color(60,179,113),     #3cb371 
267      mediumslateblue = Color(123,104,238),     #7b68ee 
268      mediumspringgreen = Color(0,250,154),     #00fa9a 
269      mediumturquoise = Color(72,209,204),     #48d1cc 
270      mediumvioletred = Color(199,21,133),     #c71585 
271      midnightblue = Color(25,25,112),     #191970 
272      mintcream = Color(245,255,250),     #f5fffa 
273      mistyrose = Color(255,228,225),     #ffe4e1 
274      moccasin = Color(255,228,181),     #ffe4b5 
275      navajowhite = Color(255,222,173),     #ffdead 
276      navy = Color(0,0,128),     #000080 
277      oldlace = Color(253,245,230),     #fdf5e6 
278      olive = Color(128,128,0),     #808000 
279      olivedrab = Color(107,142,35),     #6b8e23 
280      orange = Color(255,165,0),     #ffa500 
281      orangered = Color(255,69,0),     #ff4500 
282      orchid = Color(218,112,214),     #da70d6 
283      palegoldenrod = Color(238,232,170),     #eee8aa 
284      palegreen = Color(152,251,152),     #98fb98 
285      paleturquoise = Color(175,238,238),     #afeeee 
286      palevioletred = Color(219,112,147),     #db7093 
287      papayawhip = Color(255,239,213),     #ffefd5 
288      peachpuff = Color(255,218,185),     #ffdab9 
289      peru = Color(205,133,63),     #cd853f 
290      pink = Color(255,192,203),     #ffc0cb 
291      plum = Color(221,160,221),     #dda0dd 
292      powderblue = Color(176,224,230),     #b0e0e6 
293      purple = Color(128,0,128),     #800080 
294      red = Color(255,0,0),     #ff0000 
295      rosybrown = Color(188,143,143),     #bc8f8f 
296      royalblue = Color(65,105,225),     #4169e1 
297      saddlebrown = Color(139,69,19),     #8b4513 
298      salmon = Color(250,128,114),     #fa8072 
299      sandybrown = Color(244,164,96),     #f4a460 
300      seagreen = Color(46,139,87),     #2e8b57 
301      seashell = Color(255,245,238),     #fff5ee 
302      sienna = Color(160,82,45),     #a0522d 
303      silver = Color(192,192,192),     #c0c0c0 
304      skyblue = Color(135,206,235),     #87ceeb 
305      slateblue = Color(106,90,205),     #6a5acd 
306      slategray = Color(112,128,144),     #708090 
307      slategrey = Color(112,128,144),     #708090 
308      snow = Color(255,250,250),     #fffafa 
309      springgreen = Color(0,255,127),     #00ff7f 
310      steelblue = Color(70,130,180),     #4682b4 
311      tan = Color(210,180,140),     #d2b48c 
312      teal = Color(0,128,128),     #008080 
313      thistle = Color(216,191,216),     #d8bfd8 
314      tomato = Color(255,99,71),     #ff6347 
315      turquoise = Color(64,224,208),     #40e0d0 
316      violet = Color(238,130,238),     #ee82ee 
317      wheat = Color(245,222,179),     #f5deb3 
318      white = Color(255,255,255),     #ffffff 
319      whitesmoke = Color(245,245,245),     #f5f5f5 
320      yellow = Color(255,255,0),     #ffff00 
321      yellowgreen = Color(154,205,50)     #9acd32 
322      ) 
323
```

  


| Home | Trees | Indices | Help | WebLogo |
| --- | --- | --- | --- | --- |

|  |  |
| --- | --- |
| Generated by Epydoc 3.0.1 on Mon Jan 30 17:27:19 2012 | http://epydoc.sourceforge.net |
