# Supplementary material for: DIVERSITY in binding, regulation, and evolution revealed from high-throughput ChIP
Source: PLoS Comput Biol. 2018 Apr 23;14(4):e1006090. doi: 10.1371/journal.pcbi.1006090 (PMC5933800; doi:10.1371/journal.pcbi.1006090)
Supplement: S1 File — (GZ) [file pcbi.1006090.s009.tar.gz › DIVERSITY-master/weblogoMod/apidocs/weblogolib.color.Color-class.html]

xml version="1.0" encoding="ascii"?


weblogolib.color.Color


| Home | Trees | Indices | Help | WebLogo |
| --- | --- | --- | --- | --- |

Package weblogolib ::
Module color ::
Class Color

# Class Color

source code

```
Color specifications using CSS2 (Cascading Style Sheet) syntax.

http://www.w3.org/TR/REC-CSS2/syndata.html#color-units

Usage:

red = Color(255,0,0)
red = Color(1., 0., 0.)
red = Color.by_name("red")
red = Color.from_rgb(1.,0.,0.)
red = Color.from_rgb(255,0,0)
red = Color.from_hsl(0.,1., 0.5)

red = Color.from_string("red") 
red = Color.from_string("RED") 
red = Color.from_string("#F00") 
red = Color.from_string("#FF0000") 
red = Color.from_string("rgb(255, 0, 0)")
red = Color.from_string("rgb(100%, 0%, 0%)")
red = Color.from_string("hsl(0, 100%, 50%)")
```


Instance Methods

|  |  |
| --- | --- |
| \_\_init\_\_(self, red, green, blue) | source code |

|  |  |
| --- | --- |
| \_\_eq\_\_(self, other) | source code |

|  |  |
| --- | --- |
| \_\_repr\_\_(self) | source code |


Class Methods

|  |  |
| --- | --- |
| from\_rgb(cls, r, g, b) | source code |

|  |  |
| --- | --- |
| from\_hsl(cls, hue\_angle, saturation, lightness) | source code |

|  |  |
| --- | --- |
| from\_string(cls, string) | source code |


Static Methods

|  |  |
| --- | --- |
| names()  Return a list of standard color names. | source code |

|  |  |
| --- | --- |
| by\_name(string) | source code |

| Home | Trees | Indices | Help | WebLogo |
| --- | --- | --- | --- | --- |

|  |  |
| --- | --- |
| Generated by Epydoc 3.0.1 on Mon Jan 30 17:27:18 2012 | http://epydoc.sourceforge.net |
