# Supplementary material for: DIVERSITY in binding, regulation, and evolution revealed from high-throughput ChIP
Source: PLoS Comput Biol. 2018 Apr 23;14(4):e1006090. doi: 10.1371/journal.pcbi.1006090 (PMC5933800; doi:10.1371/journal.pcbi.1006090)
Supplement: S1 File — (GZ) [file pcbi.1006090.s009.tar.gz › DIVERSITY-master/weblogoMod/apidocs/weblogolib.colorscheme-module.html]

xml version="1.0" encoding="ascii"?


weblogolib.colorscheme


| Home | Trees | Indices | Help | WebLogo |
| --- | --- | --- | --- | --- |

Package weblogolib ::
Module colorscheme

# Module colorscheme

source code

```
Popular color codings for nucleic and amino acids. 

Classes:
    ColorScheme -- A color scheme
    ColorGroup  
    
    
Generic
    monochrome

Nucleotides
    nucleotide
    base pairing

Amino Acid
    hydrophobicity
    chemistry
    charge
    taylor

Status : Beta - Needs documentation.
```


|  |  |
| --- | --- |
| Classes | |
|  | ColorScheme  A coloring of an alphabet. |
|  | ColorGroup  Associate a group of symbols with a color |


|  |  |
| --- | --- |
| Variables | |
|  | monochrome = `ColorScheme([])` |
|  | nucleotide = `ColorScheme([ColorGroup("G", "orange"), ColorGrou...` |
|  | base\_pairing = `ColorScheme([ColorGroup("TAU", "darkorange", "W...` |
|  | hydrophobicity = `ColorScheme([ColorGroup("RKDENQ", "black", "h...` |
|  | chemistry = `ColorScheme([ColorGroup("GSTYC", "green", "polar")...` |
|  | charge = `ColorScheme([ColorGroup("KRH", "blue", "Positive"), C...` |
|  | taylor = `ColorScheme([ColorGroup('A', '#CCFF00'), ColorGroup('...` |


Variables Details

### nucleotide

Value:
:   ```
    ColorScheme([ColorGroup("G", "orange"), ColorGroup("TU", "red"), Color
    Group("C", "blue"), ColorGroup("A", "green")])
    ```

### base\_pairing

Value:
:   ```
    ColorScheme([ColorGroup("TAU", "darkorange", "Weak (2 Watson-Crick hyd
    rogen bonds)"), ColorGroup("GC", "blue", "Strong (3 Watson-Crick hydro
    gen bonds)")],)
    ```

### hydrophobicity

Value:
:   ```
    ColorScheme([ColorGroup("RKDENQ", "black", "hydrophilic"), ColorGroup(
    "SGHTAP", "green", "neutral"), ColorGroup("YVMCLFIW", "blue", "hydroph
    obic")], alphabet= seq.unambiguous_protein_alphabet)
    ```

### chemistry

Value:
:   ```
    ColorScheme([ColorGroup("GSTYC", "green", "polar"), ColorGroup("NQ", "
    purple", "neutral"), ColorGroup("KRH", "blue", "basic"), ColorGroup("D
    E", "red", "acidic"), ColorGroup("PAWFLIMV", "black", "hydrophobic")],
     alphabet= seq.unambiguous_protein_alphabet)
    ```

### charge

Value:
:   ```
    ColorScheme([ColorGroup("KRH", "blue", "Positive"), ColorGroup("DE", "
    red", "Negative")], alphabet= seq.unambiguous_protein_alphabet)
    ```

### taylor

Value:
:   ```
    ColorScheme([ColorGroup('A', '#CCFF00'), ColorGroup('C', '#FFFF00'), C
    olorGroup('D', '#FF0000'), ColorGroup('E', '#FF0066'), ColorGroup('F',
     '#00FF66'), ColorGroup('G', '#FF9900'), ColorGroup('H', '#0066FF'), C
    olorGroup('I', '#66FF00'), ColorGroup('K', '#6600FF'), ColorGroup('L',
     '#33FF00'), ColorGroup('M', '#00FF00'), ColorGroup('N', '#CC00FF'), C
    olorGroup('P', '#FFCC00'), ColorGroup('Q', '#FF00CC'), ColorGroup('R',
     '#0000FF'), ColorGroup('S', '#FF3300'), ColorGroup('T', '#FF6600'), C
    olorGroup('V', '#99FF00'), ColorGroup('W', '#00CCFF'), ColorGroup('Y',
    ...
    ```

  


| Home | Trees | Indices | Help | WebLogo |
| --- | --- | --- | --- | --- |

|  |  |
| --- | --- |
| Generated by Epydoc 3.0.1 on Mon Jan 30 17:27:18 2012 | http://epydoc.sourceforge.net |
