# Supplementary material for: DIVERSITY in binding, regulation, and evolution revealed from high-throughput ChIP
Source: PLoS Comput Biol. 2018 Apr 23;14(4):e1006090. doi: 10.1371/journal.pcbi.1006090 (PMC5933800; doi:10.1371/journal.pcbi.1006090)
Supplement: S1 File — (GZ) [file pcbi.1006090.s009.tar.gz › DIVERSITY-master/weblogoMod/apidocs/weblogolib.colorscheme-pysrc.html]

xml version="1.0" encoding="ascii"?


weblogolib.colorscheme


| Home | Trees | Indices | Help | WebLogo |
| --- | --- | --- | --- | --- |

Package weblogolib ::
Module colorscheme

# Source Code for Module weblogolib.colorscheme

```
  1   
  2  #  Copyright (c) 2003-2005 The Regents of the University of California. 
  3  #  Copyright (c) 2005 Gavin E. Crooks 
  4   
  5  #  This software is distributed under the MIT Open Source License. 
  6  #  <http://www.opensource.org/licenses/mit-license.html> 
  7  # 
  8  #  Permission is hereby granted, free of charge, to any person obtaining a  
  9  #  copy of this software and associated documentation files (the "Software"), 
 10  #  to deal in the Software without restriction, including without limitation 
 11  #  the rights to use, copy, modify, merge, publish, distribute, sublicense, 
 12  #  and/or sell copies of the Software, and to permit persons to whom the 
 13  #  Software is furnished to do so, subject to the following conditions: 
 14  # 
 15  #  The above copyright notice and this permission notice shall be included 
 16  #  in all copies or substantial portions of the Software. 
 17  # 
 18  #  THE SOFTWARE IS PROVIDED "AS IS", WITHOUT WARRANTY OF ANY KIND, EXPRESS OR  
 19  #  IMPLIED, INCLUDING BUT NOT LIMITED TO THE WARRANTIES OF MERCHANTABILITY,  
 20  #  FITNESS FOR A PARTICULAR PURPOSE AND NONINFRINGEMENT. IN NO EVENT SHALL THE 
 21  #  AUTHORS OR COPYRIGHT HOLDERS BE LIABLE FOR ANY CLAIM, DAMAGES OR OTHER  
 22  #  LIABILITY, WHETHER IN AN ACTION OF CONTRACT, TORT OR OTHERWISE, ARISING FROM, 
 23  #  OUT OF OR IN CONNECTION WITH THE SOFTWARE OR THE USE OR OTHER DEALINGS IN  
 24  #  THE SOFTWARE. 
 25   
 26  """ Popular color codings for nucleic and amino acids.  
 27   
 28  Classes: 
 29      ColorScheme -- A color scheme 
 30      ColorGroup   
 31       
 32       
 33  Generic 
 34      monochrome 
 35   
 36  Nucleotides 
 37      nucleotide 
 38      base pairing 
 39   
 40  Amino Acid 
 41      hydrophobicity 
 42      chemistry 
 43      charge 
 44      taylor 
 45   
 46  Status : Beta - Needs documentation. 
 47   
 48  """ 
 49  # Good online references include bioruby and the JalView alignment editor. 
 50  # Clamp, M., Cuff, J., Searle, S. M. and Barton, G. J. (2004),  
 51  # "The Jalview Java Alignment Editor," Bioinformatics, 12, 426-7 
 52  # http://www.jalview.org 
 53   
 54   
 55  from corebio import seq 
 56  from color import Color 
 57   


58 -class ColorScheme(object):


59      """ A coloring of an alphabet. 
 60       
 61      title : string            -- A human readable description 
 62      defualt_color : Color           -- 
 63      groups : list of color groups  
 64      alphabet : string               -- The set of colored symbols 
 65      color -- A map between a symbol and a Coloring 
 66       
 67   
 68      """ 
 69       


70 -    def __init__(self,  
 71                  groups = [],  
 72                  title = "",  
 73                  description = "", 
 74                  default_color = "black",  
 75                  alphabet = seq.generic_alphabet) :


76          """  """ 
 77          self.title= title 
 78          self.description = description 
 79          self.default_color = Color.from_string(default_color) 
 80          self.groups = groups 
 81          self.alphabet = alphabet 
 82               
 83          color = {} 
 84          for cg in groups : 
 85              for s in cg.symbols : 
 86                  color[s] = cg.color 
 87                  if s not in alphabet : 
 88                      raise KeyError("Colored symbol does not exist in alphabet.") 
 89          self._color = color

 90   


91 -    def color(self, symbol) :


92          if symbol in self._color : 
 93              return self._color[symbol] 
 94          return self.default_color

 95           


96 -class ColorGroup(object) :


97      """Associate a group of symbols with a color""" 


98 -    def __init__(self, symbols, color, description=None) :


99          self.symbols = symbols               
100          self.color =  Color.from_string(color) 
101          self.description = description

102   
103   
104            
105  monochrome = ColorScheme([]) # This list intentionally left blank 
106                  
107  # From makelogo 
108  nucleotide = ColorScheme([ 
109      ColorGroup("G", "orange"), 
110      ColorGroup("TU", "red"), 
111      ColorGroup("C",  "blue"), 
112      ColorGroup("A",  "green")  
113      ])  
114   
115  base_pairing = ColorScheme([ 
116      ColorGroup("TAU",  "darkorange", "Weak (2 Watson-Crick hydrogen bonds)"), 
117      ColorGroup("GC",    "blue", "Strong (3 Watson-Crick hydrogen bonds)")], 
118      ) 
119   
120  # From Crooks2004c-Proteins-SeqStr.pdf 
121  hydrophobicity = ColorScheme([ 
122      ColorGroup( "RKDENQ",   "black", "hydrophilic"), 
123      ColorGroup( "SGHTAP",   "green", "neutral"  ), 
124      ColorGroup( "YVMCLFIW", "blue",  "hydrophobic") ], 
125      alphabet = seq.unambiguous_protein_alphabet 
126      ) 
127   
128  # from makelogo 
129  chemistry = ColorScheme([ 
130    ColorGroup( "GSTYC",  "green",   "polar"), 
131    ColorGroup( "NQ",      "purple", "neutral"),  
132    ColorGroup( "KRH",     "blue",   "basic"), 
133    ColorGroup( "DE",      "red",    "acidic"), 
134    ColorGroup("PAWFLIMV", "black",  "hydrophobic") ], 
135    alphabet = seq.unambiguous_protein_alphabet 
136    )    
137   
138  charge = ColorScheme([ 
139      ColorGroup("KRH", "blue", "Positive" ), 
140      ColorGroup( "DE", "red", "Negative") ], 
141      alphabet = seq.unambiguous_protein_alphabet 
142      ) 
143   
144   
145  taylor = ColorScheme([ 
146      ColorGroup( 'A', '#CCFF00' ), 
147      ColorGroup( 'C', '#FFFF00' ), 
148      ColorGroup( 'D', '#FF0000'), 
149      ColorGroup( 'E', '#FF0066' ), 
150      ColorGroup( 'F', '#00FF66'), 
151      ColorGroup( 'G', '#FF9900'), 
152      ColorGroup( 'H', '#0066FF'), 
153      ColorGroup( 'I', '#66FF00'), 
154      ColorGroup( 'K', '#6600FF'), 
155      ColorGroup( 'L', '#33FF00'), 
156      ColorGroup( 'M', '#00FF00'), 
157      ColorGroup( 'N', '#CC00FF'), 
158      ColorGroup( 'P', '#FFCC00'), 
159      ColorGroup( 'Q', '#FF00CC'), 
160      ColorGroup( 'R', '#0000FF'), 
161      ColorGroup( 'S', '#FF3300'), 
162      ColorGroup( 'T', '#FF6600'), 
163      ColorGroup( 'V', '#99FF00'), 
164      ColorGroup( 'W', '#00CCFF'), 
165      ColorGroup( 'Y', '#00FFCC')], 
166      title = "Taylor", 
167      description = "W. Taylor, Protein Engineering, Vol 10 , 743-746 (1997)", 
168      alphabet = seq.unambiguous_protein_alphabet 
169      ) 
170
```

  


| Home | Trees | Indices | Help | WebLogo |
| --- | --- | --- | --- | --- |

|  |  |
| --- | --- |
| Generated by Epydoc 3.0.1 on Mon Jan 30 17:27:18 2012 | http://epydoc.sourceforge.net |
