# Supplementary material for: DIVERSITY in binding, regulation, and evolution revealed from high-throughput ChIP
Source: PLoS Comput Biol. 2018 Apr 23;14(4):e1006090. doi: 10.1371/journal.pcbi.1006090 (PMC5933800; doi:10.1371/journal.pcbi.1006090)
Supplement: S1 File — (GZ) [file pcbi.1006090.s009.tar.gz › DIVERSITY-master/weblogoMod/apidocs/weblogolib.colorscheme.ColorScheme-class.html]

xml version="1.0" encoding="ascii"?


weblogolib.colorscheme.ColorScheme


| Home | Trees | Indices | Help | WebLogo |
| --- | --- | --- | --- | --- |

Package weblogolib ::
Module colorscheme ::
Class ColorScheme

# Class ColorScheme

source code

```
object --+
         |
        ColorScheme
```

---

```
A coloring of an alphabet.

title : string            -- A human readable description
defualt_color : Color           --
groups : list of color groups 
alphabet : string               -- The set of colored symbols
color -- A map between a symbol and a Coloring
```


Instance Methods

|  |  |
| --- | --- |
| \_\_init\_\_(self, groups=[], title="", description="", default\_color="black", alphabet=seq.generic\_alphabet) | source code |

|  |  |
| --- | --- |
| color(self, symbol) | source code |

**Inherited from `object`**:
`__delattr__`,
`__getattribute__`,
`__hash__`,
`__new__`,
`__reduce__`,
`__reduce_ex__`,
`__repr__`,
`__setattr__`,
`__str__`


Properties

**Inherited from `object`**:
`__class__`


Method Details

|  |  |
| --- | --- |
| \_\_init\_\_(self, groups=[], title="", description="", default\_color="black", alphabet=seq.generic\_alphabet)  *(Constructor)* | source code |

Overrides: object.\_\_init\_\_

  


| Home | Trees | Indices | Help | WebLogo |
| --- | --- | --- | --- | --- |

|  |  |
| --- | --- |
| Generated by Epydoc 3.0.1 on Mon Jan 30 17:27:18 2012 | http://epydoc.sourceforge.net |
