# Supplementary material for: DIVERSITY in binding, regulation, and evolution revealed from high-throughput ChIP
Source: PLoS Comput Biol. 2018 Apr 23;14(4):e1006090. doi: 10.1371/journal.pcbi.1006090 (PMC5933800; doi:10.1371/journal.pcbi.1006090)
Supplement: S1 File — (GZ) [file pcbi.1006090.s009.tar.gz › DIVERSITY-master/weblogoMod/apidocs/weblogolib.LogoData-class.html]

xml version="1.0" encoding="ascii"?


weblogolib.LogoData


| Home | Trees | Indices | Help | WebLogo |
| --- | --- | --- | --- | --- |

Package weblogolib ::
Class LogoData

# Class LogoData

source code

```
object --+
         |
        LogoData
```

---

```
The data needed to generate a sequence logo.
   
- alphabet 
- length
- counts  -- An array of character counts
- entropy -- The relative entropy of each column
- entropy_interval -- entropy confidence interval
```


Instance Methods

|  |  |
| --- | --- |
| \_\_init\_\_(self, length=None, alphabet=None, counts=None, entropy=None, entropy\_interval=None, weight=None)  Creates a new LogoData object | source code |

|  |  |
| --- | --- |
| \_\_str\_\_(self)  str(x) | source code |

**Inherited from `object`**:
`__delattr__`,
`__getattribute__`,
`__hash__`,
`__new__`,
`__reduce__`,
`__reduce_ex__`,
`__repr__`,
`__setattr__`


Class Methods

|  |  |
| --- | --- |
| from\_counts(cls, alphabet, counts, prior=None)  Build a LogoData object from counts. | source code |

|  |  |
| --- | --- |
| from\_seqs(cls, seqs, prior=None)  Build a LogoData object from a SeqList, a list of sequences. | source code |


Properties

**Inherited from `object`**:
`__class__`


Method Details

|  |  |
| --- | --- |
| \_\_init\_\_(self, length=None, alphabet=None, counts=None, entropy=None, entropy\_interval=None, weight=None)  *(Constructor)* | source code |

```
Creates a new LogoData object
```

Overrides: object.\_\_init\_\_

|  |  |
| --- | --- |
| \_\_str\_\_(self)  *(Informal representation operator)* | source code |

```
str(x)
```

Overrides: object.\_\_str\_\_: *(inherited documentation)*

  


| Home | Trees | Indices | Help | WebLogo |
| --- | --- | --- | --- | --- |

|  |  |
| --- | --- |
| Generated by Epydoc 3.0.1 on Mon Jan 30 17:27:18 2012 | http://epydoc.sourceforge.net |
