# Supplementary material for: DIVERSITY in binding, regulation, and evolution revealed from high-throughput ChIP
Source: PLoS Comput Biol. 2018 Apr 23;14(4):e1006090. doi: 10.1371/journal.pcbi.1006090 (PMC5933800; doi:10.1371/journal.pcbi.1006090)
Supplement: S1 File — (GZ) [file pcbi.1006090.s009.tar.gz › DIVERSITY-master/weblogoMod/apidocs/weblogolib.LogoFormat-class.html]

xml version="1.0" encoding="ascii"?


weblogolib.LogoFormat


| Home | Trees | Indices | Help | WebLogo |
| --- | --- | --- | --- | --- |

Package weblogolib ::
Class LogoFormat

# Class LogoFormat

source code

```
 object --+    
          |    
LogoOptions --+
              |
             LogoFormat
```

---

```
Specifies the format of the logo. Requires LogoData and LogoOptions 
objects.

>>> data = LogoData.from_seqs(seqs )
>>> options = LogoOptions()
>>> options.title = "A Logo Title"
>>> format = LogoFormat(data, options) 

Raises an ArgumentError if arguments are invalid.
```


Instance Methods

|  |  |
| --- | --- |
| \_\_init\_\_(self, data, options=None)  Create a new LogoFormat instance. | source code |

**Inherited from `LogoOptions`**:
`__repr__`

**Inherited from `object`**:
`__delattr__`,
`__getattribute__`,
`__hash__`,
`__new__`,
`__reduce__`,
`__reduce_ex__`,
`__setattr__`,
`__str__`


Properties

**Inherited from `object`**:
`__class__`


Method Details

|  |  |
| --- | --- |
| \_\_init\_\_(self, data, options=None)  *(Constructor)* | source code |

```
Create a new LogoFormat instance.
```

Overrides: object.\_\_init\_\_

  


| Home | Trees | Indices | Help | WebLogo |
| --- | --- | --- | --- | --- |

|  |  |
| --- | --- |
| Generated by Epydoc 3.0.1 on Mon Jan 30 17:27:18 2012 | http://epydoc.sourceforge.net |
