# Supplementary material for: DIVERSITY in binding, regulation, and evolution revealed from high-throughput ChIP
Source: PLoS Comput Biol. 2018 Apr 23;14(4):e1006090. doi: 10.1371/journal.pcbi.1006090 (PMC5933800; doi:10.1371/journal.pcbi.1006090)
Supplement: S1 File — (GZ) [file pcbi.1006090.s009.tar.gz › DIVERSITY-master/weblogoMod/apidocs/weblogolib.logomath-module.html]

xml version="1.0" encoding="ascii"?


weblogolib.logomath


| Home | Trees | Indices | Help | WebLogo |
| --- | --- | --- | --- | --- |

Package weblogolib ::
Module logomath

# Module logomath

source code


|  |  |
| --- | --- |
| Classes | |
|  | Dirichlet  The Dirichlet probability distribution. |
|  | Gamma  The gamma probability distribution. |


Functions

|  |  |
| --- | --- |
| find\_root(f, x, y=None, fprime=None, tolerance=1.48e-8, max\_iterations=50)  Return argument 'x' of the function f(x), such that f(x)=0 to within the given tolerance. | source code |


Function Details

|  |  |
| --- | --- |
| find\_root(f, x, y=None, fprime=None, tolerance=1.48e-8, max\_iterations=50) | source code |

```
Return argument 'x' of the function f(x), such that f(x)=0 to
within the given tolerance. 

f : The function to optimize, f(x)
x : The initial guess
y : An optional second guess that shoudl bracket the root.
fprime : The derivate of f'(x) (Optional)
tolerance : The error bounds 
max_iterations : Maximum number of iterations

Raises:
    ArithmeticError :
        Failure to converge to the given tolerence

Notes:
    Uses Newton-Raphson algorihtm if f'(x) is given, else uses bisect if
    y is given and brackets the root, else uses secant. 

Status : Beta (Not fully tested)
```

  


| Home | Trees | Indices | Help | WebLogo |
| --- | --- | --- | --- | --- |

|  |  |
| --- | --- |
| Generated by Epydoc 3.0.1 on Mon Jan 30 17:27:18 2012 | http://epydoc.sourceforge.net |
