# Supplementary material for: DIVERSITY in binding, regulation, and evolution revealed from high-throughput ChIP
Source: PLoS Comput Biol. 2018 Apr 23;14(4):e1006090. doi: 10.1371/journal.pcbi.1006090 (PMC5933800; doi:10.1371/journal.pcbi.1006090)
Supplement: S1 File — (GZ) [file pcbi.1006090.s009.tar.gz › DIVERSITY-master/weblogoMod/apidocs/weblogolib.logomath-pysrc.html]

xml version="1.0" encoding="ascii"?


weblogolib.logomath


| Home | Trees | Indices | Help | WebLogo |
| --- | --- | --- | --- | --- |

Package weblogolib ::
Module logomath

# Source Code for Module weblogolib.logomath

```
  1  #!/usr/bin/env python 
  2   
  3  # -------------------------------- WebLogo -------------------------------- 
  4   
  5  #  Copyright (c) 2003-2004 The Regents of the University of California. 
  6  #  Copyright (c) 2005 Gavin E. Crooks 
  7  #  Copyright (c) 2006-2011, The Regents of the University of California, through  
  8  #  Lawrence Berkeley National Laboratory (subject to receipt of any required 
  9  #  approvals from the U.S. Dept. of Energy).  All rights reserved. 
 10   
 11  #  This software is distributed under the new BSD Open Source License. 
 12  #  <http://www.opensource.org/licenses/bsd-license.html> 
 13  # 
 14  #  Redistribution and use in source and binary forms, with or without  
 15  #  modification, are permitted provided that the following conditions are met:  
 16  # 
 17  #  (1) Redistributions of source code must retain the above copyright notice,  
 18  #  this list of conditions and the following disclaimer.  
 19  # 
 20  #  (2) Redistributions in binary form must reproduce the above copyright  
 21  #  notice, this list of conditions and the following disclaimer in the  
 22  #  documentation and or other materials provided with the distribution.  
 23  # 
 24  #  (3) Neither the name of the University of California, Lawrence Berkeley  
 25  #  National Laboratory, U.S. Dept. of Energy nor the names of its contributors  
 26  #  may be used to endorse or promote products derived from this software  
 27  #  without specific prior written permission.  
 28  # 
 29  #  THIS SOFTWARE IS PROVIDED BY THE COPYRIGHT HOLDERS AND CONTRIBUTORS "AS IS"  
 30  #  AND ANY EXPRESS OR IMPLIED WARRANTIES, INCLUDING, BUT NOT LIMITED TO, THE  
 31  #  IMPLIED WARRANTIES OF MERCHANTABILITY AND FITNESS FOR A PARTICULAR PURPOSE  
 32  #  ARE DISCLAIMED. IN NO EVENT SHALL THE COPYRIGHT OWNER OR CONTRIBUTORS BE  
 33  #  LIABLE FOR ANY DIRECT, INDIRECT, INCIDENTAL, SPECIAL, EXEMPLARY, OR  
 34  #  CONSEQUENTIAL DAMAGES (INCLUDING, BUT NOT LIMITED TO, PROCUREMENT OF  
 35  #  SUBSTITUTE GOODS OR SERVICES; LOSS OF USE, DATA, OR PROFITS; OR BUSINESS  
 36  #  INTERRUPTION) HOWEVER CAUSED AND ON ANY THEORY OF LIABILITY, WHETHER IN  
 37  #  CONTRACT, STRICT LIABILITY, OR TORT (INCLUDING NEGLIGENCE OR OTHERWISE)  
 38  #  ARISING IN ANY WAY OUT OF THE USE OF THIS SOFTWARE, EVEN IF ADVISED OF THE  
 39  #  POSSIBILITY OF SUCH DAMAGE.  
 40   
 41  from math import log, sqrt, exp 
 42   
 43  from numpy import array, asarray, float64, ones, zeros, int32,all,any, shape 
 44  import numpy as na 
 45  from corebio.moremath import * 
 46   
 47  import random 
 48   


49 -class Dirichlet(object) :


50      """The Dirichlet probability distribution. The Dirichlet is a continuous  
 51      multivariate probability distribution across non-negative unit length 
 52      vectors. In other words, the Dirichlet is a probability distribution of  
 53      probability distributions. It is conjugate to the multinomial 
 54      distribution and is widely used in Bayesian statistics. 
 55       
 56      The Dirichlet probability distribution of order K-1 is  
 57   
 58       p(theta_1,...,theta_K) d theta_1 ... d theta_K =  
 59          (1/Z) prod_i=1,K theta_i^{alpha_i - 1} delta(1 -sum_i=1,K theta_i) 
 60   
 61      The normalization factor Z can be expressed in terms of gamma functions: 
 62   
 63        Z = {prod_i=1,K Gamma(alpha_i)} / {Gamma( sum_i=1,K alpha_i)}   
 64   
 65      The K constants, alpha_1,...,alpha_K, must be positive. The K parameters,  
 66      theta_1,...,theta_K are nonnegative and sum to 1. 
 67       
 68      Status: 
 69          Alpha 
 70      """ 
 71      __slots__ = 'alpha', '_total', '_mean',  
 72       
 73       
 74       
 75   


76 -    def __init__(self, alpha) :


77          """ 
 78          Args: 
 79              - alpha  -- The parameters of the Dirichlet prior distribution. 
 80                          A vector of non-negative real numbers.   
 81          """ 
 82          # TODO: Check that alphas are positive 
 83          #TODO : what if alpha's not one dimensional? 
 84          self.alpha = asarray(alpha, float64) 
 85           
 86          self._total = sum(alpha) 
 87          self._mean = None

 88           
 89           


90 -    def sample(self) :


91          """Return a randomly generated probability vector. 
 92           
 93          Random samples are generated by sampling K values from gamma 
 94          distributions with parameters a=\alpha_i, b=1, and renormalizing.  
 95       
 96          Ref: 
 97              A.M. Law, W.D. Kelton, Simulation Modeling and Analysis (1991). 
 98          Authors: 
 99              Gavin E. Crooks <gec@compbio.berkeley.edu> (2002) 
100          """ 
101          alpha = self.alpha 
102          K = len(alpha) 
103          theta = zeros( (K,), float64) 
104   
105          for k in range(K): 
106              theta[k] = random.gammavariate(alpha[k], 1.0)  
107          theta /= sum(theta) 
108   
109          return theta

110   


111 -    def mean(self) :


112          if  self._mean ==None: 
113              self._mean = self.alpha / self._total 
114          return self._mean

115       


116 -    def covariance(self) :


117          alpha = self.alpha 
118          A = sum(alpha) 
119          #A2 = A * A 
120          K = len(alpha) 
121          cv = zeros( (K,K), float64)  
122           
123          for i in range(K) : 
124              cv[i,i] = alpha[i] * (1. - alpha[i]/A) / (A * (A+1.) ) 
125           
126          for i in range(K) : 
127              for j in range(i+1,K) : 
128                  v = - alpha[i] * alpha[j] / (A * A * (A+1.) ) 
129                  cv[i,j] = v 
130                  cv[j,i] = v 
131          return cv

132           


133 -    def mean_x(self, x) :


134          x = asarray(x, float64) 
135          if shape(x) != shape(self.alpha) : 
136              raise ValueError("Argument must be same dimension as Dirichlet") 
137          return sum( x * self.mean())

138   


139 -    def variance_x(self, x) :


140          x = asarray(x, float64) 
141          if shape(x) != shape(self.alpha) : 
142              raise ValueError("Argument must be same dimension as Dirichlet")         
143               
144          cv = self.covariance() 
145          var = na.dot(na.dot(na.transpose( x), cv), x) 
146          return var

147   
148   


149 -    def mean_entropy(self) :


150          """Calculate the average entropy of probabilities sampled 
151          from this Dirichlet distribution.  
152           
153          Returns: 
154              The average entropy. 
155               
156          Ref: 
157              Wolpert & Wolf, PRE 53:6841-6854 (1996) Theorem 7 
158              (Warning: this paper contains typos.) 
159          Status: 
160              Alpha 
161          Authors: 
162              GEC 2005 
163       
164          """ 
165          # TODO: Optimize 
166          alpha = self.alpha 
167          A = float(sum(alpha)) 
168          ent = 0.0 
169          for a in alpha: 
170              if a>0 : ent += - 1.0 * a * digamma( 1.0+a) # FIXME: Check 
171          ent /= A 
172          ent += digamma(A+1.0) 
173          return ent

174   
175   
176   


177 -    def variance_entropy(self):


178          """Calculate the variance of the Dirichlet entropy.  
179   
180          Ref: 
181              Wolpert & Wolf, PRE 53:6841-6854 (1996) Theorem 8 
182              (Warning: this paper contains typos.) 
183          """ 
184          alpha = self.alpha 
185          A = float(sum(alpha)) 
186          A2 = A * (A+1) 
187          L = len(alpha) 
188           
189          dg1 = zeros( (L) , float64) 
190          dg2 = zeros( (L) , float64) 
191          tg2 = zeros( (L) , float64)         
192           
193          for i in range(L) : 
194              dg1[i] = digamma(alpha[i] + 1.0) 
195              dg2[i] = digamma(alpha[i] + 2.0) 
196              tg2[i] = trigamma(alpha[i] + 2.0) 
197   
198          dg_Ap2 = digamma( A+2. ) 
199          tg_Ap2 = trigamma( A+2. ) 
200           
201          mean = self.mean_entropy() 
202          var = 0.0 
203       
204          for i in range(L) : 
205              for j in range(L) : 
206                  if i != j : 
207                      var += ( 
208                          ( dg1[i] - dg_Ap2 ) * (dg1[j] - dg_Ap2 ) - tg_Ap2  
209                          ) * (alpha[i] * alpha[j] ) / A2 
210                  else :  
211                      var += ( 
212                          ( dg2[i] - dg_Ap2 ) **2 + ( tg2[i] - tg_Ap2 ) 
213                          ) * ( alpha[i] * (alpha[i]+1.) ) / A2 
214   
215          var -= mean**2 
216          return var

217           
218           
219           


220 -    def mean_relative_entropy(self, pvec) :


221          ln_p = na.log(pvec) 
222          return - self.mean_x(ln_p) - self.mean_entropy()

223       
224       


225 -    def variance_relative_entropy(self, pvec) :


226          ln_p = na.log(pvec) 
227          return self.variance_x(ln_p) + self.variance_entropy()

228       
229       


230 -    def interval_relative_entropy(self, pvec, frac) :


231          mean = self.mean_relative_entropy(pvec)  
232          variance = self.variance_relative_entropy(pvec)  
233          sd = sqrt(variance) 
234           
235          # If the variance is small, use the standard 95%  
236          # confidence interval: mean +/- 1.96 * sd 
237          if mean/sd >3.0 : 
238              return max(0.0, mean - sd*1.96), mean + sd*1.96 
239           
240          g = Gamma.from_mean_variance(mean, variance) 
241          low_limit = g.inverse_cdf( (1.-frac)/2.) 
242          high_limit = g.inverse_cdf( 1. - (1.-frac)/2. ) 
243           
244          return low_limit, high_limit

245   
246   


247 -class Gamma(object) :


248      """The gamma probability distribution. (Not to be confused with the 
249      gamma function.) 
250       
251       
252      """ 
253      __slots__ = 'alpha', 'beta' 
254       


255 -    def __init__(self, alpha, beta) :


256          if alpha <=0.0 : 
257              raise ValueError("alpha must be positive") 
258          if beta <=0.0 : 
259              raise ValueError("beta must be positive") 
260          self.alpha = alpha 
261          self.beta = beta

262           
263   


264 -    def from_shape_scale(cls, shape, scale) :


265          return cls( shape, 1./scale)

266      from_shape_scale = classmethod(from_shape_scale) 
267       
268   


269 -    def from_mean_variance(cls, mean, variance) :


270          alpha = mean **2 / variance 
271          beta = alpha/mean 
272          return cls(alpha, beta)

273      from_mean_variance = classmethod(from_mean_variance) 
274   


275 -    def mean(self) :


276          return self.alpha / self.beta

277           


278 -    def variance(self) :


279          return self.alpha / (self.beta**2)

280   
281           


282 -    def sample(self) :


283          return random.gammavariate(self.alpha, 1./self.beta)

284   


285 -    def pdf(self, x) :


286          if x==0.0 : return 0.0 
287          a = self.alpha 
288          b = self.beta 
289          return (x**(a-1.)) * exp(- b*x )* (b**a) / gamma(a)

290           


291 -    def cdf(self, x) :


292          return 1.0-normalized_incomplete_gamma(self.alpha, self.beta*x)

293   


294 -    def inverse_cdf(self, p) :


295          def rootof(x) : 
296              return self.cdf(exp(x)) - p

297       
298          return exp(find_root(  rootof, log(self.mean()) ) )

299   
300  # 
301   


302 -def find_root(f, x, y=None, fprime=None, tolerance=1.48e-8, max_iterations=50):


303      """Return argument 'x' of the function f(x), such that f(x)=0 to 
304      within the given tolerance.  
305       
306      f : The function to optimize, f(x) 
307      x : The initial guess 
308      y : An optional second guess that shoudl bracket the root. 
309      fprime : The derivate of f'(x) (Optional) 
310      tolerance : The error bounds  
311      max_iterations : Maximum number of iterations 
312       
313      Raises: 
314          ArithmeticError : 
315              Failure to converge to the given tolerence 
316   
317      Notes: 
318          Uses Newton-Raphson algorihtm if f'(x) is given, else uses bisect if 
319          y is given and brackets the root, else uses secant.  
320   
321      Status : Beta (Not fully tested) 
322      """ 
323   
324     
325      def secant (f, x, tolerance, max_iterations): 
326          x0 = x 
327          x1 = x0+ 1e-4 
328          v0 = f(x0) 
329          v1 = f(x1) 
330          x2 = 0 
331           
332          for i in range(max_iterations): 
333              # print x0, x1, v0, v1, x2-x0 
334              x2 = x1 - v1*(x1-x0)/(v1-v0) 
335              if abs(x2-x1) < tolerance : return x2         
336              x0 = x1 
337              v0 = v1         
338              x1 = x2 
339              v1 = f(x1) 
340   
341          raise ArithmeticError( 
342              "Failed to converge after %d iterations, value is %f" \ 
343                  % (max_iterations,x1) )

344   
345   
346      def bisect(f, a, b, tolerance, max_iterations) : 
347          fa = f(a) 
348          fb = f(b) 
349   
350          if fa==0: return a 
351          if fb==0: return b 
352                   
353          if fa*fb >0 : 
354              raise ArithmeticError("Start points do not bracket root.") 
355           
356          for i in range(max_iterations): 
357              #print a,b, fa, fb 
358              delta = b-a 
359              xm = a + 0.5*delta # Minimize roundoff in computing the midpoint 
360              fm = f(xm) 
361              if delta < tolerance : return xm 
362               
363              if fm * fa >0 : #   Root lies in interval [xm,b], replace a 
364                  a = xm 
365                  fa = fm 
366              else : #   Root lies in interval [a,xm], replace b 
367                  b = xm 
368                  fb = fm 
369   
370          raise ArithmeticError( 
371              "Failed to converge after %d iterations, value is %f" \ 
372                  % (max_iterations, x) ) 
373       
374       
375      def newton(f, x, fprime, tolerance, max_iterations) : 
376          x0 = x 
377          for i in range(max_iterations) : 
378              x1 = x0 - f(x0)/fprime(x0) 
379              if abs(x1-x0) < tolerance: return x1 
380              x0 = x1 
381               
382          raise ArithmeticError( 
383              "Failed to converge after %d iterations, value is %f" \ 
384                  % (max_iterations,x1) ) 
385   
386      if fprime is not None : 
387          return newton(f, x, fprime, tolerance, max_iterations) 
388      elif y is not None : 
389          return bisect(f, x, y, tolerance, max_iterations) 
390      else : 
391          return secant(f, x, tolerance, max_iterations) 
392
```

  


| Home | Trees | Indices | Help | WebLogo |
| --- | --- | --- | --- | --- |

|  |  |
| --- | --- |
| Generated by Epydoc 3.0.1 on Mon Jan 30 17:27:19 2012 | http://epydoc.sourceforge.net |
