# Supplementary material for: DIVERSITY in binding, regulation, and evolution revealed from high-throughput ChIP
Source: PLoS Comput Biol. 2018 Apr 23;14(4):e1006090. doi: 10.1371/journal.pcbi.1006090 (PMC5933800; doi:10.1371/journal.pcbi.1006090)
Supplement: S1 File — (GZ) [file pcbi.1006090.s009.tar.gz › DIVERSITY-master/weblogoMod/apidocs/weblogolib.logomath.Dirichlet-class.html]

xml version="1.0" encoding="ascii"?


weblogolib.logomath.Dirichlet


| Home | Trees | Indices | Help | WebLogo |
| --- | --- | --- | --- | --- |

Package weblogolib ::
Module logomath ::
Class Dirichlet

# Class Dirichlet

source code

```
object --+
         |
        Dirichlet
```

---

```
The Dirichlet probability distribution. The Dirichlet is a continuous 
multivariate probability distribution across non-negative unit length
vectors. In other words, the Dirichlet is a probability distribution of 
probability distributions. It is conjugate to the multinomial
distribution and is widely used in Bayesian statistics.

The Dirichlet probability distribution of order K-1 is 

 p(theta_1,...,theta_K) d theta_1 ... d theta_K = 
    (1/Z) prod_i=1,K theta_i^{alpha_i - 1} delta(1 -sum_i=1,K theta_i)

The normalization factor Z can be expressed in terms of gamma functions:

  Z = {prod_i=1,K Gamma(alpha_i)} / {Gamma( sum_i=1,K alpha_i)}  

The K constants, alpha_1,...,alpha_K, must be positive. The K parameters, 
theta_1,...,theta_K are nonnegative and sum to 1.

Status:
    Alpha
```


Instance Methods

|  |  |
| --- | --- |
| \_\_init\_\_(self, alpha)  Args: - alpha -- The parameters of the Dirichlet prior distribution. | source code |

|  |  |
| --- | --- |
| sample(self)  Return a randomly generated probability vector. | source code |

|  |  |
| --- | --- |
| mean(self) | source code |

|  |  |
| --- | --- |
| covariance(self) | source code |

|  |  |
| --- | --- |
| mean\_x(self, x) | source code |

|  |  |
| --- | --- |
| variance\_x(self, x) | source code |

|  |  |
| --- | --- |
| mean\_entropy(self)  Calculate the average entropy of probabilities sampled from this Dirichlet distribution. | source code |

|  |  |
| --- | --- |
| variance\_entropy(self)  Calculate the variance of the Dirichlet entropy. | source code |

|  |  |
| --- | --- |
| mean\_relative\_entropy(self, pvec) | source code |

|  |  |
| --- | --- |
| variance\_relative\_entropy(self, pvec) | source code |

|  |  |
| --- | --- |
| interval\_relative\_entropy(self, pvec, frac) | source code |

**Inherited from `object`**:
`__delattr__`,
`__getattribute__`,
`__hash__`,
`__new__`,
`__reduce__`,
`__reduce_ex__`,
`__repr__`,
`__setattr__`,
`__str__`


Properties

**Inherited from `object`**:
`__class__`


Method Details

|  |  |
| --- | --- |
| \_\_init\_\_(self, alpha)  *(Constructor)* | source code |

```
Args:
    - alpha  -- The parameters of the Dirichlet prior distribution.
                A vector of non-negative real numbers.
```

Overrides: object.\_\_init\_\_

|  |  |
| --- | --- |
| sample(self) | source code |

```
Return a randomly generated probability vector.

Random samples are generated by sampling K values from gamma
distributions with parameters a=lpha_i, b=1, and renormalizing. 

Ref:
    A.M. Law, W.D. Kelton, Simulation Modeling and Analysis (1991).
Authors:
    Gavin E. Crooks <gec@compbio.berkeley.edu> (2002)
```

|  |  |
| --- | --- |
| mean\_entropy(self) | source code |

```
Calculate the average entropy of probabilities sampled
from this Dirichlet distribution. 

Returns:
    The average entropy.
    
Ref:
    Wolpert & Wolf, PRE 53:6841-6854 (1996) Theorem 7
    (Warning: this paper contains typos.)
Status:
    Alpha
Authors:
    GEC 2005
```

|  |  |
| --- | --- |
| variance\_entropy(self) | source code |

```
Calculate the variance of the Dirichlet entropy. 

Ref:
    Wolpert & Wolf, PRE 53:6841-6854 (1996) Theorem 8
    (Warning: this paper contains typos.)
```

  


| Home | Trees | Indices | Help | WebLogo |
| --- | --- | --- | --- | --- |

|  |  |
| --- | --- |
| Generated by Epydoc 3.0.1 on Mon Jan 30 17:27:18 2012 | http://epydoc.sourceforge.net |
