# Supplementary material for: DIVERSITY in binding, regulation, and evolution revealed from high-throughput ChIP
Source: PLoS Comput Biol. 2018 Apr 23;14(4):e1006090. doi: 10.1371/journal.pcbi.1006090 (PMC5933800; doi:10.1371/journal.pcbi.1006090)
Supplement: S1 File — (GZ) [file pcbi.1006090.s009.tar.gz › DIVERSITY-master/weblogoMod/apidocs/weblogolib.logomath.Gamma-class.html]

xml version="1.0" encoding="ascii"?


weblogolib.logomath.Gamma


| Home | Trees | Indices | Help | WebLogo |
| --- | --- | --- | --- | --- |

Package weblogolib ::
Module logomath ::
Class Gamma

# Class Gamma

source code

```
object --+
         |
        Gamma
```

---

```
The gamma probability distribution. (Not to be confused with the
gamma function.)
```


Instance Methods

|  |  |
| --- | --- |
| \_\_init\_\_(self, alpha, beta)  x.\_\_init\_\_(...) initializes x; see x.\_\_class\_\_.\_\_doc\_\_ for signature | source code |

|  |  |
| --- | --- |
| mean(self) | source code |

|  |  |
| --- | --- |
| variance(self) | source code |

|  |  |
| --- | --- |
| sample(self) | source code |

|  |  |
| --- | --- |
| pdf(self, x) | source code |

|  |  |
| --- | --- |
| cdf(self, x) | source code |

|  |  |
| --- | --- |
| inverse\_cdf(self, p) | source code |

**Inherited from `object`**:
`__delattr__`,
`__getattribute__`,
`__hash__`,
`__new__`,
`__reduce__`,
`__reduce_ex__`,
`__repr__`,
`__setattr__`,
`__str__`


Class Methods

|  |  |
| --- | --- |
| from\_shape\_scale(cls, shape, scale) | source code |

|  |  |
| --- | --- |
| from\_mean\_variance(cls, mean, variance) | source code |


Properties

**Inherited from `object`**:
`__class__`


Method Details

|  |  |
| --- | --- |
| \_\_init\_\_(self, alpha, beta)  *(Constructor)* | source code |

```
x.__init__(...) initializes x; see x.__class__.__doc__ for signature
```

Overrides: object.\_\_init\_\_: *(inherited documentation)*

  


| Home | Trees | Indices | Help | WebLogo |
| --- | --- | --- | --- | --- |

|  |  |
| --- | --- |
| Generated by Epydoc 3.0.1 on Mon Jan 30 17:27:18 2012 | http://epydoc.sourceforge.net |
