# Supplementary material for: DIVERSITY in binding, regulation, and evolution revealed from high-throughput ChIP
Source: PLoS Comput Biol. 2018 Apr 23;14(4):e1006090. doi: 10.1371/journal.pcbi.1006090 (PMC5933800; doi:10.1371/journal.pcbi.1006090)
Supplement: S1 File — (GZ) [file pcbi.1006090.s009.tar.gz › DIVERSITY-master/weblogoMod/apidocs/weblogolib.LogoOptions-class.html]

xml version="1.0" encoding="ascii"?


weblogolib.LogoOptions


| Home | Trees | Indices | Help | WebLogo |
| --- | --- | --- | --- | --- |

Package weblogolib ::
Class LogoOptions

# Class LogoOptions

source code

```
object --+
         |
        LogoOptions
```

---

```
A container for all logo formatting options. Not all of these
are directly accessible through the CLI or web interfaces. 

To display LogoOption defaults:
>>> from weblogolib import *
>>> LogoOptions()

All physical lengths are measured in points. (72 points per inch, 28.3 points per cm)
  
String attributes:
    o creator_text      -- Embedded as comment in figures.
    o logo_title             
    o logo_label
    o unit_name         -- See std_units for options. (Default 'bits') 
    o yaxis_label       -- Defaults to unit_name      
    o xaxis_label
    o fineprint         -- Defaults to WebLogo name and version
    
Boolean attributes:
    o show_yaxis 
    o show_xaxis         
    o show_ends 
    o show_fineprint        
    o show_errorbars    -- Draw errorbars (default: False)
    o show_boxes        -- Draw boxes around stack characters (default: True)
    o debug             -- Draw extra graphics debugging information. 
    o rotate_numbers    -- Draw xaxis numbers with vertical orientation? 
    o scale_width       -- boolean, scale width of characters proportional to ungaps
    o pad_right         -- Make a single line logo the same width as multiline logos (default: False)                           
                            
Other attributes:
    o stacks_per_line
    
    o yaxis_tic_interval 
    o yaxis_minor_tic_ratio 
    o yaxis_scale
    o xaxis_tic_interval 
    o number_interval

    o shrink_fraction       -- Proportional shrinkage of characters if show_boxes is true.

    o errorbar_fraction 
    o errorbar_width_fraction 
    o errorbar_gray 

    o resolution             -- Dots per inch (default: 96). Used for bitmapped output formats
    
    o default_color 
    o color_scheme 
    
    o stack_width           --
    o stack_aspect_ratio    -- Ratio of stack height to width (default: 5)

    o logo_margin           -- Default: 2 pts
    o stroke_width          -- Default: 0.5 pts
    o tic_length            -- Default: 5 pts
    o stack_margin          -- Default: 0.5 pts
    
    o small_fontsize        -- Small text font size in points
    o fontsize              -- Regular text font size in points
    o title_fontsize        -- Title text font size in points
    o number_fontsize       -- Font size for axis-numbers, in points.
    
    o text_font
    o logo_font
    o title_font
    
    o first_index
    o logo_start
    o logo_end
```


Instance Methods

|  |  |
| --- | --- |
| \_\_init\_\_(self, \*\*kwargs)  Create a new LogoOptions instance. | source code |

|  |  |
| --- | --- |
| \_\_repr\_\_(self)  repr(x) | source code |

**Inherited from `object`**:
`__delattr__`,
`__getattribute__`,
`__hash__`,
`__new__`,
`__reduce__`,
`__reduce_ex__`,
`__setattr__`,
`__str__`


Properties

**Inherited from `object`**:
`__class__`


Method Details

|  |  |
| --- | --- |
| \_\_init\_\_(self, \*\*kwargs)  *(Constructor)* | source code |

```
Create a new LogoOptions instance.

>>> L = LogoOptions(logo_title = "Some Title String")
>>> L.show_yaxis = False
>>> repr(L)
```

Overrides: object.\_\_init\_\_

|  |  |
| --- | --- |
| \_\_repr\_\_(self)  *(Representation operator)* | source code |

```
repr(x)
```

Overrides: object.\_\_repr\_\_: *(inherited documentation)*

  


| Home | Trees | Indices | Help | WebLogo |
| --- | --- | --- | --- | --- |

|  |  |
| --- | --- |
| Generated by Epydoc 3.0.1 on Mon Jan 30 17:27:18 2012 | http://epydoc.sourceforge.net |
